# The bismuth phosphanides Bi(PR_2_)_3_: sources of phosphanyl radicals, P-inversion, and reversible olefin insertion

**DOI:** 10.1039/d5sc07240a

**Published:** 2026-01-08

**Authors:** Sascha Reith, Kai Oberdorf, Sebstián Martínez, Jann B. Landgraf, Alena Ahrens, Felix Jakobi, Xiulan Xie, Crispin Lichtenberg

**Affiliations:** a Department of Chemistry, Philipps-University Marburg Hans-Meerwein-Str. 4 D-35032 Marburg (D) Germany crispin.lichtenberg@chemie.uni-marburg.de; b Department of Inorganic Chemistry, Julius-Maximilians-Universität Würzburg Am Hubland D-97074 Würzburg (D) Germany

## Abstract

Understanding and controlling radical reactions for selective transformations under mild conditions remains one of the key challenges in synthetic chemistry. The detailed investigation of previously inaccessible structural motifs can grant important insights, provide new stimuli, and offer innovative strategies for further developments in the field. In this respect, the controlled release of simple phosphanyl radicals [PR_2_]˙ from metal precursors is significantly underdeveloped to date. Here we report the synthesis, isolation, and full characterization of a series of homoleptic parent bismuth phosphanides [Bi(PRR’)_3_] (R, R’ = alkyl). The ability of these compounds to release phosphanyl radicals under mild conditions is demonstrated, revealing an unprecedented radical pathway for the inversion of phosphorus atoms, enabling ethylene activation, and facilitating reversible olefin insertion into Bi–P bonds.

## Abstract 2

The isolation and characterization of the first series of homoleptic bismuth phosphanides aBi(PRR’)_3_ is presented (R, R’ = alkyl). Radical pathways enable phosphorus inversion and the (reversible) activation of α-olefins, including ethylene.

## Graphical abstract

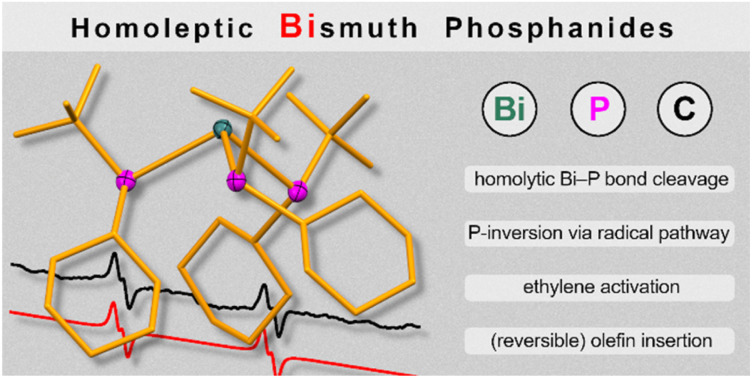

## Introduction

The exploration of simple, homoleptic complexes forms the basis for the profound understanding of principal classes of compounds. Such fundamental studies grant important insights into the intrinsic properties and reactivity patterns of the structural motif under investigation and can often be extrapolated to its behavior in more complex molecular frameworks. Among the various functional groups known in coordination chemistry, phosphanides, *i.e.* compounds featuring an M–PR_2_ bond (M = metal atom, R=H, alkyl, aryl) open up unusual pathways in CH activation and small molecule activation and play key roles in stoichiometric and catalyzed P–P and P–C bond formation as well as olefin hydrogenation reactions.^1–12^ In addition, unexpected selectivities in insertion reactions and unusual spectroscopic features such as intermolecular through-space spin–spin coupling have been reported.^13,14^ Key questions in the understanding of the M–PR_2_ structural motif center around the potential M–P multiple bond character,^15–18^ the inversion of the phosphorus atom,^18–21^ and potential radical character of the PR_2_ ligand in the coordination sphere of a transition metal.^22^ The design of reactive metal phosphanide complexes [M]–PR_2_ is an intriguing, but yet underdeveloped strategy for the release of reactive phosphanyl radicals under mild conditions to be utilized in selective intermolecular reactions.^23–27^ In this context, bismuth compounds appear as very promising candidates due to their typically low homolytic Bi–X bond dissociation energies (*e.g.*: X = C, N, O, Bi).^28–40^

**Scheme 1:**
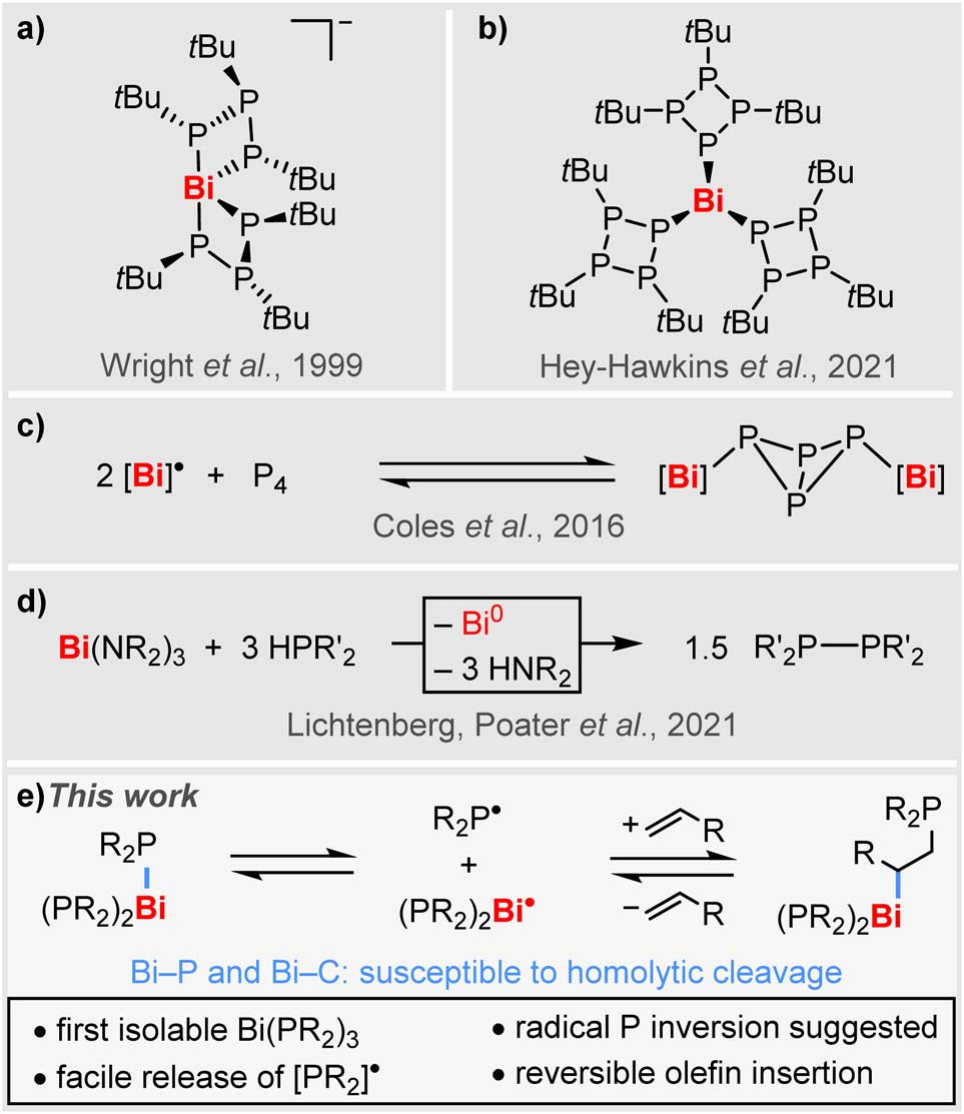
(a and b) Homoleptic bismuth compounds with phosphorus-based ligands. (c) Reaction of isolable radical [Bi]˙ wiht P_4_; [Bi] = Bi(NDippSiMe_2_)_2_O. (d) Bismuth-mediated P–P coupling *via* suggested radical intermediates (R, R’ = alkyl, aryl). (e) This work: simple homoleptic Bi(PR_2_)_3_ for [PR_2_]˙ release, radical P-inversion, and reversible olefin insertion (R = alkyl).

However, compounds featuring Bi–P bonds are rare and investigations have mostly been focused on the synthesis and structure elucidation of heteroleptic species.^41–51^ Well-defined homoleptic bismuth phosphanides have so far been limited to a very small number of special cases. For instance, the serendipitously generated complex anion [Bi(P_3_*t*Bu_3_)_2_]^−^ could be structurally characterized (Scheme 1a), but its high sensitivity precluded a satisfactory spectroscopic characterization.^52^ More recently, the neutral species [Bi(P_4_*t*Bu_3_)_3_] with its unusual phosphacyclic ligand motif has been reported (Scheme 1b), revealing a pronounced photosensitivity,^53^ while selective reactivity patterns remain to be explored. In a broader context, the reversible addition of isolable Bi(ii) radical species to P_4_ has been reported on a single instance and selective P–P bond formation from suggested [Bi]–PR_2_ intermediates has recently been observed (Scheme 1c and d).^30,41^ However, there are no examples of simple homoleptic bismuth phosphanides Bi(PR_2_)_3_, which could act as isolable sources of phosphanyl radicals [PR_2_]˙ (R = alkyl).

Here we report the synthesis, isolation, and full characterization of the first simple homoleptic bismuth phospanides, [Bi(PR_2_)_3_], uncovering the facile release of phosphanyl radicals [PR_2_]˙, suggesting an unprecedented mechanism for phosphorus inversion, and demonstrating the reversible insertion of unactivated α-olefins into Bi–P bonds involving BiC/BiP homolysis (Scheme 1e).

## Results and discussion

**Scheme 2:**
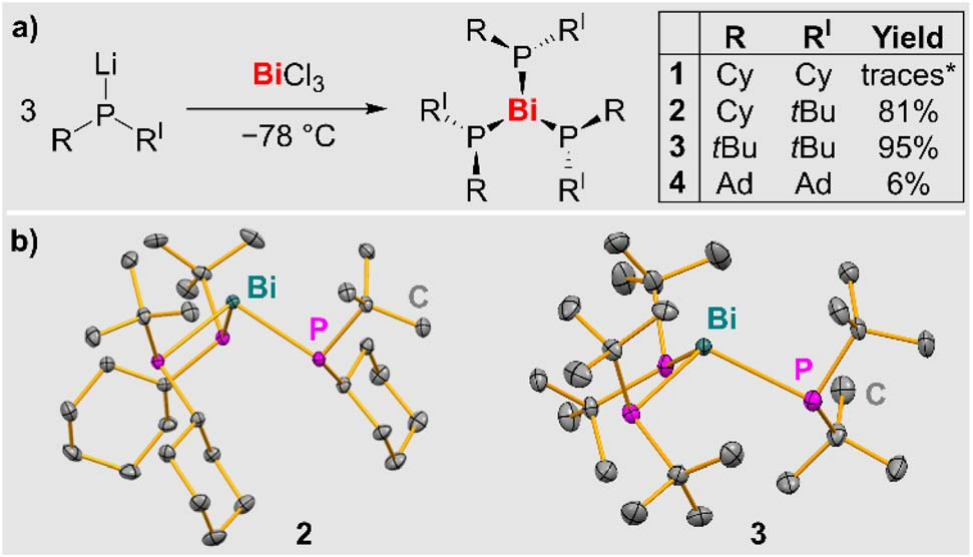
(a) Synthetic access to bismuth phosphanides 1–4 (Cy = cyclohexyl; Ad = adamantyl); *: small amounts of single-crystals confirmed the formation of 1, but a yield could not be determined due its instability. (b) Molecular structures of 2 and 3 in solid state. Displacement ellipsoids are shown at the 50% probability level. Hydrogen atoms are omitted for clarity. Selected bond lengths [Å] and angles [°]: 2: Bi–P1, 2.6605(5); Bi–P2, 2.6709(6); Bi–P3, 2.6484(7); P1–Bi–P2, 98.902(17); P2–Bi–P3, 99.515(19); P2–Bi–P3, 97.168(19). 3: Bi–P1, 2.7011(9); Bi–P2, 2.6971(9); Bi–P3, 2.6890(9); P1–Bi–P2, 106.87(3); P2–Bi–P3, 105.93(3); P2–Bi–P3, 104.11(3).

Straightforward access to the first simple bismuth phosphanides Bi(PR_2_)_3_ was granted *via* salt elimination strategies (R = alkyl; Scheme 2a). Balancing dispersion interactions and steric congestion proved to be a decisive factor in order to suppress thermal decomposition pathways in this class of compounds and to sufficiently stabilize the target species. Hints at the accessibility of compounds Bi(PR_2_)_3_ could be obtained through single-crystal XRD data on Bi(PCy_2_)_3_ (1), which eluded a detailed characterization in solution due to its thermal instability (*vide infra* and SI). By increasing the steric bulk of the substituents at the phosphorus atom, compounds Bi(P*t*BuCy)_3_ (2), Bi(P*t*Bu_2_)_3_ (3), and Bi(PAd_2_)_3_ (4) could be isolated, representing the first examples of simple homoleptic bismuth phosphanides that could be characterized in detail (Cy = cyclohexyl, Ad = adamantyl). Multiple recrystallization steps were required to obtain compound 4 in pure form leading to low isolated yields, but facile, high-yielding multi-gram syntheses could be realized for compounds 2 and 3, respectively. Compounds 2–4 were obtained as intensely colored orange (2) to deep-red (3, 4) solids. Aryl groups at the phosphorus atom increased the lability of the targeted homoleptic bismuth phosphanides (SI).

### Crystallographic characterization of compounds 2 and 3

Single-crystal XRD analysis of 2 and 3 (triclinic space group *P*1̄) confirmed the expected trigonal pyramidal coordination geometry around the central atom (Scheme 2b). The molecular structures show an apparent *C*_3_ axis which runs through the Bi atom and is orthogonal to the plane defined by the three phosphorus atoms. For each phosphorus atom, one of the organic substituents points towards the bismuth atom, while the other one points away from it. In the case of compound 2, it is important to note that each phosphorus atom represents a stereocenter, *i.e.* in the solid state, a racemic mixture of the (*R*,*R*,*R*)- and the (*S*,*S*,*S*)-enantiomer is realized. The bond angles in tri-coordinate bismuth(iii) compounds commonly approach 90°, because the 6 s(Bi) orbital tends not to contribute significantly to bond formation in these compounds due to hybridization defects.^54,55^ In compound 2, this situation is reflected by an angle sum of 295.6° around bismuth. In contrast, the higher steric pressure of the *t*Bu groups in 3 (*vs.* Cy groups in 2) induces a remarkably large angle sum of 316.9° around the central atom. This is also reflected by the Bi–P bond lengths in 2 and 3, which range from 2.6484(7) to 2.7011(9) Å and are on average 0.04 Å longer for the bulkier species 3. In the case of compound 1, data from single-crystal X-ray diffraction experiments could be obtained, but suffered from poor quality due to higher twinning of the crystal. Thus, a discussion of the bonding parameters is not possible, but the connectivity is definite (for details, see SI).

### NMR spectroscopic analysis

**Scheme 3:**
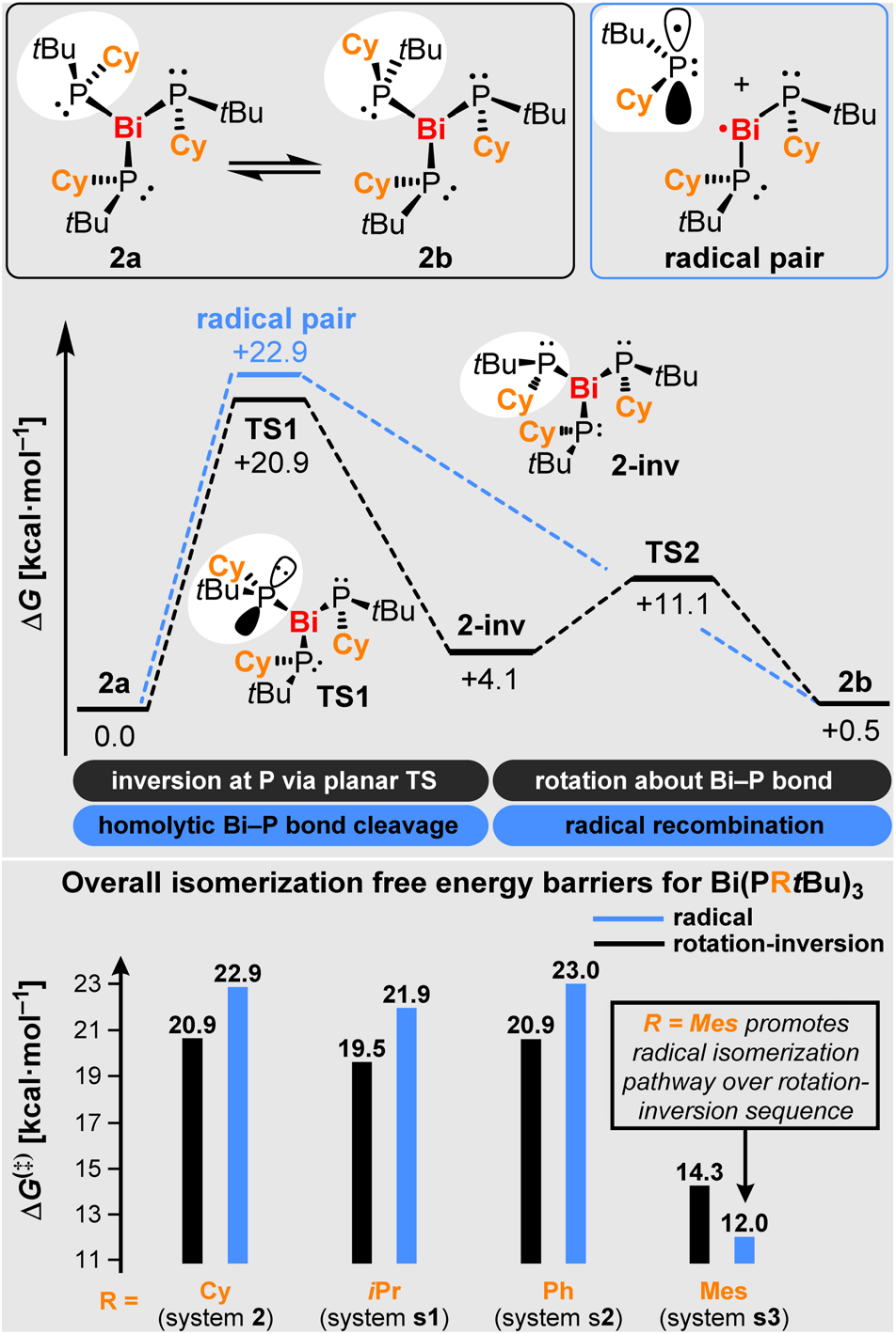
Theoretical analyses. Top: isomerization of compounds 2a and 2b (top left), along with mechanistic considerations proposing viable reaction pathways. Bottom: overall isomerization barriers for radical *vs.* rotation-inversion sequence for systems 2, s1, s2 and s3. Energies were determined by DFT calculations (see discussion and SI).

NMR spectroscopic investigations of compounds 2–4 in solution indicate the formation of typical molecular complexes without significant intermolecular interactions. The ^31^P NMR spectroscopic chemical shifts cover a relatively broad range of 42.4–86.4 ppm. For compounds 3 and 4 with their symmetrically substituted phosphorus atoms, one set of signals is observed in the solution NMR spectra, indicating that the bonding situation that has been found for 3 in the solid state is preserved in solution. In contrast, two sets of signals are observed for compound 2 with its unsymmetrically substituted phosphorus atoms. In the ^31^P NMR spectrum, one singlet is ascribed to compound 2a, which resembles the bonding situation found in the solid state (Scheme 3, top left). The second set of signals consists of three resonances of identical intensity, with ^2^*J*_PP_ coupling constants being barely resolved. This set of signals is ascribed to the diastereomer 2b, which can formally be obtained from 2a by the inversion of one phosphorus center followed by a 180° rotation about the respective Bi–P bond. Note that 2b shows three chemically inequivalent phosphorus atoms, since the C_3_ symmetry element that is present in 2a is no longer present in 2b due to the inversion of the configuration at one P atom. Variable temperature NMR spectroscopic analysis in the range of −80 to +80 °C reveal that the relative intensities of the resonances ascribed to 2a and 2b are reversibly shifted from 2a:2b = 1.00/0.93 at −80 °C *via*2a:2b = 1.00/1.09 at +25 °C to 2a:2b = 1.00/1.21 at +80 °C (concomitant decomposition is observed at elevated temperatures, but does not interfere with these analyses; *vide infra*). This translates into thermodynamic parameters of Δ*G*(293 K) = −0.027 kcal mol^−1^, Δ*H* = +0.50 kcal mol^−1^, and Δ*S* = +1.8 cal mol^−1^ for the isomerization process 2a⇌2b, as determined from a van't Hoff plot (SI). Dynamic exchange between 2a and 2b was further supported by ^31^P–^31^P EXSY NMR spectroscopic experiments (SI). From a mechanistic point of view, two classical scenarios have been encountered for the inversion of phosphorus centers:^56,57^ i) a planar transition state in the absence of electron-withdrawing substituents (with barriers commonly ranging from *ca.* 30–40 kcal mol^−1^ for trialkyl phosphanes^58–60^) and ii) a T-shaped transition state in the presence of electron-withdrawing groups at the phosphorus atom (with a calculated barrier of 53.8 kcal mol^−1^ for PF_3_).^61,62^

### Computational studies

In order to gain insights into the isomerization mechanism, we performed computational studies. In a first approach, the thermodynamic stability of relevant isomers of 2 was evaluated. Our study shows that isomers 2a and 2b (Scheme 3) present similar thermodynamic stabilities (Δ*G*_b−a_ = +0.5 kcal mol^−1^), which agrees with the experimental detection of two isomers of 2. Isomers 2c and 2d represent complexes with different relative orientations of the *t*Bu and Cy groups (see SI) and are predicted to be less stable, presenting Δ*G* values that are > 8.0 kcal mol^−1^ higher than that of 2a, and are therefore unlikely to be detected experimentally. Next, we focused on investigating the possible isomerization pathways for the interconversion of 2a and 2b. A sequence of inversion-rotation *via*TS1 and TS2, was found to be the most plausible reaction pathway, which accounts for an overall free energy barrier of +20.9 kcal mol^−1^ (Scheme 3; for correlation with experimental data from an Eyring plot see SI). Notably, a radical isomerization pathway involving homolytic Bi–P bond dissociation and re-association, presents a barrier that is only 2.0 kcal mol^−1^ higher in energy than the former pathway, suggesting that both mechanisms could be competing, especially at elevated temperature due to the positive entropy term in the barrierless homolytic bond splitting.^63^ A crossing point for the two pathways was predicted at a temperature of 334 K based on DFT calculations. In order to evaluate the impact of the substituents at the phosphorus atoms on the isomerization process, the analogous systems Bi(P*t*BuR)_3_ were studied computationally (R = *i*Pr (s1), Ph (s2), Mes (s3); Scheme 3 (bottom) and SI). Our results show that an inversion-rotation sequence is slightly preferred for s1 and s2. Remarkably, s3 is predicted to favor isomerization *via* the newly proposed radical pathway, even at ambient temperature.

### EPR spectroscopic investigations

**Scheme 4:**
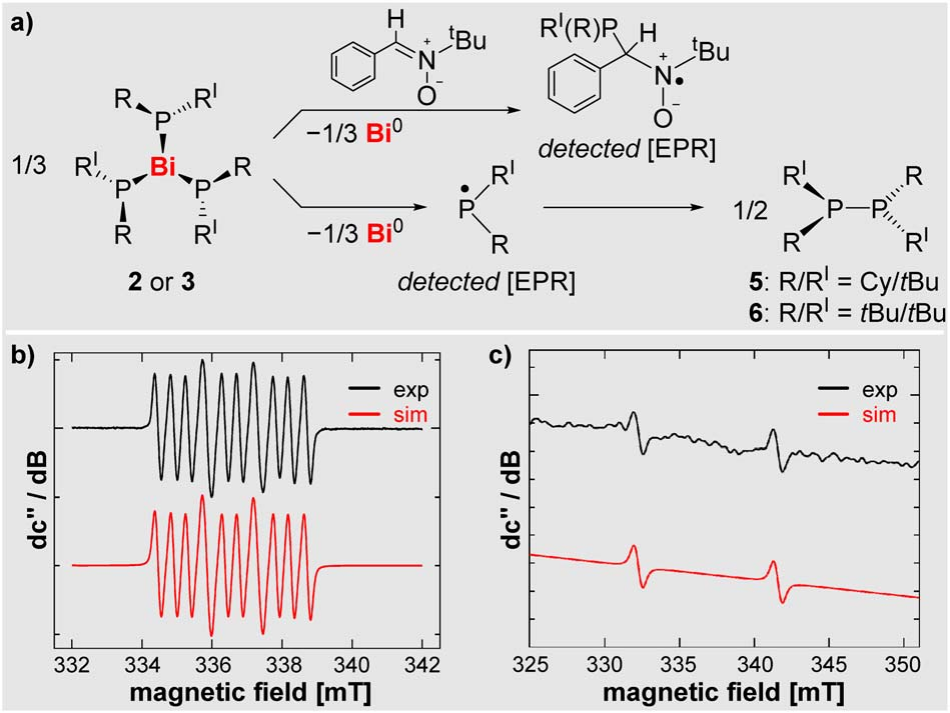
(a) Bi–P bond homolysis to give the corresponding phosphanyl radical in the presence (top) and absence (bottom) of PBN. (b) X-band EPR spectrum of the (P*t*Bu_2_)˙ radical (starting from 3 (*c* = 0.121 mol L^−1^)) trapped by PBN (3 equiv. used) in THF at room temperature; *g*_iso_ = 2.0052; coupling constants: a(^14^N) = 1.46 mT, a(^31^P) = 9.32 mT, a(^1^H) = 0.455 mT. (c) X-band EPR spectrum of the free (P*t*Bu_2_)˙ radical (starting from 3 (*c* = 0.0132 mol L^−1^))in toluene at 70 °C; *g*_iso_ = 2.0058; coupling constant: a(^31^P) = 9.32 mT.

In view of the low calculated Bi–P bond dissociation energy in compounds 2 (22.9 kcal mol^−1^) and 3 (18.3 kcal mol^−1^), these compounds were investigated by EPR spectroscopy. Indeed, resonances of the free phosphanyl radicals, (PCy*t*Bu)˙ and (P*t*Bu_2_)˙, could be detected, when monitoring toluene solutions of compounds 2 and 3 at elevated temperatures of 95 °C and 70 °C, respectively, in the cavity of the EPR spectrometer (Scheme 4, for details see SI). To the best of our knowledge, this is the first example of the direct detection of simple dialkylphosphanyl radicals in typical wet-chemical approaches without the use of stabilization strategies such as chelation or excessive bulk.^64,65^ The spectroscopic signature of reactive [PR*t*Bu]˙ radicals reveals large coupling constants of a(^31^P) = 9.31 mT. This is in congruency with data reported for an isolable sterically protected dialkylphosphanyl radical [P(C(SiMe_3_)_2_CH_2_)_2_]^•^ (a(^31^ P) = 9.07 mT).^64^

When adding the spin trap PBN (phenyl-*N-t*-butylnitrone) to solutions of 2 and 3, the corresponding radicals could readily be detected at ambient temperature (Scheme 4 and SI).

The facile entrance into radical chemistry provided by compounds 2 and 3 motivated the closer investigation of their solution behavior. In benzene solution at 25 °C, these compounds selectively form the corresponding diphosphanes Cy*t*BuP–P*t*BuCy (5) and (*t*Bu)_2_P–P(*t*Bu)_2_ (6) along with Bi^0^, leading to half-life times of 15.3 d and 9.8 d for compounds 2 and 3, respectively. At elevated temperatures of 110 °C (for 2) and 65 °C (for 3) or upon irradiation of the reaction mixture with an LED (*λ* = 365 nm), near-quantitative yields are obtained in 15 min (for 5 and 6).

### H-atom abstractions reactions

**Scheme 5:**
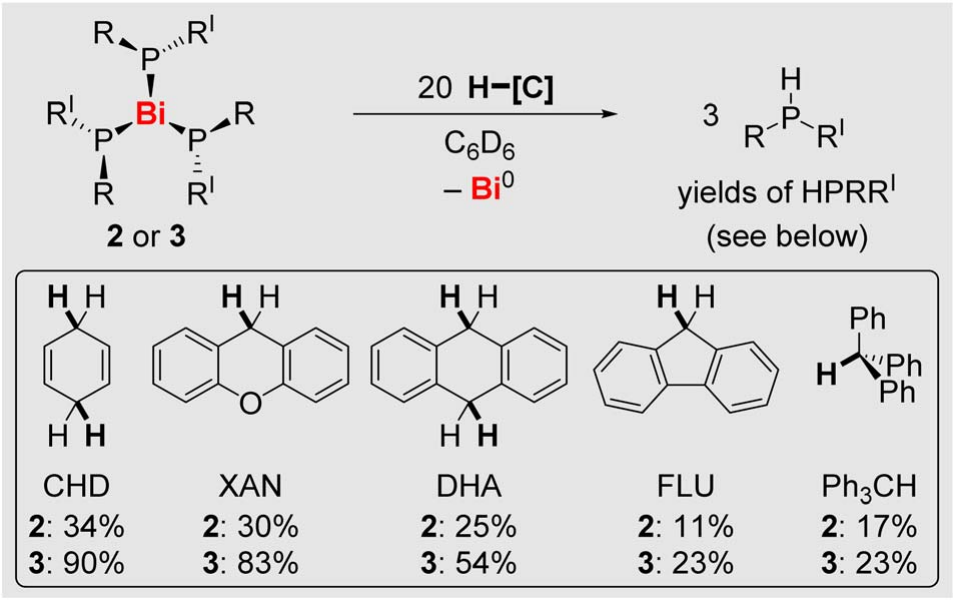
Reactions of 2 and 3 with H-atom donors (that require the scission of a C–H bond) to the corresponding phosphanes. Yields are given after 43 h reaction time (for details see SI).

In order to evaluate the reactivity of 2 and 3 towards external substrates, reactions with a series of hydrocarbon-based H-atom donors were performed. While H-atom transfer to the phosphorus atom to give phosphanes HPR_2_ was feasible in both cases, it was more effective for compound 3, the formation of diphosphanes 5 or 6 being the competing transformation (Scheme 5 and SI). For compound 3, H-atom transfer proved to be the preferred reaction pathway for substrates with a C–H bond dissociation free energy of up to 75.7 kcal mol^−1^. Thus, balancing the steric bulk of the substituents at the phosphorus atoms appears to be crucial: a sufficient bulk is essential to grant access to these compounds (*cf*. compound 1*vs.* compound 2), but further increasing the steric bulk in small increments quickly enhances the reactivity due to sterically promoted Bi–P bond homolysis (*cf*. compound 2*vs.*3).

### Reactivity as a phosphanyl radical precursor

**Scheme 6:**
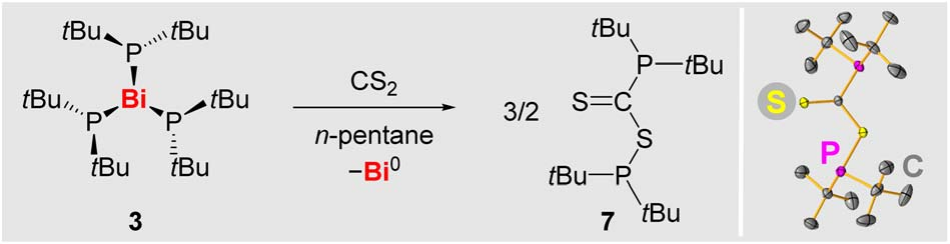
Left: reaction of 3 with CS_2_ to give 7. Right: molecular structure of 7 in the solid state as determined by single-crystal X-ray diffraction analysis (for details see SI).

The diphospane R_2_P–PR_2_ (R = NDippCH_2_) has been reported to readily insert CS_2_ into its P–P bond *via* a radical pathway, involving [PR_2_]˙.^66^ In contrast, compounds (alkyl)_2_P–P(alkyl)_2_ do not readily release phosphanyl radicals [P(alkyl)_2_]˙ under moderate conditions, as confirmed by EPR spectroscopic experiments with isolated Cy*t*BuP–P*t*BuCy (5) and (*t*Bu)_2_P–P(*t*Bu)_2_ (6) (SI). In view of the ability of 3 to release phosphanyl radicals, its reactivity towards the potential phosphanyl radical scavenger CS_2_ was probed (Scheme 6). Indeed, the addition of CS_2_ (2.2 equiv.) to a solution of 3 led to an immediate color change from dark red *via* dark violet to smaragd green, along with the precipitation of a dark solid (presumably Bi^0^). After workup, compound 7 was isolated as a dark green solid in 92% yield and fully characterized (SI). This demonstrates the ability of 3 to transfer phosphanyl radicals to external substrates.

### Reactivity towards olefins

**Scheme 7:**
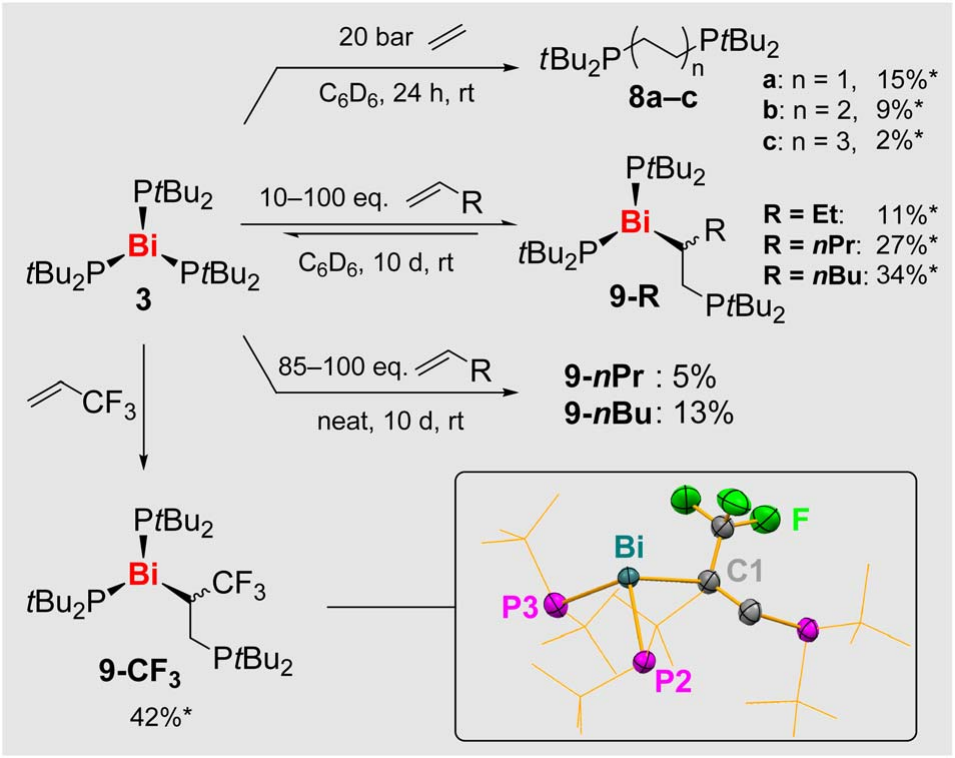
Reactivity of 3 towards olefines, namely: ethylene; the chemical equilibrium between 3 and 1-butene, 1-pentene and 1-hexene; reactions under neat conditions; the insertion of 3,3,3-trifluoropropene and the molecular structure of 9-CF_3_ in solid state, the displacement ellipsoids being shown at the 50% probability level. Hydrogen atoms are omitted, and the *t*Bu units are shown as wireframe for clarity. Selected bond lengths [Å] and angles [°]: 9-CF_3_: Bi–P2, 2.6796(15); Bi–P3, 2.6881(16); Bi–C1, 2.382(6); C1–C2, 1.501(8); C1–C3, 1.488(8); P2–Bi–P3, 105.18(5); P2–Bi–C1, 95.66(14); P3–Bi–C1, 106.79(17). Yields marked with * were determined *via*^31^P NMR spectroscopy.

With compound 3 as a promising candidate, we conducted initial reactivity studies towards olefins including ethylene, a benchmark substrate in the field.^67–69^ Reactions of 3 with ethylene yielded mixtures of compounds *t*Bu_2_P(C_2_H_4_)_n_P*t*Bu_2_ (8a–c) suggesting that up to three consecutive olefin insertion reactions into Bi–P/Bi–C bonds as well as reductive elimination processes can take place (Scheme 7).^70–72^ Reactions with non-activated terminal alkenes H_2_C = CHR (R = Et, *n*Pr, *n*Bu) were monitored by ^31^P NMR spectroscopy, showing one new set of resonances each, which was ascribed to the mono-insertion products (*t*Bu_2_P)_2_Bi–(C_2_H_3_R)P*t*Bu_2_ (9-R, Scheme 7). These reactions were strongly impeded by the exclusion of ambient light.

Irradiation of the reaction mixtures with LEDs (*λ* = 365–525 nm) increased the rates of reactions, but even in the most promising cases (*λ* = 525 nm), the selectivity towards the insertion product was only improved at early stages of the reaction (32% spectroscopic yield of 9-*n*Bu after 2 h), when significant amounts of unreacted and inseparable 3 were still present (for details see SI).

**Scheme 8:**
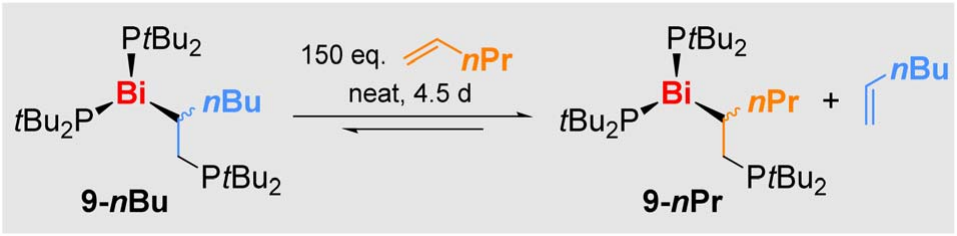
Crossover experiment between 8-*n*Bu and 1-pentene (H_2_C = CH*n*Pr), demonstrating the reversibility of olefin insertion with Bi(PtBu_2_)_3_*via* Bi–P and Bi–C bond cleavage/formation.

In stoichiometric reactions of 3 with 1-pentene and 1-hexene under ambient light, only partial conversion of the starting material 3 was observed, which was accompanied by its slow degradation to give the diphosphane 6 (*vide supra*). This led to the initial hypothesis of an equilibrium reaction, which could be confirmed: reacting 3 with neat 1-pentene or 1-hexene followed by a tedious work-up to remove the side-product 6 led to the isolation of yellow powders of 9-*n*Pr and 9-*n*Bu, which were pure by ^31^P NMR spectroscopy, when using the respective olefin as the solvent. When other solvents were applied, mixtures of 3, 6, and 9-R were obtained, *i.e.* the starting material 3 is partially regenerated when there is no excess olefin present in solution. In good agreement with these findings, compounds 9-R proved to be also vacuum-sensitive, hampering a detailed characterization of these compounds to date. Furthermore, a crossover experiment was performed: dissolving 9-*n*Bu in 1-pentene led to its slow conversion to 9-*n*Pr (and *vice versa*), confirming the reversible insertion of α-olefins into Bi–P bonds (Scheme 8).

When it comes to the utilization of reactive radical intermediates for synthetic purposes, the reversibility of homolytic bond dissociation reactions can be crucial. For literature-known, carbon-centered radicals generated by reversible Bi–C homolysis, this has led to unprecedented catalytic applications in olefin polymerization, C–N coupling, and radical cyclo-isomerization.^29,31,32,71,72^ The key steps in such synthetic applications have been focused on the reversible homolytic Bi–X bond dissociation of one type of Bi–X bond (typically a Bi–C bond). Here we show that the peerless release of [PR_2_]˙ radicals under mild reaction conditions (tied to reversible Bi–P bond cleavage/formation) can be combined with reversible Bi–C bond formation, an important step towards greater functional group diversity in controlled radical reactions.

A more robust olefin insertion product 9-CF_3_ could be obtained from the reaction of 3 with electron-deficient 3,3,3-trifluoropropene (Scheme 7, bottom). In contrast to the other insertion products 9-R, compound 9-CF_3_ did not release the previously inserted olefin, when dissolved in common organic solvents such as benzene, which can be ascribed to the more polar nature of the Bi–C bond in 9-CF with its electron-withdrawing CF_3_ group at the bismuth-bound carbon atom. ^31^P NMR spectroscopy indicated the presence of only one phosphorus compound 9-CF_3_ besides traces (<2%) of diphosphane 6. However, the ^1^H NMR spectrum showed additional broadened resonances in the aliphatic region, which was ascribed to lower oligomers of 3,3,3-trifluoropropene. This was in agreement with an oily residue that co-precipitated with crystalline 9-CF_3_ and could not be separated due to similar solubility properties. The oligomeric nature of this side product was further confirmed by elemental analysis and high-resolution mass spectrometry, the latter also supporting the formation of 9-CF_3_. Despite the difficulties in isolating 9-CF_3_ in pure form, its molecular structure in the solid state could unambiguously be identified by single-crystal X-ray diffraction analyses (monoclinic space group *P*2_1_/*n*; *Z* = 4; Scheme 7 bottom). Compound 9-CF_3_ forms a typical mononuclear species with a trigonal pyramidal coordination geometry around the bismuth atom (angle sum around Bi, 307°). Notably, the introduction of the secondary alkyl group as a bismuth-bound ligand increases the pyramidalization of the central atom (as compared to 3), with two large (P1–Bi–P2/C1, 105.2–106.8°) and one small angle (P2–Bi–C1, 95.7°) around the central atom. The newly formed Bi–C bond (2.38 Å) is exceptionally long compared to Bi–C bonds in more traditional motifs,^46,47,73–76^ which was ascribed to steric congestion around the central atom and the presence of the electron-withdrawing CF_3_ group at the α-C-atom.

## Conclusions

In summary we present the synthesis, isolation, and characterization of the first series of parent dialkyl bismuth phosphanides Bi(PRR’)_3_. This new class of compounds shows facile release of simple phosphanyl radicals [P(alkyl)_2_]˙. This enables the inversion of the bismuth-bound P atoms through a radical dissociation/re-association mechanism, adding a peerless alternative to the two well-established pathways for P-inversion *via* trigonal planar or T-shaped transition states. Reactivity studies on the title compounds Bi(PRR’)_3_ show that in the absence of external substrates, selective near-quantitative radical P–P coupling reactions dominate. The radical reactivity can be extended to external substrates: the radical transfer on CS_2_ and the insertion of ethylene and unactivated α-olefins into Bi–P bonds is facilitated, the latter proceeding in a reversible manner. This combines reversible Bi–P homolysis with reversible Bi–C homolysis for the first time, opening up perspectives for dual mode controlled radical reactions. It is anticipated that these fundamental investigations of a new class of compounds will stimulate research into selective stoichiometric and catalyzed radical reactions of bismuth phosphanide structural motifs [Bi]–PR_2_ embedded in supporting ligand scaffolds. Research along these lines is currently being pursued in our laboratories.

## Author contributions

The synthesis and the analysis of the compounds were conducted by SR and KO with the support of JBL, AA and FJ. X-Ray diffraction analyses were conducted by SR and KO. EPR analyses were performed and interpreted by CL. XX carried out EXSYX ^31^P–^31^P NMR experiments. DFT-calculations were conducted by SM and CL. The manuscript was drafted by SR and CL and was finalized with contributions from all authors.

## Conflicts of interest

There are no conflicts to declare.

## Supplementary Material

SC-OLF-D5SC07240A-s001

SC-OLF-D5SC07240A-s002

## Data Availability

Data supporting this article have been included as part of the supplementary information (SI). Supplementary information: experimental, analytical, and theoretical details. See DOI: https://doi.org/10.1039/d5sc07240a. CCDC 2451991–2451995 (1, 2, 3, 5, 9-CF_3_) and 2502129 (7) contain the supplementary crystallographic data for this paper.^77*a*–*f*^
