# Supplementary material for: The bismuth phosphanides Bi(PR2)3: sources of phosphanyl radicals, P-inversion, and reversible olefin insertion
Source: Chem Sci. 2026 Jan 8;17(10):5183–91. doi: 10.1039/d5sc07240a (PMC12815243; doi:10.1039/d5sc07240a)
Supplement: SC-017-D5SC07240A-s001 [file SC-017-D5SC07240A-s001.pdf]

Supplementary Information (SI) for Chemical Science.
This journal is © The Royal Society of Chemistry 2026

Supporting Information

The Bismuth Phosphanides $Bi(PR_2)_3$: Sources of Phosphanyl Radicals, P-Inversion, and Reversible Olefin Insertion

Sascha Reith,[a] Kai Oberdorf,[a] Sebstián Martínez,[a] Jann B. Landgraf,[b] Alena Ahrens,[a] Felix Jakobi,[a] Xiulan Xie,[a] and Crispin Lichtenberg*[a]

a. Department of Chemistry, Philipps-University Marburg, Hans-Meerwein-Str. 4, D-35032 Marburg (D), E-mail: crispin.lichtenberg@chemie.uni-marburg.de

b. Department of Inorganic Chemistry, Julius-Maximilians-Universität Würzburg, Am Hubland, D-97074 Würzburg (D)

Table of Contents

General considerations 2
Synthesis of [Bi(PCy$_2$)$_3$] (1) 3
Synthesis of [Bi(P(*t*Bu)Cy)$_3$] (2) 3
Synthesis of [Bi(P*t*Bu$_2$)$_3$] (3) 5
Synthesis of [Bi(PAd$_2$)$_3$] (4) 6
Aryl-substituted bismuth phosphanides 7
Temperature dependent ^{31}P{^{1}H} NMR measurements of 2 10
Eyring Plot 13
P–P coupling of the bismuth phosphanides 2 and 3 17
Reactivity as a phosphanyl radical precursor 21
Reactivity towards alkenes 22
Hydrogen atom transfer (HAT) reactivity 30
NMR spectra 37
UV/Vis spectra 45
Mass spectra 46
Cyclic voltammetry 58
Electron-paramagnetic-resonance spectroscopy (EPR) 60
Computational Studies 63
X-Ray diffraction analysis and molecular structures 77
Cartesian coordinates 84
References 140

General considerations

All manipulations were carried out in a glovebox or using Schlenk-type techniques under a dry argon atmosphere. Solvents were dried by continuous distillation over sodium metal or CaH_2 for several days, degassed via three freeze-pump cycles and stored over molecular sieves 4 Å. The ^{1}H, $^{1}H\{^{31}P\}$, and $^{13}C\{^{1}H\}$ NMR spectra were recorded on a *Bruker AV III 500* or *Bruker AV II 300* NMR spectrometer. Chemical shifts are reported in ppm relative to the residual proton signals of the solvent (for ^{1}H) or relative to the signal of the solvent itself (^{13}C). The $^{31}P\{^{1}H\}$ NMR spectra were recorded on a *Bruker AV III HD 250* and chemical shifts are reported in ppm relative to H_3PO_4. All NMR data was processed using the MestReNova software package. IR spectroscopic measurements were conducted on a *Bruker Alpha ATR-IR* spectrometer. Elemental analyses were performed by the service of the Chemistry Department of the Philipps University Marburg, Germany, using a CHN(S) analyzer *vario MICRO Cube* (*Elementar)*. $BiCl_3$, PCl_3, $AlCl_3$, $LiAlH_4$, $MgSO_4$, cyclohexyl chloride, *t*-butyl chloride, adamantane, *n*-butyl lithium (2.5 M in hexane), 3,3,3-trifluoropropene, 1-hexene, 1-pentene, 1,4-cyclohexadiene, xanthene, 9,10-dihydroanthracene, fluorene, triphenylmethane, and phenyl-*N*-*t*-butylnitrone were obtained commercially (Sigma-Aldrich, Acros, Strem, Alfa Aesar) and - if not noted otherwise - used after degassing. $BiCl_3$ and $AlCl_3$ were sublimed and transferred into the glovebox. The phosphanes were prepared according to literature procedures.[1–4]

Synthesis of [Bi(PCy_2)$_3$] (1)

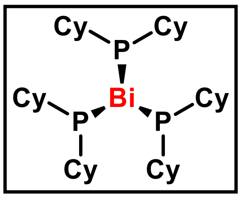

Di(cyclohexyl)phosphane (500 mg, 2.52 mmol, 3.0 equiv.) was dissolved in Et_2O (3.5 mL) and cooled to −78 °C. *n*-BuLi (1.01 mL, 2.53 mmol, 1.01 equiv., 2.5 M in *n*-hexane) was added dropwise, and the solution was stirred for 15 min at −78 °C, and afterwards stirred overnight at room temperature. $BiCl_3$ (265 mg, 0.84 mmol, 1.0 equiv.) was suspended in Et_2O (3 mL), and both mixtures were cooled to −100 °C (N_2(l)/*n*-pentane). The phosphanide solution was added slowly to the $BiCl_3$ suspension at −130 °C and the dark orange mixture was stirred for 1 h at −100 °C. The suspension was concentrated under reduced pressure, while keeping the solution at −100 °C over a period of 40 h (overnight storing in a freezer at −80 °C). A filtration with a precooled frit (at −73 °C) and washing the residue with cold (−100 °C) Et_2O (2 x 1.0 mL) led to a red solution. The filtrate was dried under reduced pressure while keeping the solution at −100 °C over a period of 16 h, yielding a red solid. Minor amounts of single-crystalline material suitable for XRD could be obtained by re-crystallization from *n*-pentane (0.5 mL) at −30 °C. However, **1** could not be purely isolated due to its facile decomposition in solution.

^{31}P{^{1}H}-NMR (C_6D_6, 298 K, ppm, 101 MHz): 147.9 (unspecified side products), 145.5 (possible intermediate of decomposition reactions), 13.8 (s, **1**, *P*–Cy), −20.9 (P_2Cy_4).

Synthesis of [Bi(P(*t*Bu)Cy)$_3$] (2)

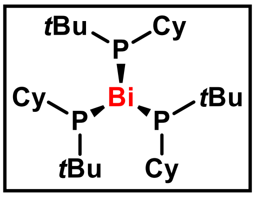

Cylcohexyl-(*tert*-butyl)phosphane (3.323 g, 19.29 mmol, 3.0 equiv.) was dissolved in THF (15 mL) and cooled to −78 °C. *n*-BuLi (7.80 mL, 19.50 mmol, 3.03 equiv., 2.5 M in *n*-hexane) was added dropwise, and the solution was stirred for 15 min at −78 °C and afterwards stirred overnight at room temperature. $BiCl_3$ (2.027 g, 6.43 mmol, 1.0 equiv.) was dissolved in THF (10 mL) and both solutions were cooled to −78 °C. The yellow phosphanide solution was added slowly to the $BiCl_3$ solution at −78 °C, and the resulting brown mixture was stirred for 15 min at −78 °C. All volatiles were removed under reduced pressure and *n*-pentane (20 mL) was added to the dark orange residue. The suspension was filtrated over celite and the residue was washed with *n*-pentane (3x10 mL). After removing all volatiles, [Bi(P(*t*Bu)Cy)$_3$] (**2**) was obtained as dark orange powder. Yield: 3.786 g, 5.23 mmol, 81%.

Two different isomers of **2** were detected *via* NMR spectroscopy. In the assignment of NMR spectroscopic resonances, signals for **2a** (with all *t*Bu groups in a *cis*-arrangement) and **2b** (with one *t*Bu group in *trans*-position relative to the two remaining *t*Bu groups) are

labeled as c for *cis* and tr for *trans*, if they can be distinguished. Due to the diastereomeric ratio of **2a** / **2b** = 1.00:1.08 at room temperature, the sum of all integrals in the spectrum (when normalized to 9H for a signal of one *t*Bu group of **2a**) amounts to 1.00*60 + 1.08*60 = 125 (final value rounded to 0 decimal places). In the assignment of resonances listed below, all integrals of resonances for **2b** have been divided by 1.08 so that an integer number of protons is assigned to each functional group, resulting in an overall number of 60H for **2a** and 60H for **2b** in the list of signal assignments.

^{1}H-NMR (C_6D_6, 298 K, ppm, 500 MHz): 1.13–1.40 (m, 12H, 3x C^4H_2 (6H, c)+ 3x C^4H_2 (6H, tr)), 1.40–1.49 (m, 47H, 3x C–CH_3 (27H, c), $^3J_{PH}$ = 10.5 Hz; C–CH_3 (9H, tr), $^3J_{PH}$ = 10.8 Hz; C–CH_3 (9H, tr), $^3J_{PH}$ = 10.7 Hz; 1x $C^{cyclo}H_2$ (2H, tr)), 1.49–1.54 (m, 9H, C–CH_3 (9H, tr), $^3J_{PH}$ = 11.2 Hz), 1.54–1.69 (m, 22H, 6x $C^{cyclo}H_2$ (12H, c); 5x $C^{cyclo}H_2$ (10H, tr)), 1.70–1.86 (m, 12H, 6x $C^{cyclo}H_2$ (12H, c)), 2.09–2.39 (m, 13H, 6x $C^{cyclo}H_2$ (12H, tr); P–C^1H (1H, tr)), 2.77–2.85 (m, 1H, P–C^1H (1H, tr)), 3.09–3.19 (m, 3H, P–C^1H (3H, c)), 3.30–3.41 (m, 1H, P–C^1H (1H, tr)).

^{1}H{^{31}P}-NMR (C_6D_6, 298 K, ppm, 500 MHz): 1.13–1.41 (m (m, 12H, 3x C^4H_2 (6H, c); 3x C^4H_2 (6H, tr)), 1.40–1.54 (m, 58H, 3x C–CH_3 (27H, c); 3x C–CH_3 (27H, tr) 4H, 1x $C^{cyclo}H_2$ (2H, tr)), 1.55–1.69 (m, 22H, 6x $C^{cyclo}H_2$ (12H, c); 5x $C^{cyclo}H_2$ (10H, tr)), 1.70–1.87 (m, 12H, 6x $C^{cyclo}H_2$ (12H, c)), 2.09–2.39 (m, 13H, 6x $C^{cyclo}H_2$ (12H, tr); P–C^1H (1H, tr)), 2.77–2.86 (m, 1H, P–C^1H (1H, tr)), 3.09–3.19 (m, 3H, P–C^1H (3H, c)), 3.30–3.41 (m, 1H, P–C^1H (1H, tr)).

^{13}C{^{1}H}-NMR (C_6D_6, 298 K, ppm, 125 MHz): 26.7 (s, C^4 (tr)), 26.8 (s, C^4 (tr)), 27.0 (s, C^4 (c)), cc27.1 (s, C^4 (tr)), 27.9 (d, $C^{3,5}$ (tr), $^3J_{PC}$ = 6.2 Hz), 28.0–28.2 (m, $C^{3,5}$ (c, tr)), 28.4–28.5 (m, $C^{3,5}$ (tr)), 28.7–28.9 (m, $C^{3,5}$ (tr)), 32.1–33.0 (m, *C*–CH$_3$ (c, tr)), 33.4–33.8 (m, C–*C*H$_3$ (c, tr)), 34.5 (d, *C*–CH$_3$ (tr), $^1J_{PC}$ = 38.8 Hz), 35.2–35.3 (m, $C^{2,6}$ (c)), 35.6–35.7 (m, $C^{2,6}$ (tr)), 35.8 (d, $C^{2,6}$ (tr), $^2J_{PC}$ = 12.4 Hz), 36.2 (m, $C^{2,6}$ (tr)), 36.3–36.7 (m, $C^{2,6}$ (c, tr)), 37.0–37.5 (m, $C^{2,6}$ (c, tr), P–C^1H (c, tr)), 38.4 (d, P–C^1H (tr), $^1J_{PC}$ = 38.6 Hz), 39.0 (s, P–C^1H (tr), $^1J_{PC}$ = 12.1 Hz).

^{13}C{^{1}H, ^{31}P}-NMR (C_6D_6, 298 K, ppm, 125 MHz): 26.8 (s, C^4 (tr)), 26.8 (s, C^4 (tr)), 27.0 (s, C^4 (c)), 27.1 (s, C^4 (tr)), 27.9 (s, $C^{3,5}$ (tr)), 28.1 (s, $C^{3,5}$ (tr)), 28.2 (s, $C^{3,5}$ (c)), 28.4 (s, $C^{3,5}$ (tr)), 28.5 (s, $C^{3,5}$ (tr)), 28.8 (s, $C^{3,5}$ (tr)), 28.9 (s, $C^{3,5}$ (tr)), 32.2 (s, *C*–CH$_3$ (c)), 32.5 (s, *C*–CH$_3$ (tr)), 32.8 (s, *C*–CH$_3$ (tr)), 33.5 (s, C–*C*H$_3$ (tr)), 33.6 (s, C–*C*H$_3$ (c)), 33.7 (s, C–*C*H$_3$ (tr)), 33.8 (s, C–*C*H$_3$ (tr)), 34.5 (s, *C*–CH$_3$ (tr)), 35.3 (s, $C^{2,6}$ (c)), 35.6 (s, $C^{2,6}$ (tr)), 35.6 (s, $C^{2,6}$ (tr)), 35.8 (s, $C^{2,6}$ (tr)), 36.3 (s, $C^{2,6}$ (tr)), 36.5 (s, $C^{2,6}$ (c)), 36.5 (s, $C^{2,6}$ (tr)), 37.1 (s, $C^{2,6}$ (tr)), 37.3 (s, P–C^1H (tr)), 37.3 (s, P–C^1H (c)), 38.4 (s, P–C^1H (tr)), 39.0 (s, P–C^1H (tr)).

^{31}P{^{1}H}-NMR (C_6D_6, 298 K, ppm, 101 MHz): 43.7 (s, 1P, *P* (tr)) 46.9 (s, 3P, *P* (c)), 47.8–48.8 (m, 2P, 1x *P*(tr)).

^{1}H-NMR (toluene-d_8, 298 K, ppm, 500 MHz):[a] 1.14-1.35 (m, 12H, 3x C^4H_2 (6H, c); 3x C^4H_2 (6H, tr)), 1.36–1.45 (m, 47H, 3x C–CH_3 (27H, c), $^3J_{PH}$ = 10.8 Hz; C–CH_3 (9H, tr), $^3J_{PH}$ = 10.8 Hz; C–CH_3 (9H, tr; no coupling constant can be determined due to overlap to another signal); 1x $C^{cyclo}H_2$ (2H, tr)), 1.45–1.50 (m, 13H, C–CH_3 (9H, tr), $^3J_{PH}$ = 11.2 Hz; 2x $C^{cyclo}H_2$ (4H, tr)), 1.50–1.62 (m, 16H, 6x $C^{cyclo}H_2$ (12H, c); 2x $C^{cyclo}H_2$ (4H, tr)),[a] 1.62–1.69 (m, 6H, 3x $C^{cyclo}H_2$ (6H, tr))), 1.73–1.83 (m, 12H, 6x $C^{cyclo}H_2$ (12H, c)), 2.09–2.30 (m, 14H, 4x $C^{cyclo}H_2$ (8H, tr), P–C^1H (1H, tr); overlap with CH_3 group of toluene-d_8), 2.77–2.84 (m, 1H, P–C^1H (1H, tr)), 3.08–3.20 (m, 3H, P–C^1H (3H, c)), 3.29–3.39 (m, 1H, P–C^1H (1H, tr)). a: In this spectrum, the unequivocal assignment of protons in cyclohexyl groups was not possible, as severe signal overlap complicated the interpretation. In sum, the integrals were somewhat larger than expected for the assigned number of protons, which is due to overlap with the signal of the methyl group of the solvent toluene-*d8*, due to minor amounts of *n*-pentane in the solvent and possibly due to differences in relaxation times.

^{31}P{^{1}H}-NMR (toluene-d_8, 298 K, ppm, 202.5 MHz): 42.4 (s, 1P, *P* (tr)) 45.5 (s, 3P, *P* (c)), 46.9–47.1 (m, 2P, 1x *P*(tr)).

ε_{max} (C_6H_6, nm): 300, 341, 439.

LIFDI-MS: positive mode, found 722.37024 m/z, calcd. for $C_{30}H_{60}BiP_3$ 722.37117 m/z.

Elemental analysis: calcd. (%) for $C_{30}H_{60}BiP_3$ (722.71 g·mol^{-1}): C 49.86 H 8.37; found: C 50.17 H 8.14.

Synthesis of [Bi(P*t*Bu$_2$)$_3$] (3)

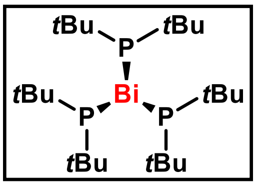

Di(*tert*-butyl)phosphane (4.250 g, 29.06 mmol, 3.00 equiv.) was dissolved in THF (20 mL) and cooled to −78 °C. *n*-BuLi (11.75 mL, 29.36 mmol, 3.01 equiv., 2.5 M in *n*-hexane) was added dropwise, and the solution was stirred for 15 min at −78 °C and afterwards stirred overnight at room temperature. $BiCl_3$ (3.055 g, 9.69 mmol, 1.00 equiv.) was dissolved in THF (15 mL) and both solutions were cooled to −78 °C. The yellow phosphanide solution was added slowly to the $BiCl_3$ solution at −78 °C, and the resulting red mixture was stirred for 15 min at −78 °C. All volatiles were removed under reduced pressure and *n*-pentane (25 mL) was added to the dark red residue. The suspension was filtrated over celite and the residue was washed with *n*-pentane (3x15 mL). After removing all volatiles [Bi(P*t*Bu$_2$)$_3$] (**3**) was obtained as dark red powder. Yield: 5.933 g, 9.21 mmol, 95%.

^{1}H-NMR (C_6D_6, 298 K, ppm, 500 MHz): 1.58 (d, 54H, $^3J_{PH}$ = 10.7 Hz, C–CH_3).

$^{1}H\{^{31}P\}$-NMR (C_6D_6, 298 K, ppm, 500 MHz): 1.58 (s, 54H, C–C*H*$_3$).
$^{13}C\{^{1}H\}$-NMR (C_6D_6, 298 K, ppm, 125 MHz): 34.3–34.5 (m,[a] C–*C*H$_3$), 36.8 (d, $^{1}J_{PC}$ = 45.8 Hz, *C*–CH$_3$). a: the appearance of a multiplet for the CH_3 groups ascribed to virtual coupling effects. A singlet is observed for the CH_3 group in the $^{13}C\{^{1}H, ^{31}P\}$-NMR spectrum (*vide infra*).
$^{13}C\{^{1}H, ^{31}P\}$-NMR (C_6D_6, 298 K, ppm, 125 MHz): 34.4 (m, C–*C*H$_3$), 36.8 (d, *C*–CH$_3$).
$^{31}P\{^{1}H\}$-NMR (C_6D_6, 298 K, ppm, 202.5 MHz): 86.4 (s, *P*–^{t}Bu).
ε_{max} (C_6H_6, nm): 311, 355, 487.
LIFDI-MS: positive mode, found 644.32615 m/z, calcd. for $C_{24}H_{54}BiP_3$ 644.32422 m/z.
Elemental analysis: calcd. (%) for $C_{24}H_{54}BiP_3$ (644.60 g·mol^{-1}): C 44.72 H 8.44; found: C 44.65 H 8.63.

Synthesis of [Bi(PAd$_2$)$_3$] (4)

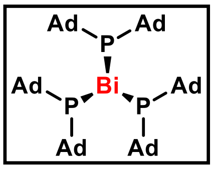

Di(adamantyl)phosphane (800 mg, 2.65 mmol, 3.01 equiv.) was dissolved in THF (10 mL) and cooled to −78 °C. *n*-BuLi (1.06 mL, 2.66 mmol, 3.02 equiv., 2.5 M in *n*-hexane) was added dropwise, and the solution was stirred for 10 min at −78 °C. The slightly yellow solution was stirred overnight, warming to room temperature. $BiCl_3$ (278 g, 0.88 mmol, 1.0 equiv.) was dissolved in THF (4 mL) and both solutions were cooled to −78 °C. The phosphanide solution was added slowly to the $BiCl_3$ solution at −78 °C, and the red mixture was stirred for 15 min at −78 °C. All volatiles were removed under reduced pressure, and THF (5 mL) was added to the dark red residue. The suspension was filtrated over glass microfiber filter and layered with *n*-hexane (2 mL). Storing the solution for 3 days at −30 °C led to a red precipitate. The residual solution was layered with n-hexane (2 mL) again and the process repeated until only a slightly colored solution remained. The analytically pure fractions of red precipitates were combined and dried in vacuo to give [Bi(PAd$_2$)$_3$] (**4**) as a dark red powder. Yield: 61.6 mg, 0.055 mmol, 6%.
^{1}H-NMR (C_6D_6, 298 K, ppm, 500 MHz): 1.69 (d, 18H, $^{3}J_{PH}$ = 11.7 Hz, α-C*H*$_2$), 1.93 (d, 18H, $^{3}J_{PH}$ = 10.9 Hz, α-C*H*$_2$), 2.05 (s, 18H, β-C*H*), 2.40–2.56 (m, 36H, γ-C*H*$_2$).
$^{1}H\{^{31}P\}$-NMR (C_6D_6, 298 K, ppm, 500 MHz): 1.69 (d, 18H, α-C*H*$_2$), 1.93 (d, 18H, α-C*H*$_2$), 2.05 (s, 18H, β-C*H*), 2.40–2.55 (m, 36H, γ-C*H*$_2$).
$^{13}C\{^{1}H\}$-NMR (C_6D_6, 298 K, ppm, 125 MHz): 30.3–30.5 (m, β-*C*H), 37.4 (s, α-*C*H$_2$) 41.6 (d, $^{1}J_{PC}$ = 48.6 Hz, P*C*$_q$), 45.7–46.1 (m, β-*C*H$_2$).
$^{31}P\{^{1}H\}$-NMR (C_6D_6, 298 K, ppm, 202 MHz): 77.9 (s, *P*–Ad).
Elemental analysis: calcd. (%) for $C_{60}H_{60}BiP_3$ (1113.28 g·mol^{-1}): C 64.72 H 8.15; found: C 64.73 H 7.915.

Aryl-substituted bismuth phosphanides

When developing a protocol to access simple, homoleptic bismuth phosphanides Bi(PRR')$_3$, reactions with one or two aryl substituents per phosphorus atom were also tested. While attempts to synthesize Bi(PPh$_2$)$_3$ and Bi(PMes$_2$)$_3$ led to the rapid precipitation of a dark solid, when the lithium phosphanide was combined with $BiCl_3$ at low termperature, the compound with a mixed substitution pattern at phosphorus, Bi(PtBuMes)$_3$ gave a deep-red colored solution that persisted at least for minutes at –78 °C. After work-up, all reactions yielded the corresponding diphosphane as the exclusive or main product according to NMR spectroscopic analyses.

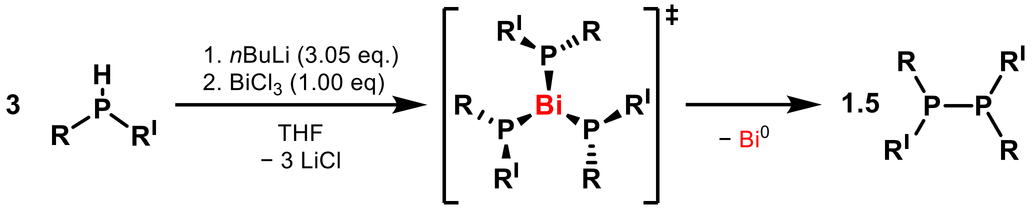

Scheme S1. Decomposition of proposed bismuth phosphanides to the corresponding diphosphanes and elemental bismuth.

Attempted synthesis of Bi(PPh$_2$)$_3$

Under inert conditions, diphenylphosphane (0.447 mg, 2.40 mmol, 1.00 equiv.) was dissolved in THF (10 mL). The solution was cooled to −78 °C and within 5 min *n*BuLi (1.0 mL, 2.50 mmol, 2.5M in *n*-hexane, 1.04 equiv.) was added dropwise. The resulting solution was stirred for 1 h and a color change from colorless to light yellow was observed. In a separate flask $BiCl_3$ (252 mg, 0.80 mmol, 0.33 equiv.) was dissolved in THF (12 mL) and cooled to −78 °C. The solution of the lithium phosphanide was added dropwise to the $BiCl_3$ solution within 5 min. A color change was observed from light yellow to dark red. Immediately after that the color changed again to the color of the corresponding tetraphenyldiphospahne (colorless) and a formation of a black participate, presumably Bi^0, was observed. The solvent was removed under reduced pressure and the residue was suspended in benzene (10 mL) and filtered through celite®. After rinsing the residue with small amounts of benzene, the solvent was removed from the filtrate under reduced pressure to afford the tetraphenyldiphosphane (430 mg, 1.16 mmol, 97%) as a colorless powder.

^{31}P{^{1}H}-NMR (C_6D_6, 298 K, ppm, 202 MHz): −13.6 (s, *P*Ph$_2$).

Lit:[5] **^{31}P{^{1}H}-NMR** (C_6D_6): −13.6 (s).

Attempted synthesis of $Bi(PMes_2)_3$

Under inert conditions, dimesitylphosphane (0.649 mg, 2.40 mmol, 1.00 equiv.) was dissolved in THF (10 mL). The solution was cooled to −78 °C and within 5 min *n*BuLi (1.0 mL, 2.50 mmol, 2.5M in *n*-hexane, 1.04 equiv.) was added dropwise. The resulting solution was stirred for 1 h and a color change from colorless to light yellow was observed. In a separate flask, $BiCl_3$ (252 mg, 0.80 mmol, 0.33 equiv.) was dissolved in THF (12 mL) and cooled to −78 °C. The solution of the lithium phosphanide was added dropwise to the $BiCl_3$ solution within 5 min. A color change was observed from yellow to dark red. Immediately after that the color changed again to the color of the corresponding tetramesityldiphospahne (light yellow) and the formation of a black participate, presumably Bi^0, was observed. The solvent was removed under reduced pressure and the residue was suspended in benzene (10 mL) and filtered through celite®. After rinsing the residue with small amounts of benzene, the solvent was removed under reduced pressure to afford the tetramesityldiphosphane (605 mg, 1.12 mmol, 94%) as a light-yellow powder.

$^{31}P\{^1H\}$-NMR (C_6D_6, 298 K, ppm, 202 MHz): −30.2 (s, *P*Mes_2).
Lit:[6] **$^{31}P\{^1H\}$-NMR** (CD_2Cl_2): −30.3 (s).

Attempted synthesis of Bi(P*t*BuMes)$_3$

Under inert conditions, *tert*-butylmesitylphosphane (0.150 mg, 0.72 mmol, 1.00 equiv.) was dissolved in THF (5 mL). The solution was cooled to −78 °C and within 5 min *n*BuLi (0.3 mL, 0.75 mmol, 2.5M in *n*-hexane, 1.04 equiv.) was added dropwise. The resulting solution was stirred for 1 h and a color change from colorless to light yellow was observed. In a separate flask $BiCl_3$ (75.7 mg, 0.24 mmol, 0.33 equiv.) was dissolved in THF (5 mL) and cooled to −78 °C. The solution of the lithium phosphanide was added dropwise to the $BiCl_3$ solution within 5 min. A color change was observed from yellow to dark orange. After roughly 5 min, while not cooling any more, the color changed to yellow and a formation of a black participate, presumably Bi^0, was observed. The solvent was removed under reduced pressure and the residue was suspended in benzene 10 mL and filtered through celite®. After rinsing, the solvent was removed under reduced pressure to afford the 205 mg of a yellow solid, which was identified *via* ^{31}P NMR as a mixture of compounds with di-*tert*-butyl-dimesityldiphosphane as the main component.

$^{31}P\{^1H\}$-NMR (C_6D_6, 298 K, ppm, 202 MHz): −50.4 (s, unknown sideproduct), −17.2 (s, (*Pt*BuMes)$_2$ (*rac* or *meso*)) , −14.7 (s, (*Pt*BuMes)$_2$ (*meso* or *rac*)), 45.3 (s, unknown side product), 111.2 (s, MestBu*P*Cl).

The following integrals were obtained for the signals listed above: −50.4 ppm: 0.51; −17.2 ppm: 1.00; −14.7 ppm: 0.99; 45.3 ppm: 0.24; 111.2 ppm: 0.21, making the diphosphane (resonances at −17.2 and −14.7 ppm) the main product according to ^{31}P NMR spectroscopic analyses. Analytical data indicates the formation of the *rac* and the *meso* isomer of *t*BuMesP–PMes*t*Bu in a 1:1 ratio, which is agreement with a radical mechanism and with the literature on related compounds.[7–9]

Temperature dependent $^{31}P\{^{1}H\}$ NMR measurements of 2

To better understand the behavior of compound **2** in solution, we carried out temperature-dependent $^{31}P\{^{1}H\}$ NMR spectroscopic investigations. The measurements were carried out in the range of 193 K to 353 K in steps of 10 K at a frequency of 202 MHz in toluene-d_8.

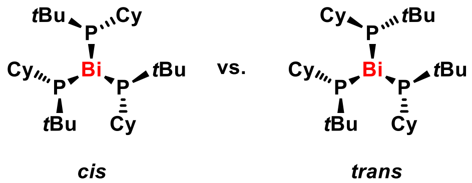

Figure S1. Illustration of *cis*- (**2a**) and *trans*-isomers (**2b**) of **2**.

While cooling **2** to lower temperatures, the relative order of the signals (according to their chemical shifts) and the splitting of the signals changed. At 213 K all three signals of the *trans*-species (**2b**) are upfield-shifted compared to the signal of the *cis*-species. In addition to this, the integral of the signal for the *cis*-species is larger than the sum of integrals of the signals due to the *trans*-species. At 273 K, two signals of the *trans*-species show higher chemical shifts than that of the *cis*-species. At higher temperatures (343 K), two signals of the *trans*-species overlap. Comparing the integrals of the *cis*- and *trans*-isomers shows that the *trans*-species is favored at higher temperatures.

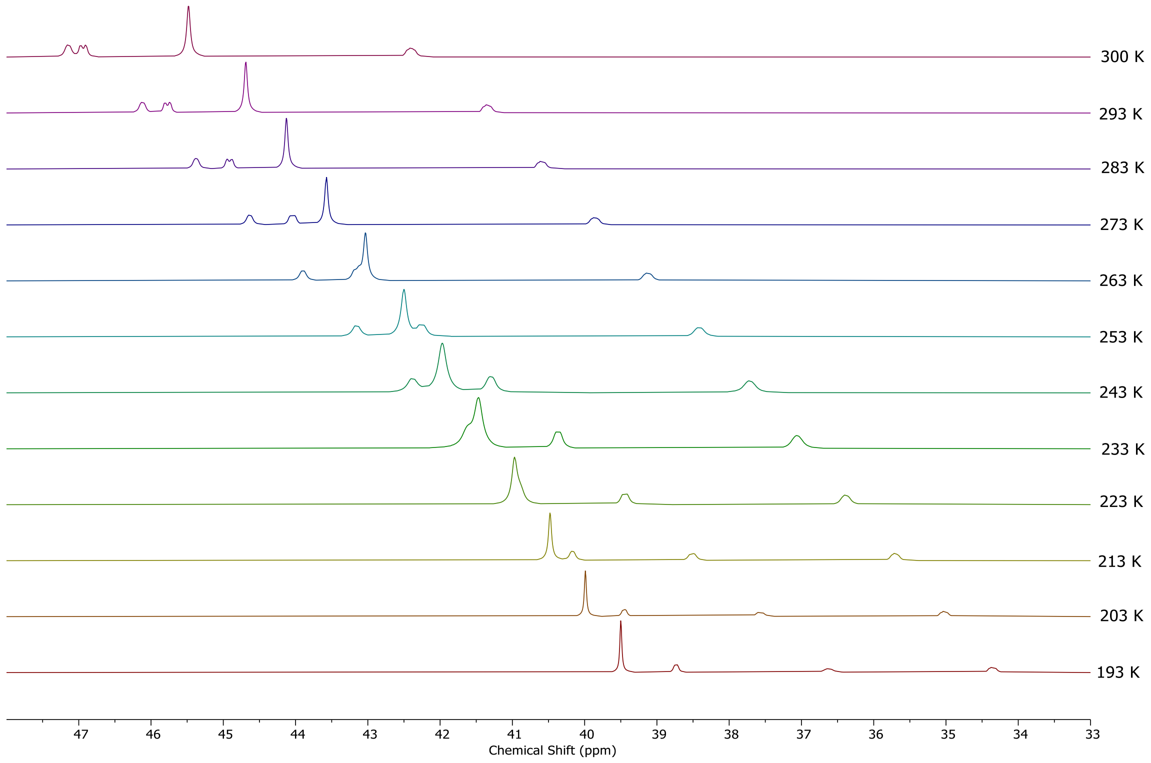

Figure S2. Temperature dependent $^{31}P\{^{1}H\}$-NMR spectra of [Bi(P(*t*Bu)Cy)$_3$] (**2**) in the range of 33–48 ppm in toluene-d_8 (202 MHz) starting at 193 K up to 300 K (rt).

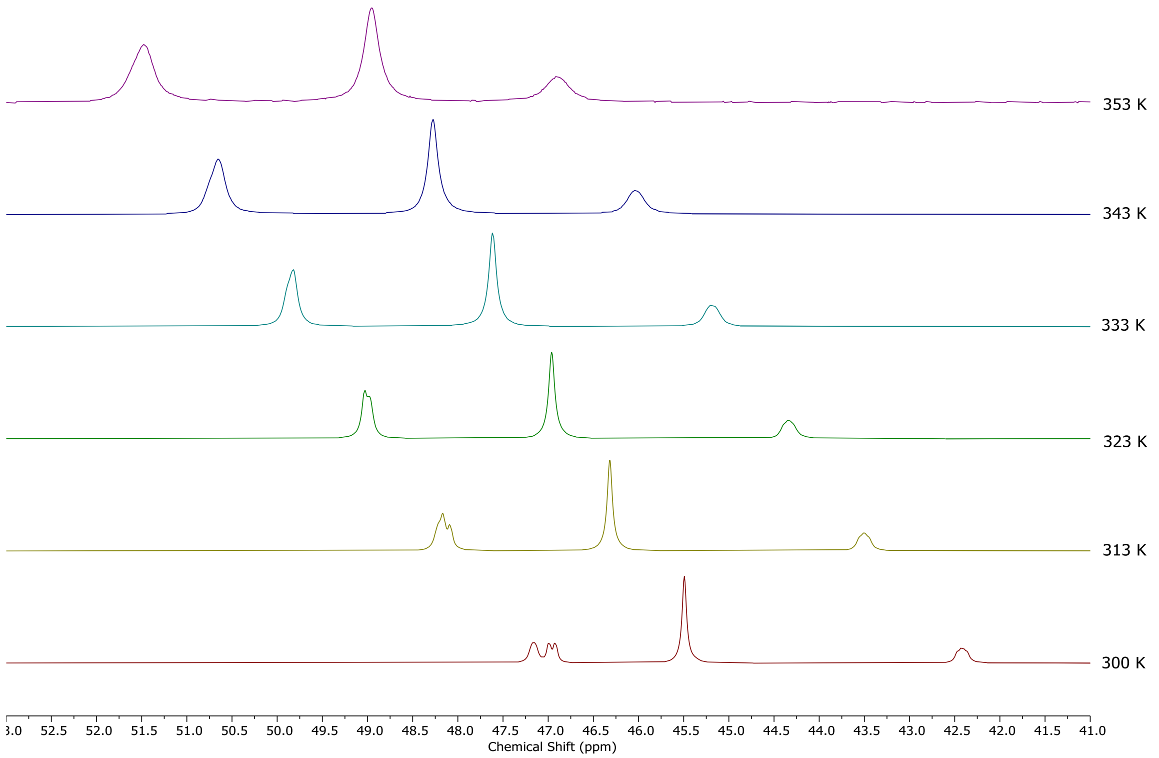

Figure S3. Temperature dependent $^{31}P\{^1H\}$-NMR spectra of [Bi(P(*t*Bu)Cy)$_3$] (**2**) in the range of 41–53 ppm in toluene-d$_8$ (202 MHz) starting at 300 K (rt) up to 353 K.

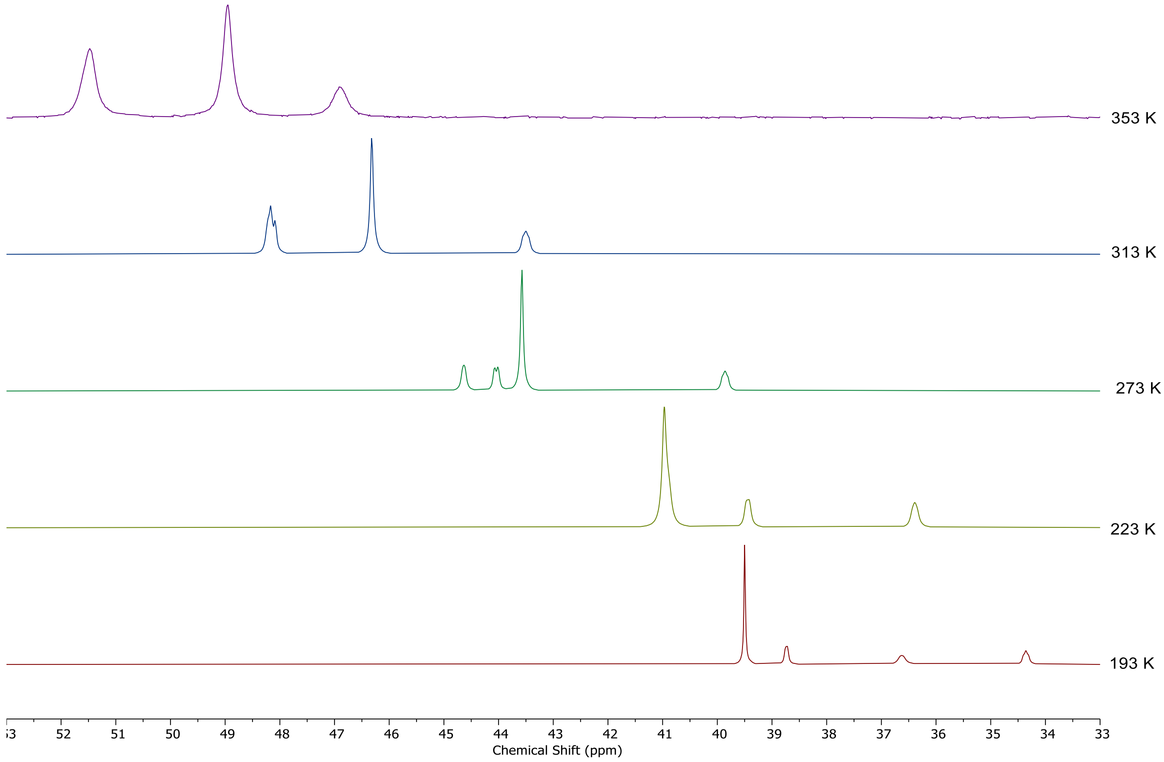

Figure S4. Temperature dependent $^{31}P\{^1H\}$-NMR spectra of [Bi(P(*t*Bu)Cy)$_3$] (**2**) in the range of 33–53 ppm in toluene-d$_8$ (202 MHz) starting at 193 K (rt) up to 353 K.

Table S1. Sum of integrals and ratio of the two diastereomers of **2** at selected temperatures.

Temperature [K]	**2a**	**2b**	$K\ (\frac{\mathbf{2b}}{\mathbf{2a}})$
273	3.23	3.17	0.981
283	3.12	3.13	1.003
293	3.01	3.08	1.023
300	2.92	3.07	1.051
313	2.79	3.04	1.090
323	2.71	3.06	1.129
333	2.69	3.08	1.145
343	2.58	3.01	1.167
353	2.55	3.08	1.208

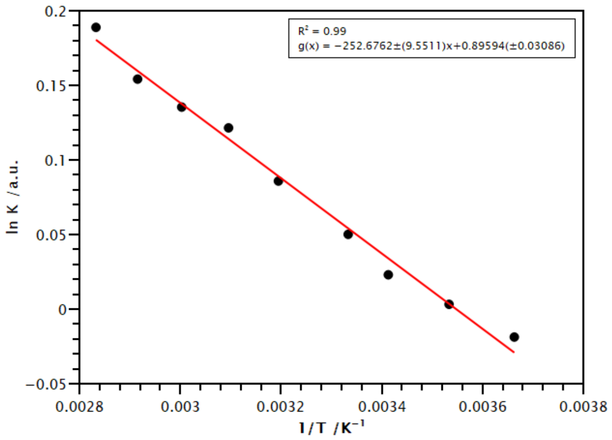

Figure S5. Van't Hoff plot for the interconversion of the diastereomers **2a** and **2b** for the temperature range of 273–353 K.

From the van't Hoff plot of the interconversion of the two diastereomers **2a** and **2b**, the standard reaction enthalpy $\Delta_r H$ and standard reaction entropy $\Delta_r S$ for the reaction **2a** → **2b** can be calculated as follows:

$$\Delta_r H = -R \cdot -252.6762(\pm 9.5511)$$

$$\Delta_r H = -8.314\, J \cdot mol^{-1} \cdot K^{-1} \cdot -252.6762(\pm 9.5511) \cdot K$$

$$\underline{\Delta_r H = 2100.7499(\pm 79.4078)\, J \cdot mol^{-1}}$$

$$\Delta_r S = R \cdot 0.89594(\pm 0.03086)$$

$$\Delta_r S = 8.314\, J \cdot mol^{-1} \cdot K^{-1} \cdot 0.89594(\pm 0.03086)$$

$$\underline{\Delta_r S = 7.45197(\pm 0.25657)\, J \cdot mol^{-1} \cdot K^{-1}}$$

This translates into $\Delta_r H$ = +0.50 kcal·mol^{-1} and $\Delta_r S$ = +1.8 cal·mol^{-1}. From these values, $\Delta_r G$*(293 K)* = −0.027 kcal·mol^{-1} can be calculated. These values show that there is a small temperature-dependency of the free reaction enthalpy for the interconversion of **2a** and **2b**.

Eyring Plot

The VT NMR spectroscopic data of compound **2** were further investigated by line-shape analysis. In principle, this offers the possibility to deduce rate constants for the interconversion of **2a** and **2b** at varying temperatures, which would create a basis for setting up an Eyring plot, thereby deducing activation parameters of the isomerization.

It has to be stressed, however, that there is a number of points which hamper this analysis and therefore make it less accurate in this particular case. The line shape analysis as implemented in the topspin software is meant for intramolecular processes. This can be assumed to be correct for the rotation-inversion mechanism, but is not necessarily the case for the radical dissociation / re-association mechanism. Furthermore, there is the formation of the diphosphane side-product taking place at higher temperatures, which may affect the (apparent) kinetic parameters. Importantly, the coupling in the ^{31}P NMR spectrum is not well-resolved for compound **2b**. This leads to uncertainties in the fitting of the line-broadening, which creates a larger possible error in the rate constant obtained in the fitting process and the parameters derived thereof (such as the activation enthalpy and activation entropy obtained from the Eyring plot).

Based on the VT ^{31}P NMR spectroscopic data of compound **2** in toluene-*d*8, the rates of isomerization at varying temperatures were determined.[10] Line-shape analysis allowed the rate constant *k* to be estimated at seven different temperatures in the range of 193–353 K. The activation parameters ($\Delta H^{\ddagger}$ and $\Delta S^{\ddagger}$) for the isomerization between **2a** and **2b** were obtained from an Eyring plot.[11]

Table S2. Determined parameters used for the Eyring plot for **2** at selected temperatures.

Temperature [K]	overlap [%]	k [s^{-1}]	1/T [K^{-1}]	$ln(k/T)$
353	95.963	4.50E+01	2.83E−03	−2.060
333	97.197	2.52E+01	3.00E−03	−2.583
300	96.019	8.08E+00	3.33E−03	−3.614
283	96.692	4.97E+00	3.53E−03	−4.042
253	97.761	2.00E+00	3.95E−03	−4.839
223	97.853	6.15E−01	4.48E−03	−5.893
193	89.630	5.10E−02	5.18E−03	−8.240

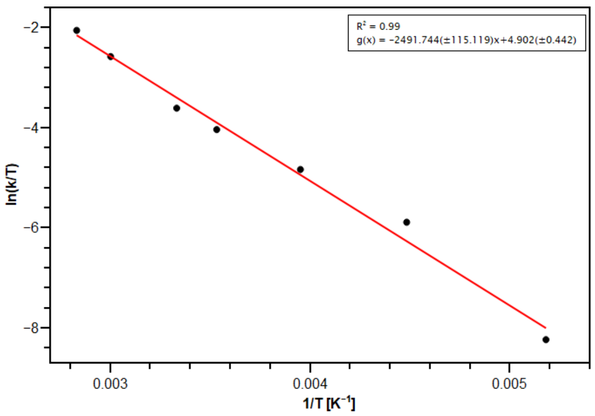

Figure S6. Eyring plot for the interconversion of the diastereomers **2a** and **2b** for the temperature range of 193–353 K.

From the Eyring plot of the interconversion of the two diastereomers **2a** and **2b**, the activation enthalpy $\Delta H^{\ddagger}$ and activation entropy $\Delta S^{\ddagger}$ for the reaction **2a** → **2b** can be calculated as follows:

$$\Delta H^{\ddagger} = -R \cdot -2491.744(\pm 114.119) \cdot K$$
$$\Delta H^{\ddagger} = -8.314\, J \cdot mol^{-1} \cdot K^{-1} \cdot -2491.744(\pm 114.119) \cdot K$$
$$\underline{\Delta H^{\ddagger} = 20.716(\pm 0.949)\ kJ \cdot mol^{-1}}$$

$$\Delta S^{\ddagger} = \left(4.902(\pm 0.442) - \ln\left(\frac{k_b}{h}\right)\right) \cdot R$$
$$\Delta S^{\ddagger} = \left(4.902(\pm 0.442) - \ln\left(\frac{1.38 \cdot 10^{-23}}{6.63 \cdot 10^{-34}}\right)\right) \cdot 8.314\, J \cdot mol^{-1} \cdot K^{-1}$$
$$\Delta S^{\ddagger} = -0.157(\pm 0.0389)\ kJ \cdot mol^{-1} \cdot K^{-1}$$

This translates into $\Delta H^{\ddagger}$ = +4.951 kcal·mol^{-1} and $\Delta S^{\ddagger}$ = −37.471 cal·mol^{-1}·K^{-1}. From these values, $\Delta G^{\ddagger}$ (300 K) = 16.192 kcal·mol^{-1} can be calculated. These values show that there is a small discrepancy between the calculated and the experimentally determined Gibbs activation energy for the interconversion of **2a** and **2b**. A minor contribution to these differences can be traced back to the different solvent used for the experiment (toluene-*d*8) and the calculations (C_6H_6). In addition to that, three major points have been mentioned above, which cause potential errors in the experimental investigation (intra- vs intermolecular processes; occurrence of side reactions; non-resolved coupling in the signals of **2b**). Especially the last point (non-resolved coupling) proved to be problematic in the line-shape analysis, as it led to uncertainties in the

fitting of the line-broadening, which creates a larger possible error in the rate constant obtained in the fitting process and the parameters derived thereof (specifically, the activation enthalpy and activation entropy obtained from the Eyring plot).

As such, it can be concluded that in view of the limitations of the line-shape analysis in this particular case, this analysis is in overall agreement with the theoretical analysis of the isomerization of **2a** and **2b**.

In addition to the temperature-dependent ^{31}P NMR spectroscopic measurements, ^{31}P-^{31}P NMR exchange spectroscopy experiments (EXSY) have been carried out. For slow exchange where the line widths of NMR signals are unaffected by the dynamic processes, the observation of signal coalescence is very restricted or even impossible. A total line shape analysis often proved to be inapplicable. The study of exchange dynamics for such cases has been realized by the observation of two-dimensional (2D) exchange spectroscopy (EXSY).[12] The EXSY experiment has proved to be an especially powerful method and thus been successfully applied to characterize many chemical and biological exchange processes even in the presence of complicated exchange networks.[13,14] The 2D experiment, originally developed for ^{1}H observation, was soon extended to ^{13}C NMR spectroscopy,[15,16] then followed by other heteronucleus (X-nucleus) spectroscopies, e.g. X = ^{6}Li, ^{19}F, and ^{31}P.[17–25]

Experimental. The ^{31}P-^{31}P EXSY spectrum was recorded on a Bruker AVANCE III 500 MHz spectrometer equipped with a 5mm BBO (broadband observation) Cryo Probe Prodigy. The pulse sequence EXSYX for recording the Exchange Spectroscopy on X-nuclei was used. Decoupling of ^{1}H during the evolution of the ^{31}P chemical shift was fulfilled by setting a 180° refocusing pulse on the ^{1}H channel in the middle of t_1. The phase cycle and the pulsed field gradients are in accord with those from the standard EXSY in Bruker pulse program library. For the two-dimensional ^{31}P-^{31}P EXSY spectrum, the spectral width was 40 to 80 ppm, with 16 to 32 scans per increment and 128 to 256 increments. The mixing time used was 0.5 s and 1.5 s for compound **2** and 1.5 s and 5.0 s for the equilibrium measurement of a sample containing **2** and the diphosphane **5** (molar ratio **2**:**5** = 10:1). A relaxation delay of 2.5 s was applied.

Results. Two species of complex **2** were observed in solution, with near-equal population at room temperature (the isomers **2a** and **2b**). Thus, in the ^{31}P spectrum a singlet at 45.9 ppm with an intensity of 3 was assigned the resonance of **2a** and the other three signals at 47.3 ppm, 46.9 ppm and 42.6 ppm each showed a relative intensity of 1 (and are assigned to the isomer **2b**). As revealed in Figure S7, exchange was observed between 46.9–42.6 ppm (within **2b**), 45.9–47.3 ppm, 45.9–46.9 ppm, and 45.9–42.6 ppm (**2a** with all signals of **2b**).

Furthermore, ^{31}P-^{31}P EXSY spectra were recorded on a sample of complex **2** in the presence of diphosphane **5**. No exchange could be detected between all the species of **2** and **5**, even with mixing time as long as 5 s. Therefore, we concluded that any possible exchange going on in the solution to be slower than 0.06 s^{-1} and is thus highly unlikely to be relevant for the isomerization process of **2a** and **2b**.

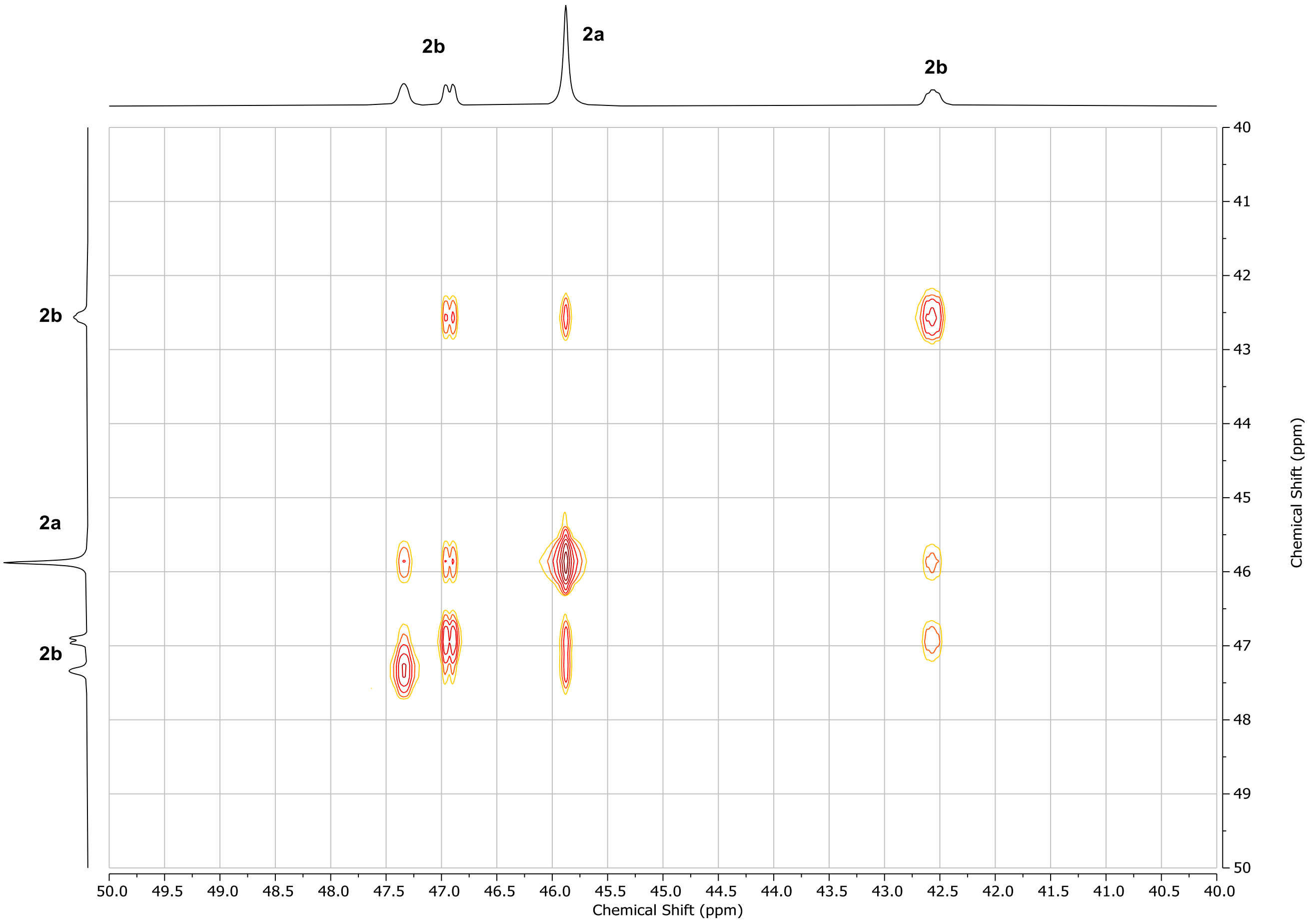

Figure S7. ^{31}P-^{31}P EXSY (EXSYX) spectrum (202.5 MHz) of sample **2** in C_6D_6 at room temperature.

P–P coupling of the bismuth phosphanides 2 and 3

In solution the compounds **1**–**4** decompose to the corresponding diphosphanes and a black solid, presumably Bi^0. This hampered the isolation of **1** in synthetically relevant amounts and diminished the isolated yield of **4** so far.

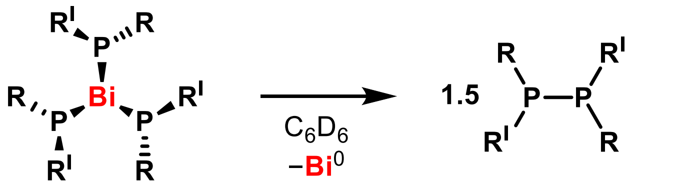

2: R = Cy, R^I = *t*Bu
3: R = R^I = *t*Bu

5: R = Cy, R^I = *t*Bu
6: R = R^I = *t*Bu

Scheme S2. Decomposition of bismuth phosphanides to the corresponding diphosphanes and elemental bismuth.

The decomposition rates of selected bismuth phosphanides were investigated. Specifically, compounds **2** and **3** were analyzed. A concentrated solution of **2** (28.2 mg, 0.0390 mmol) or **3** (25.2 mg, 0.0391 mmol) in C_6D_6 (0.5 mL) was prepared. The solution was regularly monitored by $^{31}P\{^1H\}$ NMR spectroscopy to validate the amount of diphosphane.

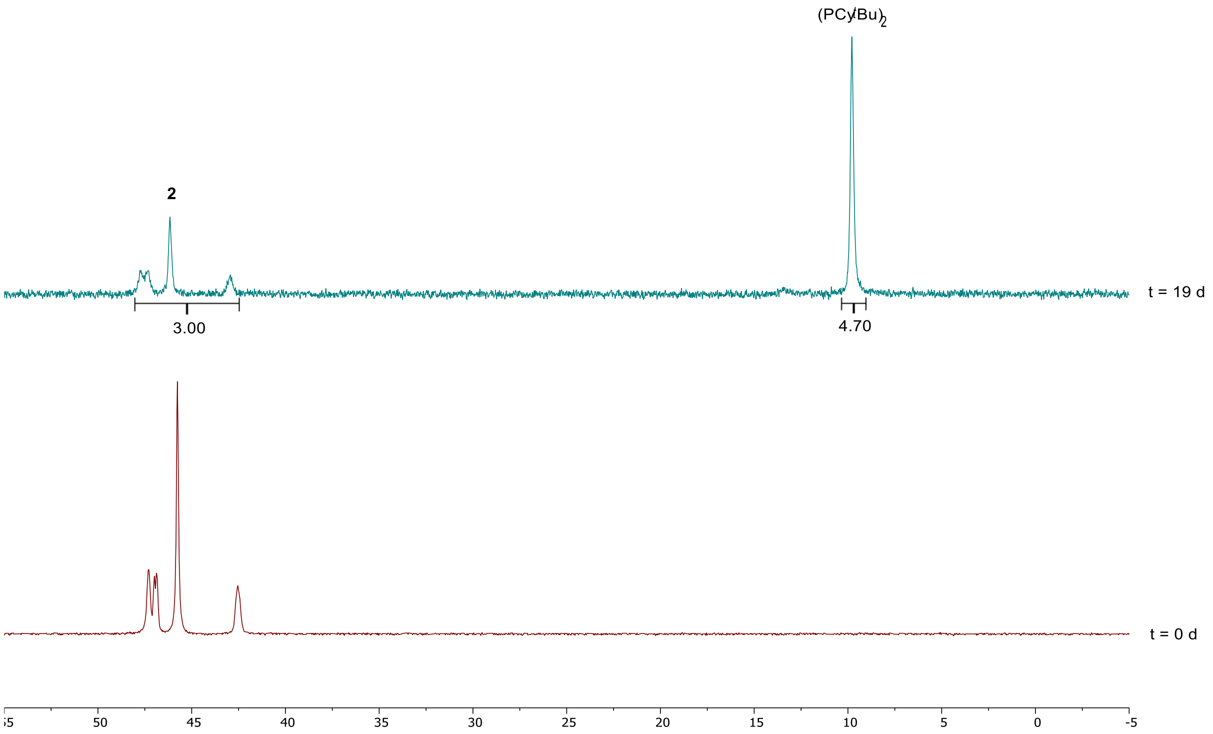

Figure S8. $^{31}P\{^1H\}$-NMR spectra of $[Bi(P(tBu)Cy)_3]$ (**2**) in the range of −5–55 ppm in C_6D_6 (202 MHz) at 300 K. Red: start. Blue: after 19 d.

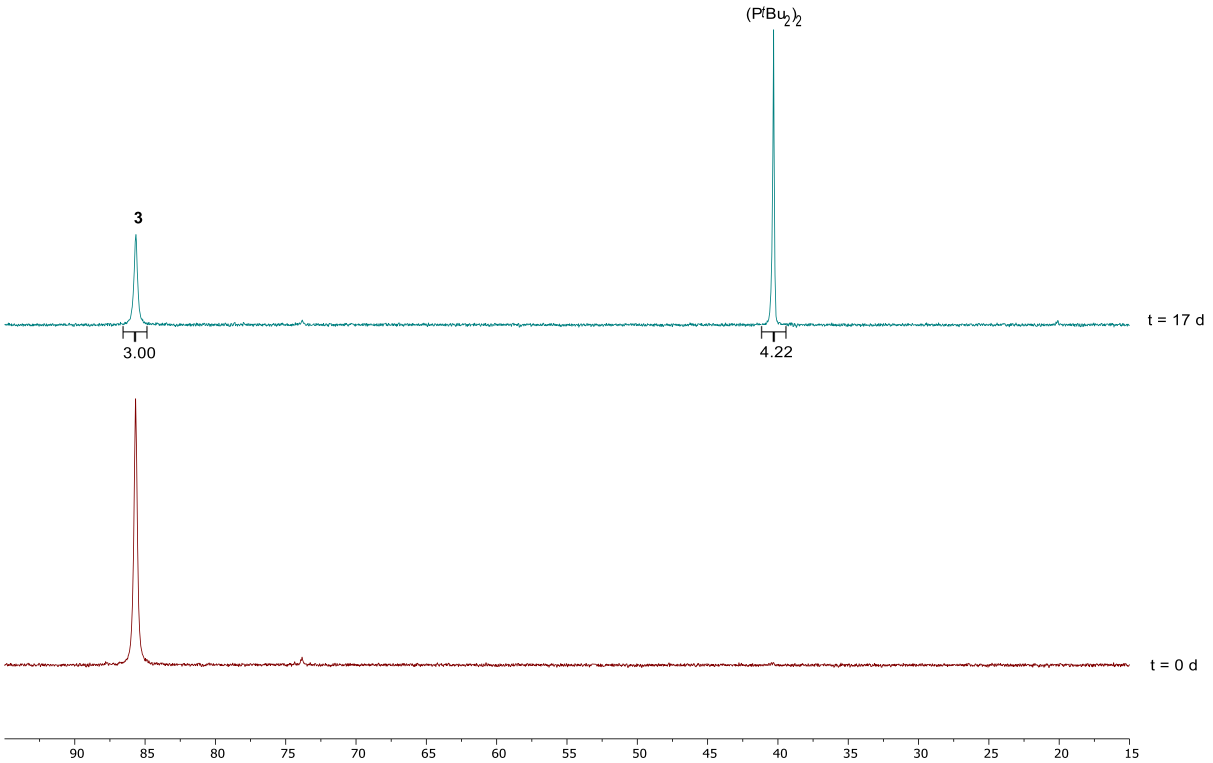

Figure S9. ^{31}P{^{1}H}-NMR spectra of [Bi(P*t*Bu$_2$)$_3$] (**3**) in the range of 15–95 ppm in C_6D_6 (202 MHz) at 300 K. Red: start. Blue: after 17 d.

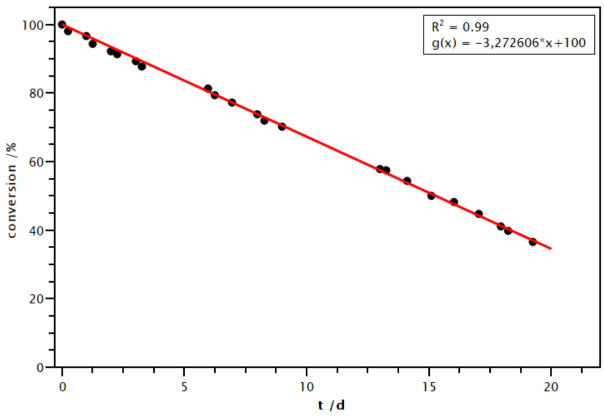

Figure S10. Decomposition of **2** (c = 78.0 mmol/L) in C_6D_6 monitored *via* ^{31}P{^{1}H} NMR spectroscopy.

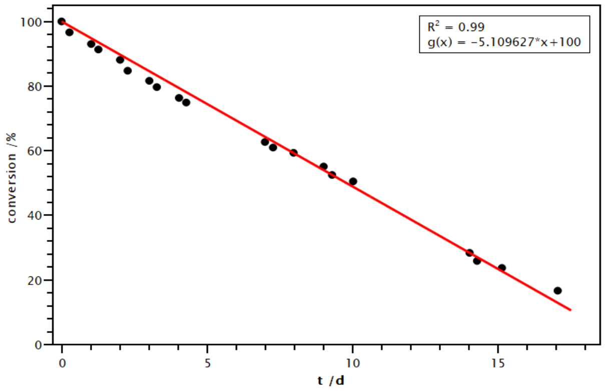

Figure S11. Decomposition of **3** (c = 78.2 mmol/L) in C_6D_6 monitored *via* $^{31}P\{^1H\}$ NMR spectroscopy.

Fitting the conversions of **2** and **3** against the time leads to graphs S30 and S31. The half-life times $t_{1/2}$ of **2** and **3** can be determined as follows:

$$g(x) = b \cdot x + 100$$

$$50 = b \cdot x + 100$$

$$x = -50/b$$

$t_{1/2}(\mathbf{2}) = -50/-3.272606\ d^{-1}$ $t_{1/2}(\mathbf{3}) = -50/ -5.109627\ d^{-1}$

$\underline{t_{1/2}(\mathbf{2}) = 15.27\ d}$ $\underline{t_{1/2}(\mathbf{3}) = 9.78\ d}$

Exchange reactions of **2** (28.0 mg, 0.0387 mmol, 1.00 equiv.) and **3** (25.0 mg, 0.0389 mmol, 1.00 equiv.) in a 1:1 ratio in 2.0 mL toluene showed a new set of signals in the range of the chemical shifts observed for the starting materials **2** and **3**, indicating the formation of mixed heteroleptic bismuthphosphanides. Due to an overlap of these signals a specific ratio was not determined. Besides the typical P–P coupling products **5** (^{31}P NMR shift: 9.8 ppm) and **6** (40.5 ppm) a signals at 22.0 and 30.3 ppm were detected, suggesting the formation of the mixed diphosphane *t*Bu$_2$P–PCy*t*Bu (ratio **5** / **6** / *t*Bu$_2$P–PCy*t*Bu = 0.19:0.65:0.16 after a reaction time of 16 h at ambient temperature).

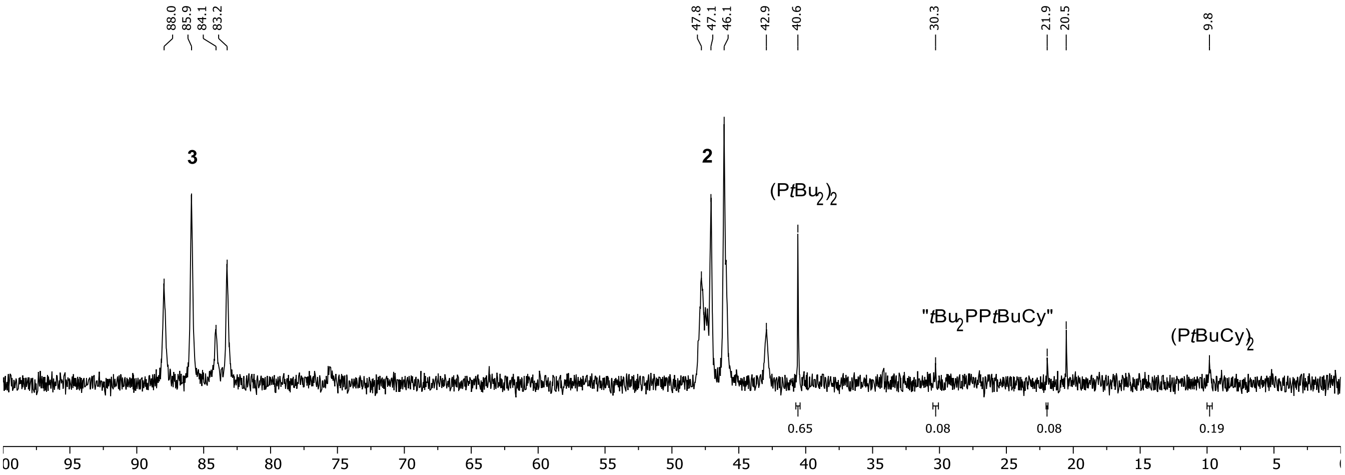

Figure S12. $^{31}P\{^{1}H\}$-NMR spectrum of the reaction of 2 and **3** after 16 h in toluene (298 K, 202 MHz).

Reactivity as a phosphanyl radical precursor

Compound **3** (150 mg, 0.23 mmol, 1.00 equiv.) was dissolved in 2 mL *n*-pentane and CS_2 (0.03 mL, 51.2 mmol, 2.2 equiv.) were added dropwise. An immediate color change from dark red to violet and the formation of a black precipitate, elemental bismuth, was observed. After stirring the suspension for 1 hour another color change to smaragd green was observed. After filtration the solvent was removed from the filtrate under reduced pressure to afford compound **7** (118 mg, 32.1 mmol, 92%) as a dark green powder.

Attempts of ^{31}P NMR spectroscopic reaction monitoring did not allow for the detection of any intermediates of the reaction.

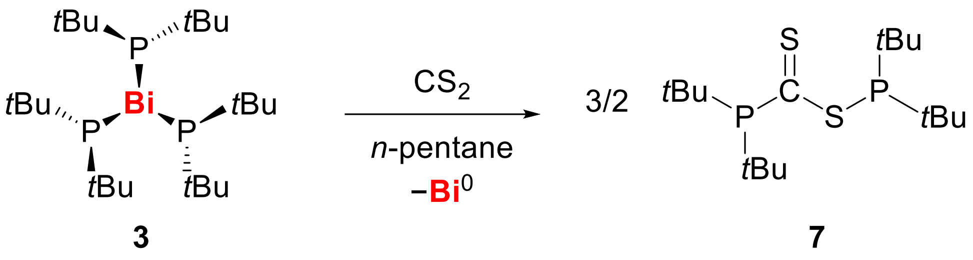

Scheme S3. Reaction of CS_2 with **3** leading to compounds **7**.

Compound 7

^{1}H-NMR (C_6D_6, 298 K, ppm, 500 MHz): 1.39 (d, 18H, $^3J_{PH}$ = 11.7 Hz, CPC(C*H*$_3$)$_3$), 1.45 (d, 18H, $^3J_{PH}$ = 10.9 Hz, SPC(C*H*$_3$)$_3$).

^{13}C{^{1}H}-NMR (C_6D_6, 298 K, ppm, 125 MHz): 29.8 (d, $^2J_{PC}$ = 14.1 Hz, CPC(*C*H$_3$)$_3$), 30.5 (d, $^2J_{PC}$ = 14.0 Hz, SPC(*C*H$_3$)$_3$), 38.8 (m, overlapping, 2 × P*C*(CH$_3$)$_3$), 246.7 (dd, $^1J_{PC}$ = 64.2 Hz, $^2J_{PC}$ = 20.2 Hz, *C*=S).

^{31}P{^{1}H}-NMR (C_6D_6, 298 K, ppm, 202 MHz): 97.1 (s, P–S), 76.9 (s, *P*–*C*).

Elemental analysis: calcd. (%) for $C_{17}H_{36}P_2S_2$ (366.54 g·mol^{-1}): C 55.71 H 9.90 S 17.49; found: C 55.90 H 10.002 S 17.50.

Reactivity towards alkenes

Initial reactivity studies showed that in the presence of olefins, compound **2** predominantly underwent phosphanyl radical homocoupling to give the diphosphane **5**, with the olefin remaining unreacted. In contrast, intriguing olefin insertion reactions were observed with the more reactive bismuth phosphanide **3**.

Reactions towards ethylene

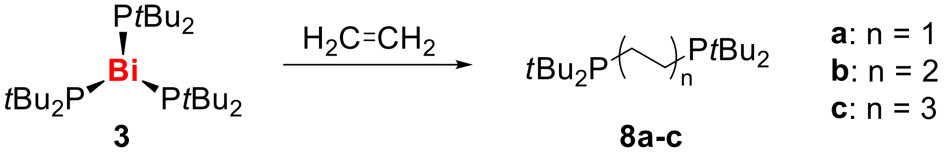

Scheme S4. Reaction of ethylene with **3** leading to compounds **8a–c**.

A solution of compound **3** (25 mg, 0.0389 mmol, 1.00 equiv.) in C_6D_6 (3 mL) was added in a high-pressure vessel under inert conditions. The vessel was pressurized with ethylene (20 bar) and the solution was stirred under these conditions for 24 h. The resulting suspension was filtrated, and the colorless solution was investigated. Via ^{31}P NMR spectroscopy, the main species was identified as the diphosphane **6** (42%). Besides that, the insertion / reductive elimination products *t*$Bu_2P(C_2H_4)P$*t*Bu_2 (**8a**, 15%), *t*$Bu_2P(C_2H_4)_2P$*t*Bu_2 (**8b**, 9%), *t*$Bu_2P(C_2H_4)_3P$*t*Bu_2 (**8c**, 2%), starting material **3** (8%) and HP*t*Bu_2 (1%) were detected and the ^{31}P NMR shifts are in agreement for literature known compounds (**8a** and **8b**).[26,27] These assignments were supported by HPLC-MS analyses, which were in agreement with the relative molar ratios determined by NMR spectroscopy and showed signals for the corresponding species of **6**, **8a–c** and HP*t*Bu_2 (the latter being obtained from hydrolysis of unreacted **3**) (all species detected as $M+H^+$ in mass spectra). Signals in the range of 58–74 ppm indicate an intermediate resulting from an insertion reaction, with at least one Bi–P bond still being intact. Attempts at isolating these intermediates were unsuccessful to date.

Changing the pressure (1 bar or 5 bar), time (4 h or 48 h) or temperature (40 °C or 60 °C) did not lead to increased yields of desired products.

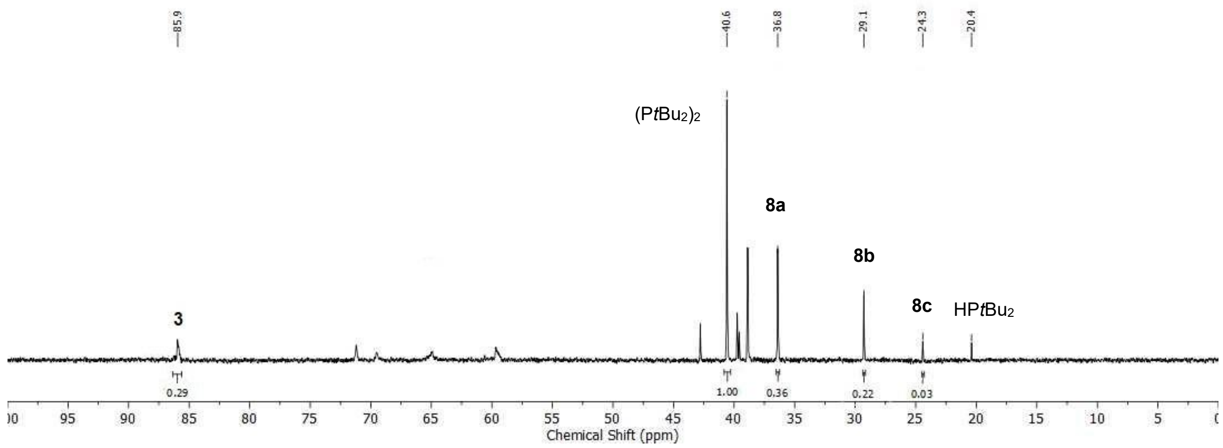

Figure S13. $^{31}P\{^{1}H\}$-NMR spectrum of the reaction of **3** with ethylene after 24 h in C_6D_6 (298 K, 202 MHz).

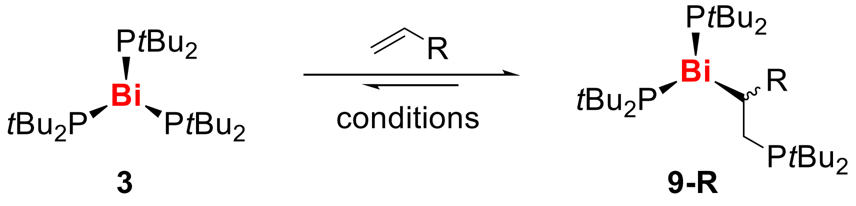

Scheme S5. Insertion of α-olefins $H_2C{=}CHR$ (R = Et, *n*Pr, *n*Bu) into one Bi–P bond of **3** leading to the insertion products **9-R**.

Reaction towards liquid substrates

Compound **3** (300 mg, 0.47 mmol, 1.00 equiv.) was dissolved in the alkene (5 mL of 1-pentene (45.7 mmol, 98.0 equiv.) or 1-hexene (40.0 mmol, 85 equiv.)) and stirred for up to 10 days. The reaction was monitored with ^{31}P NMR spectroscopy and was finished when no starting material **3** was detected. A color change from red to orange and the formation of a dark precipitate (presumably elemental bismuth) was observed. Afterwards the suspension was filtered, and the filtrate was carefully concentrated under reduced pressure until small traces of a solid started to precipitate. The solution was stored at −30 °C overnight, leading to the precipitation of solid material. The supernatant was removed and the solid was re-dissolved in fresh alkene (roughly 0.025 mL per mg of raw product). This re-crystallization procedure was repeated until the solid was pure by ^{31}P NMR spectroscopy (up to 21 times). Yield (**9-*n*Pr**): 15 mg, 0.02 mmol, 5%. Yield (**9-*n*Bu**): 45 mg, 0.06 mmol, 13%. Compared to that, the spectroscopic yields are higher (**9-*n*Pr**: 27%; **9-*n*Bu**: 34%), but due to the decomposition of the compounds in solution and the number of re-crystallization steps the isolated yield is reduced.

Re-dissolving the isolated products **9-*n*Pr** or **9-*n*Bu** in C_6D_6 led to a mixture of the starting material **3** and decomposition products (i.e. Bi^0 and the corresponding diphosphane) (ratio **9-*n*Bu**:**3**:**6** = 18:57:25) after approx. 5 min. Qualitatively identical results were obtained when performing these analyses with exclusion of light. Single crystals suitable for XRD analyses could not be obtained to date.

Reactions with only 1 equivalent of the alkenes led only to small amounts of the insertion products **9-*n*Pr** or **9-*n*Bu** and showed the diphosphane **6** and the starting material **3** as the main species (**9-*n*Bu**:**3**:**6** = 3:45:52) after 10 days. Increasing the amount of olefin to up to 5 equivalents increased the amount of the insertion products **9-R** but showed similar amounts of the diphosphane **6** and the starting material **3** (**9-*n*Bu**:**3**:**6** = 8:43:49). This led to the assumption that the insertion of the alkenes into one of the Bi–P is an equilibrium reaction, and a larger amount of the alkene is needed to obtain a larger conversion of the insertion products **9-R**.

Exclusion of light. Reactions were also performed under the exclusion of ambient light. In a brown glass NMR tube, **3** (25 mg, 0.0389 mmol, 1.00 equiv.) was dissolved in 1-hexene (0.5 mL, 4.00 mmol, 103 equiv.). The reaction was monitored by ^{31}P NMR spectroscopy, showing only the formation of the diphosphane **6**. The reaction was aborted after 12 d.

Irradiation experiments. In addition to that, the same reaction was also performed under photochemical conditions, i.e. the sample was irradiated with different light sources. Specifically, compound **3** (25 mg, 0.0389 mmol, 1.00 equiv.) was dissolved in 1-hexene (0.5 mL, 4.00 mmol, 103 equiv.) and irradiated with LEDs showing wavelengths of λ = 365 nm or 460 nm. The reaction was monitored *via* ^{31}P NMR and after 30 min diphosphane **6** was the main product in both cases (spectroscopic yield: >75%; $HPtBu_2$ was detected as the major side product with a spectroscopic yield of <10%).

In a separate experiment, **3** (25 mg, 0.0389 mmol, 1.00 equiv.) was dissolved in 1-hexene (0.5 mL, 4.00 mmol, 103 equiv.) and irradiated with a green LED (525 nm). Reaction monitoring *via* ^{31}P NMR spectroscopy showed that the maximum yield of **9-*n*Bu** was achieved after 2 h (**9-*n*Bu** : **3** : **6** : $HPtBu_2$ = 27:40:22:11, corresponding to a spectroscopic yield of 32% for **9-*n*Bu**). However, **9-*n*Bu** could so far not be separated from remaining **3** and further irradiation with a green LED (525 nm) led to decomposition of both, **3** and **9-*n*Bu** (e.g.: after 4 h, **9-*n*Bu** : **3** : **6** : $HPtBu_2$ = 17:26:43:13). Thus, the reaction was performed under ambient light when aiming at the isolation of **9-nBu** (see main text).

9-*n*Pr:
$^{31}P\{^1H\}$-NMR (1-pentene, 298 K, ppm, 202.5 MHz): 22.4 (s, P_3), 78.6 (s, P_2), 82.7 (s, P_1).

9-*n*Bu:
$^{31}P\{^1H\}$-NMR (1-hexene, 298 K, ppm, 202.5 MHz): 21.2 (s, P_3), 78.5 (s, P_2), 82.7 (s, P_1).

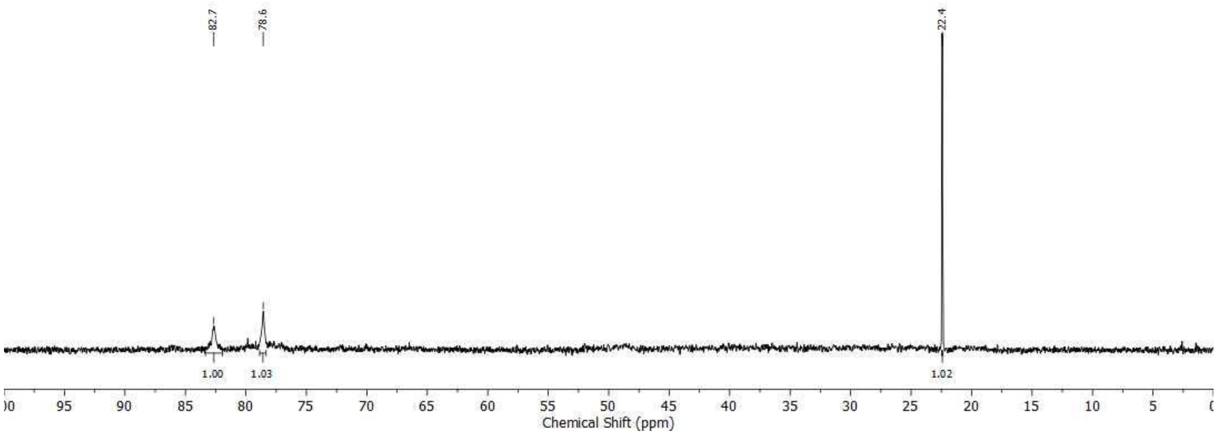

Figure S14. $^{31}P\{^1H\}$-NMR spectrum of **9-*n*Pr** in 1-pentene (298 K, 202 MHz).

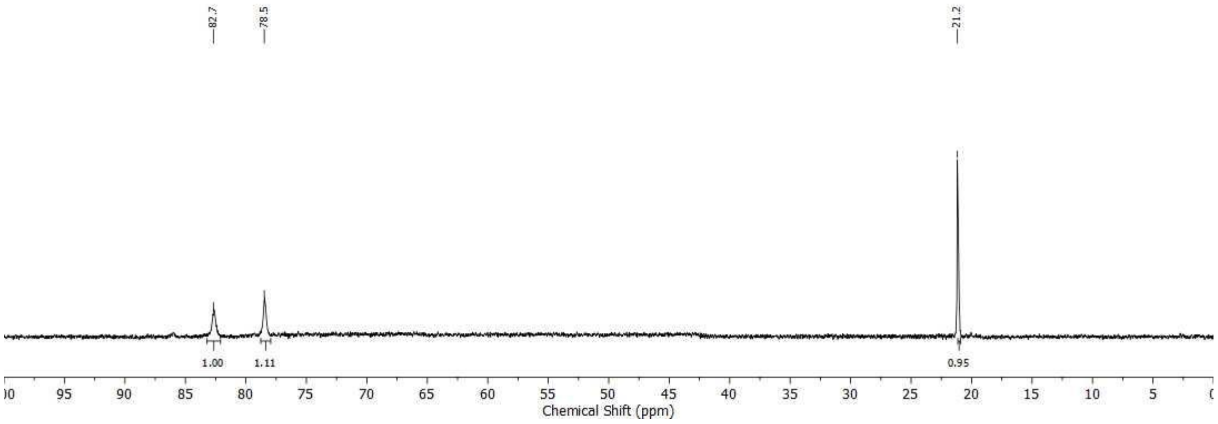

Figure S15. $^{31}P\{^1H\}$-NMR spectrum of **9-*n*Bu** in 1-hexene (298 K, 202 MHz).

Reaction towards gaseous substrates

Compound **3** (300 mg, 0.47 mmol, 1.00 equiv.) was dissolved in benzene (6 mL). The solution was frozen in liquid nitrogen, and the gas phase was removed under reduced pressure. While under cooling, the gaseous alkenes (1-butene or 3,3,3-trifluoropropene) were led into the flask and the solution was slowly warmed to room temperature with a pressure valve attached set to keep the pressure at a maximum value of 1 atm. This process was repeated two more times. The reactions were monitored via ^{31}P NMR spectroscopy. Smaller scale reactions in deuterated solvents gave identical results.

In the case of the reaction with 3,3,3-trifluoropropene a full conversion was achieved after 8.25 d, no equilibrium conditions were observed and a classical work-up was carried out. By removing the solvent und reduced pressure, re-dissolving the orange powder in *n*-pentane (1 mL) and storing the solution −30 °C for 5 days an orange crude product was obtained (containing single-crystals of **9-CF$_3$**). After 9 re-crystallization cycles, an orange solid was

obtained (25 mg, 0.03 mmol, 7%) which was pure by ^{31}P NMR spectroscopy. Compared to that, the spectroscopic yield is higher (**9-CF$_3$**: 42%), but due to the decomposition of the compounds in solution and the number of re-crystallization steps, the yield isolated material is reduced.

In the case of the reaction with 1-butene, no full conversion was observed after 10 d, with 40% of the starting material remaining in solution. Besides the desired product **9-Et** (11%) and the starting material **3** (4%), only the corresponding diphosphane **6** (54%) and the phosphane HP*t*Bu$_2$ (31%) were detected via ^{31}P NMR spectroscopy.

9-CF$_3$ (pure by ^{31}P NMR spectroscopy, additional resonances due to fluorinated hydrocarbons appear in the ^{1}H NMR spectrum and are not included in the list of signal assignments below, also see Figure S16 and main text):
^{1}H-NMR (C_6D_6, 298 K, ppm, 500 MHz): 1.58 (d, 18H, $^{3}J_{PH}$ = 11.1 Hz, C–CH_3), 1.80 (d, 18H, $^{3}J_{PH}$ = 10.8 Hz, C–CH_3), 2.18–2.24 (m, 21H, C–CH_3 + HC=CH_2 + *H*C=CH$_2$).
^{31}P{^{1}H}-NMR (C_6D_6, 298 K, ppm, 202.5 MHz): 33.4 (q, $^{4}J_{PF}$ = 17.8 Hz P_3), 85.4 (s, P_2), 91.9 (s, P_1).
Elemental analysis: calcd. (%) for $C_{27}H_{57}BiF_3P_3$ (740.65 g·mol^{-1}): C 43.79 H 7.76; calcd. (%) for $C_{27}H_{57}BiF_3P_3$ +2 x $C_3H_3F_3$:[b] C 42.49 H 6.81; found: C 42.73 H 6.61.
b: The result of the elemental analysis confirms that compound **9-CF$_3$** is contaminated with fluorinated hydrocarbons as supported by ^{1}H NMR spectroscopy and HR-MS analyses.
ESI(+) MS: found 71.0498, 145.122, 208.9794, 281.0367, 298.0313, 353.0942, 354.0939, 370.0888, 393.0420, 450.1122, 469.1107, 501.1011, 557.1636, 595.2272, 691.2452, 707.2397 m/z, calcd. 71,0491 ($H_7C_4O_1$), 145,1223 ($H_{17}C_8O_2$), 208.9794 (Bi), 281.0383 ($C_7H_7F_{10}$), 298.0318 ($C_4H_{10}Bi_1P_1$), 353.0946 ($C_{14}H_{14}F_9$), 354.0944 ($Bi_1P_1C_8H_{18}$), 370.0894 ($Bi_1P_1O_1C_8H_{18}$), 393.0427 ($Bi_1P_1F_3C_7H_{12}$) 450.1131 ($Bi_1P_1C_{11}H_{21}F_3$), 469.1121 ($Bi_1P_1C_{11}H_{21}F_4$), 501.1069 ($C_{15}H_{17}F_{16}$), 557.1695 ($C_{19}H_{25}F_{16}$), 595.227($Bi_1P_2F_3C_{19}H_{39}$) 691.2464 ($Bi_1P_2F_6C_{22}H_{42}$), 707.2413 ($Bi_1P_2F_6C_{22}H_{42}O_1$) m/z.

9-Et (detected in a mixture of compounds, *vide supra*)
^{31}P{^{1}H}-NMR (C_6D_6, 298 K, ppm, 202.5 MHz): 26.8 (s, P_3), 78.5 (s, P_2), 82.2 (s, P_1).

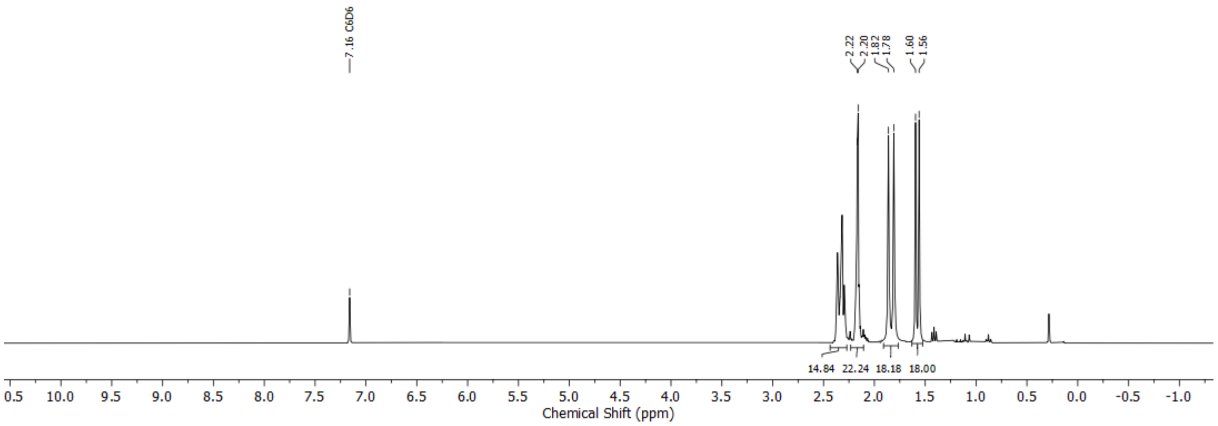

Figure S16. ^{1}H-NMR spectrum of **9-CF$_3$** in C_6D_6 (298 K, 500 MHz).

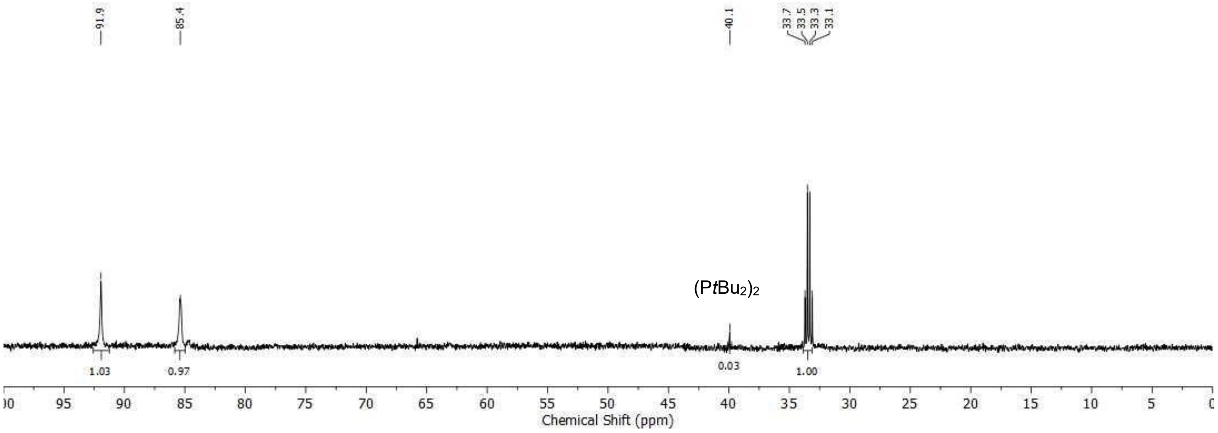

Figure S17. ^{31}P{^{1}H}-NMR spectrum of **9-CF$_3$** in C_6D_6 (298 K, 202 MHz).

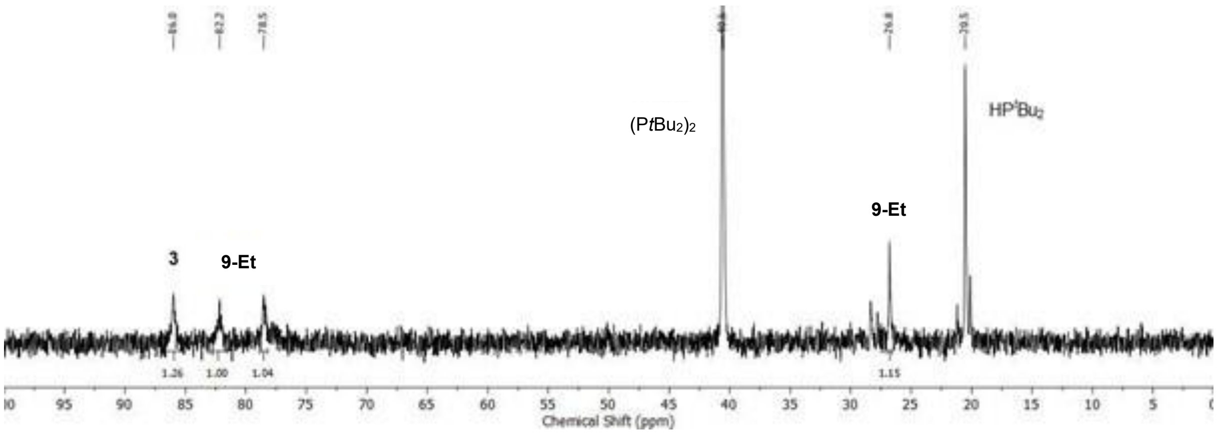

Figure S18. ^{31}P{^{1}H}-NMR spectrum of the insertion product of 1-butene into [Bi(P*t*Bu$_2$)$_3$] (**3**) in C_6D_6 (298 K, 202 MHz).

Exchange reaction with 1-hexene and 1-pentene

9-*n*Pr (15 mg, 0.0210 mmol, 1.0 equiv.) was dissolved in 1-hexene (0.4 mL, 3.18 mmol, 152 equiv.) and was stirred for 4.5 days. During this time, small amounts of a dark solid precipitated, presumably elemental bismuth. The suspension was filtered, and the yellow solution was analyzed via ^{31}P NMR spectroscopy.
Besides the starting material **9-*n*Pr** (20%) the diphosphane (74%) and **9-*n*Bu** (6%) were formed.

9-*n*Bu (15 mg, 0.0210 mmol, 1.0 equiv.) was dissolved in 1-hexene (0.4 mL, 3.66 mmol, 174 equiv.) and was stirred for 4.5 days. During this time, small amounts of a dark solid precipitated, presumably elemental bismuth. The suspension was filtered, and the yellow solution was analyzed via ^{31}P NMR spectroscopy.
Besides the starting material **9-*n*Bu** (14%) the diphosphane (67%) and **9-*n*Pr** (19%) were formed.

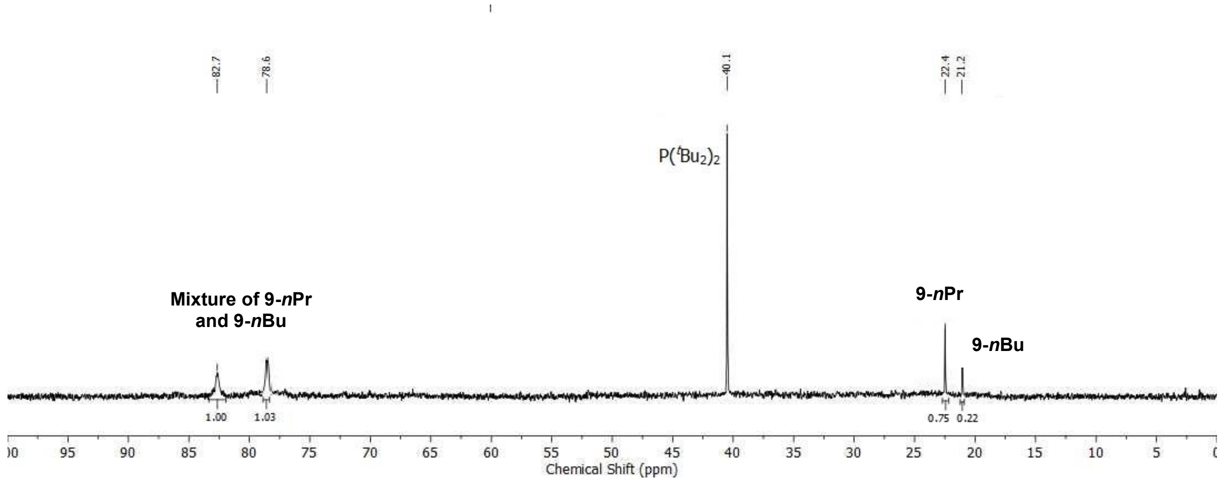

Figure S19. ^{31}P{^{1}H}-NMR spectrum of the exchange from **9-*n*Pr** to **9-*n*Bu** in 1-hexene (298 K, 202 MHz).

Experiments of 3 with 1-hexene: variation of molar ratio of starting materials

To test the impact of excess olefin on the outcome of the insertion reaction between **3** and 1-hexene, **3** was reacted with different amounts of 1-hexene in C_6D_6 solution in independent experiments. Specifically, four reactions have been carried out. **3** (25 mg, 0.0389 mmol, 1.00 equiv.) was dissolved C_6D_6 (0.5 mL) and different amounts of 1-hexene (1.0 equiv.; 5 equiv., 10 equiv.; 50 equiv.) were added. The reactions were kept at ambient light and room temperature for seven days and were monitored regularly via ^{31}P NMR spectroscopy. During

this time, small amounts of a dark solid precipitated, presumably elemental bismuth. The following ratios were observed after 7 days:

1:1 (**3**:1-hexene): Starting material **3** (25%) the diphosphane (67%) and **9-*n*Bu** (8%) were formed.

1:5 (**3**:1-hexene): Starting material **3** (22%) the diphosphane (65%) and **9-*n*Bu** (13%) were formed.

1:10 (**3**:1-hexene): Starting material **3** (19%) the diphosphane (68%) and **9-*n*Bu** (13%) were formed.

1:50 (**3**:1-hexene): Starting material **3** (14%) the diphosphane (64%) and **9-*n*Bu** (22%) were formed.

This gives further evidence for the reaction between **3** and 1-hexene being an equilibrium reaction.

Hydrogen atom transfer (HAT) reactivity

To a solution of 8 mg of **2** (0.0111 mol, 1.0 equiv) or **3** (0.0124 mol, 1.0 equiv.) in C_6D_6 (0.5 mL), 20 equiv. of C–H donor substrates (Table S2) were added. After stirring the solution for 1 hour, the reaction mixture was monitored regularly by $^{31}P\{^1H\}$ NMR for the formation of corresponding phosphane (δ(HP*t*Bu$_2$) = 20.0 ppm; δ(HP(*t*Bu)Cy) = −2.1 ppm).[2]

In reactions of **2** with H-atom donors, only for 1,4-cyclohexadiene, large amounts of the phosphane were generated. The formation of the diphosphane was always a significantly competing or even major reaction pathway.

In reactions of **3** with 1,4-cyclohexadiene (CHD), xanthene (XAN), and 9,10-dihydroanthracene (DHA) after 43 h, the corresponding phosphane was detected as the main product and only minor amounts of the decomposition product (diphosphane) were observed. While the reaction with fluorene (FLU) still showed significantly larger amounts of the phosphane (as compared to the diphosphane), the reaction with Ph_3CH led to only slightly larger yields of the phosphane (as compared to the diphosphane). The formation of other phosphorus-containing compounds (for instance due to reactions with the organic core of the hydrogen atom donors) was not observed.

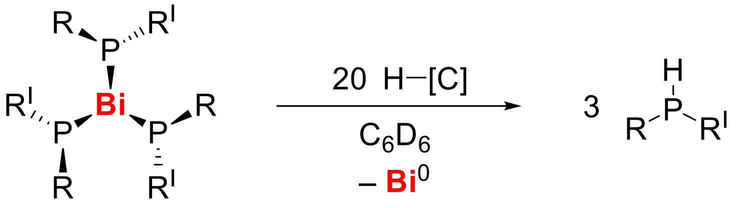

2 or **3**

Scheme S6. Reactivity of bismuth phosphanides **2** or **3** with H-atom donors (that require the scission of a C–H bond) to the corresponding phosphanes.

Table S3. Literature known bond dissociation free energies (BDFEs) of the H-atom donors used in this work. a = in the gas phase; b = in DMSO.[28]

Substrate		BDFE [kcal·mol^{-1}]
Full name	abbreviation	
1,4-cyclohexadiene	CHD	67.8^a
xanthene	XAN	70.3^b
9,10-dihydroanthracene	DHA	72.9^b
fluorene	FLU	74.3^b
triphenylmethane	Ph_3CH	75.7^b

Table S4. Conversion of **2** and **3** with H-atom donors to the corresponding phosphanes and diphosphanes after 43 h reaction time.

	**Compound 2**		**Compound 3**	
Substrate	Phosphane [%]	Diphosphane [%]	Phosphane [%]	Diphosphane [%]
CHD	34	28	90	3
XAN	30	57	83	2
DHA	25	29	54	2
FLU	11	51	23	12
Ph_3CH	17	58	23	20

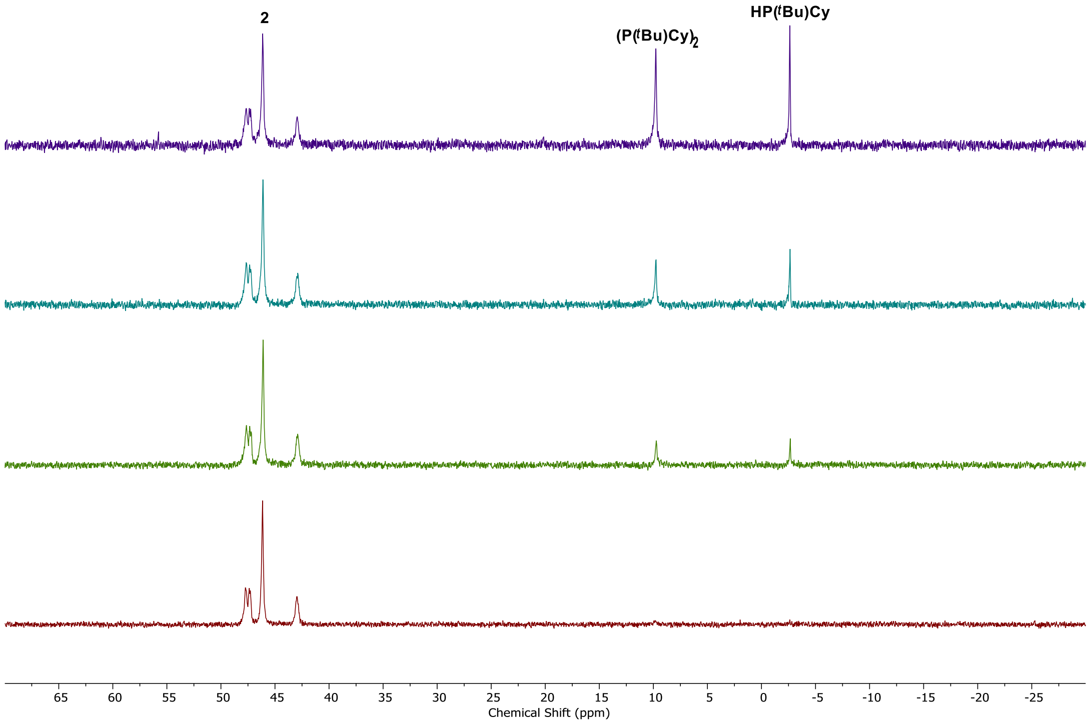

Figure S20. $^{31}P\{^1H\}$ NMR spectrum of **2** with 20 equiv. 1,4-cyclohexadiene (CHD) in 0.5 mL C_6D_6 (298 K, 101.2 MHz). red: after 1 h. green: after 18 h. blue: after 24 h. purple: after 43 h.

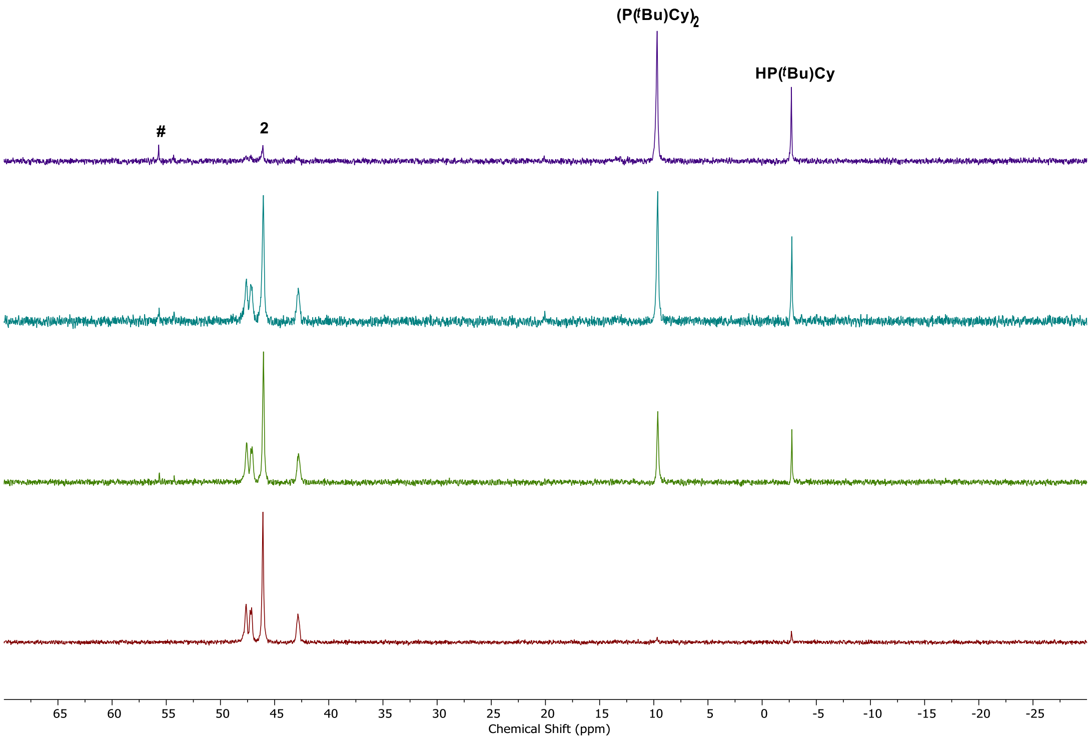

Figure S21. $^{31}P\{^{1}H\}$ NMR spectrum of **2** with 20 equiv. xanthene (XAN) in 0.5 mL C_6D_6 (298 K, 101.2 MHz). red: after 1 h. green: after 18 h. blue: after 24 h. purple: after 43 h. # unspecified side product.

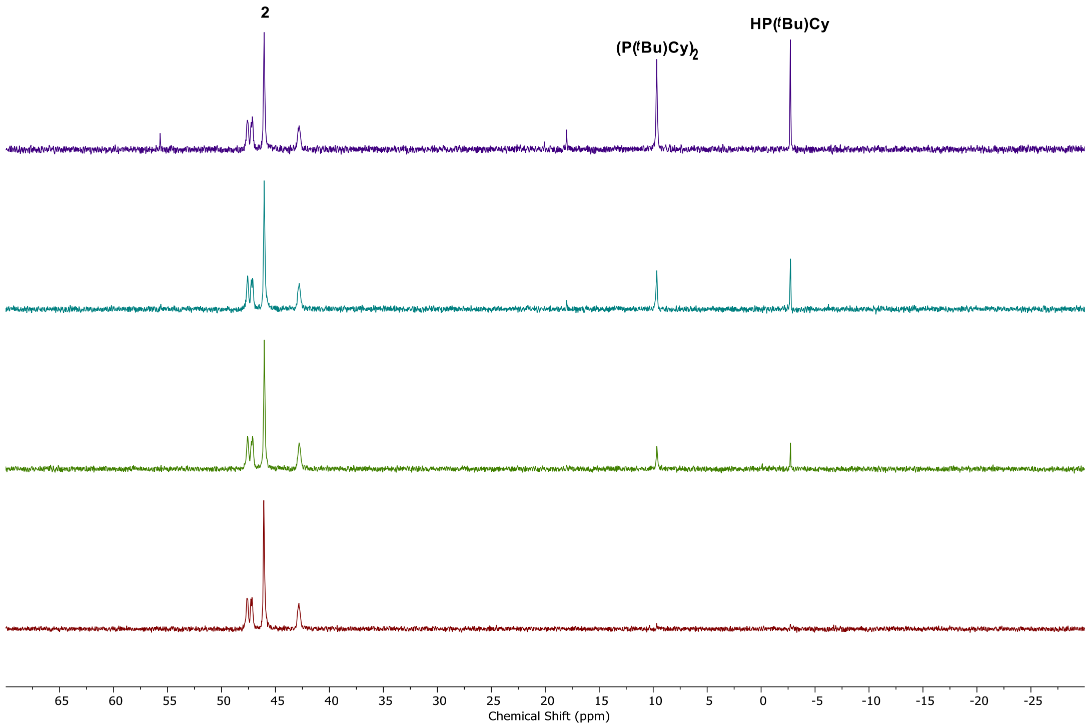

Figure S22. $^{31}P\{^{1}H\}$ NMR spectrum of **2** with 20 equiv. 9,10-dihydroanthracene (DHA) in 0.5 mL C_6D_6 (298 K, 101.2 MHz). red: after 1 h. green: after 18 h. blue: after 24 h. purple: after 43 h.

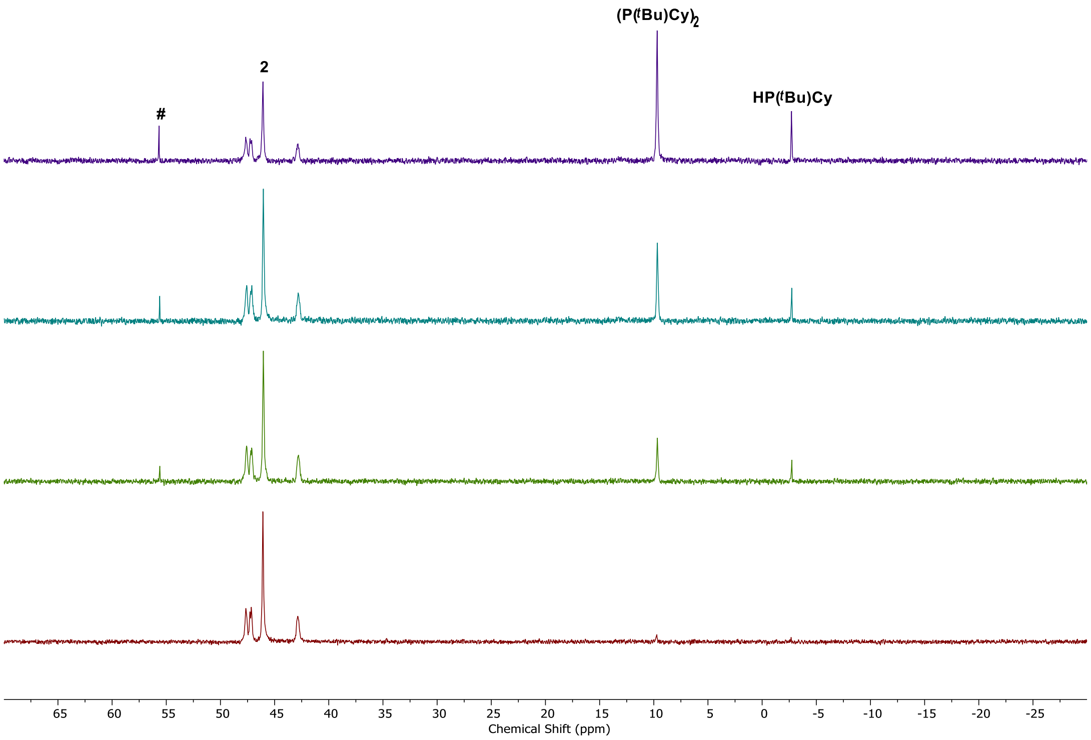

Figure S23. $^{31}P\{^1H\}$ NMR spectrum of **2** with 20 equiv. fluorene (FLU) in 0.5 mL C_6D_6 (298 K, 101.2 MHz). red: after 1 h. green: after 18 h. blue: after 24 h. purple: after 43 h. # unspecified side product.

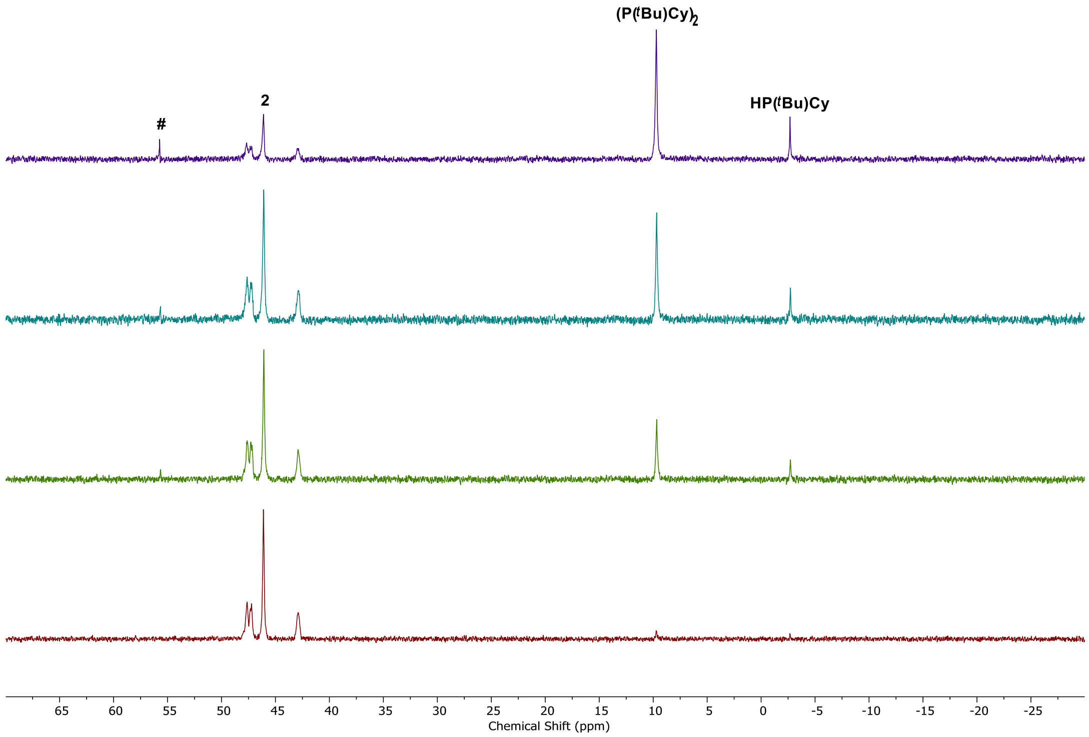

Figure S24. $^{31}P\{^1H\}$ NMR spectrum of **2** with 20 equiv. triphenylmethane (Ph_3CH) in 0.5 mL C_6D_6 (298 K, 101.2 MHz). red: after 1 h. green: after 18 h. blue: after 24 h. purple: after 43 h. # unspecified side product.

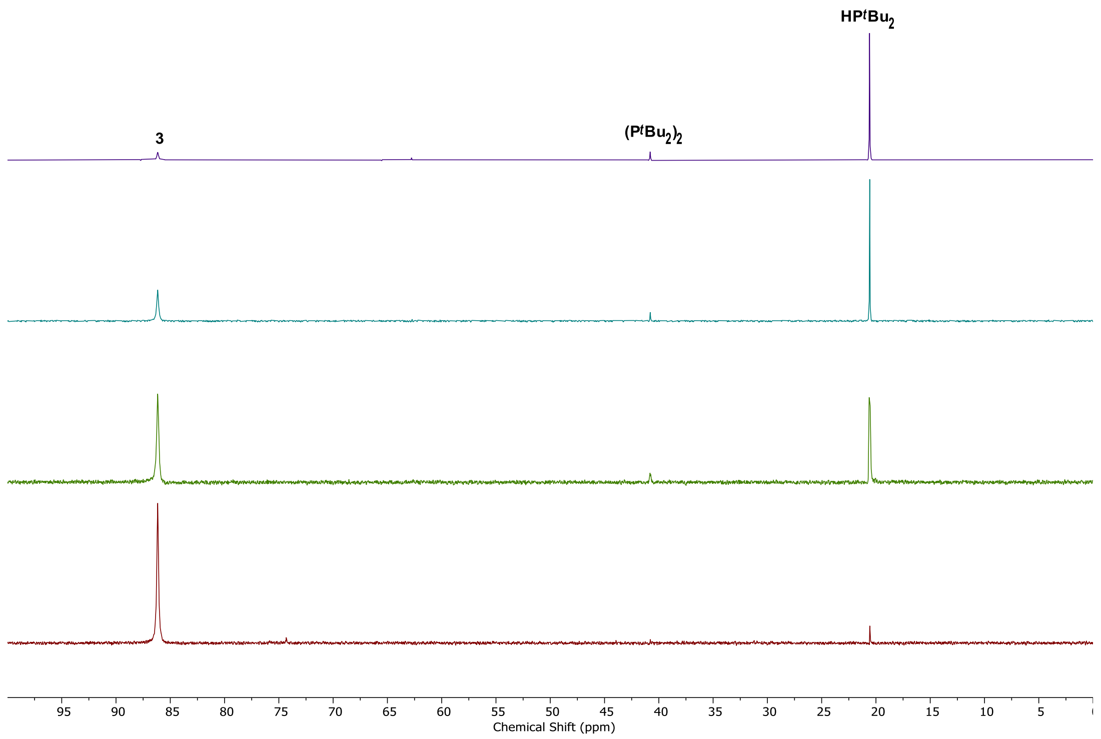

Figure S25. $^{31}P\{^1H\}$ NMR spectrum of **3** with 20 equiv. 1,4-cyclohexadiene (CHD) in 0.5 mL C_6D_6 (298 K, 101.2 MHz). red: after 1 h. green: after 18 h. blue: after 24 h. purple: after 43 h.

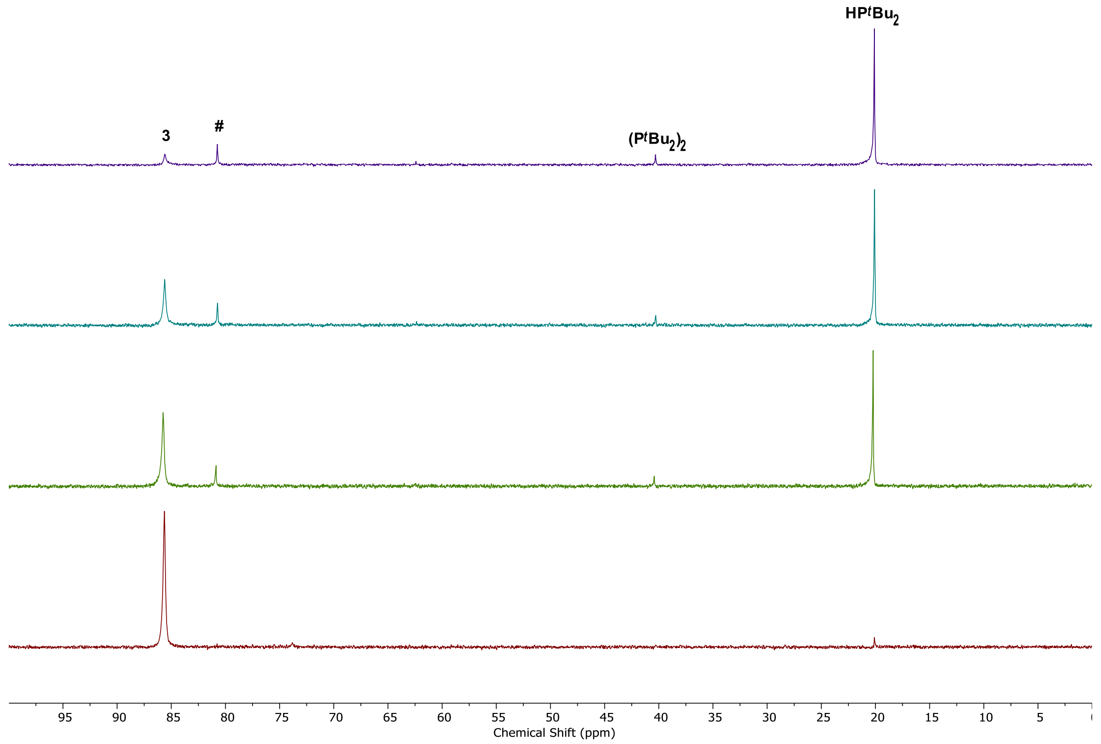

Figure S26. $^{31}P\{^1H\}$ NMR spectrum of **3** with 20 equiv. xanthene (XAN) in 0.5 mL C_6D_6 (298 K, 101.2 MHz). red: after 1 h. green: after 18 h. blue: after 24 h. purple: after 43 h. # unspecified side product.

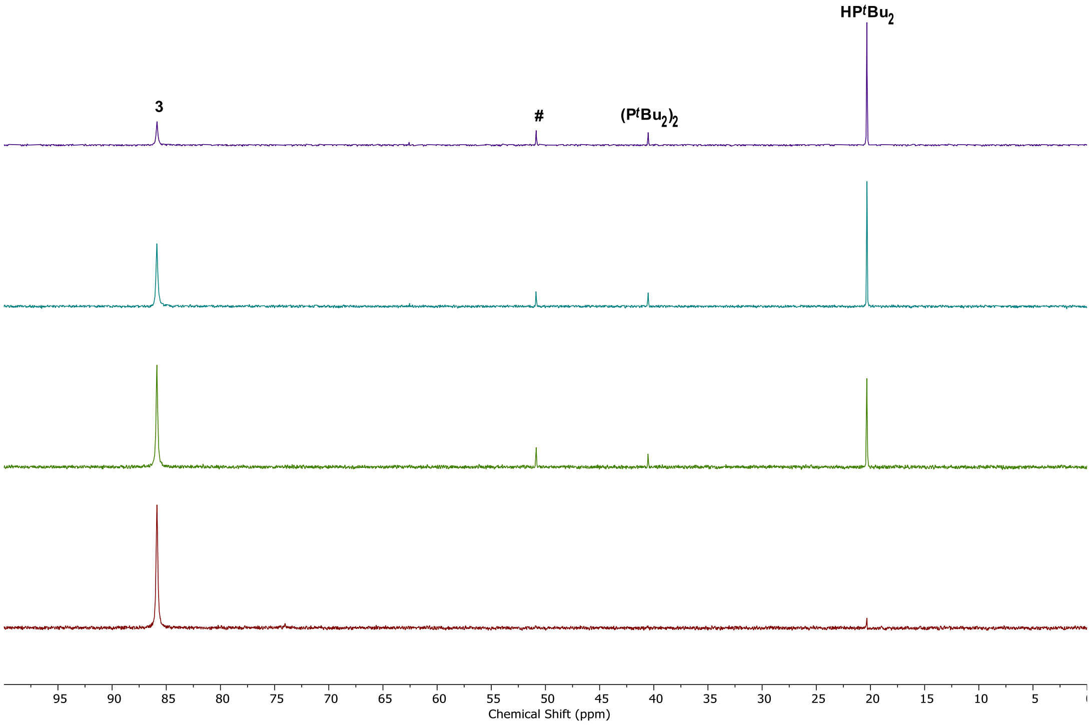

Figure S27. $^{31}P\{^1H\}$ NMR spectrum of **3** with 20 equiv. 9,10-dihydroanthracene (DHA) in 0.5 mL C_6D_6 (298 K, 101.2 MHz). red: after 1 h. green: after 18 h. blue: after 24 h. purple: after 43 h. # unspecified side product.

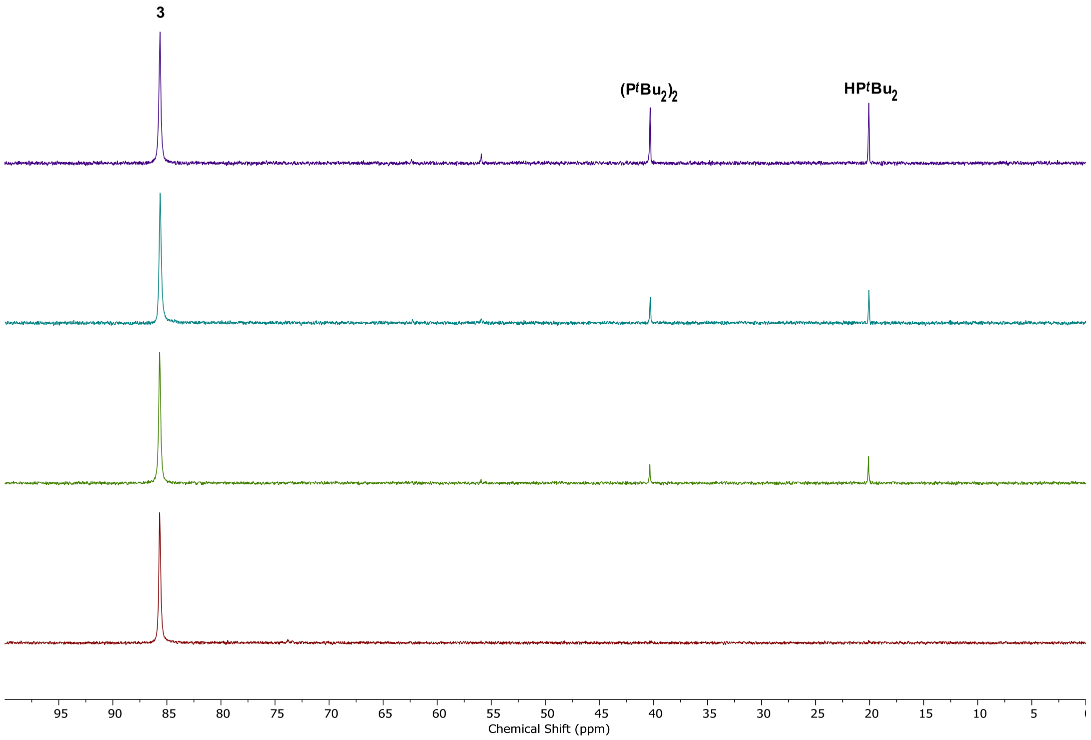

Figure S28. $^{31}P\{^1H\}$ NMR spectrum of **3** with 20 equiv. fluorene (FLU) in 0.5 mL C_6D_6 (298 K, 101.2 MHz). red: after 1 h. green: after 18 h. blue: after 24 h. purple: after 43 h.

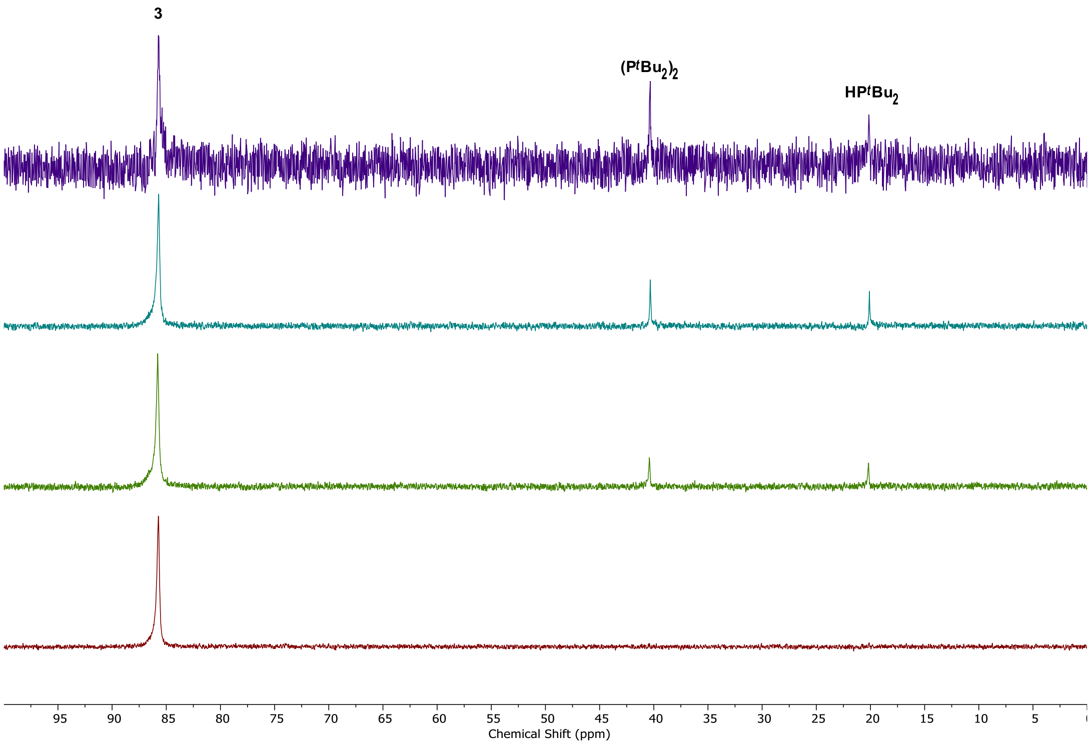

Figure S29. $^{31}P\{^{1}H\}$ NMR spectrum of **3** with 20 equiv. triphenylmethane (Ph_3CH) in 0.5 mL C_6D_6 (298 K, 101.2 MHz). red: after 1 h. green: after 18 h. blue: after 24 h. purple: after 43 h.

NMR spectra

Bi(PCy2)3 (1)

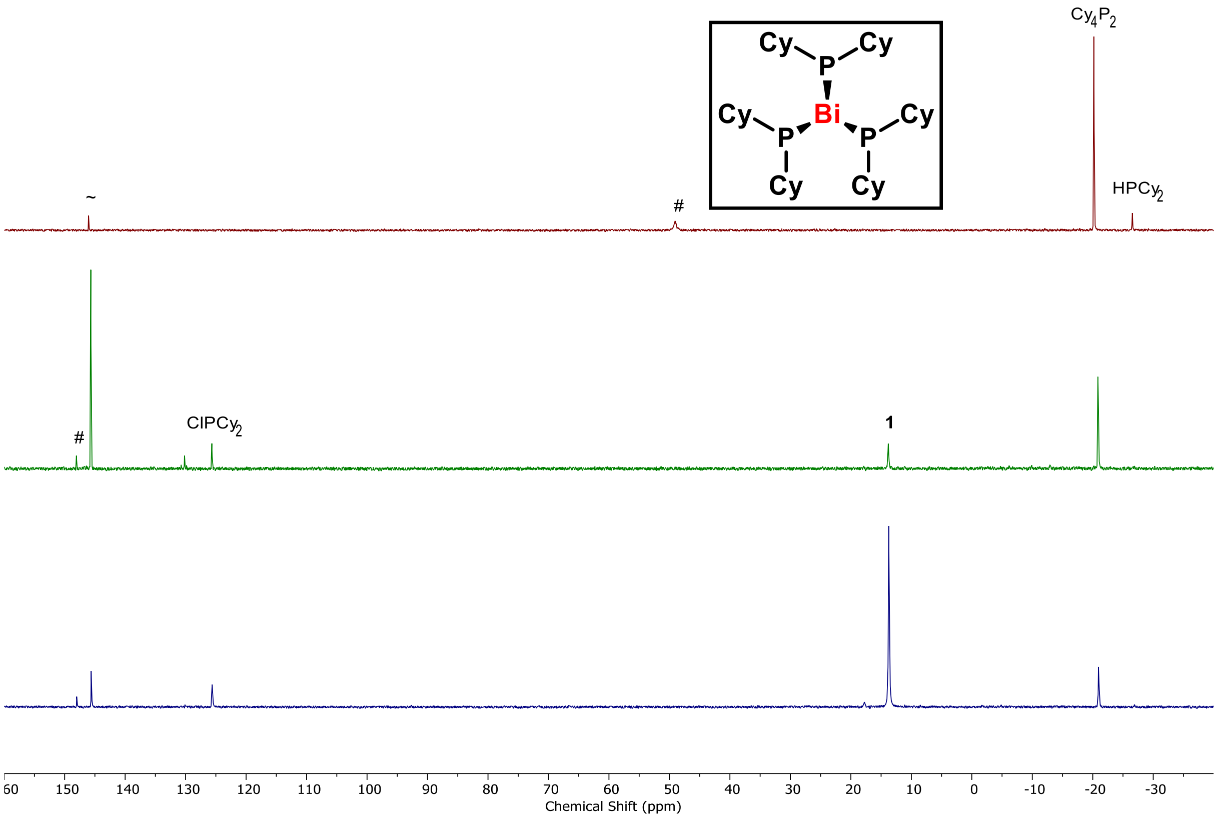

Figure S30. $^{31}P\{^1H\}$-NMR spectrum in C_6D_6 of the red solid obtained after crystallization of **1** (298 K, 101 MHz). blue: after 5 min in solution at rt. green: after 30 min in solution at rt. red: after 45 min in solution at rt. #: unspecified side products. ~: possible intermediate of decomposition reactions.

Bi(P(*t*Bu)Cy)3 (2)

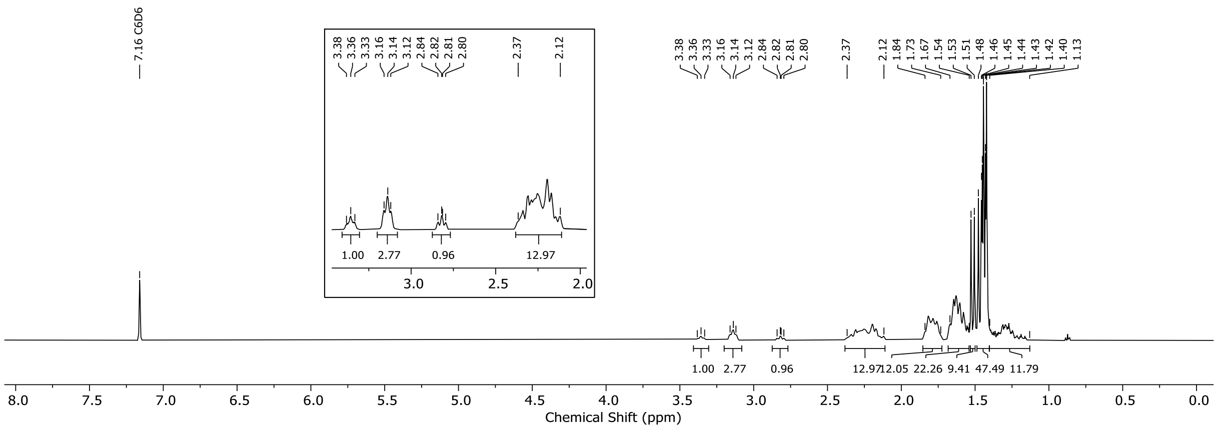

Figure S31. ^1H-NMR spectrum of [Bi(P(*t*Bu)Cy)3] (**2**) in C_6D_6 (298 K, 500 MHz).

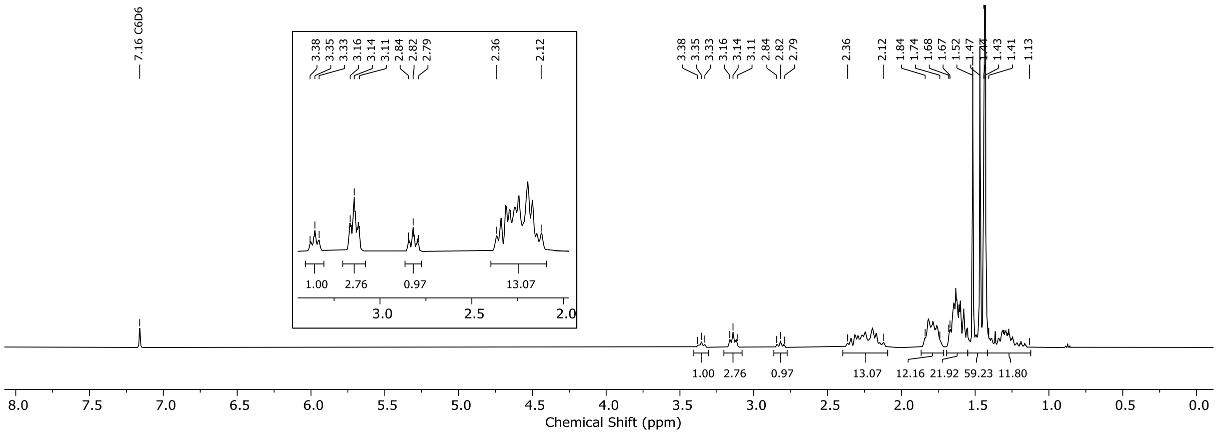

Figure S32. $^{1}H\{^{31}P\}$-NMR spectrum of [Bi(P(*t*Bu)Cy)$_3$] (**2**) in C_6D_6 (298 K, 500 MHz).

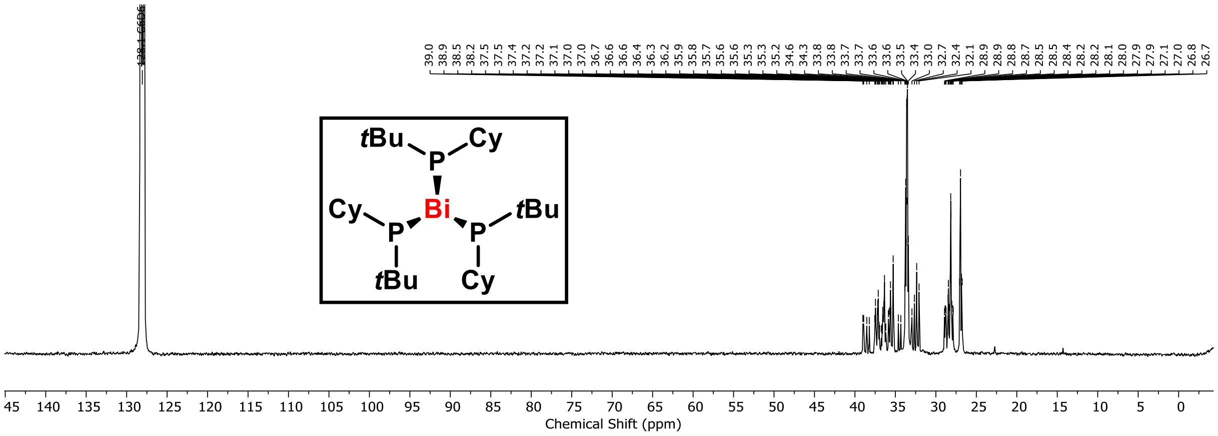

Figure S33. $^{13}C\{^{1}H\}$-NMR spectrum of [Bi(P(*t*Bu)Cy)$_3$] (**2**) in C_6D_6 (298 K, 500 MHz).

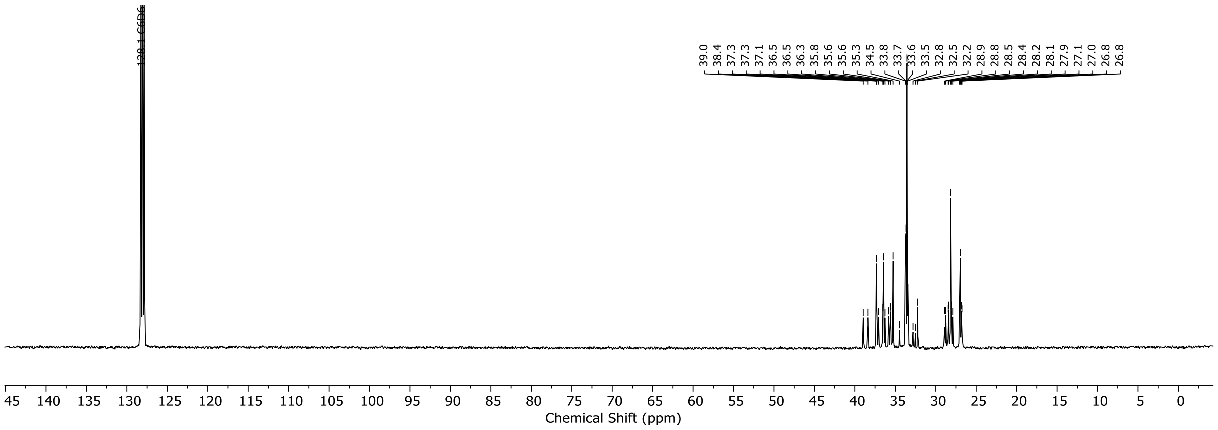

Figure S34. $^{13}C\{^{1}H,^{31}P\}$-NMR spectrum of [Bi(P(*t*Bu)Cy)$_3$] (**2**) in C_6D_6 (298 K, 125 MHz).

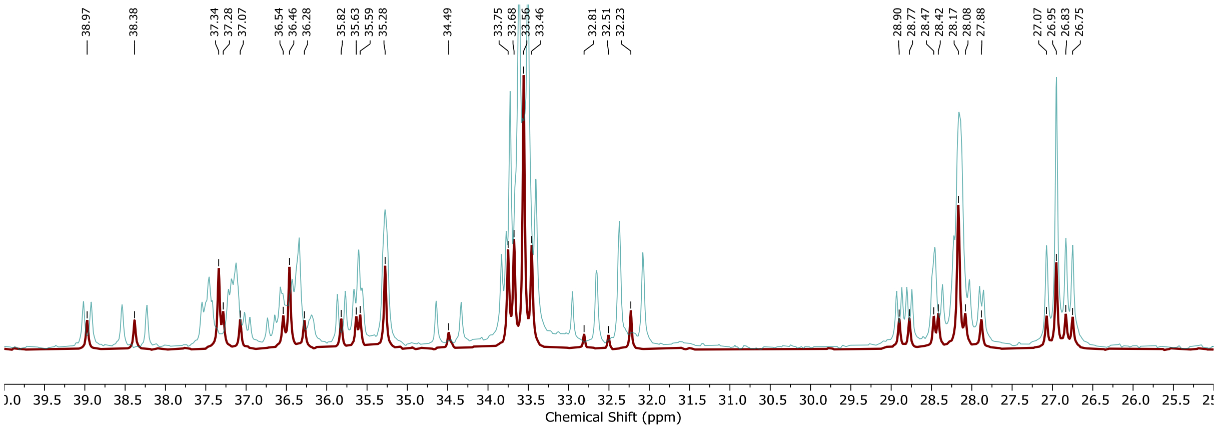

Figure S35. $^{13}C\{^{1}H,^{31}P\}$-NMR spectrum (red) and $^{13}C\{^{1}H\}$-NMR spectrum (blue) of [Bi(P(*t*Bu)Cy)$_3$] (**2**) in the region of 25–40 ppm in C_6D_6 (298 K, 125 MHz).

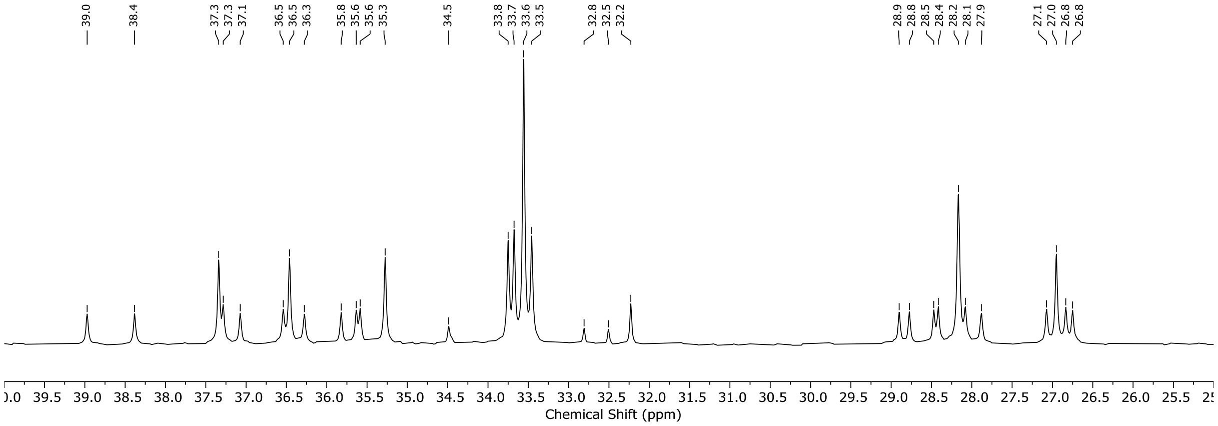

Figure S36. $^{13}C\{^{1}H,^{31}P\}$-NMR spectrum of [Bi(P(*t*Bu)Cy)$_3$] (**2**) in the region of 25–40 ppm in C_6D_6 (298 K, 125 MHz).

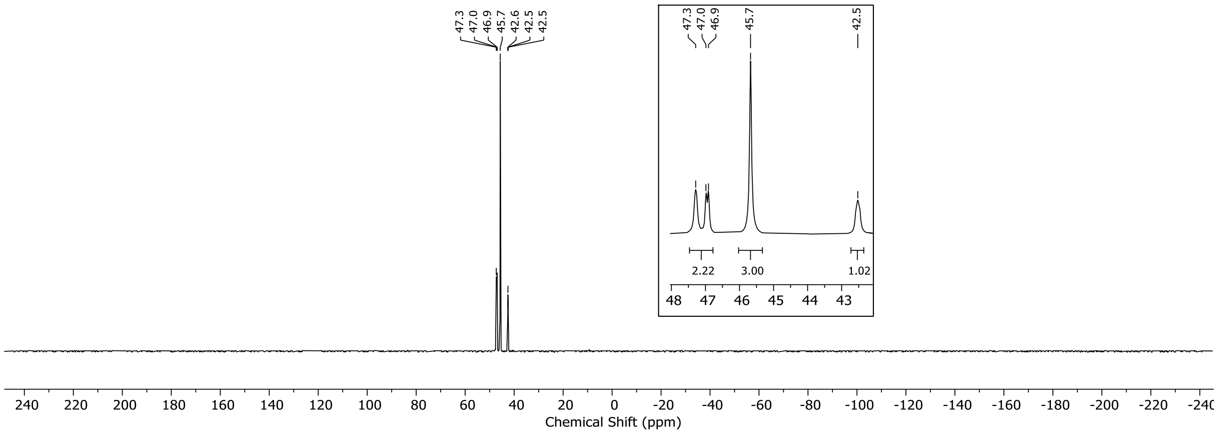

Figure S37. $^{31}P\{^{1}H\}$-NMR spectrum of [Bi(P(*t*Bu)Cy)$_3$] (**2**) in C_6D_6 (298 K, 202.5 MHz).

Bi(P*t*Bu$_2$)$_3$ (3)

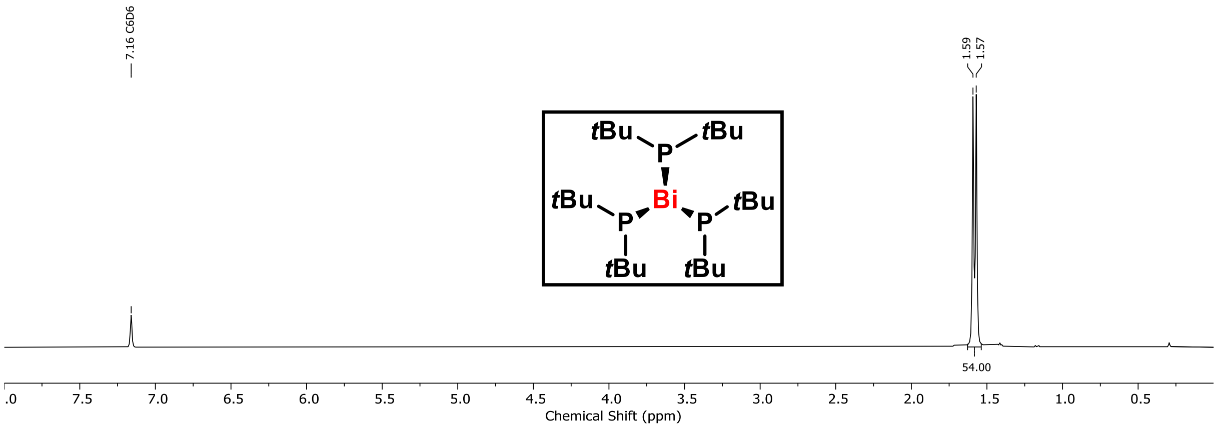

Figure S38. ^{1}H-NMR spectrum of [Bi(P*t*Bu$_2$)$_3$] (**3**) in C_6D_6 (298 K, 500 MHz).

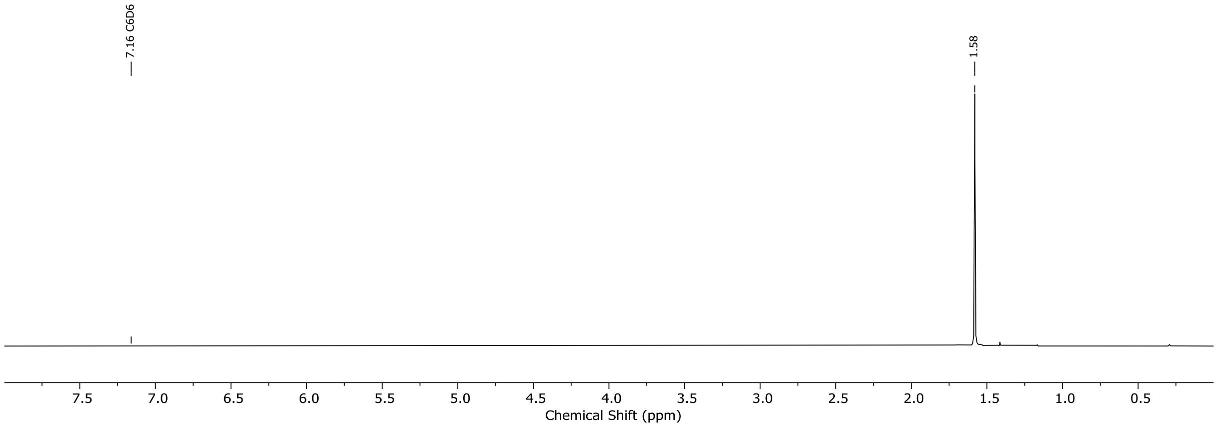

Figure S39. ^{1}H{^{31}P}-NMR spectrum of [Bi(P*t*Bu$_2$)$_3$] (**3**) in C_6D_6 (298 K, 500 MHz).

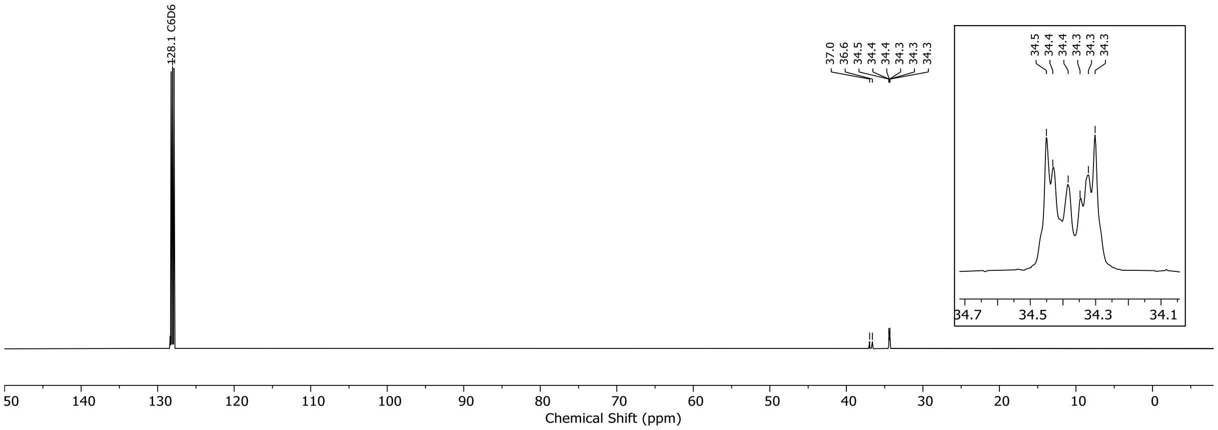

Figure S40. ^{13}C{^{1}H}-NMR spectrum of [Bi(P*t*Bu$_2$)$_3$] (**3**) in C_6D_6 (298 K, 125 MHz).

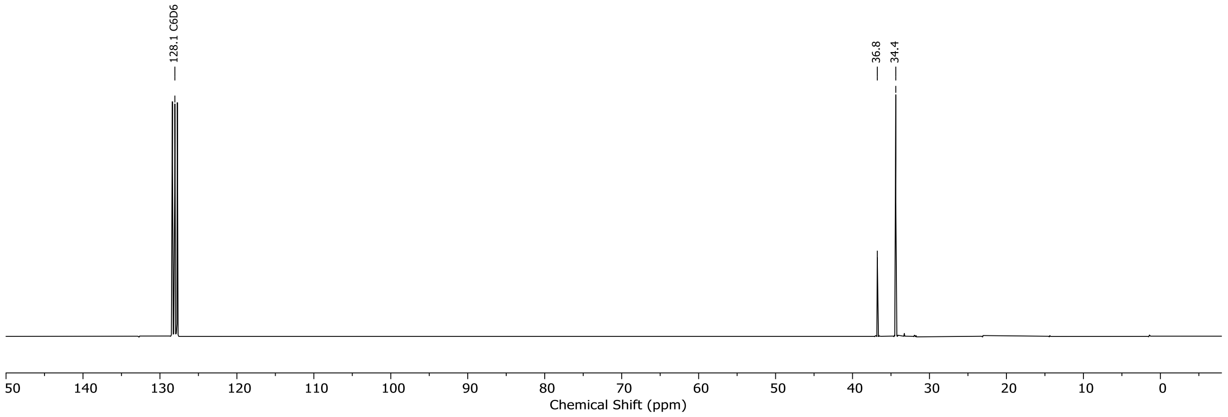

Figure S41. $^{13}C\{^{1}H, ^{31}P\}$-NMR spectrum of [Bi(P*t*Bu$_2$)$_3$] (**3**) in C_6D_6 (298 K, 125 MHz).

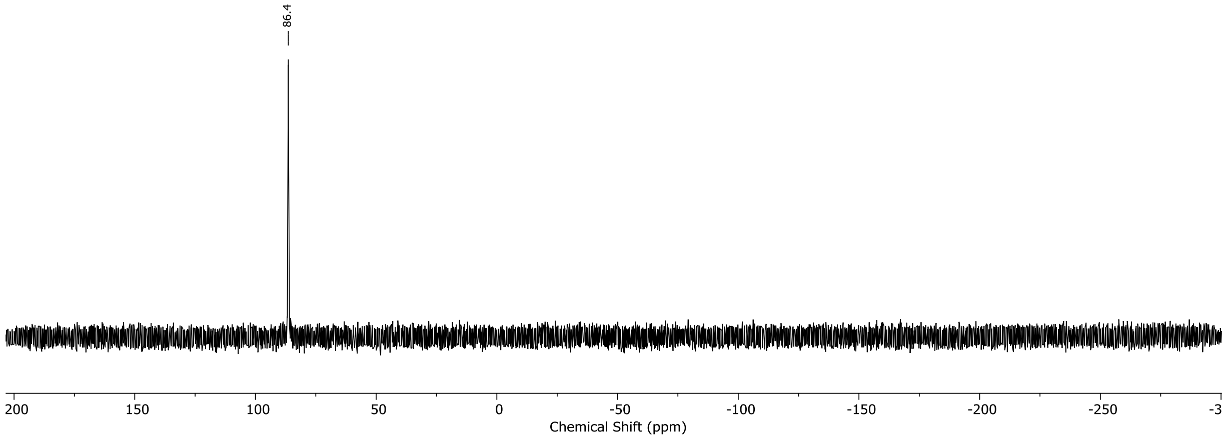

Figure S42. $^{31}P\{^{1}H\}$-NMR spectrum of [Bi(P*t*Bu$_2$)$_3$] (**3**) in C_6D_6 (298 K, 202.5 MHz).

Bi(PAd$_2$)$_3$ (4)

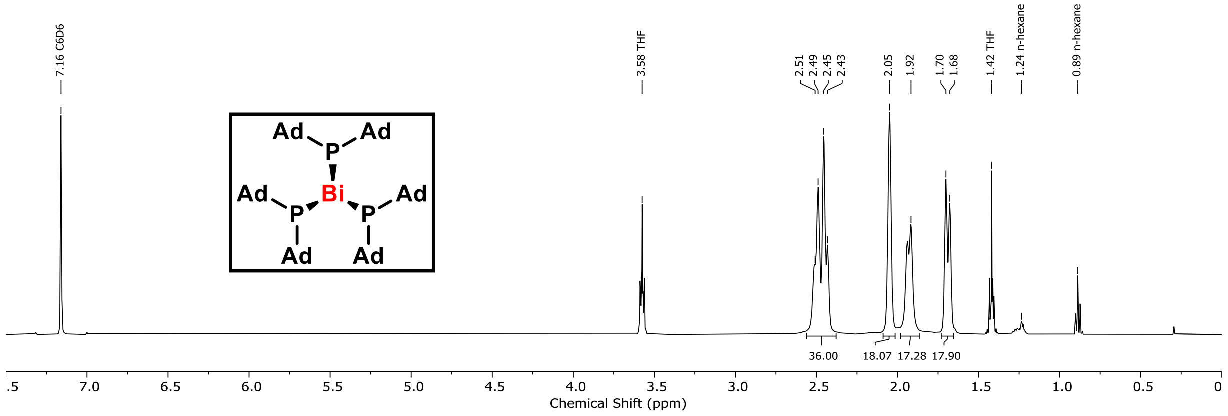

Figure S43. ^{1}H-NMR spectrum of [Bi(PAd$_2$)$_3$] (**4**) in C_6D_6 (298 K, 500 MHz).

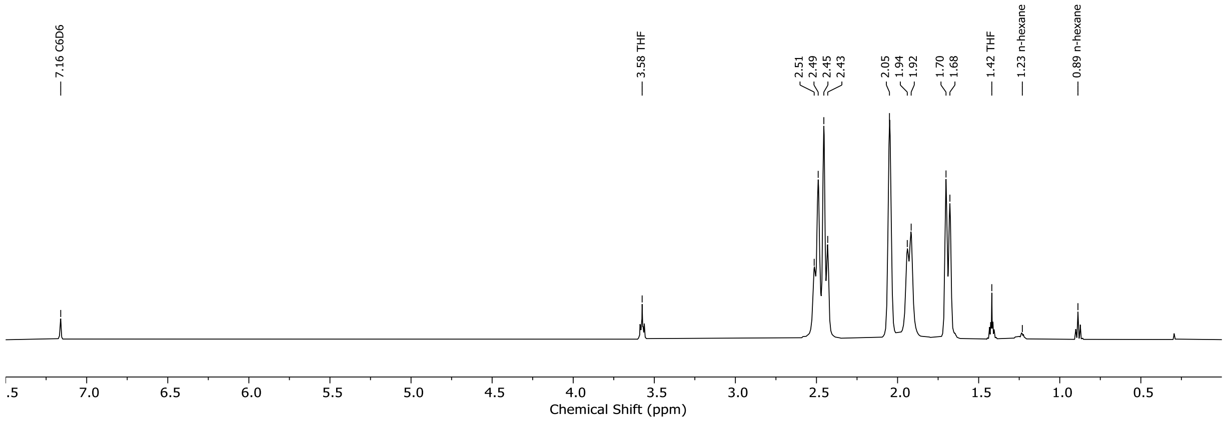

Figure S44. $^{1}H\{^{31}P\}$-NMR spectrum of $[Bi(PAd_2)_3]$ (**4**) in C_6D_6 (298 K, 500 MHz).

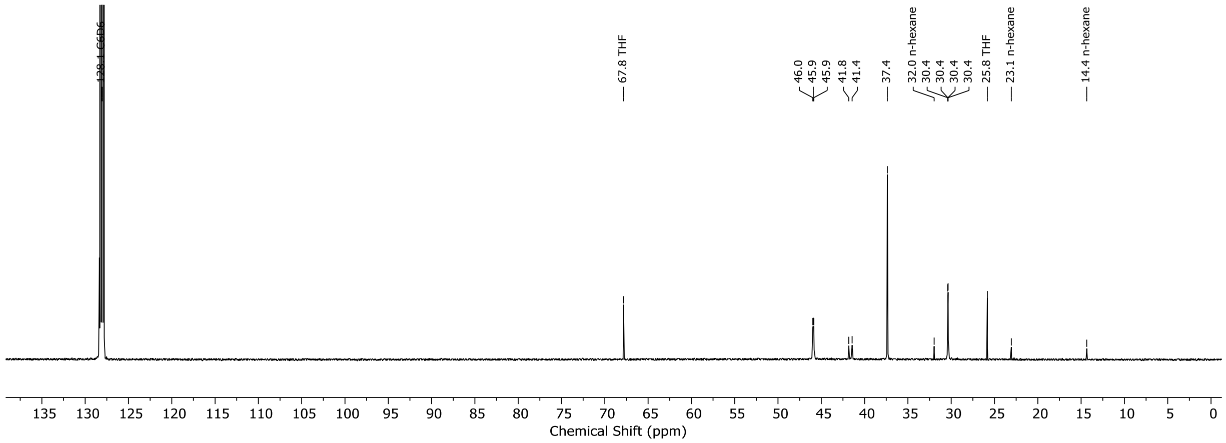

Figure S45. $^{13}C\{^{1}H\}$-NMR spectrum of $[Bi(PAd_2)_3]$ (**4**) in C_6D_6 (298 K, 125 MHz).

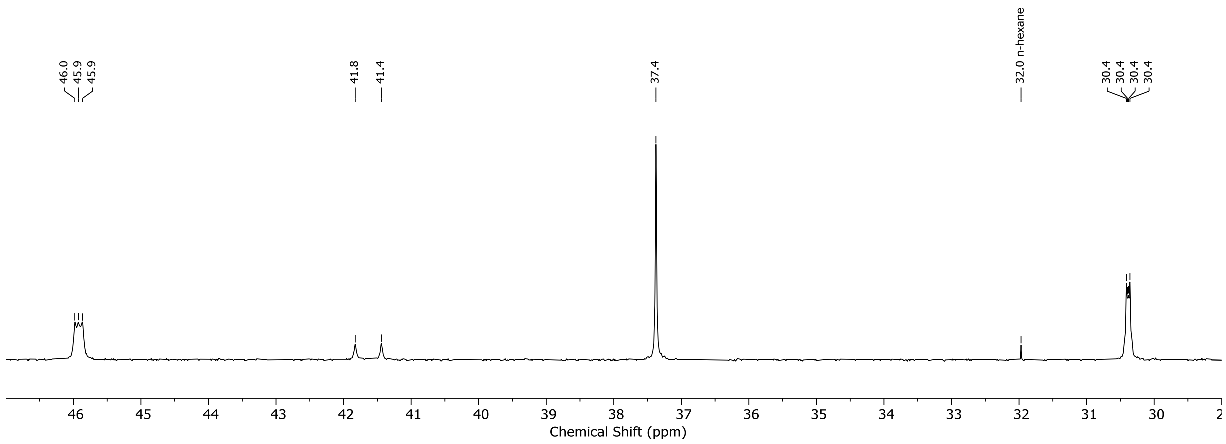

Figure S46. $^{13}C\{^{1}H\}$-NMR spectrum of $[Bi(PAd_2)_3]$ (**4**) in a region from 29–47 ppm in C_6D_6 (298 K, 125 MHz).

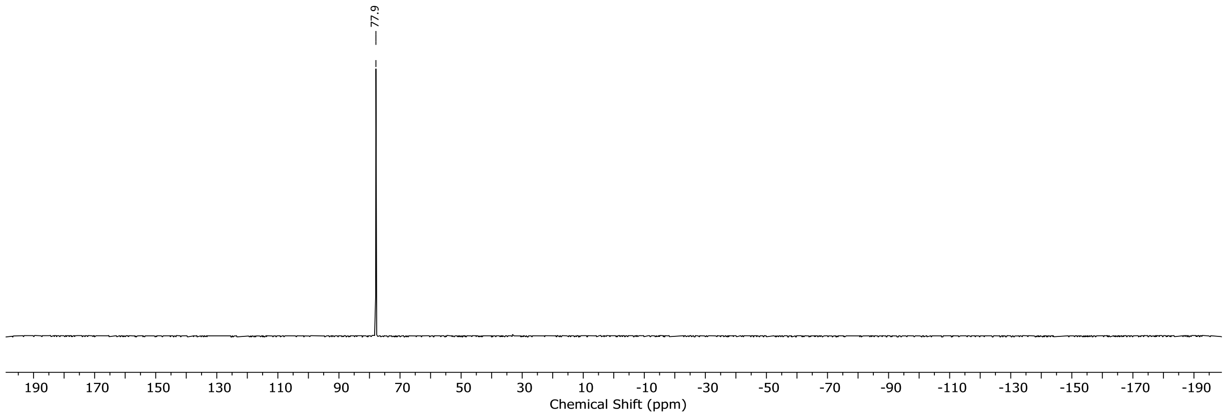

Figure S47. $^{31}P\{^{1}H\}$-NMR spectrum of $[Bi(PAd_2)_3]$ (**4**) in C_6D_6 (298 K, 202 MHz).

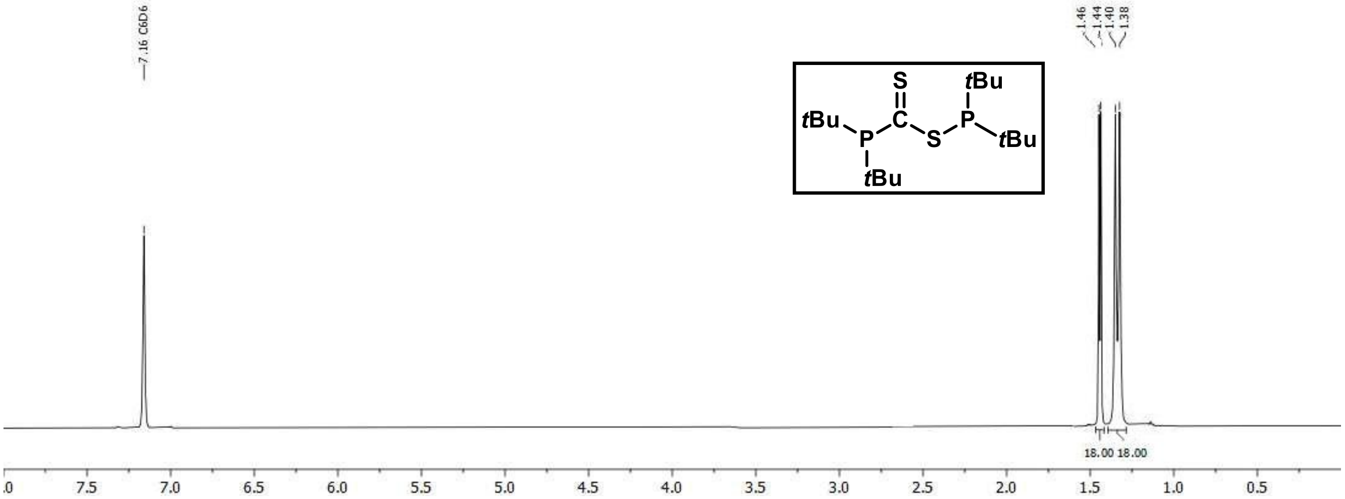

Figure S48. ^{1}H-NMR spectrum of **7** in C_6D_6 (298 K, 500 MHz).

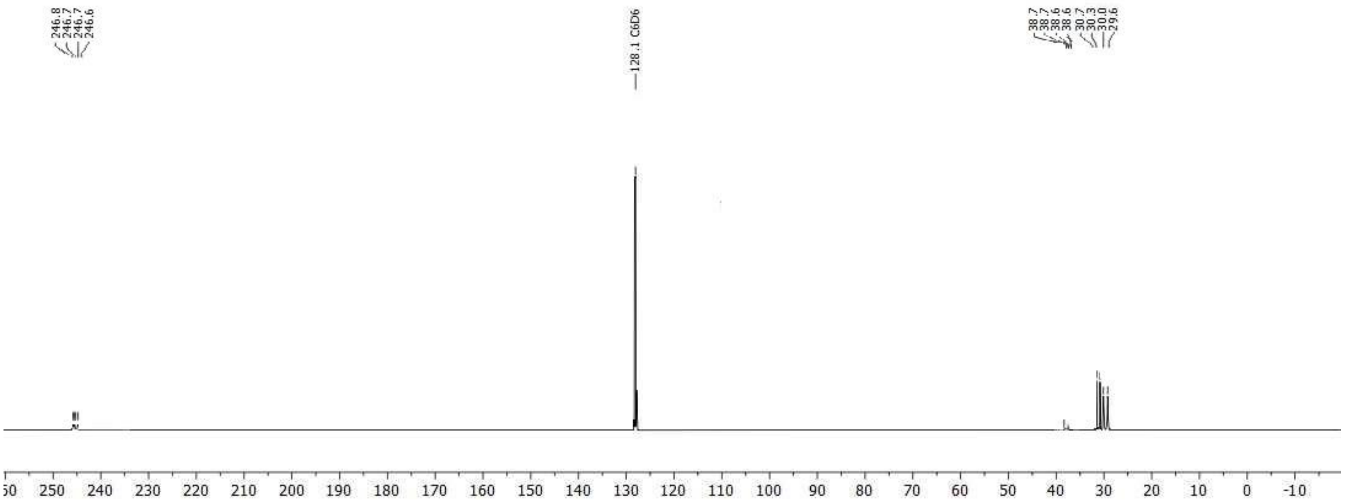

Figure S49. $^{13}C\{^{1}H\}$-NMR spectrum of **7** in C_6D_6 (298 K, 125 MHz).

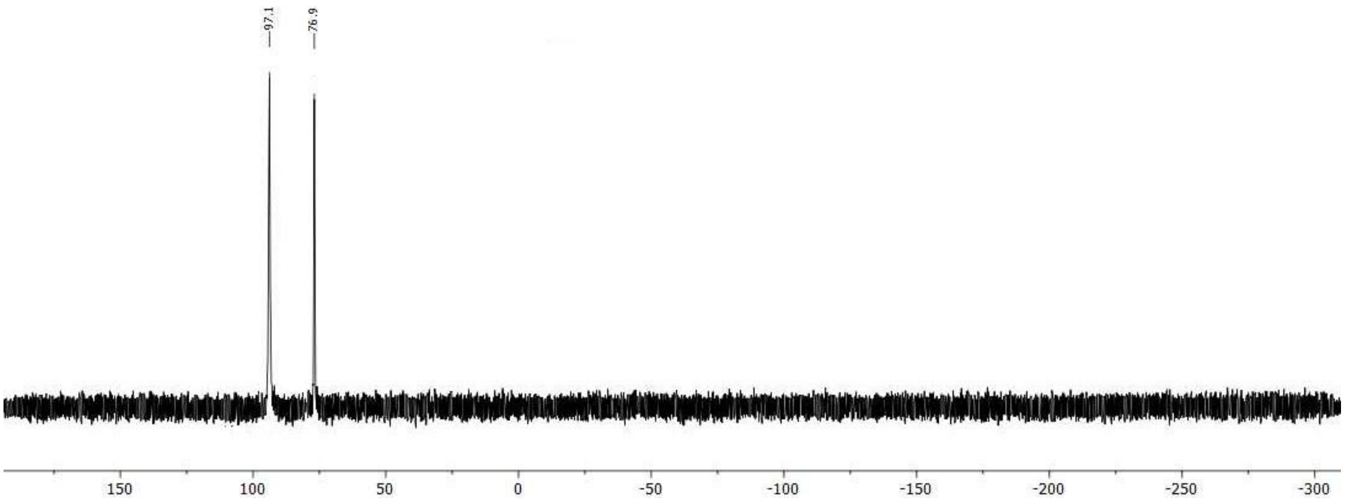

Figure S50. $^{31}P\{^1H\}$-NMR spectrum of **7** in C_6D_6 (298 K, 202 MHz).

UV/Vis spectra

The UV/VIS measurement were recorded on an *Analytik Jena Specord S600* using *WinASPECT* software. The event at around 680 nm is assigned to a lamp change.

Bi(P(*t*Bu)Cy)₃ (2)

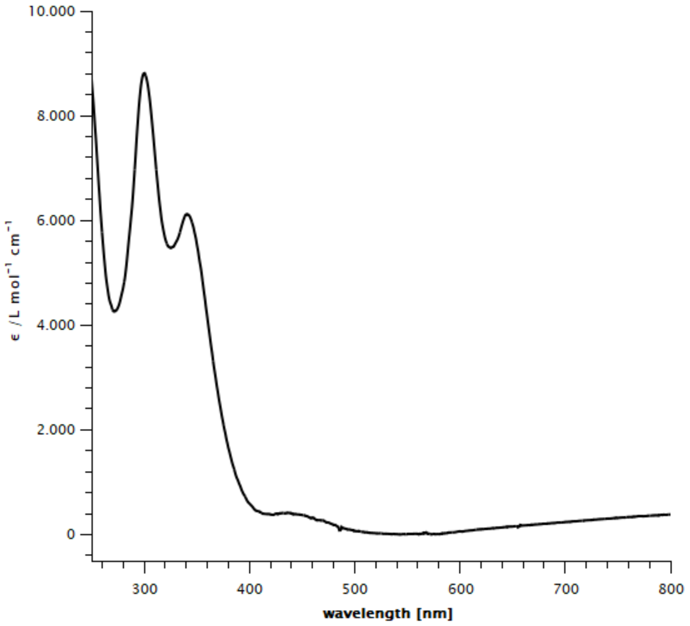

Figure S51. UV-VIS spectrum of [Bi(P(*t*Bu)Cy)$_3$] (**2**) in benzene.

Bi(P*t*Bu$_2$)$_3$ (3)

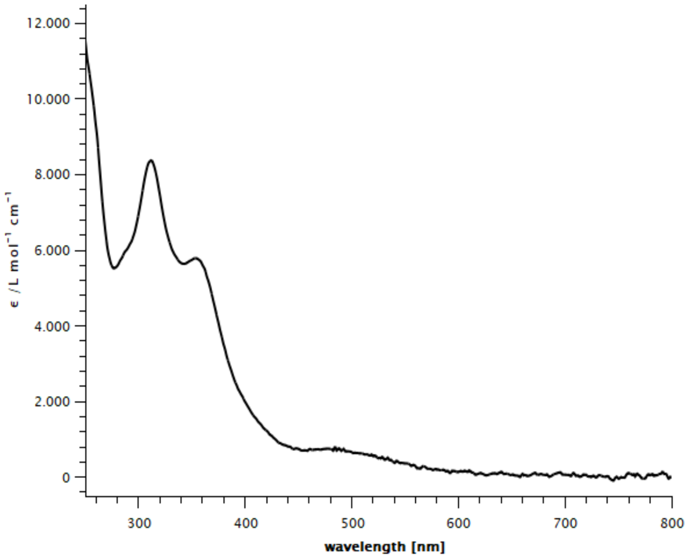

Figure S52. UV-VIS spectrum of [Bi(P*t*Bu$_2$)$_3$] (**3**) in benzene.

Mass spectra

Bi(P(*t*Bu)Cy)$_3$ (2)

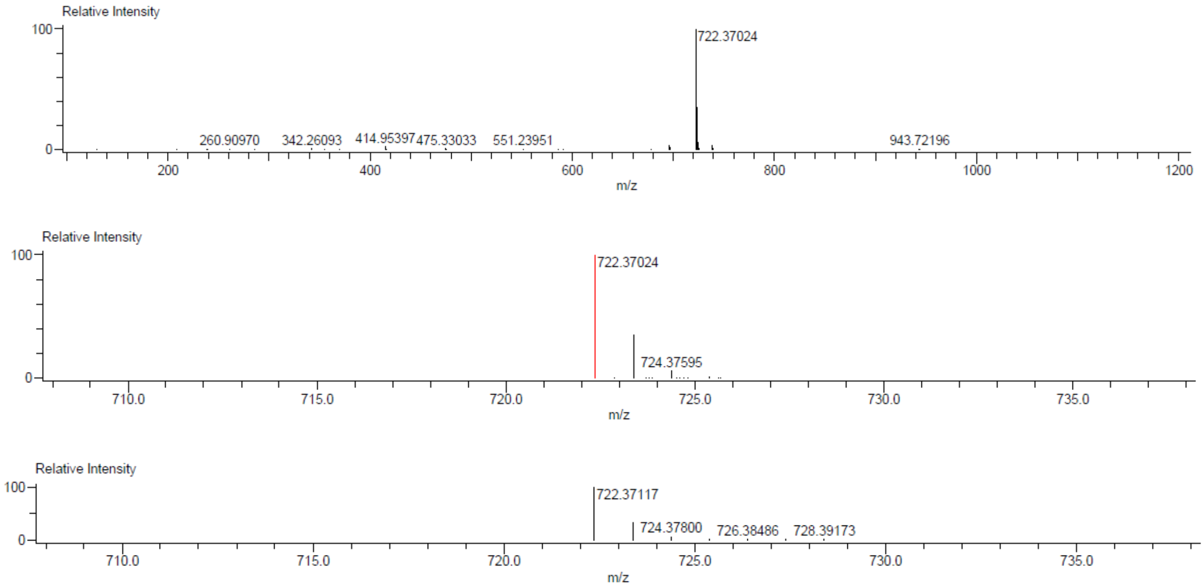

Mass	Intensity	Relative Intensity	Calc. Mass	Mass Difference [mDa]	Mass Difference [ppm]	Possible Formula	Unsaturation Number
722.37024	63805.06	100.00	722.37117	-0.92	-1.28	$^{12}C_{30}{}^{1}H_{60}{}^{209}Bi_{1}{}^{31}P_{3}$	6.0

Figure S53. LIFDI-MS (HR) spectrum of [Bi(P(*t*Bu)Cy)$_3$] (**2**) in benzene. Top: full spectrum. Middle: high resolution extract. Bottom: calculated isotope pattern.

Bi(P*t*Bu$_2$)$_3$ (3)

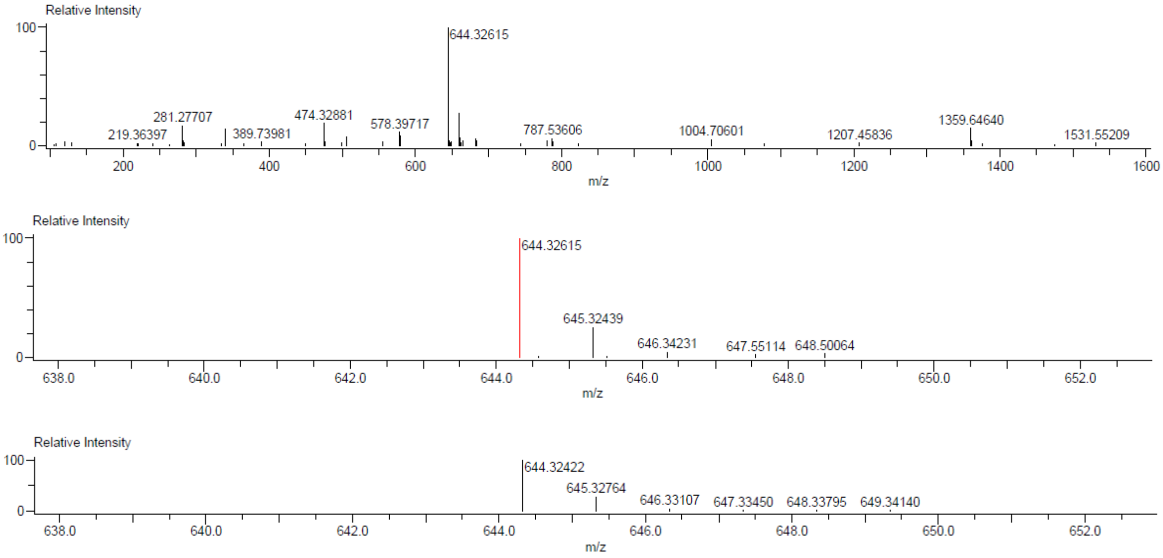

Mass	Intensity	Relative Intensity	Calc. Mass	Mass Difference [mDa]	Mass Difference [ppm]	Possible Formula	Unsaturation Number
644.32615	19625.08	100.00	644.32422	1.93	3.00	$^{12}C_{24}{}^{1}H_{54}{}^{209}Bi_{1}{}^{31}P_{3}$	3.0

Figure S54. LIFDI-MS (HR) spectrum of [Bi(P*t*Bu$_2$)$_3$] (**3**) in benzene. Top: Whole spectrum. Middle: High resolution extract. Bottom: Calculated isotope pattern.

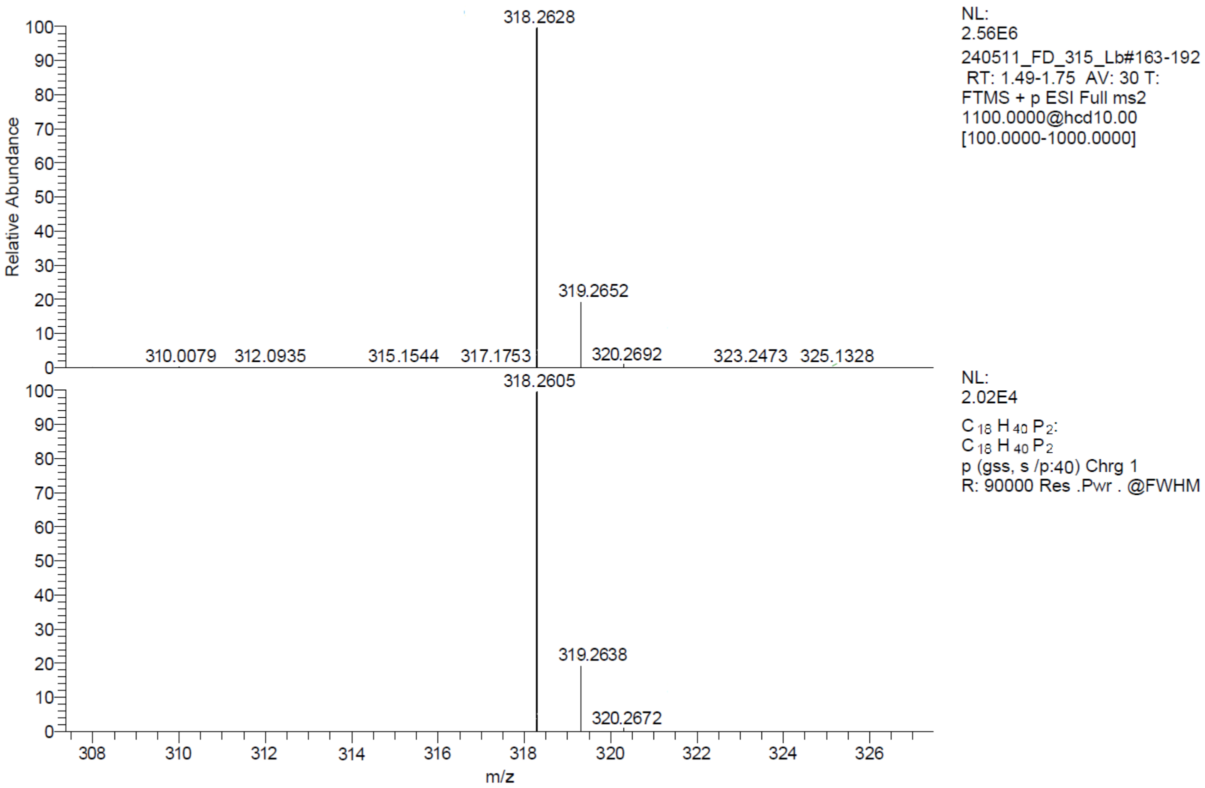

Figure S55. ESI(+)-MS (HR) spectrum of **8a** in THF. Top: High resolution spectrum of the signal at 318 m/z. Bottom: Calculated isotope pattern for $[P_2C_{18}H_{40}]^+$.

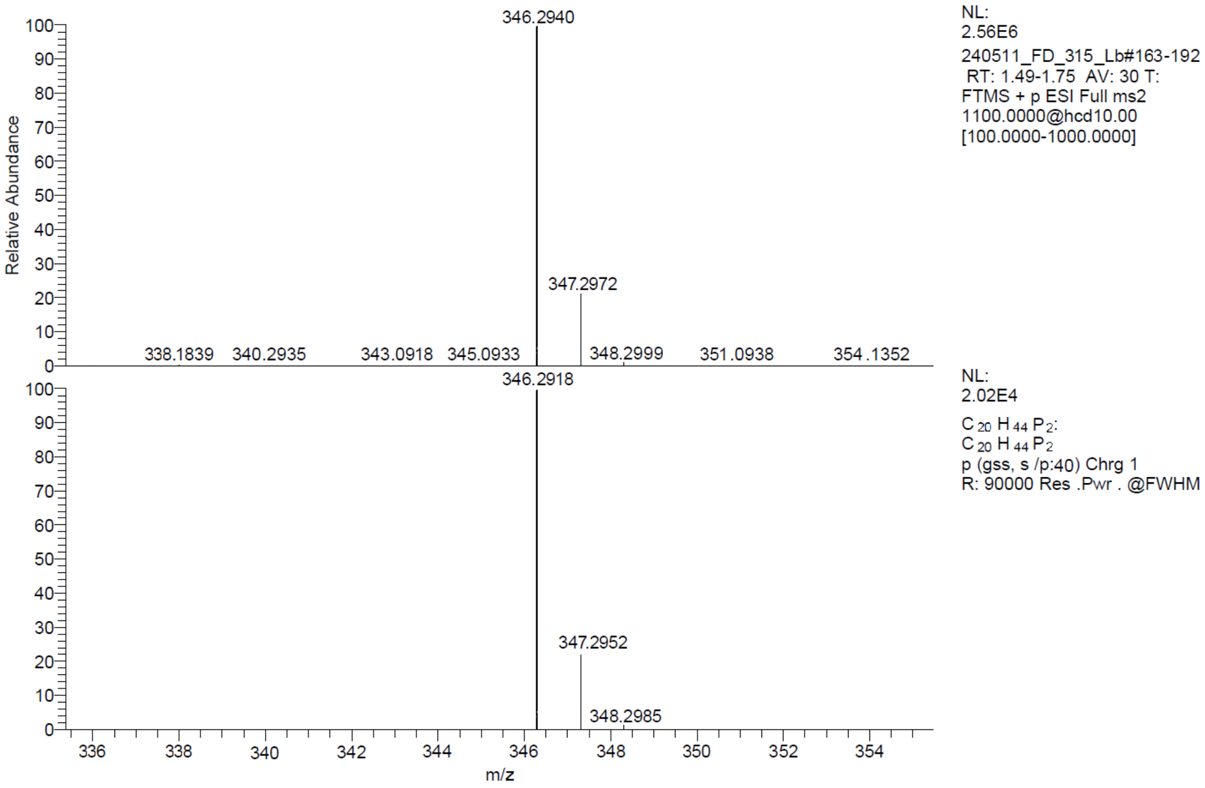

Figure S56. ESI(+)-MS (HR) spectrum of **8b** in THF. Top: High resolution spectrum of the signal at 346 m/z. Bottom: Calculated isotope pattern for $[P_2C_{20}H_{44}]^+$.

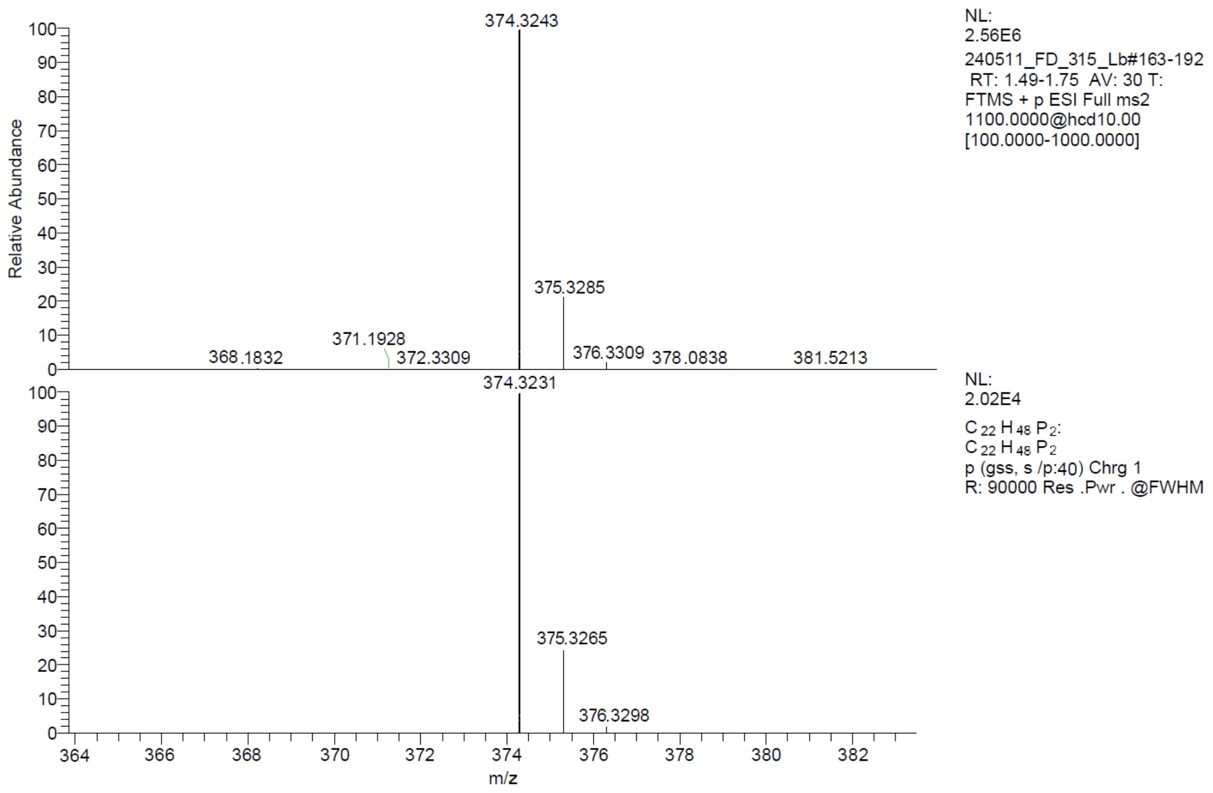

Figure S57. ESI(+)-MS (HR) spectrum of **8c** in THF. Top: High resolution spectrum of the signal at 374 m/z. Bottom: Calculated isotope pattern for $[P_2C_{22}H_{48}]^+$.

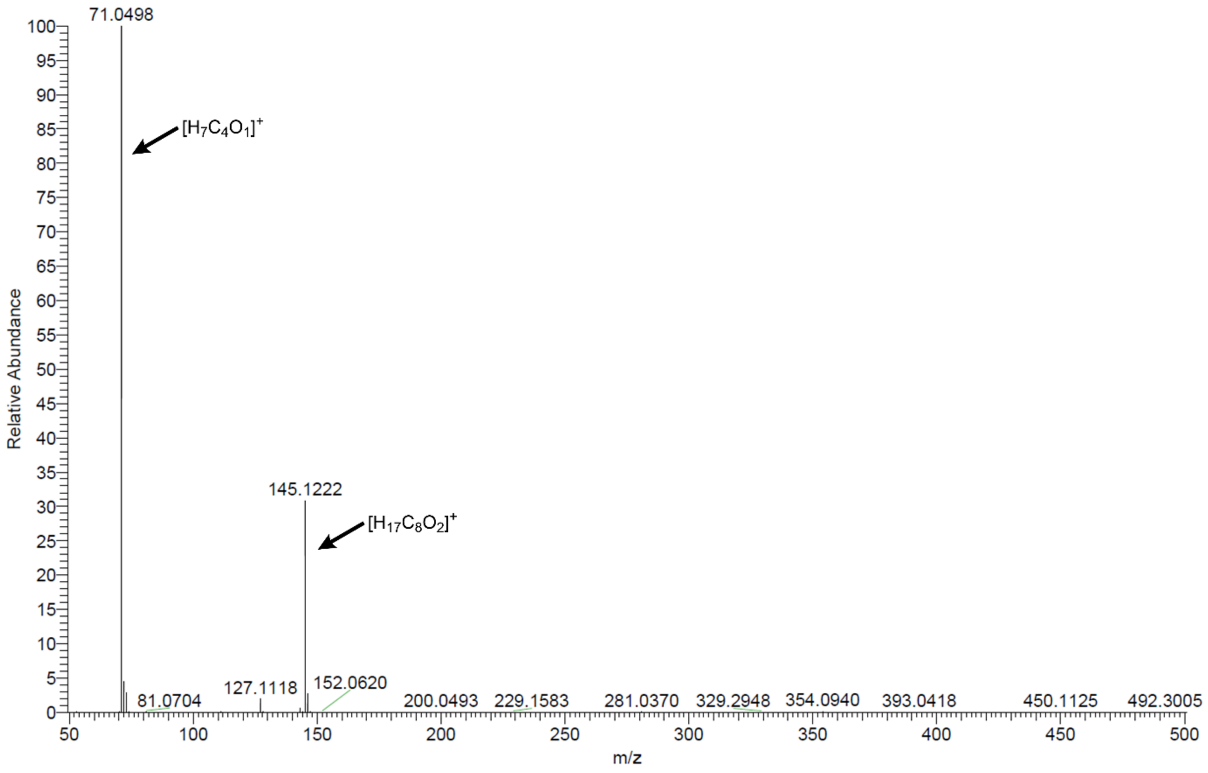

Figure S58. ESI(+)-MS (HR) spectrum of **9-CF₃** in THF in the area of 50–500 m/z.

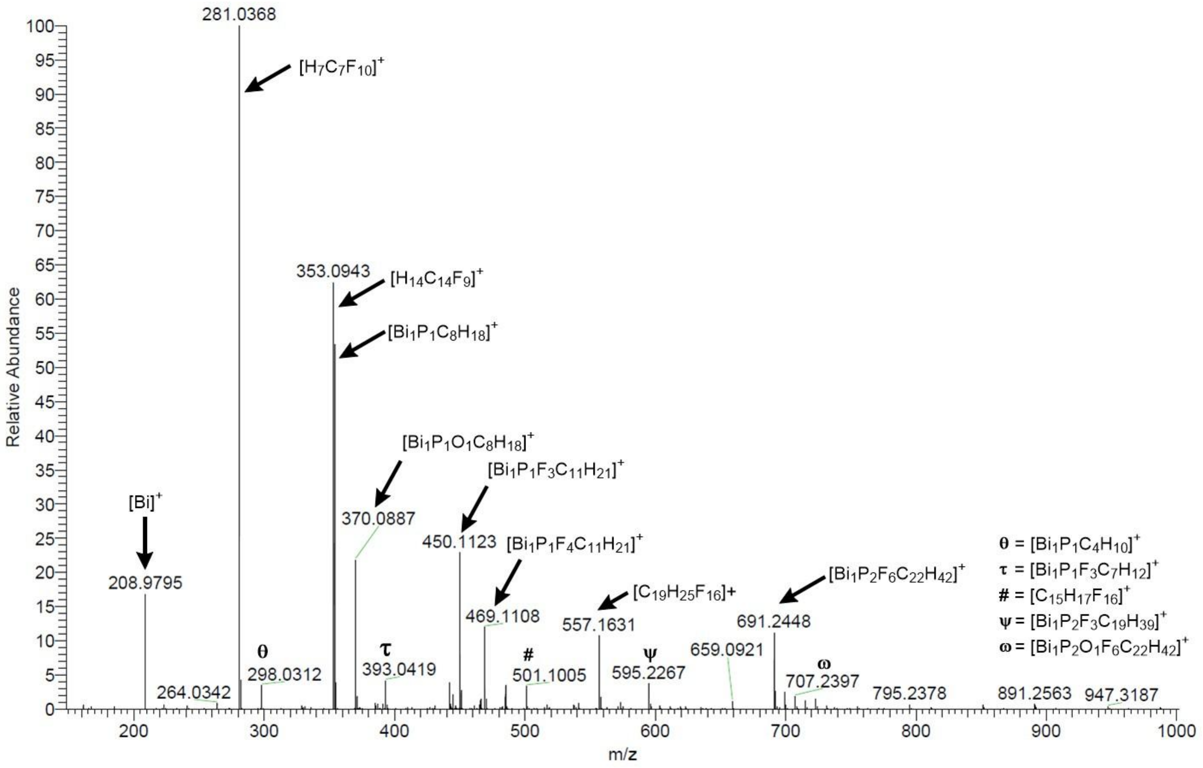

Figure S59. ESI(+)-MS (HR) spectrum of **9-CF3** in THF in the area of 100–1000 m/z.

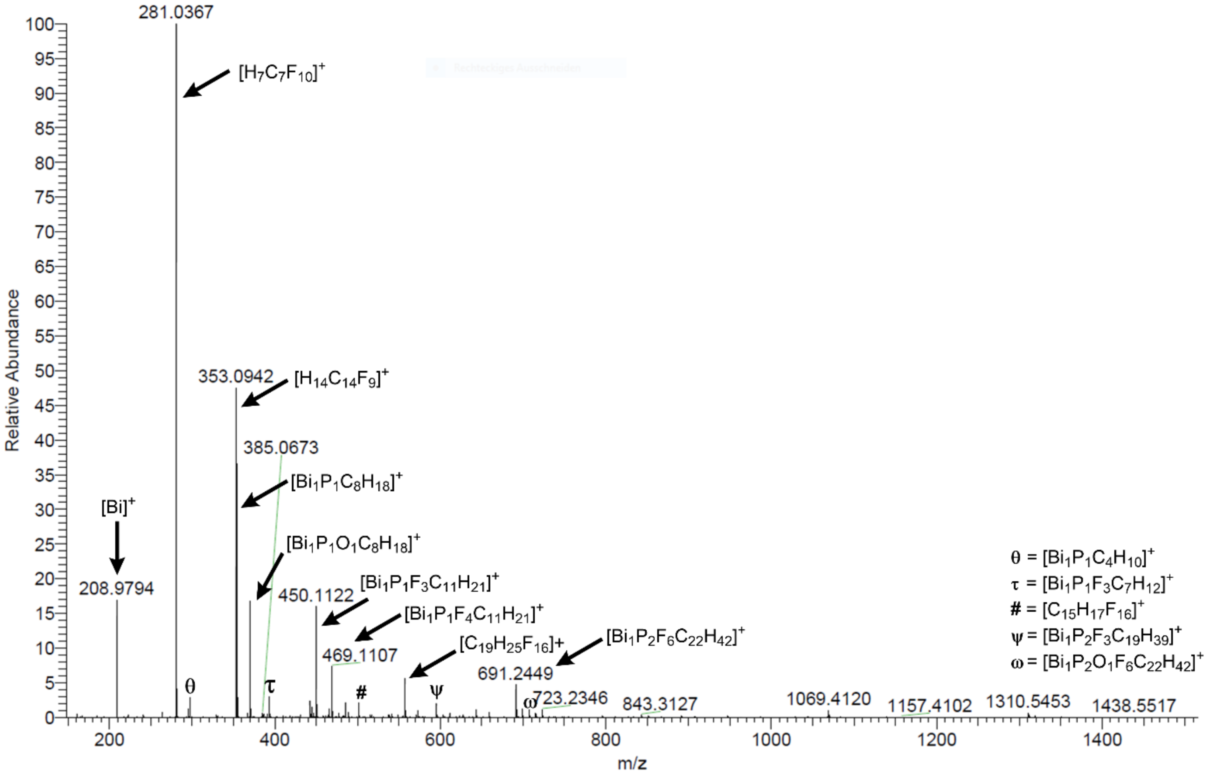

Figure S60. ESI(+)-MS (HR) spectrum of **9-CF3** in THF in the area of 150–1500 m/z.

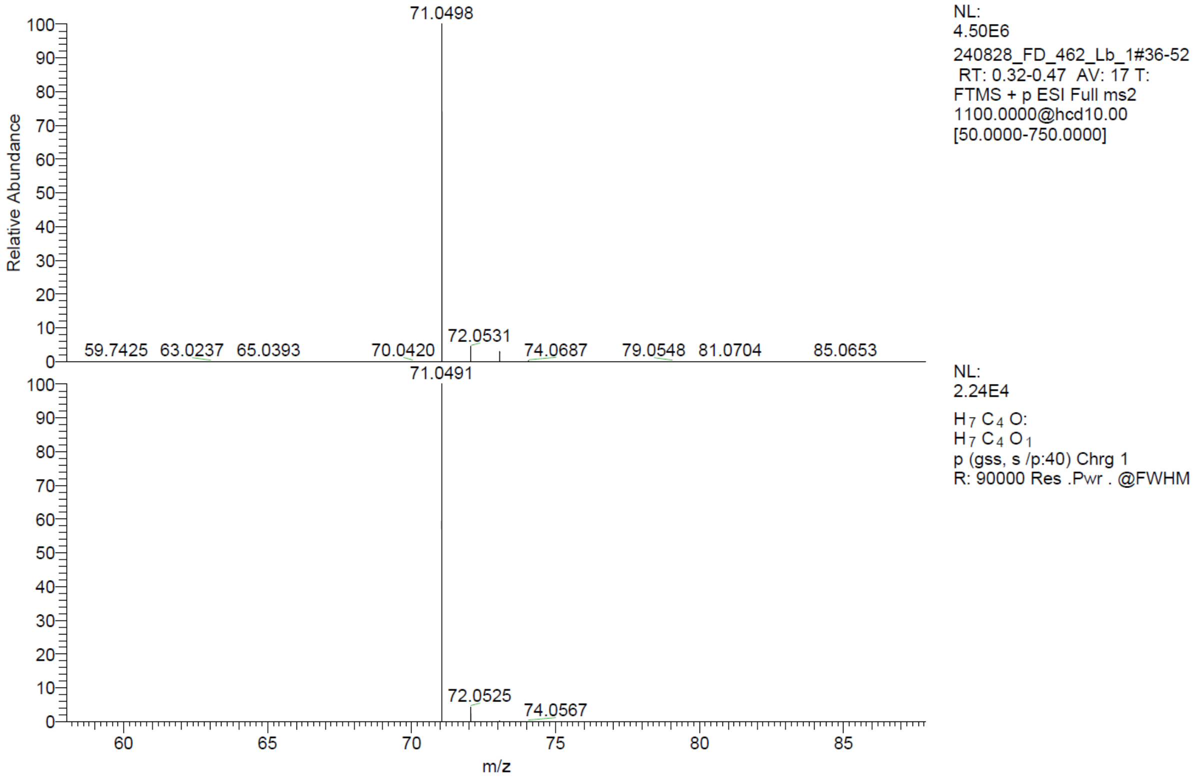

Figure S61. ESI(+)-MS (HR) spectrum of **9-CF$_3$** in THF. Top: High resolution spectrum of the Signal at 71 m/z. Bottom: Calculated isotope pattern for $[O_1C_4H_7]^+$.

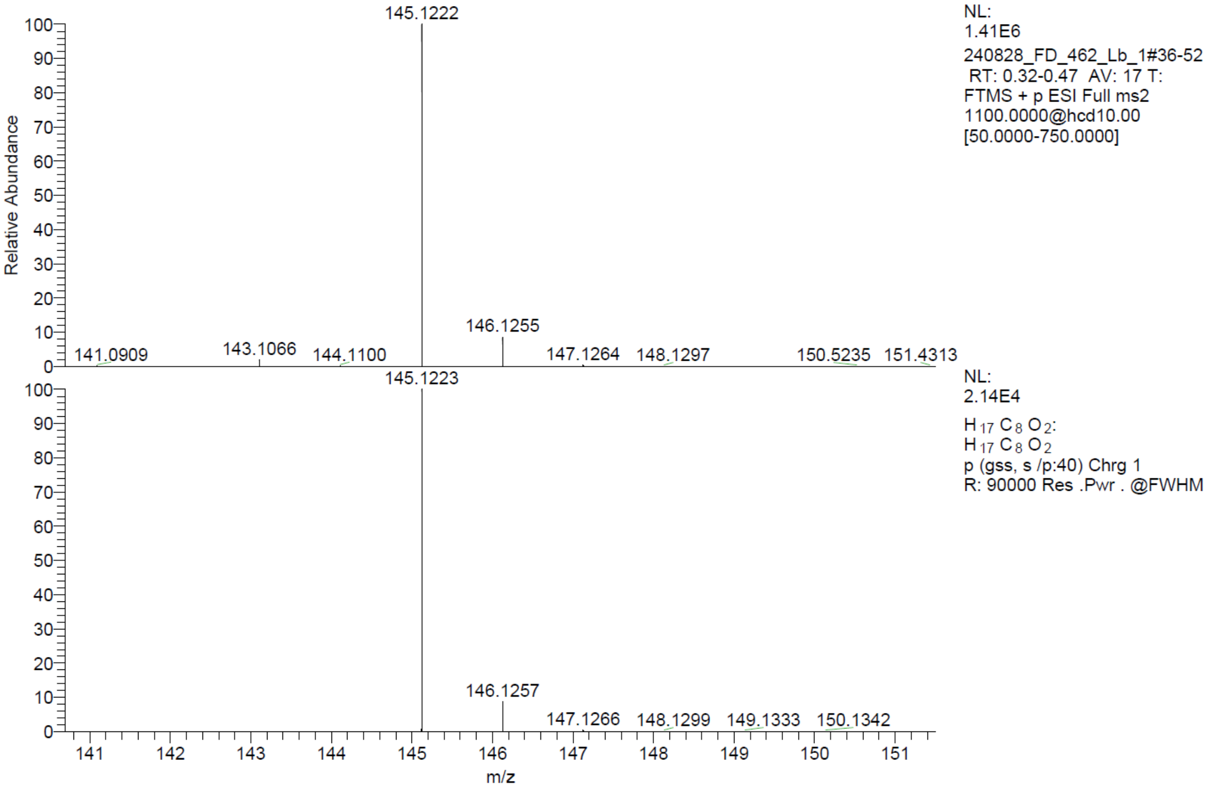

Figure S62. ESI(+)-MS (HR) spectrum of **9-CF$_3$** in THF. Top: High resolution spectrum of the Signal at 145 m/z. Bottom: Calculated isotope pattern for $[O_2C_8H_{17}]^+$.

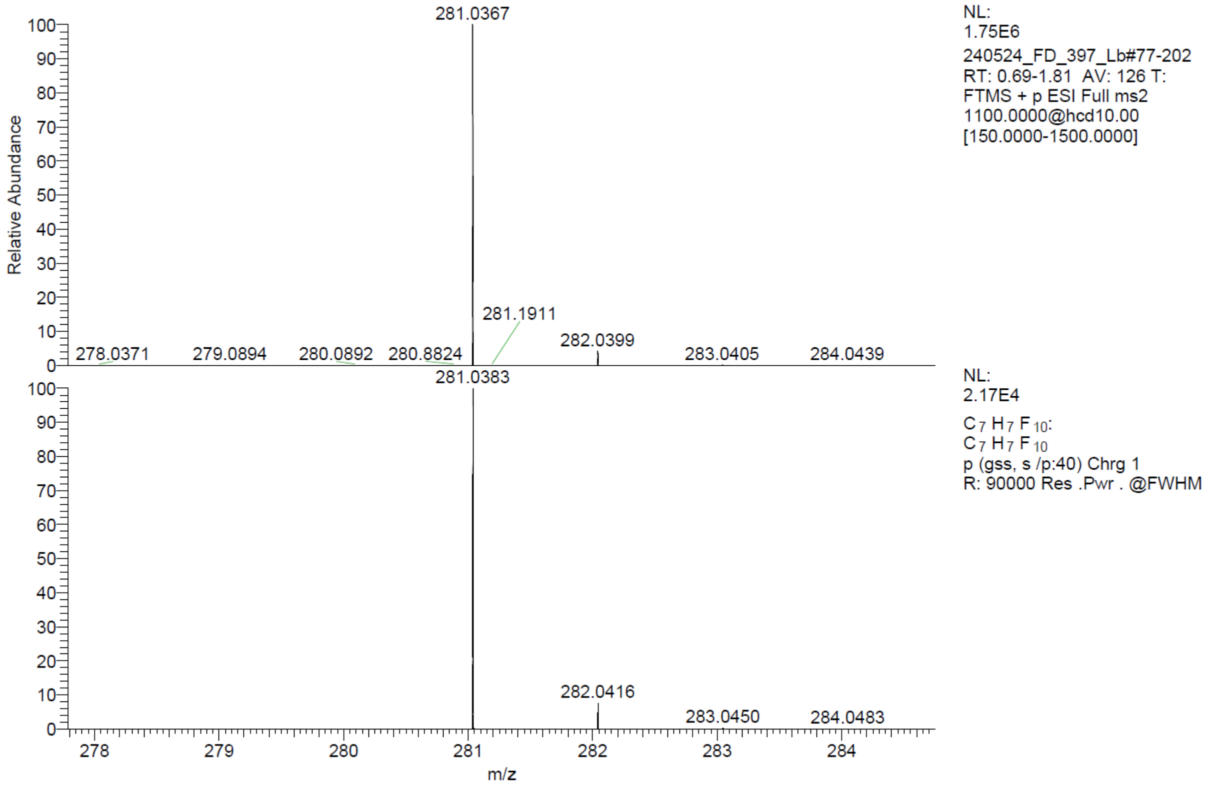

Figure S63. ESI(+)-MS (HR) spectrum of **9-CF$_3$** in THF. Top: High resolution spectrum of the Signal at 281 m/z. Bottom: Calculated isotope pattern for $[F_{10}C_7H_7]^+$.

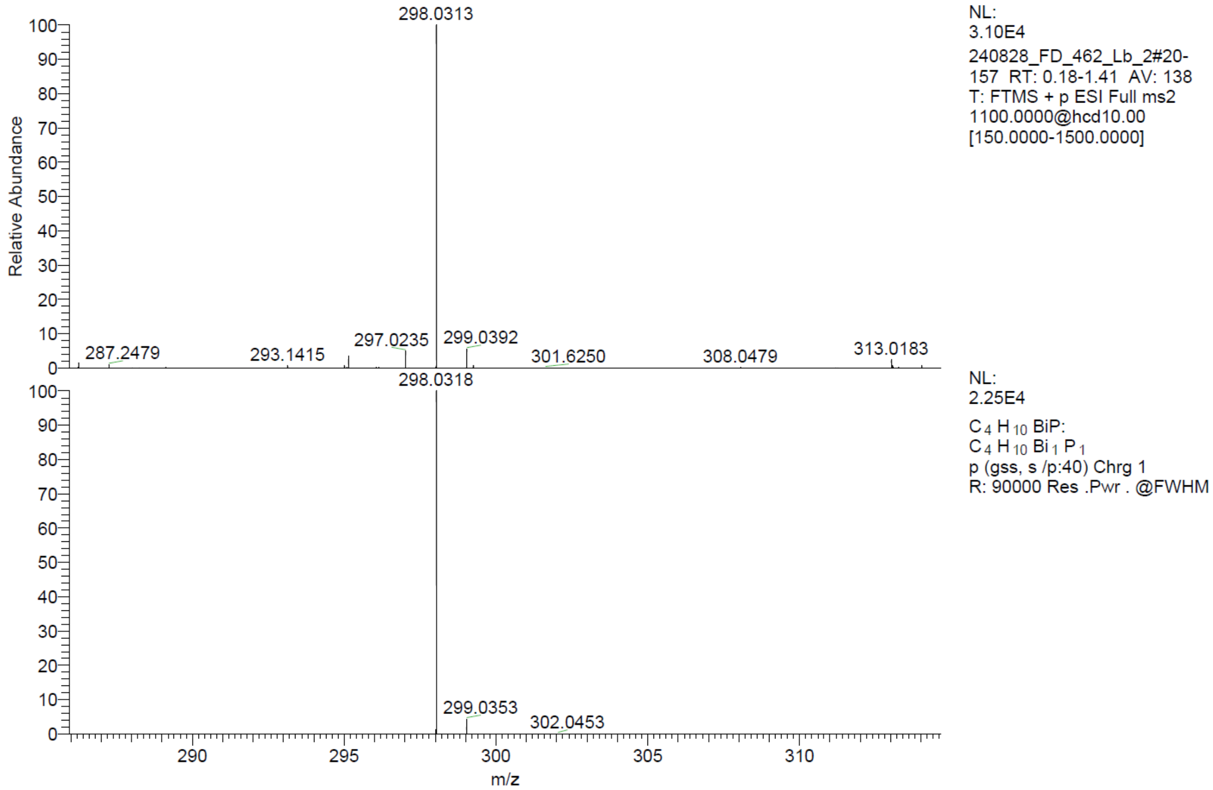

Figure S64. ESI(+)-MS (HR) spectrum of **9-CF$_3$** in THF. Top: High resolution spectrum of the Signal at 298 m/z. Bottom: Calculated isotope pattern for $[Bi_1P_1C_4H_{10}]^+$.

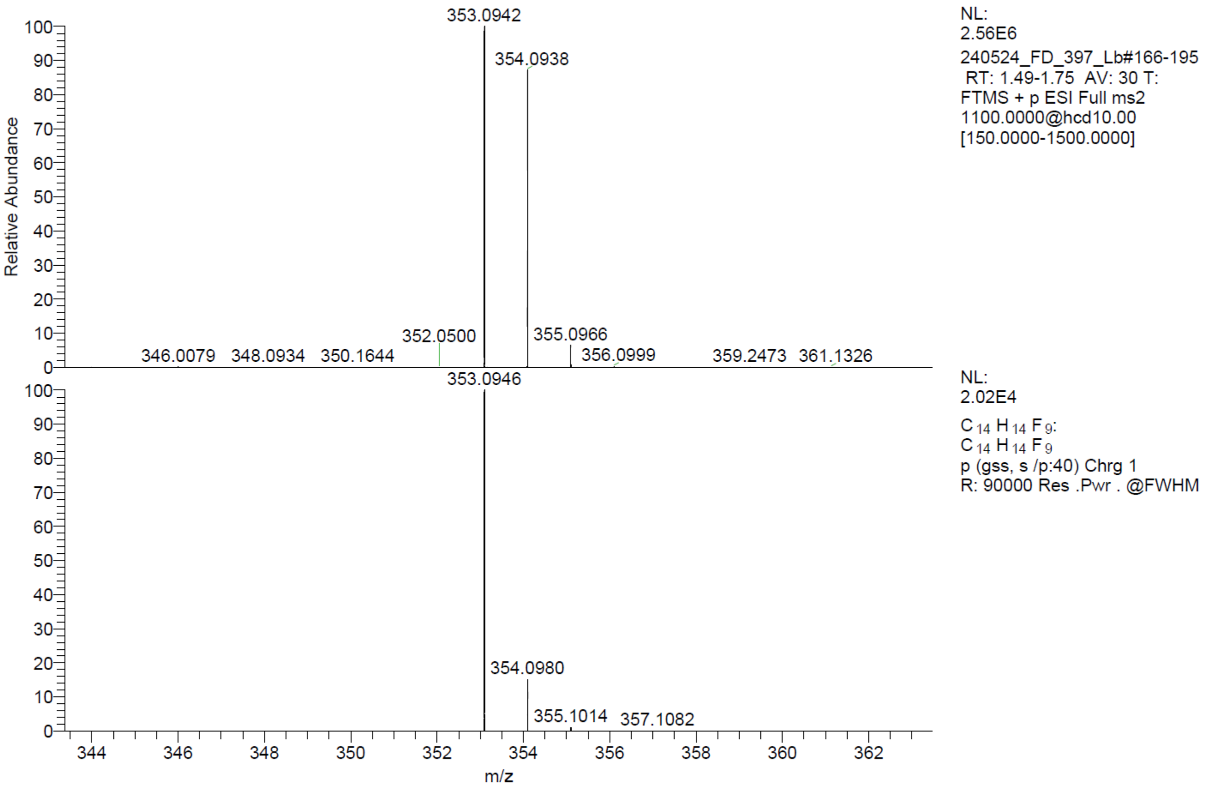

Figure S65. ESI(+)-MS (HR) spectrum of **9-CF$_3$** in THF. Top: High resolution spectrum of the Signal at 353 m/z. Bottom: Calculated isotope pattern for $[F_9C_{14}H_{14}]^+$.

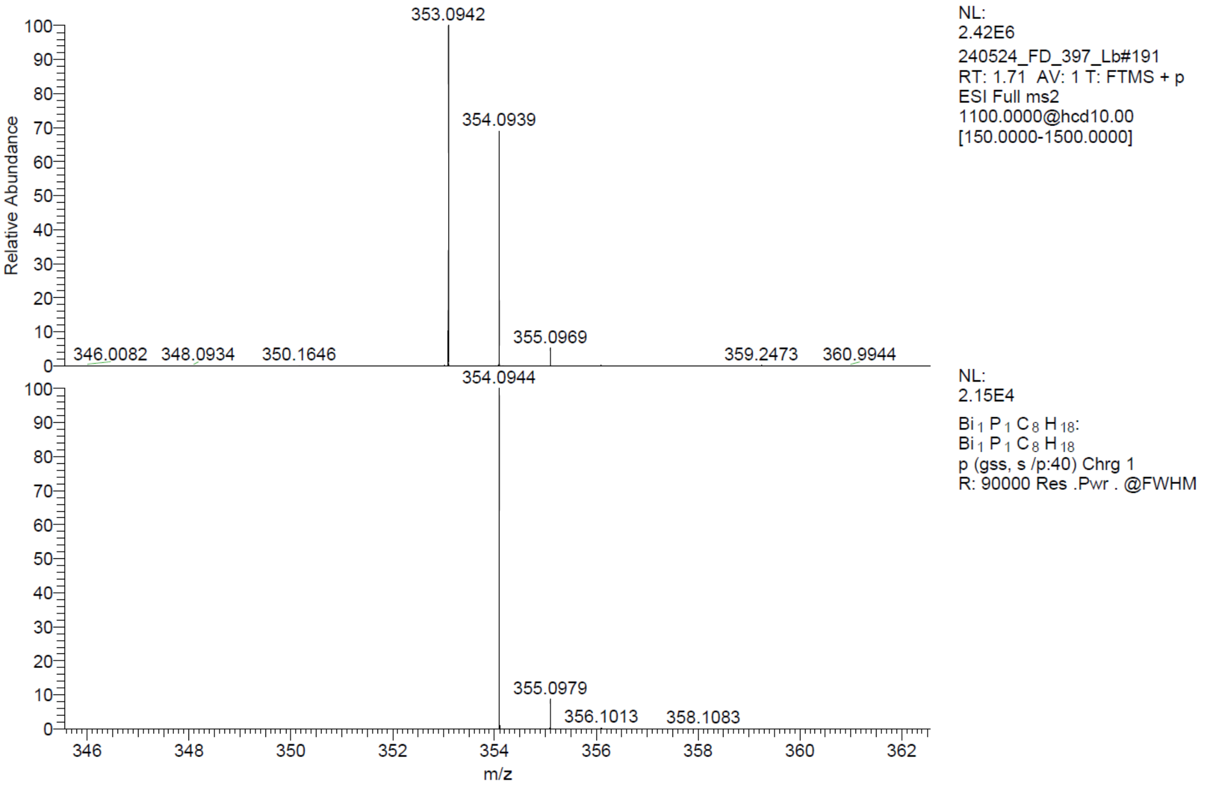

Figure S66. ESI(+)-MS (HR) spectrum of **9-CF$_3$** in THF. Top: High resolution spectrum of the Signal at 354 m/z. Bottom: Calculated isotope pattern for $[Bi_1P_1C_8H_{18}]^+$.

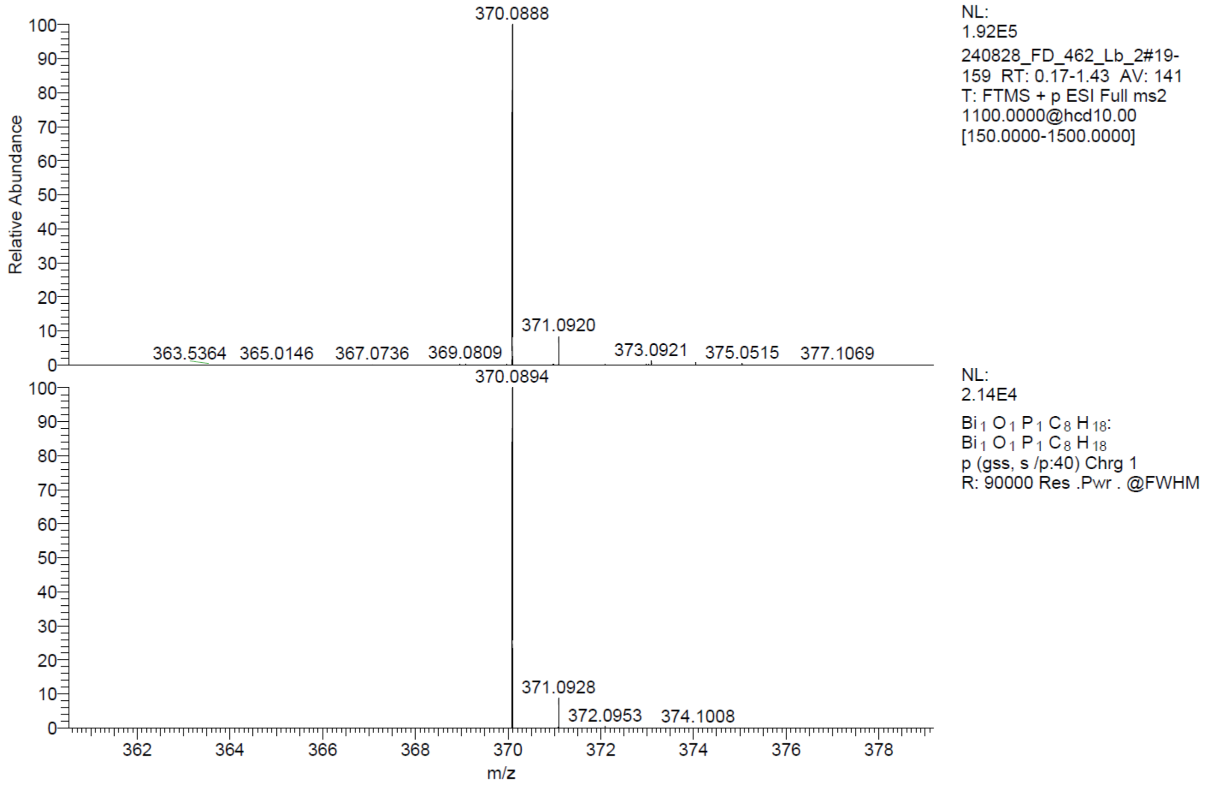

Figure S67. ESI(+)-MS (HR) spectrum of **9-CF$_3$** in THF. Top: High resolution spectrum of the Signal at 370 m/z. Bottom: Calculated isotope pattern for $[Bi_1P_1O_1C_8H_{18}]^+$.

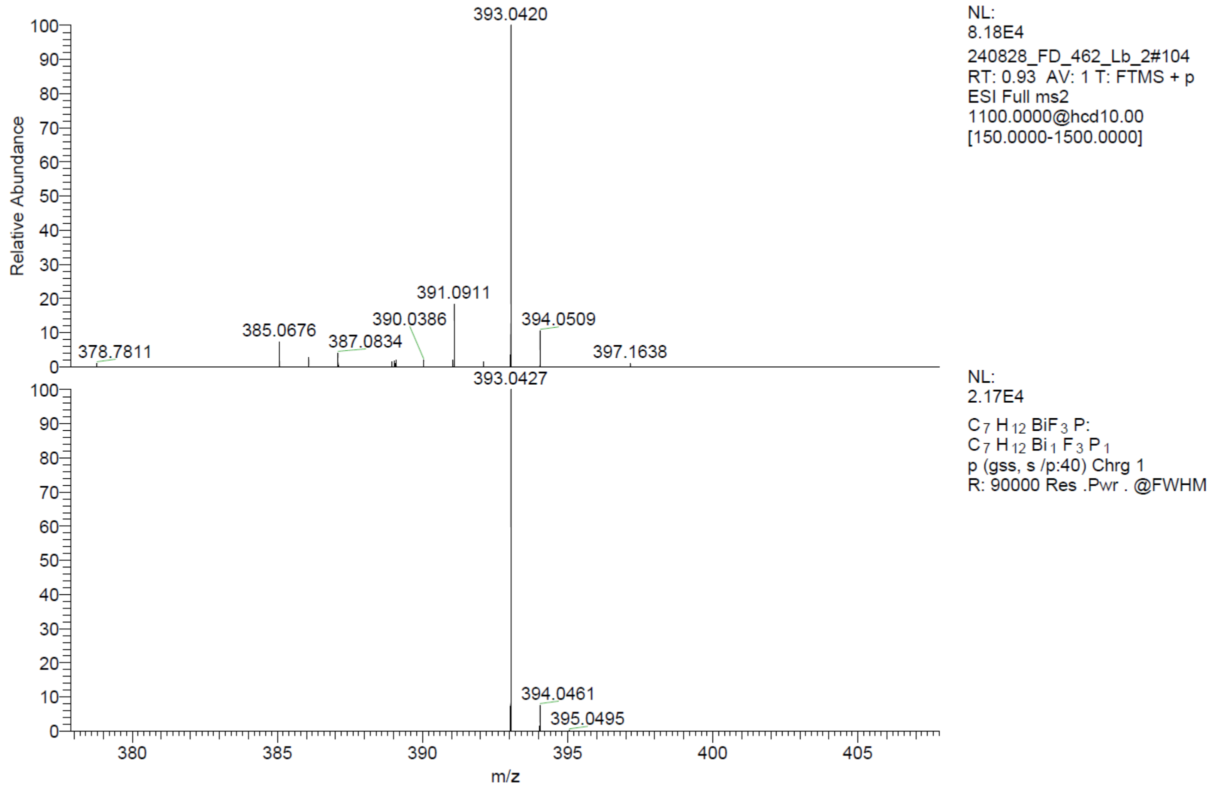

Figure S68. ESI(+)-MS (HR) spectrum of **9-CF$_3$** in THF. Top: High resolution spectrum of the Signal at 393 m/z. Bottom: Calculated isotope pattern for $[Bi_1P_1F_3C_7H_{12}]^+$.

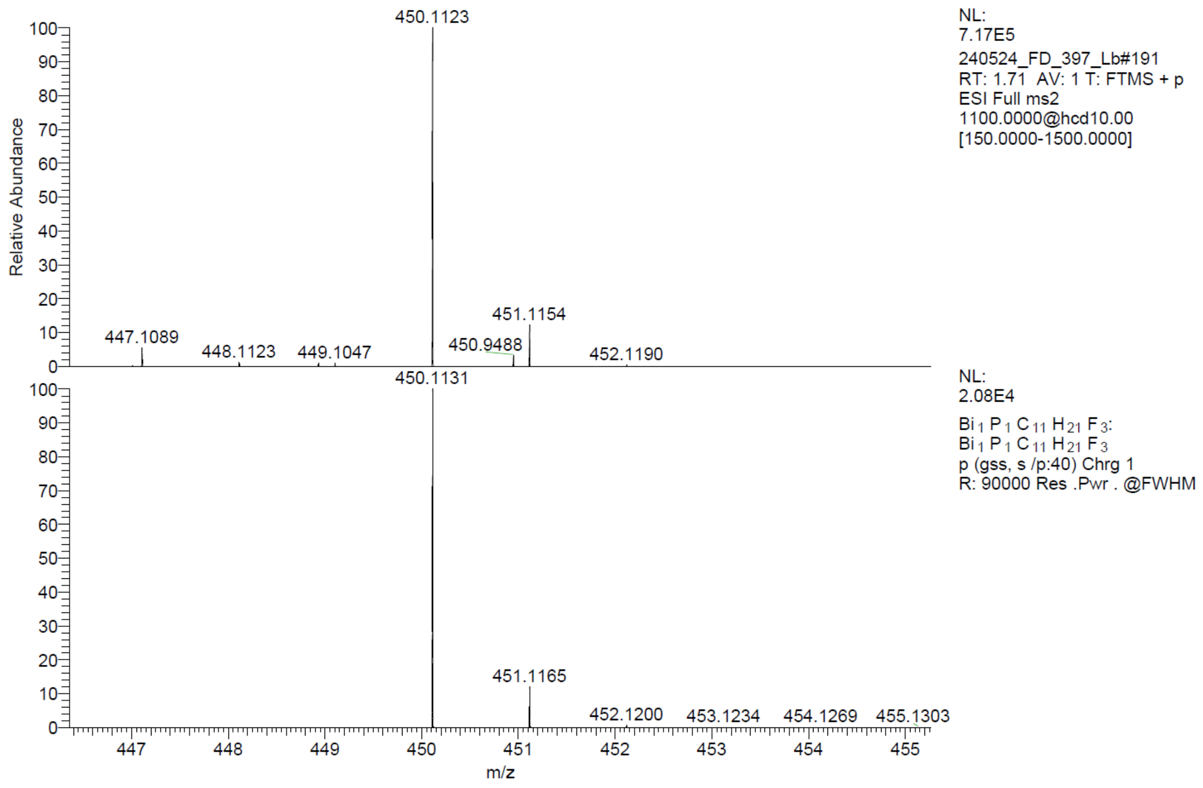

Figure S69. ESI(+)-MS (HR) spectrum of **9-CF₃** in THF. Top: High resolution spectrum of the Signal at 450 m/z. Bottom: Calculated isotope pattern for $[Bi_1P_1F_3C_{11}H_{21}]^+$.

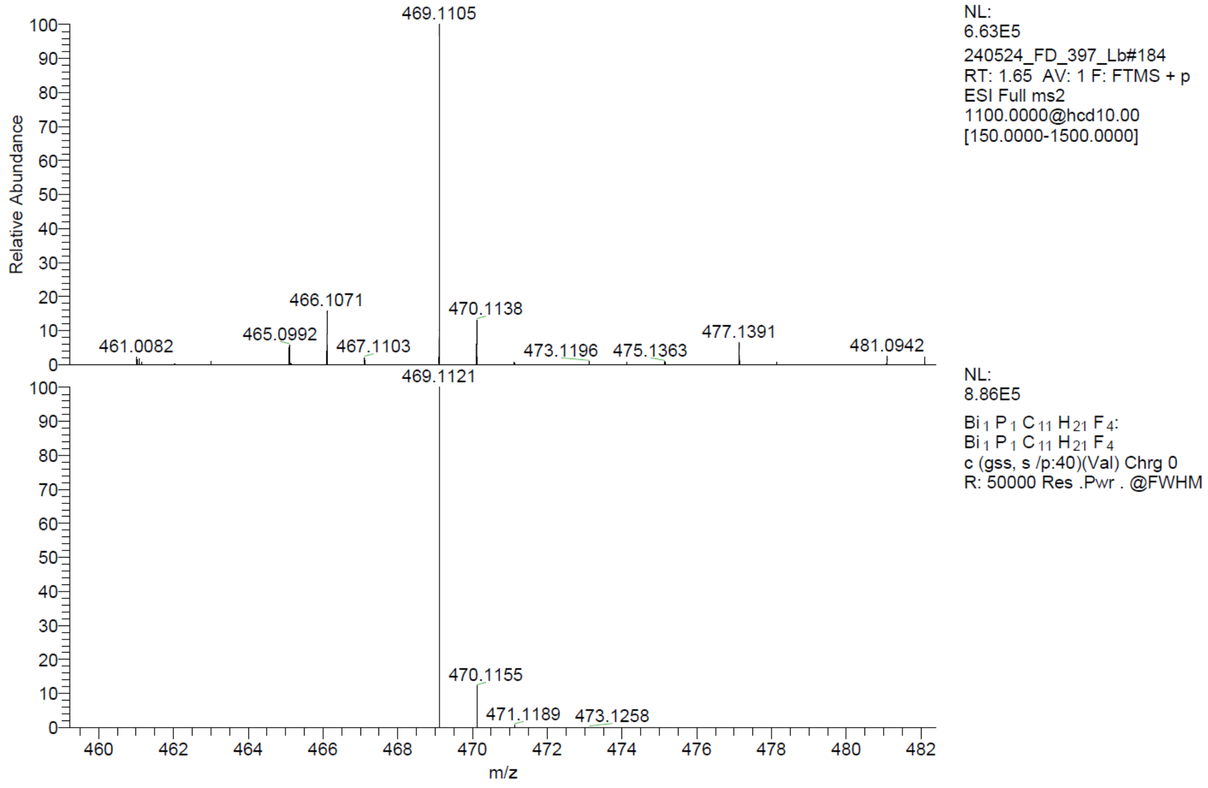

Figure S70. ESI(+)-MS (HR) spectrum of **9-CF₃** in THF. Top: High resolution spectrum of the Signal at 469 m/z. Bottom: Calculated isotope pattern for $[Bi_1P_1F_4C_{11}H_{21}]^+$.

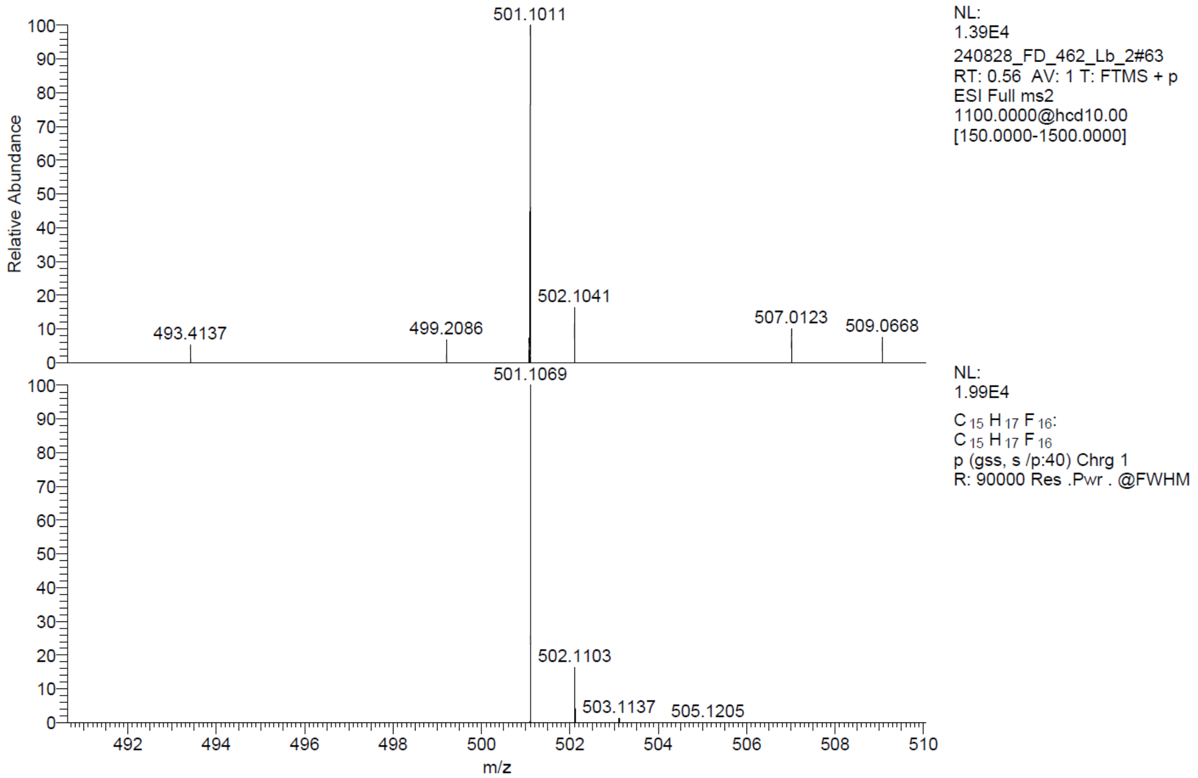

Figure S71. ESI(+)-MS (HR) spectrum of **9-CF$_3$** in THF. Top: High resolution spectrum of the Signal at 501 m/z. Bottom: Calculated isotope pattern for $[F_{16}C_{15}H_{17}]^+$.

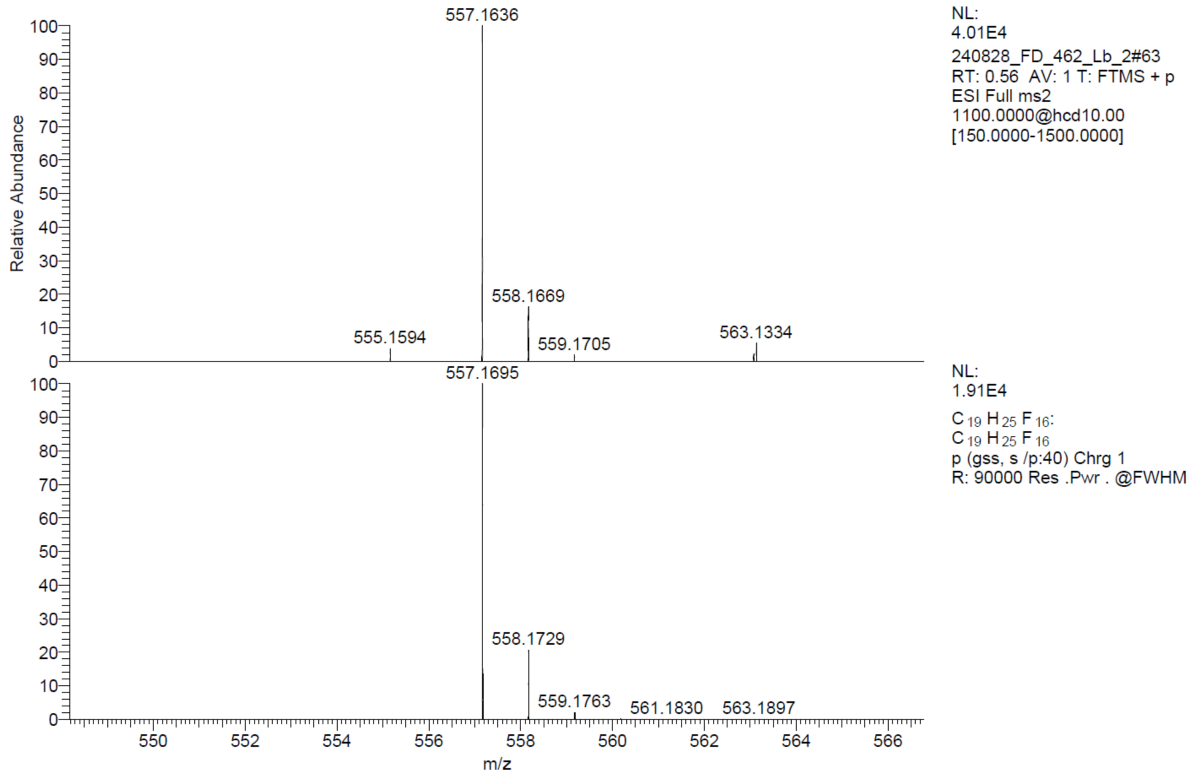

Figure S72. ESI(+)-MS (HR) spectrum of **9-CF$_3$** in THF. Top: High resolution spectrum of the Signal at 557 m/z. Bottom: Calculated isotope pattern for $[F_{16}C_{19}H_{25}]^+$.

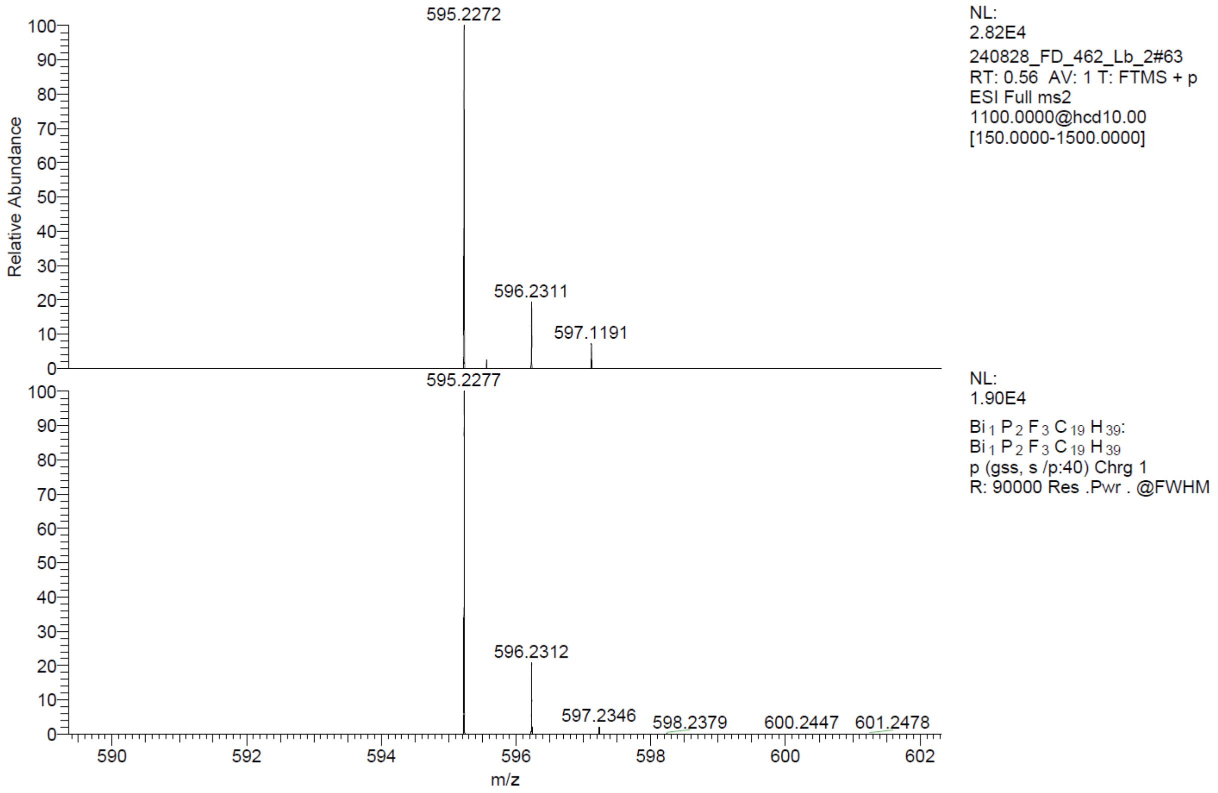

Figure S73. ESI(+)-MS (HR) spectrum of **9-CF$_3$** in THF. Top: High resolution spectrum of the Signal at 595 m/z. Bottom: Calculated isotope pattern for $[Bi_1P_2F_3C_{19}H_{39}]^+$.

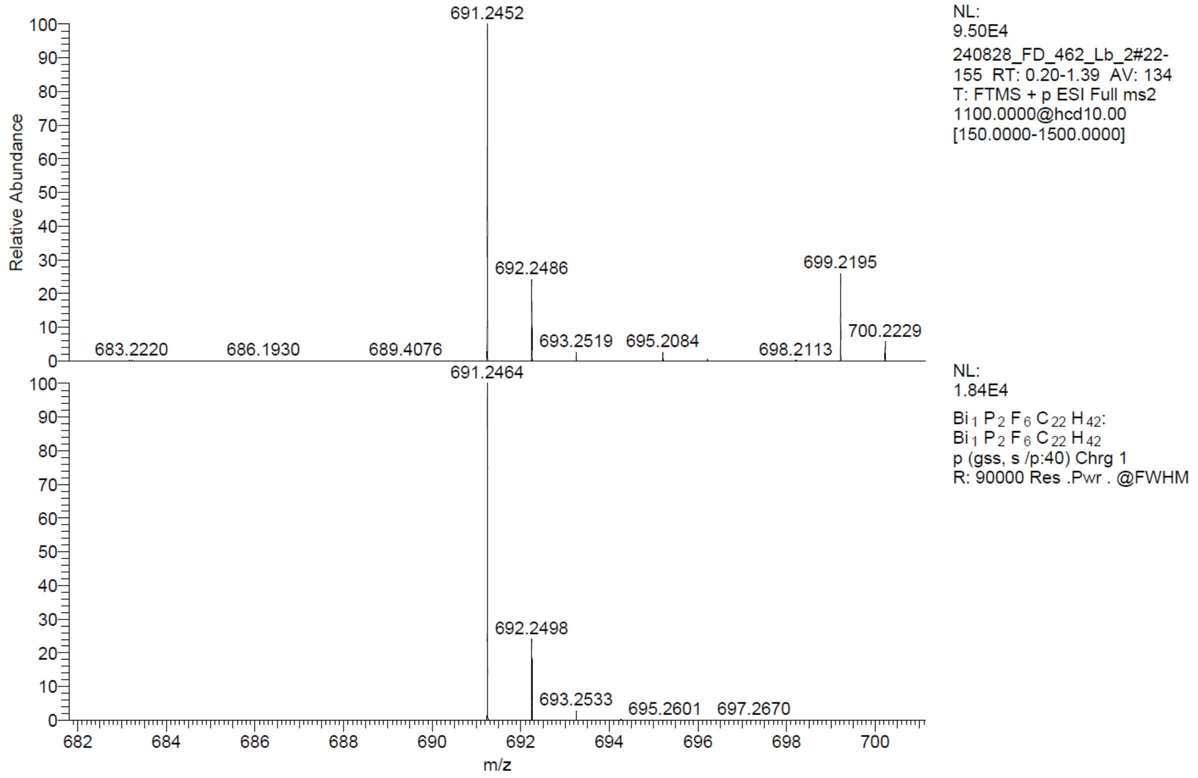

Figure S74. ESI(+)-MS (HR) spectrum of **9-CF$_3$** in THF. Top: High resolution spectrum of the Signal at 691 m/z. Bottom: Calculated isotope pattern for $[Bi_1P_2F_6C_{22}H_{42}]^+$.

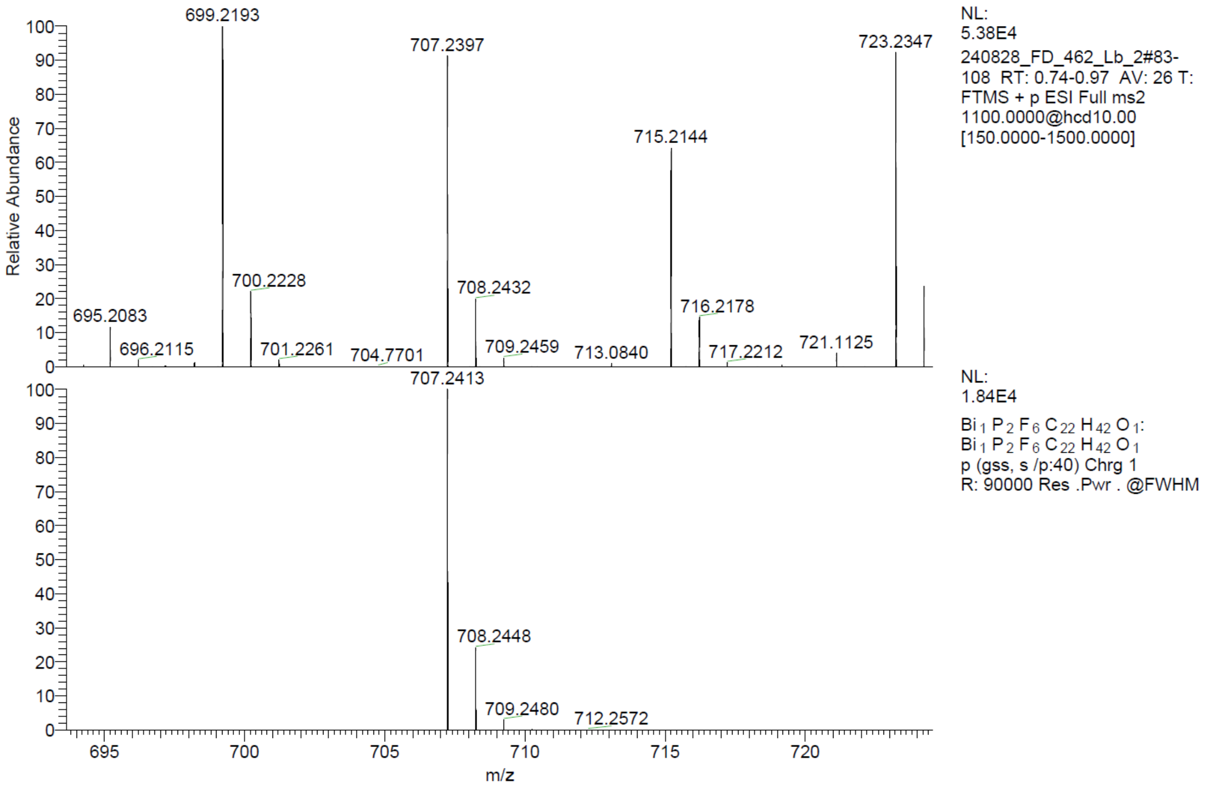

Figure S75. ESI(+)-MS (HR) spectrum of **9-CF$_3$** in THF. Top: High resolution spectrum of the Signal at 707 m/z. Bottom: Calculated isotope pattern for $[Bi_1P_2F_6O_1C_{22}H_{42}]^+$.

Cyclic voltammetry

Cyclic voltammograms of **2** and **3** were recorded using a *Gamry Interface 1010 potentiostat*. A standard three-electrode cell configuration was employed using a platinum disk working electrode, a platinum wire counter electrode, and a silver wire, separated by a *Vycor®* tip, serving as the reference electrode. Concentrations of 1 mM of analyte and 0.1 M nBu$_4$N[PF$_6$], which acted as electrolyte, were used in the default measurement setup. The [FeCp$_2$] / [FeCp$_2$]$^+$ (Fc/Fc$^+$) redox couple was utilized as an internal standard. The measurements were performed with a scan rate of 100 mV/s, with two full cycles. Peak potentials and currents of the second cycle of each measurement were determined using the *Gamry Framework^{TM} Data Acquisition* Software (ver. 7.9.0).

The cyclic voltammogram of **2** shows two reduction waves at potentials of −1.24 V and −2.85 V and two oxidation waves at −1.75 V and 0.20 V. The cyclic voltammogram is closely related to that of **3** (vide infra), which can be attributed to the high similarity of these compounds.

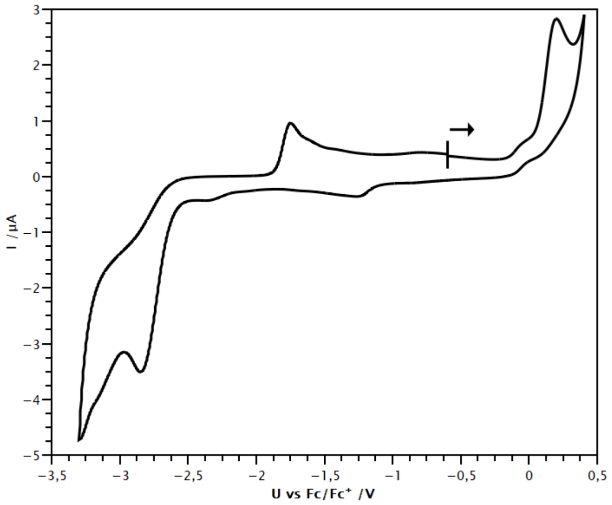

Figure S76. Cyclic voltammogram of **2** in THF at a scan rate of 100 mV/s (0.1 M nBu$_4$N[PF$_6$] vs Fc/Fc$^+$).

The cyclic voltammogram of **3** shows a rather complex pathway. A reduction at a potential at −2.66 V and three oxidations at −1.73 V, −0.91 V and 0.20 V were observed. All detected redox processes show no reversibility.

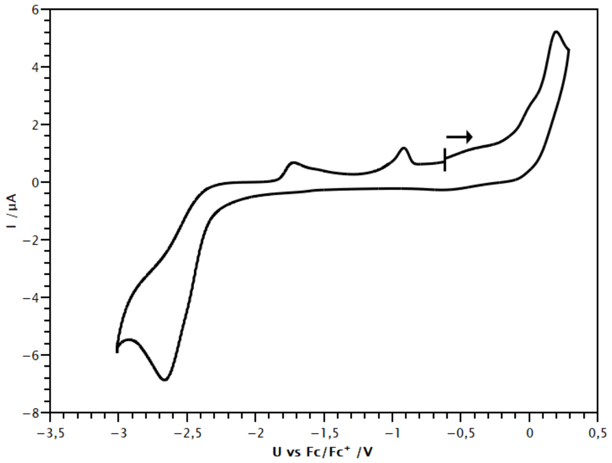

Figure S77. Cyclic voltammogram of **3** in THF at a scan rate of 100 mV/s (0.1 M nBu$_4$N[PF$_6$] vs Fc/Fc$^+$).

Electron-paramagnetic-resonance spectroscopy (EPR)

Detecting free radicals

X-band EPR spectroscopic measurements were carried out at a given temperature, using a Bruker ELEXSYS E580 CW/FT EPR spectrometer. The spectral simulations were performed using MATLAB 9.6 (2019a) and the EasySpin 5.2.25 toolbox.[29]

To observe the free phosphanyl radicals, compounds **2** and **3** were dissolved in toluene. The samples were inserted into the cavity of the spectrometer. The temperature in the cavity had been pre-set to 95 °C (for **2**) or 70 °C (for **3**), and the measurement was immediately started. After eight (in the case of **2**) or two (in the case of **3**) accumulative scans, the tuning was lost, presumably due to deposition of a dark precipitate at the inner glass wall of the EPR tube. Baseline drifts are ascribed to inconstant heat dissipation.

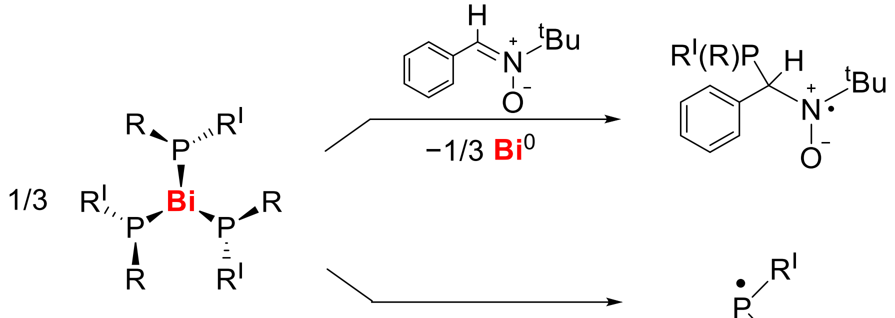

Scheme S7. Bi–P bond homolysis in **2** and **3**, followed by trapping of phosphanyl radicals with PBN (top) or the detection of the free phosphanyl radicals (bottom).

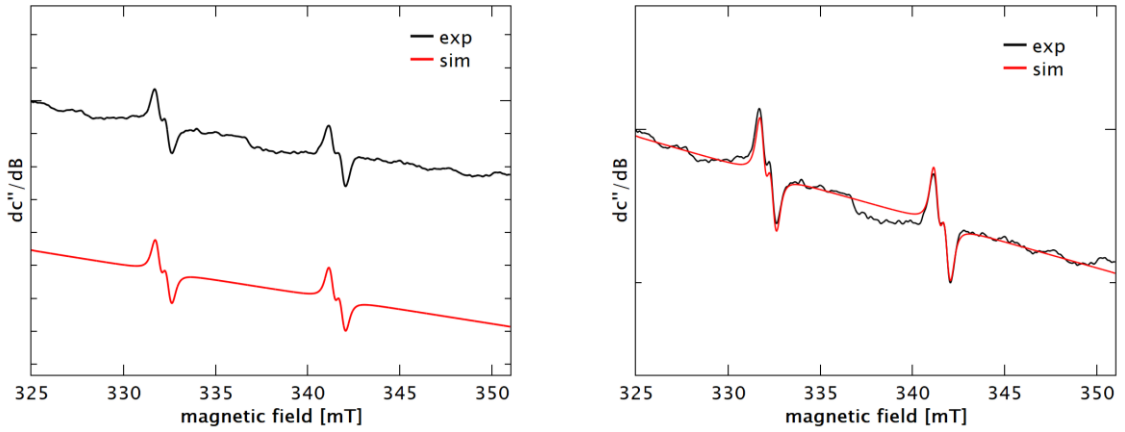

Figure S78. Experimental (black) and simulated (red) continuous-wave (CW) X-band EPR spectra of a solution containing **2** (c = 0.121 mol/L) in toluene. The observed resonance shows coupling constants of a(^{31}P) = 264.2 MHz (94.1 G, 9.41 mT), a(^{1}H) = 14.1 MHz (5.01 G, 0.501 mT), and a g_{iso} value of 2.0062. Spectrometer settings: microwave frequency = 9.463517 GHz, 0.20 mT modulation amplitude at 100 kHz, microwave power = 50 mW, temperature = 95 °C, number of accumulated scans = 8, conversion time = 1 ms.

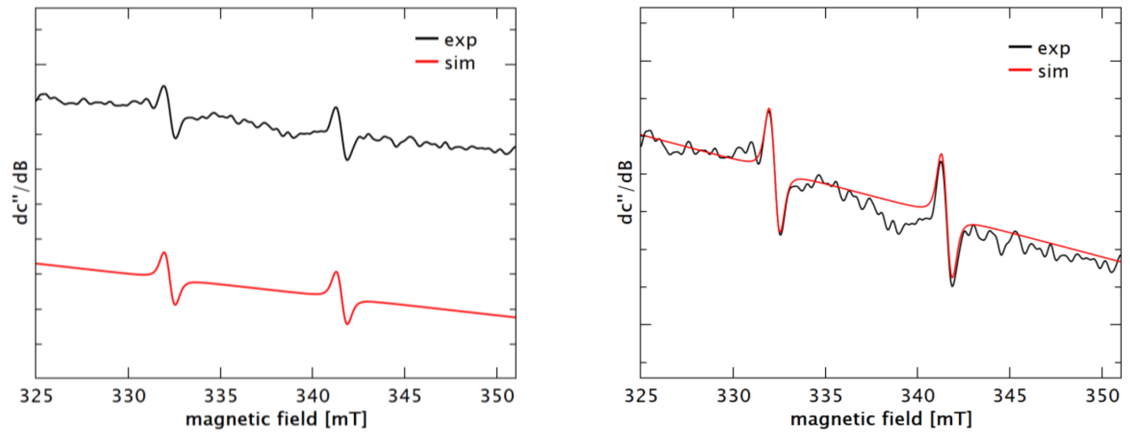

Figure S79. Experimental (black) and simulated (red) continuous-wave (CW) X-band EPR spectra of a solution containing **3** (c = 0.0132 mol/L) in toluene. The observed resonance shows a coupling constant of a(^{31}P) = 261.7 MHz (93.2 G, 9.32 mT) and a g_{iso} value of 2.0058. Spectrometer settings: microwave frequency = 9.463184 GHz, 0.20 mT modulation amplitude at 100 kHz, microwave power = 10 mW, temperature = 70 °C, number of accumulated scans = 2, conversion time = 1 ms.

The isolated diphosphanes **5** and **6** were EPR-silent under same conditions as noted above (in Figures S78 and S79) for compounds **2** and **3** or at slightly higher temperatures of 100 °C.

Detecting trapped radicals

In order to trap the free radicals, compounds **2** and **3** were mixed with 3 equiv. phenyl-*N*-*t*-butylnitrone (PBN) at room temperature in THF. The resulting radicals can be assigned to the trapped phosphanyl radicals and are in agreement with the trend that can be expected from two literature examples, see Table S4.[30,31]

Table S5: Comparison of the coupling constants of PBN coupled phosphanyl radicals.

Trapped radical	a(^{14}N) [mT]	a(^{31}P) [mT]	a(^{1}H) [mT]	Ref.
$(PPh_2)^•$	1.425	1.838	0.325	30
$(PCy_2)^•$	1.439	1.211	0.335	31
(P*t*BuCy)$^•$	1.47	1.03	0.330	This work
(P*t*Bu$_2$)$^•$	1.46	0.874	0.455	This work

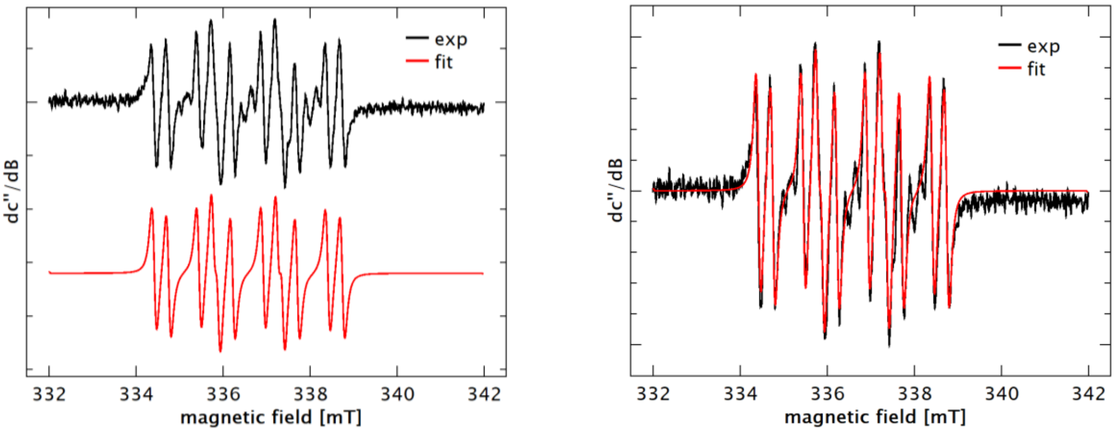

Figure S80. Experimental (black) and simulated (red) continuous-wave (CW) X-band EPR spectra of a solution containing 1 equiv. **2** (c = 0.0324 mol/L) and 3 equiv. PBN in THF (0.1 mL). The observed resonance shows coupling constants of a(^{14}N) = 41.4 MHz (14.7 G, 1.47 mT), a(^{31}P) = 28.9 MHz (10.3 G, 1.03 mT), a(^{1}H) = 9.27 MHz (3.30 G, 0.330 mT) and a g_{iso} value of 2.0053. Spectrometer settings: microwave frequency = 9.447741 GHz, 0.05 mT modulation amplitude at 100 kHz, microwave power = 10 mW, number of accumulated scans = 10, conversion time = 4 ms.

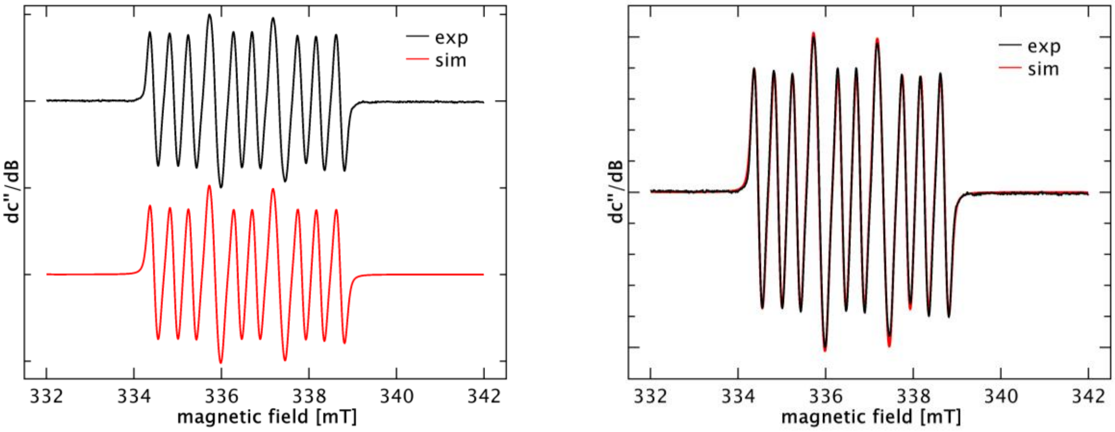

Figure S81. Experimental (black) and simulated (red) continuous-wave (CW) X-band EPR spectra of a solution containing 1 equiv. **3** (c = 0.0327 mol/L) and 3 equiv. PBN in THF (0.1 mL). The observed resonance shows coupling constants of a(^{14}N) = 40.9 MHz (14.6 G, 1.46 mT), a(^{31}P) = 24.5 MHz (8.74 G, 0.874 mT), a(^{1}H) = 12.8 MHz (4.55 G, 0.455 mT) and a g_{iso} value of 2.0052. Spectrometer settings: microwave frequency = 9.447749 GHz, 0.2 mT modulation amplitude at 100 kHz, microwave power = 10 mW, number of accumulated scans = 1, conversion time = 4 ms.

Computational Studies

Computational Details

All geometry optimizations and single point energy calculations were performed using the Gaussian16[32] suite of programs at the B3LYP+GD3/def2-TZVP$_{(Benzene,SMD)}$//B3LYP-L+GD3/def2-SVP level of theory.[33–40] Rotation energy barriers were estimated using the SCAN coordinate function, implemented in Gaussian16.[32]

General Considerations

In addition to Bi(P(*t*Bu)Cy)$_3$ (**2**) presented in the main part, we computed three additional systems, namely Bi(P(*t*Bu)R)$_3$ with R = *i*Pr (**s1**), Ph (**s2**), and Mes (**s3**).

For each system, the relative thermodynamic stability of four different isomers was determined. In the next step, those isomers were identified, which are predicted to be most stable and are close enough in energy to envision an equilibrium scenario. For these isomers, interconversion via different isomerization mechanisms was investigated: a rotation - inversion sequence or a radical pathway involving homolytic Bi–P bond dissociation and re-association. The results are discussed in the sections below.

System Bi(P(*t*Bu)Cy)$_3$ (2)

In Figure S82 we present the relative thermodynamic stability of the isomers of compound **2**: **2a**, **2b**, **2c** and **2d**. Important geometry-optimized molecular structures for the discussion in this paragraph are shown in Cart S1.

The pathway for the isomerization of **2a** to give **2b**, which is discussed in the main part, consists of an inversion to give **2-inv** and a subsequent rotation step to then give **2b**. The relevant barrier for the rotation event (which is not rate-limiting) was estimated from the scan shown in Figure S83. All computed values are shown in Table S6.

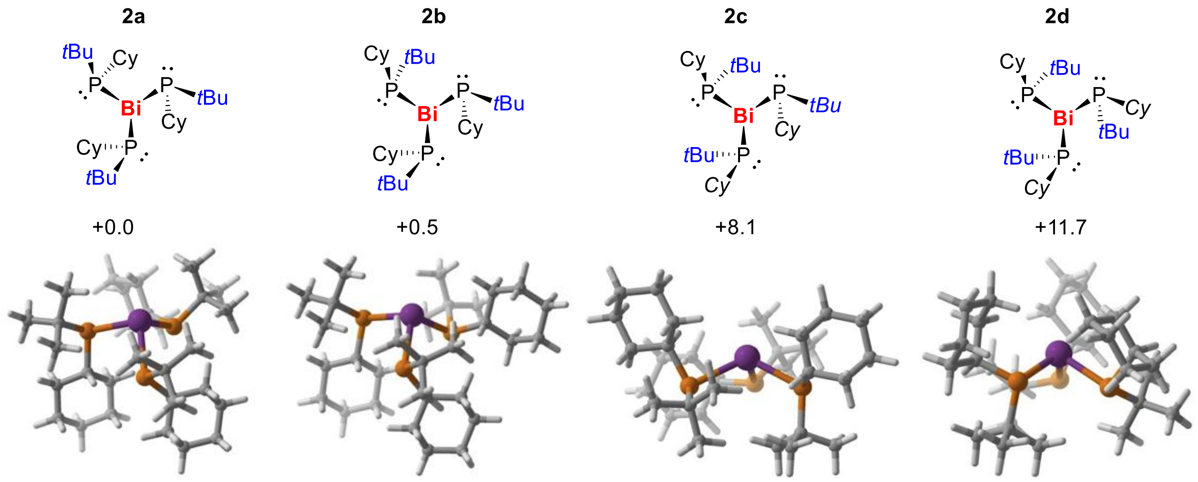

Figure S82. Relative thermodynamic stability of compound **2** isomers (numbers in kcal·mol^{-1}) and optimized structures.

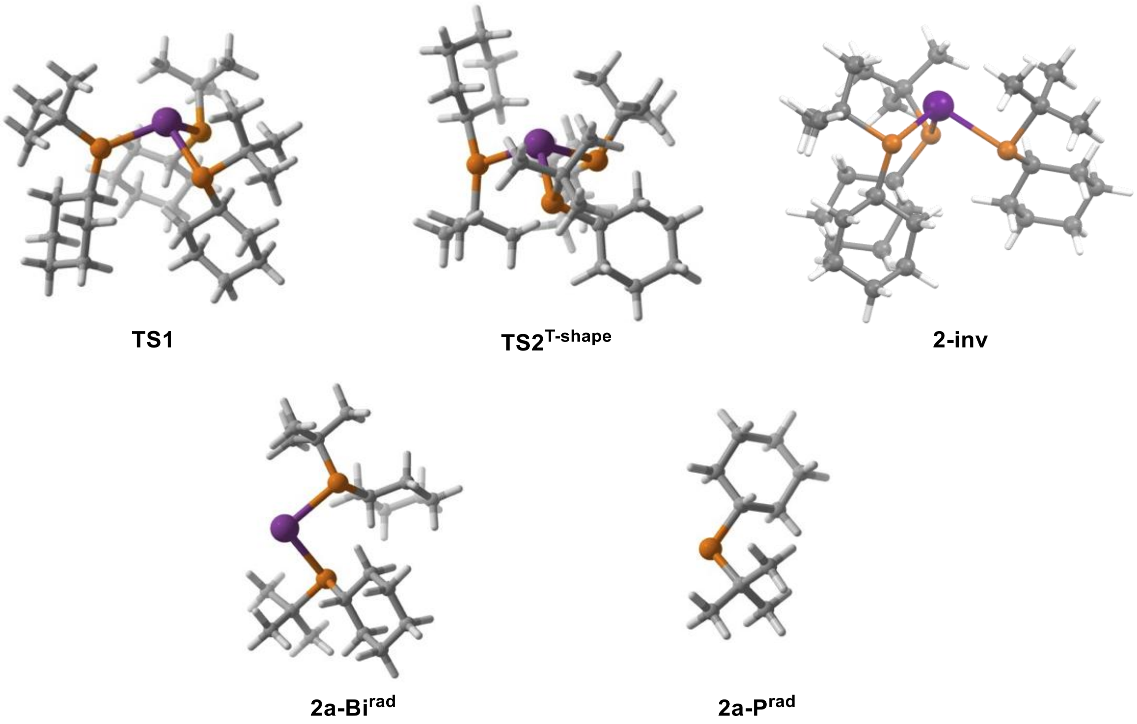

Chart S1. Additional computed structures including transition states (TS) and radical species (rad) for the system Bi(P(*t*Bu)Cy)3 (**2**).

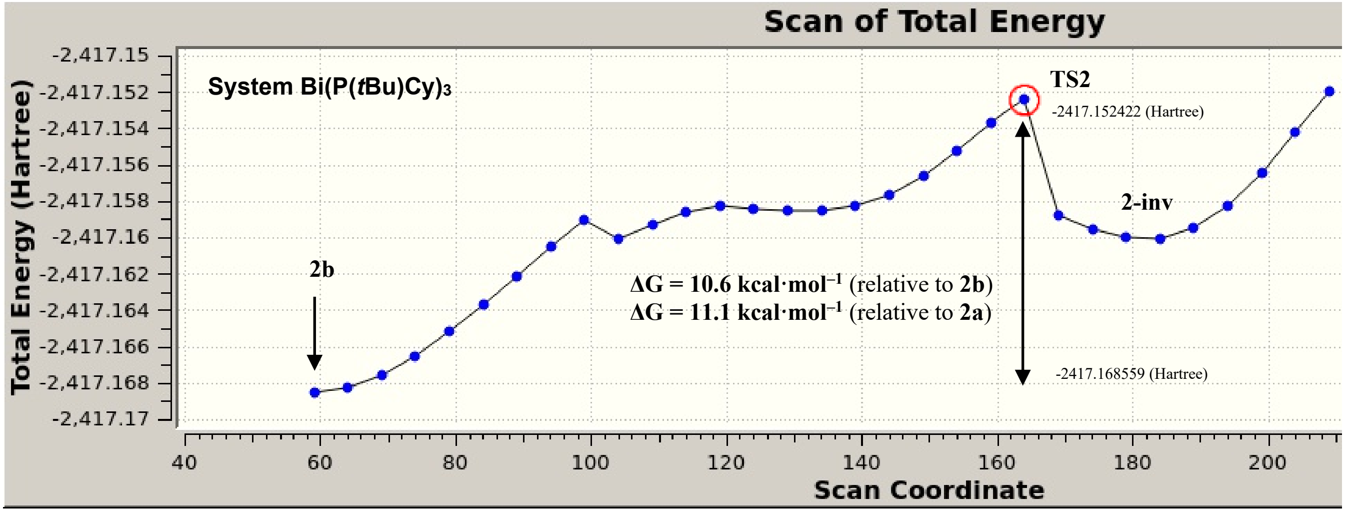

Figure S83. Rotational barrier calculation of the system Bi(P(*t*Bu)Cy)3 (**2**) for the pathway with an inversion/rotation sequence discussed in the main part.

If the inversion and rotation sequence proceeds in the reverse order as compared to the situation described in the main part (i.e. rotation to give **2-rot** through **TS1'** followed by a subsequent inversion to then give **2b**, the critical barrier is still that of the inversion step, but it is significantly higher in energy (**TS2'** = +25.5 kcal·mol^{-1}; Figure S84 (top)). The relevant barrier for the rotation event (which is not rate-limiting) was estimated from the scan shown in Figure S84 (bottom).

Alternatively, we also considered an isomerization mechanism via a transition state **TS-2**$^{\text{T-shape}}$, involving a T-shaped geometry around the phosphorus atom. Such a transition state **TS-2**$^{\text{T-shape}}$ (see Chart S1) accounts for a much higher free energy barrier (+54.9 kcal·mol^{-1}) than **TS-1** and is therefore rather unlikely to be relevant. It was therefore not considered for the other systems of this study.

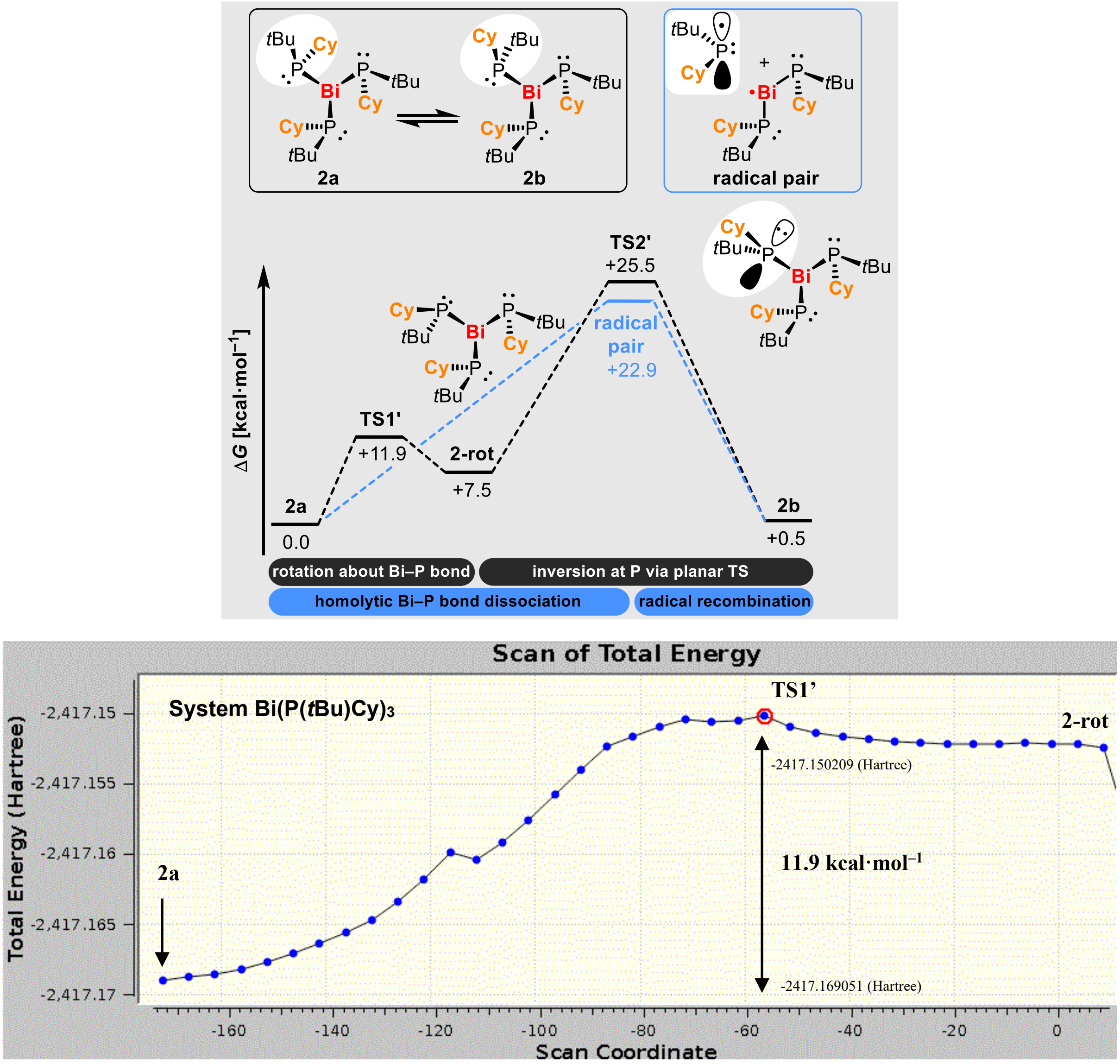

Figure S84. Top: rotation/inversion sequence for the isomerization of **2a** to give **2b** (with radical isomerization pathway given for comparison). Bottom: Rotational barrier calculation of the system Bi(P(*t*Bu)Cy)$_3$ (**2**) in the rotation/inversion sequence.

System Bi(P(*t*Bu)*i*Pr)$_3$ (s1)

In Figure S85, we present the relative thermodynamic stability of the isomers of compound **s1,** namely: **s1-a**, **s1-b**, **s1-c**, and **s1-d**. Isomers **s1-a** and **s1-b** are predicted to be the most stable isomers and present similar free energies ($\Delta G_{b\text{-}a}$ = +0.8 kcal·mol^{-1}), which suggests a plausible interconversion scenario like the one presented in the main part for the isomers **2a**/**2b**.

Starting from **s1-a**, rotation and inversion were considered. In agreement with the results obtained for compound **2**, the rotational barrier (**s1-TS1**: +12.6 kcal·mol^{-1}) is lower than the barrier for an inversion step (**s1-TS2**: +19.5 kcal·mol^{-1}), making the inversion the rate-limiting step. The radical isomerization pathway presents a slightly higher barrier (+21.9 kcal·mol^{-1}), which indicates that both pathways could operate at the same time, even more so at elevated temperature, when the dissociation pathway should be entropically favored. Scan calculations performed to estimate the rotational barrier of the system Bi(P(*t*Bu)*i*Pr)$_3$ (**s1**), converting **s1-a** into **s1-rot**, are shown in Figure S86. Additional computed structures, including transition states and radical species, are shown in Chart S2. All computed values are shown in Table S6.

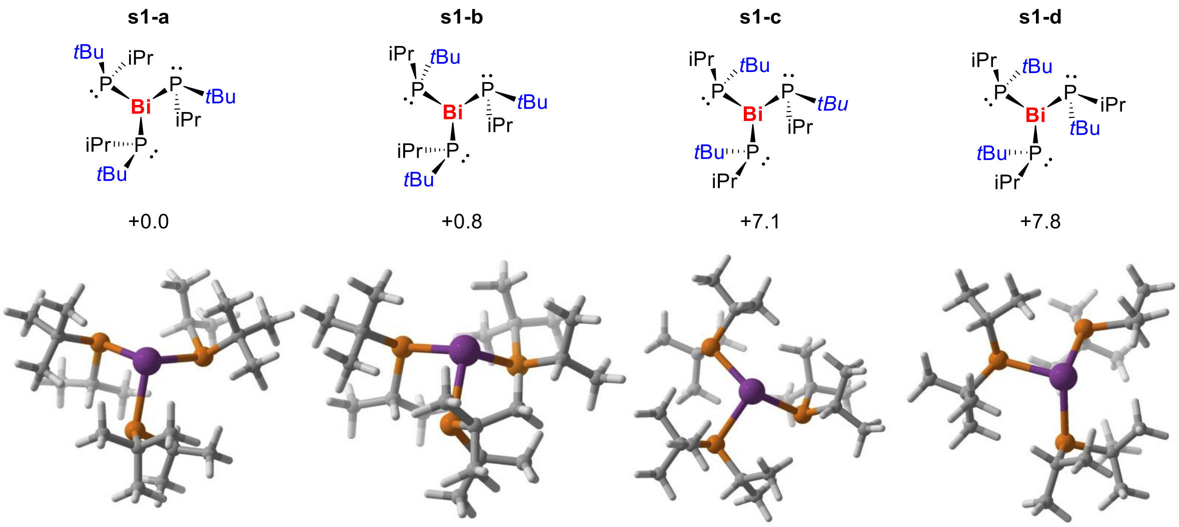

Figure S85. Relative thermodynamic stability of compound **s1** isomers (numbers in kcal·mol^{-1}) and optimized structures.

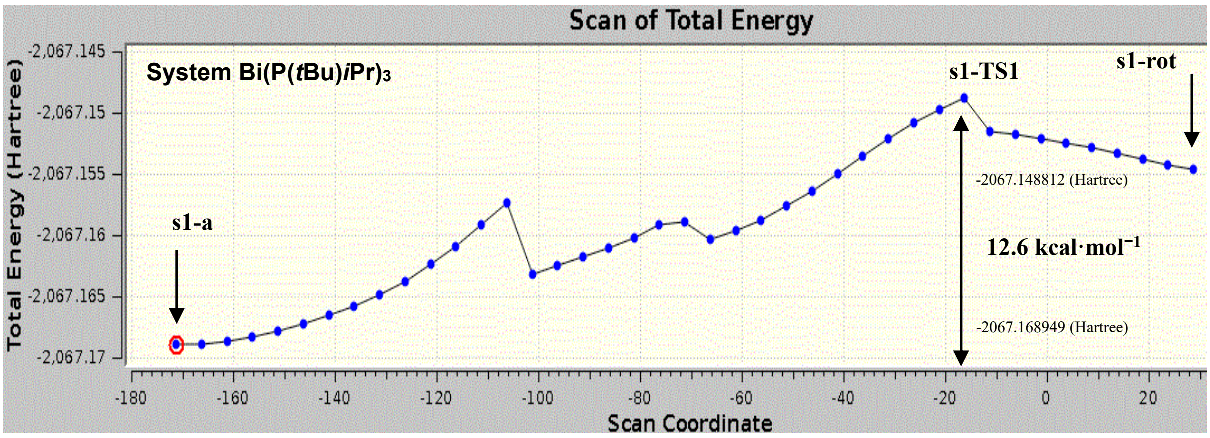

Figure S86. Rotational barrier calculation for the system Bi(P(*t*Bu)*i*Pr)$_3$ (**s1**).

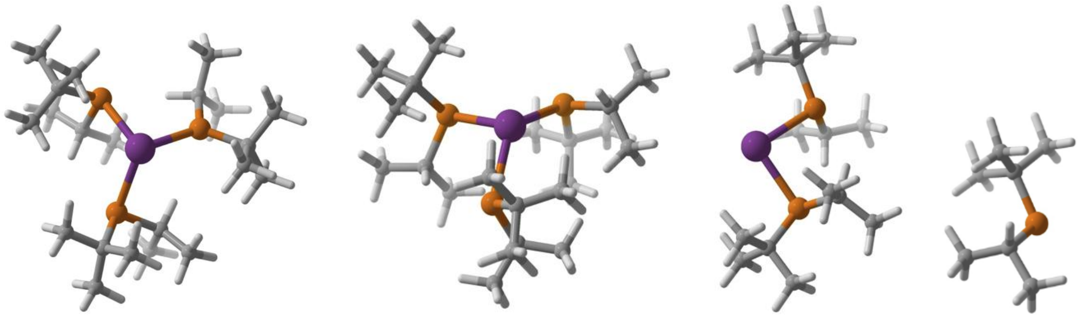

s1-TS2 s1-rot s1-a-Bi^{rad} s1-a-P^{rad}

Chart S2. Additional computed structures including transition states (TS) and radical species (rad) for the system Bi(P(*t*Bu)*i*Pr)$_3$ (**s1**).

System Bi(P(*t*Bu)Ph)$_3$ (s2)

In Figure S87 we present the relative thermodynamic stability of the isomers of compound **s2,** namely: **s2-a**, **s2-b**, **s3-c** and **s4-d**. Isomers **s2-a**, **s2-b** and **s2-d** are predicted to be the most stable isomers and present similar free energies. For comparison with the other systems, we present the plausible isomerization pathways between isomers **s2-a** and **s2-b**.

Starting from **s2-a**, rotation and inversion were considered. In agreement with the results obtained for compound **2**, the rotational barrier (**s2-TS1**: +8.3 kcal·mol^{-1}) is lower than the barrier for an inversion step (**s2-TS2**: +20.9 kcal·mol^{-1}), making the inversion the rate-limiting step. The radical isomerization pathway presents a slightly higher barrier (+23.0 kcal·mol^{-1}), which indicates that both pathways could operate at the same time, even more so at elevated temperature, when the dissociation pathway should be entropically favored. Scan calculations performed to estimate the rotational barrier of the system Bi(P(*t*Bu)Ph)$_3$ (**s2**), converting **s2-a** into **s2-rot**, are shown in Figure S88. Additional computed structures, including transition states and radical species, are shown in Chart S3. All computed values are shown in Table S6.

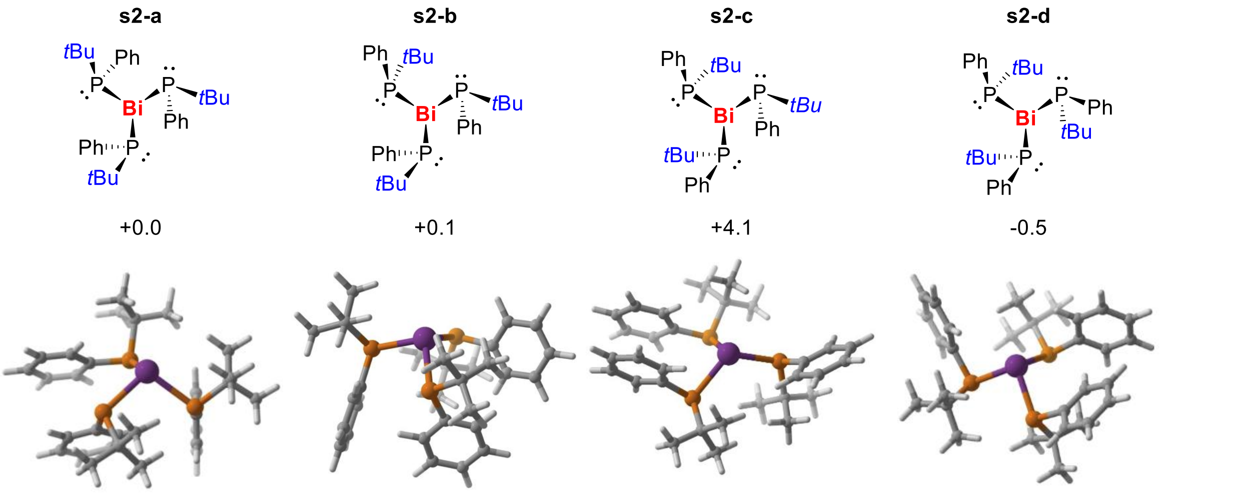

Figure S87. Relative thermodynamic stability of isomers of **s2** (numbers in kcal·mol^{-1}) and optimized structures.

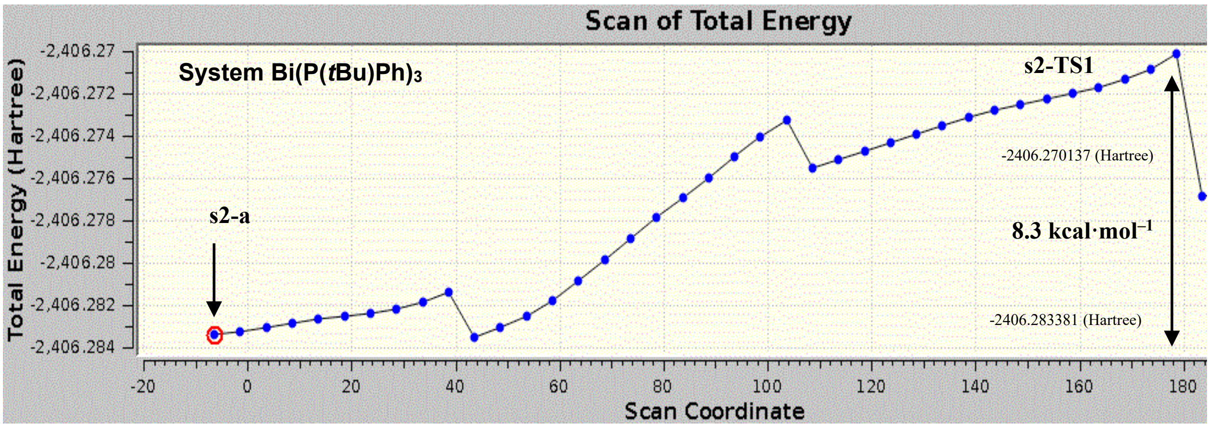

Figure S88. Rotational barrier calculations for the system Bi(P(*t*Bu)Ph)$_3$ (**s2**).

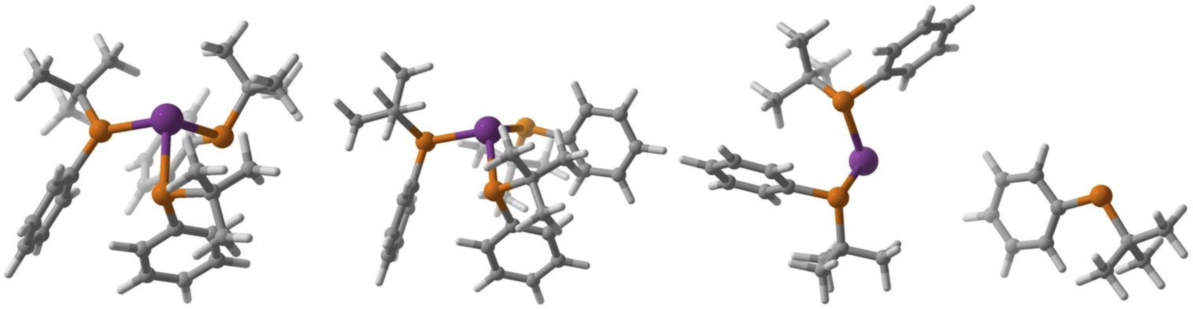

s2-TS2 s2-rot s2-a-Bi^{rad} s2-a-P^{rad}

Chart S3. Additional computed structures including transition states (TS) and radical species (rad) for the system Bi(P(*t*Bu)Ph)$_3$ (**s2**)

System Bi(P(*t*Bu)Mes)₃ (s3)

In Figure S89 we present the relative thermodynamic stability of the isomers of compound **s3,** namely: **s3-a**, **s3-b**, **s3-c** and **s3-d**. Isomers **s3-a** and **s3-b** are predicted to be the most stable isomers and present similar free energies. For comparison with the other systems, we present two pathways for the isomerization of **s3-a** to **s3-b**.

Starting from **s3-a**, rotation and inversion were considered. For **s3-a**, the rotational barrier (**s3-TS1**: +13.2 kcal·mol^{-1}) is lower than the barrier for an inversion step (**s3-TS2**: +14.2 kcal·mol^{-1}), but the energy values are close to each other highlighting the impact of the sterically demanding mesityl group. The radical isomerization pathway presents a slightly lower barrier (+12.0 kcal·mol^{-1}), which indicates that it would be slightly preferred at ambient temperature, even more so at elevated temperature, when the dissociation pathway should be entropically favored. Scan calculations performed to estimate the rotational barrier of the system Bi(P(*t*Bu)Mes)$_3$ (**s3**), converting **s3-a** into **s3-rot**, are shown in Figure S90. Additional computed structures, including transition states and radical species, are shown in Chart S2. All computed values are shown in Table S6.

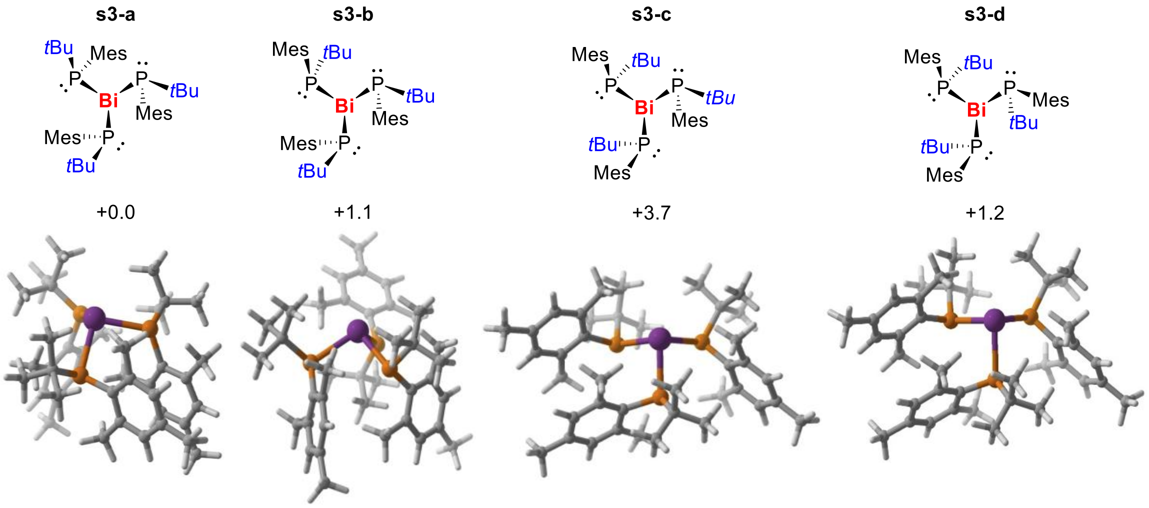

Figure S89. Relative thermodynamic stability of isomer of compound **s3** (numbers in kcal·mol^{-1}) and optimized structures.

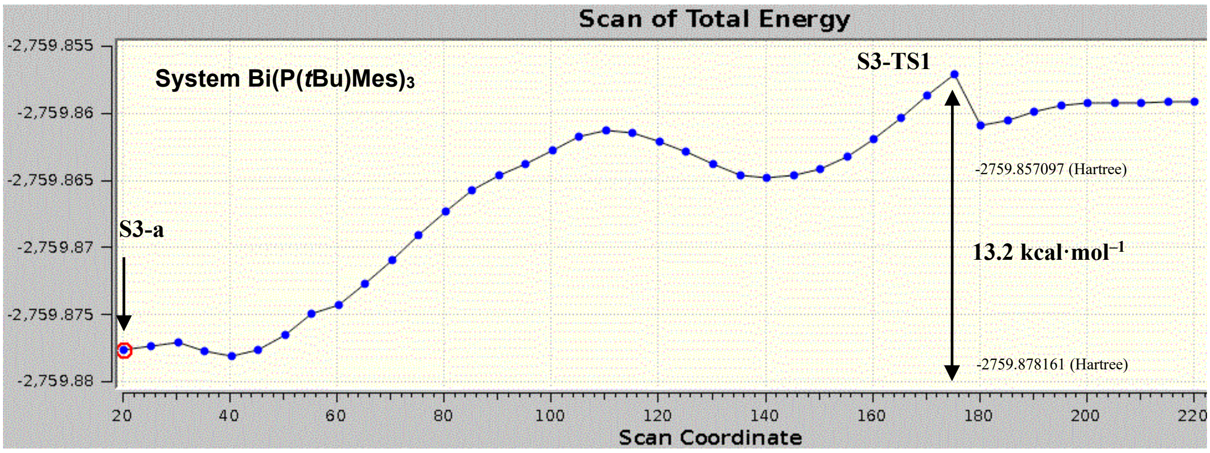

Figure S90. Rotational barrier calculations for the system Bi(P(*t*Bu)Mes)$_3$ (**s3**).

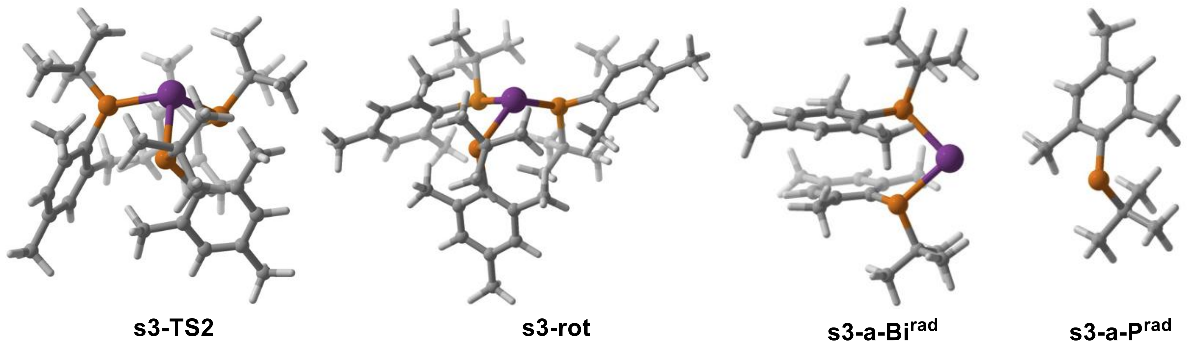

Chart S4. Additional computed structures including transition states (TS) and radical species (rad) for the system Bi(P(*t*Bu)Mes)$_3$ (**s3**).

Homolytic Bi–P bond dissociation energies of compounds 1, 3, and 4.

Below we present the computed homolytic Bi–P bond dissociation energies of compounds **1**, **3**, and **4**. Related computed structures are shown in Chart S5. All computed values are shown in Table S6.

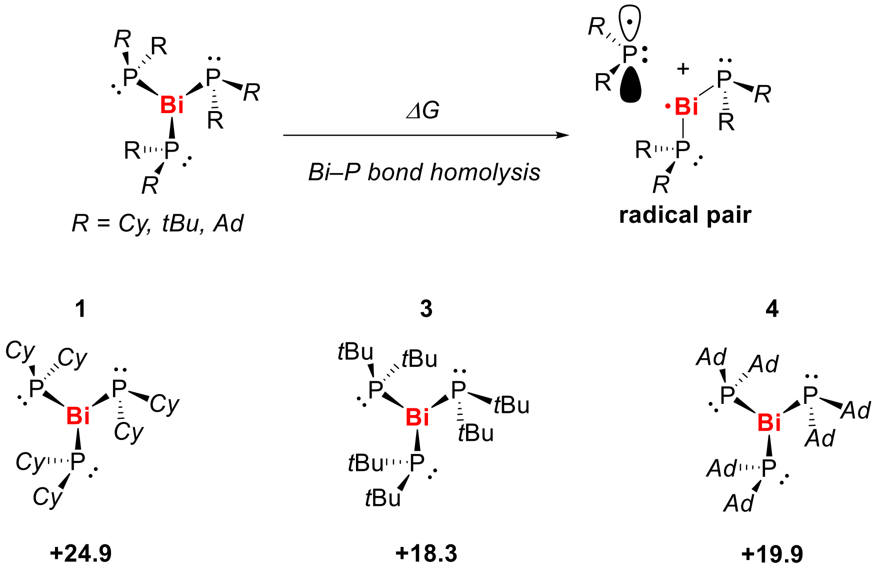

Figure S91. Homolytic Bi–P bond dissociation energies of compounds **1**, **3**, and **4** in kcal·mol^{-1}.

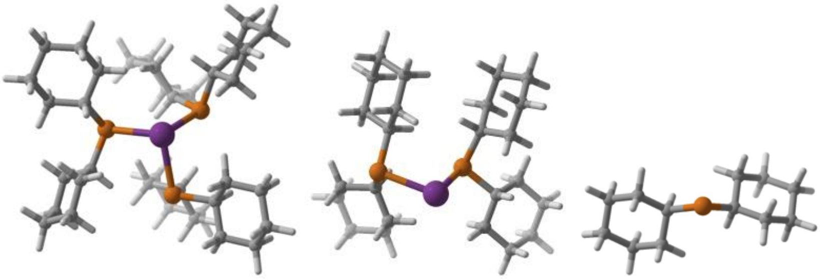

1 | 1-Bi^{rad} | 1-P^{rad}

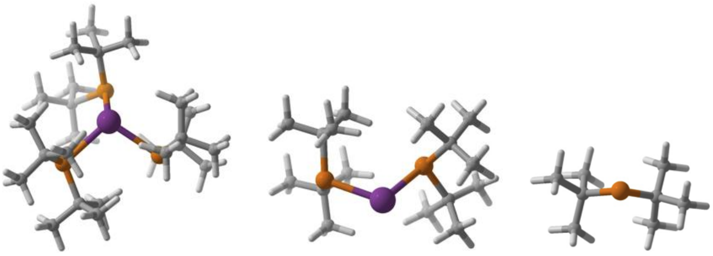

3 | 3-Bi^{rad} | 3-P^{rad}

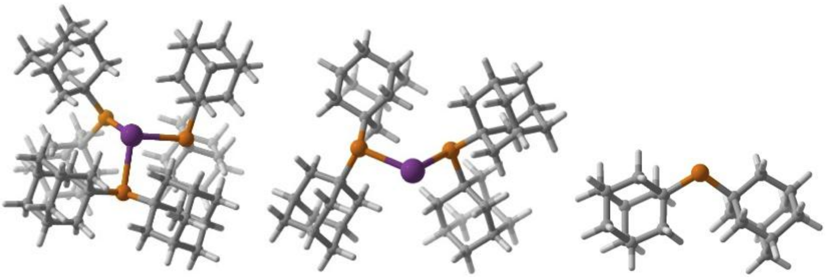

4 | 4-Bi^{rad} | 4-P^{rad}

Chart S5. Computed structures for homolysis of compounds **1**, **3** and **4**.

Alternative isomerization mechanisms.

For the isomerization between **2a** and **2b**, the mechanisms of inversion (via a T-shaped and a trigonal planar transition state) and homolytic dissociation have been discussed above. In addition, a polar mechanism via $[Bi(PtBuCy)_2]^+$ and $[PtBuCy]^-$, resulting from heterolytic Bi–P bond cleavage, can also be considered. DFT calculations assign a heterolytic bond dissociation energy of $\Delta G = +91.5$ kcal·mol^{-1} to this process, making it unrealistic under the given conditions. Furthermore, a mechanism involving a bismuthinidene [Bi(P*t*BuCy)$_2$] and a diphosphane *t*BuCyP–PCy*t*Bu may also be considered. The triplet and the singlet electronic structure of the bismuthinidene were considered, the triplet structure being lower in energy by 3.6 kcal·mol^{-1}. It has to be pointed out that the applied level of theory only gives a first approximation of these energies. The formation of ^{3}[Bi(P*t*BuCy)$_2$] and *t*BuCyP–PCy*t*Bu from **2a** is endergonic by 19.8 kcal·mol^{-1}. Importantly, this does not include a kinetic barrier for this process, yet; i.e. the relevant barrier for this process can be expected to be significantly higher. In addition, the bismuthinidene would be expected to be very short-lived and would be expected to undergo rapid degradation (rather than selective insertion into the P–P bond of the diphosphane). In this

situation, we decided to perform an experiment as an additional test for this mechanism. Specifically, we performed ^{31}P-^{31}P EXSY NMR spectroscopy on a sample containing **2a**, **2b**, and the diphosphane *t*BuCyP–PCy*t*Bu. No cross signals between the diphosphane and another species were observed, ruling out this mechanistic scenario for the isomerization between **2a** and **2b**.

Insertion reactions of 3 with olefins.

The thermodynamic parameters of the insertion reactions of **3** with ethylene, 1-pentene, and 1-hexene to give compounds **9-H** (not experimentally observed), **9-*n*Pr**, and **9-*n*Bu** have been determined by DFT calculations by geometry optimizations and frequency analyses with a benzene solvent model (for general theoretical approach see above). All these reactions are exothermic (ΔH = −11.4 / –6.4 / −6.6 kcal·mol^{-1} for the formation of **9-H** / **9-*n*Pr** / **9-*n*Bu**), but endergonic (ΔG = +1.1 / +8.1 / +7.9 kcal·mol^{-1} for the formation of **9-H** / **9-*n*Pr** / **9-*n*Bu**). This is in agreement with an excess of the olefin being necessary in order to obtain relevant yields of the desired insertion products **9-*n*Pr** and **9-*n*Bu** and with the release of the olefins 1-pentene and 1-hexene from the isolated compounds **9-*n*Pr** and **9-*n*Bu** upon solvation in a solvent other than the olefin. The almost identical thermodynamic parameters for the formation of **9-*n*Pr** and **9-*n*Bu**, respectively, are also in line with the results of the olefin exchange experiments (formation of **9-*n*Bu** upon solvation of **9-*n*Pr** in 1-hexene). The fact that the insertion of ethylene into a Bi–P bond of **3** is thermodynamically more facile than in the cases of the other olefins suggests that subsequent insertion events might also be more favorable than in the case of the other olefins. This would be in line with the experimentally observed formation of **8b** and **8c**, which are suggested to proceed via the insertion of ethylene into the Bi–C bond of **9-H**.

Computed values for the optimized structures.

Table S6. G_{corr} is the thermal correction to Gibbs free energy and $G_{298\ gas\ phase}$ is the sum of electronic and thermal free energies at the B3LYP+GD3/def2svp level of theory in gas phase (values in hartree); $E_{0\ benzene}$ is the electronic energy obtained at B3LYP+GD3/def2tzvp(SMD)//M06L+GD3/def2svp level of theory in benzene as the solvent (SMD, solvation model based on density; values in hartree); $G_{298\ benzene}$ is the sum of $E_{0\ benzene}$ and G_{corr} (values in hartree). Note that **2-rot** and **TS2'** are geometries for the rotation/inversion sequence, which is not discussed in the main part, since the key TS is higher in energy than in the inversion/rotation sequence.

Structure	G_{corr}	G_{298} gas phase	E_0 benzene	G_{298} benzene
**System Bi(P(*t*Bu)Cy)$_3$ (2)**				
2a	0.776445	-2416.392626	-2418.825413	-2418.048968
2b	0.777189	-2416.391370	-2418.825294	-2418.048105
2c	0.774631	-2416.378876	-2418.810762	-2418.036131
2d	0.776369	-2416.374007	-2418.806729	-2418.030360
TS1	0.775748	-2416.356063	-2418.791446	-2418.015698
TS2‘	0.773271	-2416.348594	-2418.781589	-2418.008318
$TS2^{T\text{-}shape}$	0.773262	-2416.303271	-2418.734674	-2417.961412
2-inv	0.777111	-2416.384655	-2418.819551	-2418.04244
2-rot	0.777406	-2416.378387	-2418.814494	-2418.037088
2a-Bi^{rad}	0.504903	-1682.493842	-1684.108348	-1683.603445
2a-P^{rad}	0.242523	-733.855630	-734.651639	-734.409116
**System Bi(P(*t*Bu)*i*Pr)$_3$ (s1)**				
s1-a	0.582641	-2066.586320	-2068.456071	-2067.873430
s1-b	0.583590	-2066.585345	-2068.455806	-2067.872216
s1-c	0.583406	-2066.575134	-2068.445559	-2067.862153
s1-d	0.581951	-2066.574499	-2068.443012	-2067.861061
s1-TS2	0.581869	-2066.553361	-2068.424238	-2067.842369
s1-inv	0.583206	-2066.578438	-2068.450282	-2067.867076
s1-rot	0.583044	-2066.573551	-2068.445044	-2067.862000
s1-a-Bi^{rad}	0.376274	-1449.291508	-1450.529989	-1450.153715
s1-a-P^{rad}	0.178520	-617.255333	-617.863350	-617.684830
**System Bi(P(*t*Bu)Ph)$_3$ (s2)**				
s2-a	0.568285	-2405.715096	-2407.930687	-2407.362402
s2-b	0.570468	-2405.715060	-2407.932691	-2407.362223
s2-c	0.566177	-2405.707442	-2407.922049	-2407.355872
s2-d	0.568276	-2405.715109	-2407.931437	-2407.363161
s2-TS2	0.571559	-2405.680543	-2407.900582	-2407.329023

s2-inv	0.570342	-2405.710213	-2407.927827	-2407.357485
s2-rot	0.570498	-2405.708562	-2407.926529	-2407.356031
s2-a-Bi^{rad}	0.366205	-1675.374400	-1676.845200	-1676.479000
s2-a-P^{rad}	0.173996	-730.296378	-731.020700	-730.846704
**System Bi(P(*t*Bu)Mes)$_3$ (s3)**				
s3-a	0.799774	-2759.077908	-2761.906205	-2761.106431
s3-b	0.801113	-2759.075286	-2761.905838	-2761.104725
s3-c	0.802316	-2759.072696	-2761.902894	-2761.100578
s3-d	0.799120	-2759.076014	-2761.903686	-2761.104566
s3-TS2	0.799836	-2759.052498	-2761.883435	-2761.083599
s3-inv	0.798471	-2759.079802	-2761.90574	-2761.107269
s3-rot	0.796932	-2759.068927	-2761.897175	-2761.100243
s3-a-Bi^{rad}	0.522689	-1910.960317	-1912.839563	-1912.316874
s3-a-P^{rad}	0.248238	-848.092447	-849.0186262	-848.7703882
**Bi–P homolysis of compounds 1, 3 and 4**				
1	0.887125	-2648.418649	-2651.206386	-2650.319261
1-Bi^{rad}	0.579821	-1837.176162	-1839.027432	-1838.447611
1-P^{rad}	0.27894	-811.197251	-812.1109105	-811.8319705
3	0.663369	-2184.351066	-2186.428993	-2185.765624
3-Bi^{rad}	0.430878	-1527.803387	-1529.181605	-1528.750727
3-P^{rad}	0.205747	-656.513272	-657.1914515	-656.9857045
4	1.320602	-3576.467637	-3580.637564	-3579.316962
4-Bi^{rad}	0.868453	-2455.879855	-2458.65349	-2457.785037
4-P^{rad}	0.423962	-1120.548062	-1121.924161	-1121.500199
**Polar and bismuthinidene-type mechanism**				
[Bi(PCy*t*Bu)$_2$]$^+$	0.506943	-1682.274454	-1683.932713	-1683.42577
(PCy*t*Bu)$^-$	0.241111	-733.872409	-734.7184781	-734.4773671
^{1}[Bi(PCy*t*Bu)]	0.240031	-948.583098	-949.3880541	-949.1480231
^{3}[Bi(PCy*t*Bu)]	0.239043	-948.595003	-949.3927716	-949.1537286
*t*BuCyP–PCy*t*Bu	0.515473	-1467.762079	-1469.379134	-1468.863661
**Reactions of olefins with 3**				
Ethylene	0.029145	-78.503298	-78.62559419	-78.59644919
1-Pentene	0.106791	-196.295022	-196.6260875	-196.5192965
1-Hexene	0.133055	-235.55704	-235.9578447	-235.8247897
9-H	0.716221	-2262.854677	-2265.076685	-2264.360464
9-*n*Pr	0.797017	-2380.639345	-2383.069034	-2382.272017
9-*n*Bu	0.823341	-2419.901714	-2422.401217	-2421.577876

X-Ray diffraction analysis and molecular structures

Data for **1** (CCDC 2451991), **2** (CCDC 2451992), **3** (CCDC 2451993), **5** (CCDC 2451994), **7** (CCDC 2502129) and **9-CF₃** (CCDC 2451995) were collected at 100 K, if not noted otherwise, on a Bruker Quest D8 diffractometer or a Bruker D8 Venture using a graphite-monochromated Mo-K λ radiation (λ = 0.71073Å) and equipped with an *Oxford Instrument Cooler Device.* The structures have been solved using either OLEX SHELXT V2014/1[41,42] and refined by means of least-squares procedures on a F^2 with the aid of the program SHELXL-2016/6[43] include in the software package WinGX version 1.63[43] or using CRYSTALS.[44] The Atomic Scattering Factors were taken from *International Tables for X-Ray Crystallography*.[45] All non-hydrogen atoms were refined anisotropically. All hydrogens atoms were refined by using a riding model. Absorption corrections were introduced by using the MULTISCAN and X-Red program.[42,46] Drawings of molecules are performed with the programs MERCURY and POV-Ray with 50% probability displacement ellipsoids for non-H atoms. Depiction of H atoms is generally omitted for clarity.

Table S9. Crystal data and structure refinement for [Bi(PCy$_2$)$_3$] (**1**).

Identification code	**1**_SRPX19a_0m
CCDC Number	2451991
Empirical formula	$C_{36}H_{66}BiP_3$
Formula weight / g mol^{-1}	800.77
Temperature / K	100
Crystal system	triclinic
Space group	$P\bar{1}$
a / Å	10.975(2)
b / Å	13.652(4)
c / Å	14.195(4)
α / °	98.330(17)
β / °	105.443(14)
γ / °	109.025(15
V / Å^3	1874.4(9)
Z	2
ρ_{calc} / g cm^{-3}	1.419
μ / mm^{-1}	4.853
F(000)	820.0
Crystal size / mm^3	0.09 × 0.067 × 0.051
Radiation	MoKα (λ = 0.71073)
2Θ range for data collection / °	3.874 to 49.998
Index ranges	$-13 \le h \le 13$, $-16 \le k \le 16$, $-16 \le l \le 16$
Reflections collected	46698
Independent reflections	6588 [R_{int} = 0.0644, R_{sigma} = 0.0364]
Data/restraints/parameters	6588/0/361
Goodness-of-fit on F^2	1.154
Final *R* indexes [*I*≥2σ (*I*)]	R_1 = 0.0427, wR_2 = 0.0986
Final *R* indexes [all data]	R_1 = 0.0474, wR_2 = 0.1002
Largest diff. peak/hole / e Å^{-3}	5.37/−3.39

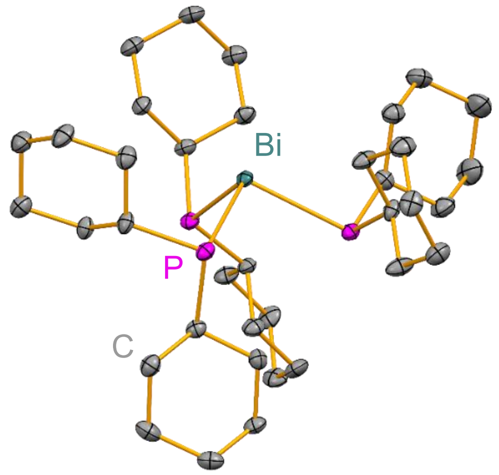

Figure S92. Molecular structure of [Bi(PCy$_2$)$_3$] (**1**). Displacement ellipsoids are shown at the 50% probability level. The quality of the diffraction data does not allow for a detailed discussion of bonding parameters, but the connectivity is definite.

Table S10. Crystal data and structure [Bi(P(*t*Bu)Cy)$_3$] (**2**).

Identification code	**2**_SRPX21a_1
CCDC Number	2451992
Empirical formula	$C_{30}H_{60}BiP_3$
Formula weight / g mol^{-1}	722.67
Temperature / K	100
Crystal system	triclinic
Space group	$P\bar{1}$
a / Å	10.6674(18)
b / Å	11.2245(18)
c / Å	16.111(3)
α / °	105.555(10)
β / °	90.969(10)
γ / °	115.023(9)
V / Å^3	1665.1(5)
Z	2
ρ_{calc} / g cm^{-3}	1.441
μ / mm^{-1}	5.455
F(000)	736.0
Crystal size / mm^3	0.117 x 0.106 x 0.05
Radiation	MoKα (λ = 0.71073)
2Θ range for data collection / °	4.204 to 60.094
Index ranges	$-15 \le h \le 15$, $-15 \le k \le 15$, $-22 \le l \le 22$
Reflections collected	138525
Independent reflections	9739 [R_{int} = 0.0524, R_{sigma} = 0.0201]
Data/restraints/parameters	9739 /0/ 316
Goodness-of-fit on F^2	1.069
Final *R* indexes [*I*≥2σ (*I*)]	R_1 = 0.0156, wR_2 = 0.0312
Final *R* indexes [all data]	R_1 = 0.0175, wR_2 = 0.0317
Largest diff. peak/hole / e Å^{-3}	0.78/−0.87

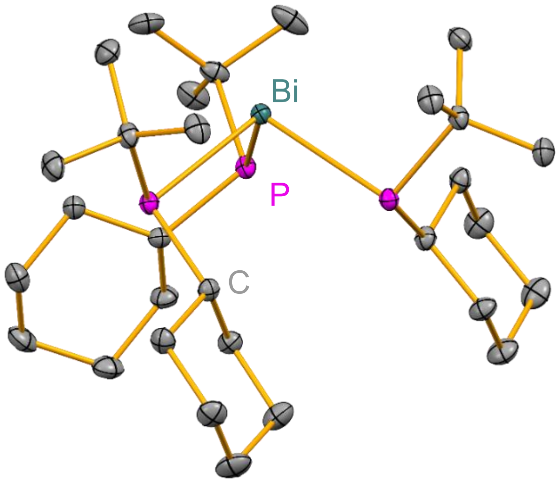

Figure S93. Molecular structure of [Bi(P(*t*Bu)Cy)$_3$] (**2**). Displacement ellipsoids are shown at the 50% probability level.

Table S8. Crystal data and structure refinement for Bi(P*t*Bu$_2$)$_3$ (**3**).

Identification code	**3**_KaOb025Mo_a
CCDC Number	2451993
Empirical formula	$C_{24}H_{54}BiP_3$
Formula weight / g mol^{-1}	644.56
Temperature / K	99.9(5)
Crystal system	monoclinic
Space group	$P2_1/c$
a / Å	11.5915(3)
b / Å	16.9444(4)
c / Å	16.3179(5)
α / °	90
β / °	108.020(3)
γ / °	90
V / Å^3	3047.82(15)
Z	4
ρ_{calc} / g cm^{-3}	1.405
μ / mm^{-1}	5.950
F(000)	1304.0
Crystal size / mm^3	0.063 × 0.092 × 0.122
Radiation	MoKα (λ = 0.71073)
2Θ range for data collection / °	4.4082 to 64.4888
Index ranges	$-16 \leq h \leq 16$, $-23 \leq k \leq 24$, $-23 \leq l \leq 23$
Reflections collected	36069
Independent reflections	9096 [R_{int} = 0.0454, R_{sigma} = 0.0483]
Data/restraints/parameters	9096/0/279
Goodness-of-fit on F^2	1.059
Final *R* indexes [*I*≥2σ (*I*)]	R_1 = 0.0323, wR_2 = 0.0704
Final *R* indexes [all data]	R_1 = 0.0490, wR_2 = 0.0743
Largest diff. peak/hole / e Å^{-3}	2.08/−0.91

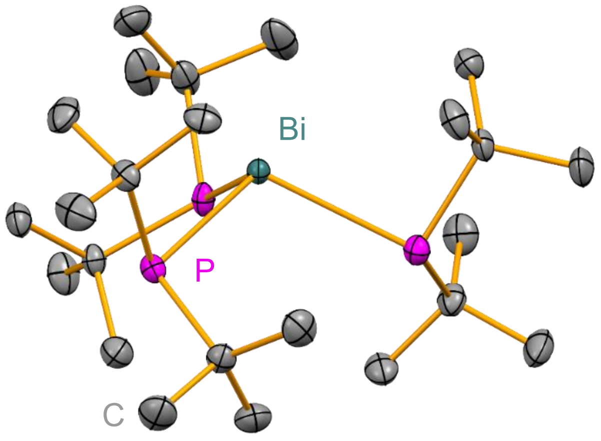

Figure S94. Molecular structure of Bi(P*t*Bu$_2$)$_3$ (**3**). Displacement ellipsoids are shown at the 50% probability level.

Table S11. Crystal data and structure refinement for $P_2Cy_2tBu_2$.

Identification code	SRPX31a_2
CCDC Number	2451994
Empirical formula	$C_{20}H_{40}P_2$
Formula weight / g mol^{-1}	342.46
Temperature / K	278.15
Crystal system	monoclinic
Space group	$P2_1/n$
a / Å	8.8768(5)
b / Å	18.1553(9)
c / Å	13.2912(6)
α / °	90
β / °	90.053(2)
γ / °	90
V / Å^3	2142.02(19)
Z	4
ρ_{calc} / g cm^{-3}	1.062
μ / mm^{-1}	0.201
F(000)	760.0
Crystal size / mm^3	0.091 × 0.251 × 0.252
Radiation	MoKα (λ = 0.71073)
2Θ range for data collection / °	3.798 to 60.142
Index ranges	$-12 \le h \le 12$, $-25 \le k \le 25$, $-18 \le l \le 18$
Reflections collected	40063
Independent reflections	6275 [R_{int} = 0.0510, R_{sigma} = 0.0378]
Data/restraints/parameters	6275/0/206
Goodness-of-fit on F^2	1.076
Final *R* indexes [$I \geq 2\sigma(I)$]	R_1 = 0.0429, wR_2 = 0.0999
Final *R* indexes [all data]	R_1 = 0.0547, wR_2 = 0.1050
Largest diff. peak/hole / e Å^{-3}	0.25/−0.25

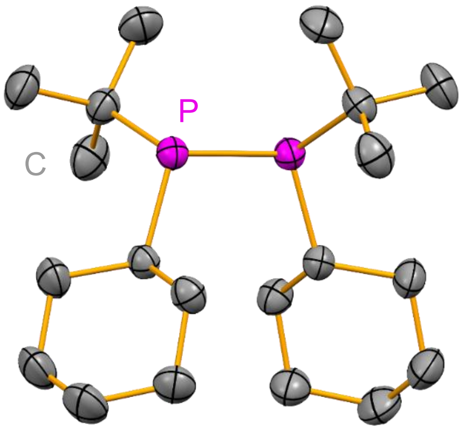

Figure S95. Molecular structure of $P_2Cy_2tBu_2$. Displacement ellipsoids are shown at the 50% probability level.

Table S11. Crystal data and structure refinement for **7**.

Identification code	**7**_SRPX20a_1
CCDC Number	2502129
Empirical formula	$C_{17}H_{36}P_2S_2$
Formula weight / g mol^{-1}	366.52
Temperature / K	100.00
Crystal system	triclinic
Space group	$P\bar{1}$
a / Å	8.7077(4)
b / Å	12.9666(6)
c / Å	14.9308(7)
α / °	105.127(2)
β / °	90.987(2)
γ / °	95.913(2)
V / Å^3	1617.04(13)
Z	3
ρ_{calc} / g cm^{-3}	1.129
μ / mm^{-1}	0.390
F(000)	600
Crystal size / mm^3	0.86 × 0.78 × 0.293
Radiation	MoKα (λ = 0.71073)
2Θ range for data collection / °	3.718 to 60.102
Index ranges	$-12 \le h \le 12$, $-18 \le k \le 18$, $-21 \le l \le 21$
Reflections collected	123184
Independent reflections	9439 [R_{int} = 0.0333, R_{sigma} = 0.0153]
Data/restraints/parameters	9439/3/354
Goodness-of-fit on F^2	1.190
Final R indexes [$I \geq 2\sigma(I)$]	R_1 = 0.0533, wR_2 = 0.1200
Final R indexes [all data]	R_1 = 0.0553, wR_2 = 0.1210
Largest diff. peak/hole / e Å^{-3}	1.60/−0.65

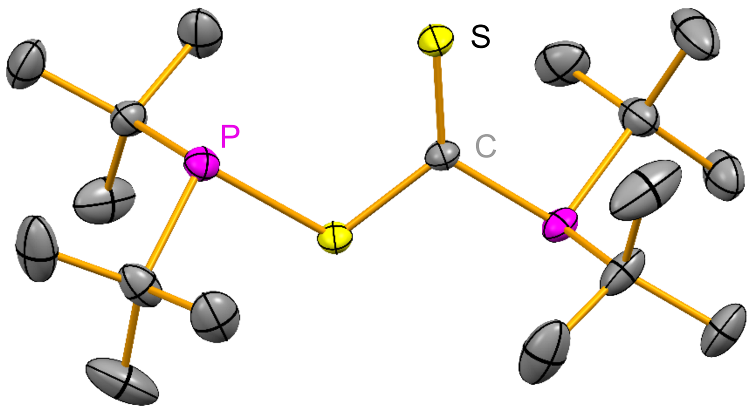

Figure S96. Molecular structure of the main species of product **7**. Displacement ellipsoids are shown at the 50% probability level. The disorder around the P–S–C–P axis and the second species (disordered and on a inversion center) were not shown here. Hydrogen atoms are omitted for clarity.

Table S11. Crystal data and structure refinement for **9-CF3**.

Identification code	**9-CF3**_KaOb033_JL23_a
CCDC Number	2451995
Empirical formula	$C_{27}H_{57}BiF_3P_3$
Formula weight / g mol^{-1}	740.61
Temperature / K	180(2)
Crystal system	monoclinic
Space group	$P2_1/n$
a / Å	10.733(2)
b / Å	16.156(5)
c / Å	19.970(6)
α / °	90
β / °	100.112(13)
γ / °	90
V / Å^3	3409.1(16)
Z	4
ρ_{calc} / g cm^{-3}	1.443
μ / mm^{-1}	5.342
F(000)	14960
Crystal size / mm^3	0.212 × 0.139 × 0.134
Radiation	MoKα (λ = 0.71073)
2Θ range for data collection / °	4.766 to 53.006
Index ranges	$-13 \le h \le 13$, $-20 \le k \le 20$, $-25 \le l \le 24$
Reflections collected	92992
Independent reflections	7031 [R_{int} = 0.0618, R_{sigma} = 0.0255]
Data/restraints/parameters	7031/0/327
Goodness-of-fit on F^2	1.087
Final R indexes [$I \geq 2\sigma(I)$]	R_1 = 0.0377, wR_2 = 0.0864
Final R indexes [all data]	R_1 = 0.0484, wR_2 = 0.0908
Largest diff. peak/hole / e Å^{-3}	3.30/−0.72

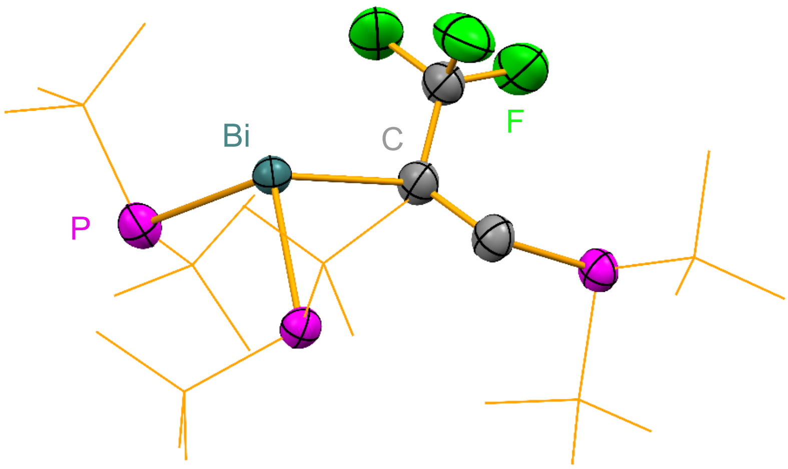

Figure S97. Molecular structure of the insertion product **9-CF3**. Displacement ellipsoids are shown at the 50% probability level.

Cartesian coordinates

2a

Bi	0.02677100	-0.10357700	-1.44240000
P	-0.84933100	-1.97574200	0.30916500
P	2.34090700	0.62399600	-0.24286600
P	-1.46665600	1.92386700	-0.40736500
C	3.49546800	-1.27788800	-2.19852100
H	3.55757300	-2.08838600	-1.46174100
H	4.29775100	-1.44181100	-2.94089800
H	2.53703200	-1.37709800	-2.73268600
C	2.52575500	-0.49026700	1.27723400
H	1.50839900	-0.43707000	1.70152900
C	3.65262200	0.11351800	-1.56063600
C	3.46975600	0.14680000	2.32114300
H	4.51133800	0.10304800	1.95808400
H	3.23271200	1.21553000	2.44840300
C	2.84822800	-1.98149400	1.11035800
H	2.16998200	-2.43781700	0.37931200
H	3.87315000	-2.10107100	0.71901500
C	-0.49552000	2.43704300	1.14709400
H	0.57610000	2.49211600	0.88837100
C	-2.60162800	-1.35194300	0.68498900
H	-2.39406700	-0.29909300	0.93392400
C	5.04127200	0.22168400	-0.90079700
H	5.18476500	1.19686800	-0.40863100
H	5.82836200	0.11343300	-1.66831800
H	5.20375200	-0.56648800	-0.15130900
C	-1.40163800	2.73054700	-3.11230500
H	-0.61524400	2.03258700	-3.43871900
H	-1.43733700	3.54845400	-3.85279800
H	-2.36778100	2.20050600	-3.15522100
C	-1.12772100	3.31173400	-1.71087300
C	3.37112000	-0.58707700	3.66697800
H	4.06811100	-0.13798700	4.39507500
H	2.35431400	-0.44375800	4.07593100
C	-1.56183700	-3.35124300	-2.22373500
H	-2.57755500	-2.93709900	-2.22064300
H	-1.60164800	-4.32446300	-2.74597100
H	-0.92669500	-2.68607700	-2.82943800
C	-0.67491400	1.36230200	2.23987000
H	-1.74934000	1.27133000	2.48351300
H	-0.35677600	0.37379200	1.87629800
C	-0.99659000	-3.54979500	-0.80680600
C	3.54820700	1.18345500	-2.66707400
H	2.56808100	1.16063500	-3.17244600
H	4.31727500	1.00438900	-3.43898100
H	3.69754500	2.19836200	-2.26634800
C	0.29498200	3.88961200	-1.66686100
H	0.51890100	4.37969000	-0.70853300
H	0.41753600	4.64858200	-2.46056500
H	1.05979200	3.11353200	-1.82239600
C	3.64756500	-2.08810500	3.51497200
H	3.52400200	-2.60152500	4.48358100
H	4.70341800	-2.23142200	3.21754400
C	2.73862900	-2.71842100	2.45270800
H	1.68689700	-2.67848000	2.78910700
H	2.98551700	-3.78585000	2.31903400
C	-4.92920900	-0.57204500	0.07282800
H	-4.66142500	0.48493800	0.25071300
H	-5.69942700	-0.57441300	-0.71739400
C	-3.67607200	-1.31616200	-0.41326700
H	-3.95726100	-2.34382000	-0.70043000
H	-3.28329000	-0.82583400	-1.31844100
C	-0.92543100	3.80623800	1.71363800
H	-0.75441300	4.60474300	0.97904300
H	-2.01201500	3.79332300	1.91908900
C	0.09562800	1.70397200	3.52100800
H	1.17674300	1.71492600	3.29938200
H	-0.06122100	0.91253300	4.27338800
C	-3.15645000	-1.98710200	1.97891500
H	-3.40808100	-3.04724500	1.80482500
H	-2.38326500	-1.97502900	2.76461400
C	-4.41253800	-1.24367100	2.45767200
H	-4.81267100	-1.72361000	3.36709300
H	-4.13120400	-0.21314100	2.74355700

C	-1.86911500	-4.56852300	-0.04793600
H	-2.92503700	-4.26336600	-0.01701500
H	-1.52315200	-4.71360500	0.98803400
H	-1.82461000	-5.54776700	-0.55706200
C	-5.48473000	-1.18622500	1.36313200
H	-6.36264900	-0.61750800	1.71370500
H	-5.84052700	-2.21256200	1.15293600
C	-2.17123200	4.42610200	-1.47096600
H	-3.19752800	4.03447900	-1.55120200
H	-2.05022900	5.21622100	-2.23365500
H	-2.07194800	4.90228300	-0.48678900
C	-0.16333400	4.15318200	3.00271900
H	0.90932600	4.26976400	2.76144900
H	-0.50621800	5.12969500	3.38522600
C	-0.32076900	3.06979700	4.07341400
H	-1.37855400	3.02544500	4.39306300
H	0.26859500	3.32352500	4.97072300
C	0.43118700	-4.11825700	-0.92631700
H	0.41243600	-5.05728900	-1.50684300
H	0.86672400	-4.34015500	0.06002300
H	1.10910900	-3.42545100	-1.45143100

2b

Bi	0.05233400	-1.02123200	0.86012900
P	-1.41775600	-0.72302600	-1.38015400
P	0.53073200	1.59777100	1.36001900
P	2.44302800	-1.56162700	-0.30327900
C	-1.95014400	1.37174200	2.96371400
H	-2.56991100	1.84129100	2.18972600
H	-2.42324200	1.58441800	3.93970500
H	-1.99184600	0.28083500	2.81601400
C	-0.22834800	2.60308400	-0.06056900
H	0.06822800	2.00129400	-0.93667800
C	-0.50242600	1.89442100	2.96118100
C	0.48877200	3.96620900	-0.19296300
H	0.22442900	4.60877800	0.66523900
H	1.58099600	3.82639500	-0.14699200
C	-1.74917800	2.79942700	-0.15675300
H	-2.26824300	1.83744900	-0.07862700
H	-2.10050100	3.41875100	0.68589500
C	3.01224600	0.13598300	-0.94573700
H	2.81850600	0.89111900	-0.16391900
C	-3.10593000	-0.68767800	-0.49017200
H	-2.89908100	0.02755200	0.32717000
C	-0.50075000	3.41015700	3.24182500
H	0.51999000	3.82445400	3.23314000
H	-0.93455300	3.60555300	4.23879000
H	-1.10127800	3.96747300	2.50840700
C	2.78197300	-2.95231900	2.13255700
H	1.90932600	-2.52553900	2.65090900
H	3.46381100	-3.33751800	2.91060300
H	2.43719600	-3.81364400	1.53633800
C	3.51930800	-1.91183700	1.26623800
C	0.09677900	4.67731200	-1.49741800
H	0.59612300	5.65953000	-1.55571100
H	0.46903900	4.08474000	-2.35231100
C	-2.52004400	-2.53023500	-3.22908000
H	-3.43551800	-2.71980700	-2.65066200
H	-2.38492900	-3.38215300	-3.91875600
H	-2.68104700	-1.62878900	-3.84196300
C	2.18512800	0.49990100	-2.19653700
H	2.35319200	-0.26770700	-2.97288500
H	1.10663300	0.47761500	-1.97999100
C	-1.27527400	-2.39616400	-2.33026100
C	0.28308600	1.18749100	4.08511000
H	0.31998200	0.09488200	3.93962700
H	-0.20509500	1.37030400	5.05855700
H	1.31927800	1.55507500	4.14965000
C	3.82941600	-0.66970900	2.11544300
H	4.41833700	0.07781300	1.56502500
H	4.41623500	-0.95972600	3.00577400
H	2.91353800	-0.16860900	2.46387100
C	-1.42270200	4.83864100	-1.62415200
H	-1.68046300	5.30668400	-2.58922600
H	-1.78148200	5.52717900	-0.83621300
C	-2.13561600	3.49015900	-1.47213000
H	-1.86175900	2.82700100	-2.31261800

H	-3.23020900	3.62267700	-1.51985200
C	-5.44631800	0.30066900	-0.56545000
H	-5.20099600	1.05938300	0.20116500
H	-6.21011700	0.75366600	-1.22005500
C	-4.17995800	-0.01611300	-1.37402600
H	-4.44178000	-0.68342700	-2.21256700
H	-3.77514200	0.90444500	-1.82465200
C	4.51258700	0.18978900	-1.30114800
H	5.13295900	-0.01096800	-0.41712600
H	4.74310800	-0.60365500	-2.03615400
C	2.57367900	1.87129800	-2.76195400
H	2.34218700	2.64890600	-2.01404200
H	1.95972400	2.09834000	-3.64998400
C	-3.66923100	-1.95068200	0.18449000
H	-3.91879700	-2.70487800	-0.58032500
H	-2.91345200	-2.40883500	0.84282400
C	-4.93539800	-1.62636900	0.99300400
H	-5.33634100	-2.54632900	1.45148200
H	-4.66575700	-0.95241600	1.82747000
C	-1.11410800	-3.66050300	-1.46945300
H	-1.95182600	-3.81344700	-0.77659500
H	-0.18031400	-3.63451100	-0.88611800
H	-1.05820400	-4.54951200	-2.12362800
C	-6.00262100	-0.95074100	0.12388400
H	-6.88876800	-0.69667700	0.72968700
H	-6.34516500	-1.66555700	-0.64751900
C	4.82845000	-2.57310900	0.77941800
H	4.62239500	-3.49476000	0.21264700
H	5.45206700	-2.84401500	1.65030300
H	5.42870400	-1.91443100	0.13824400
C	4.90997400	1.55694400	-1.88063100
H	4.77619500	2.32710300	-1.09857500
H	5.98331400	1.55415000	-2.13654400
C	4.06542000	1.93180400	-3.10189800
H	4.28011200	1.22377000	-3.92379300
H	4.34151600	2.93446600	-3.47005600
C	-0.03195500	-2.25507600	-3.23091100
H	0.05418800	-3.14173000	-3.88362600
H	0.89295100	-2.19213500	-2.63876100
H	-0.09309200	-1.36377100	-3.87500300

2c

Bi	-0.2411960	0.0306860	-0.5334160
P	1.6336950	-1.2220190	1.0241090
P	0.2464770	2.5704140	0.3135660
P	-2.7738650	0.2654620	0.3915520
C	0.8571450	-2.7566590	1.8898790
C	2.5962840	-1.8504190	-0.5041100
C	4.0711540	-2.1396690	-0.1463320
C	2.0160770	-2.9702720	-1.3865600
C	4.9182440	-2.3595780	-1.4080980
C	2.8710530	-3.1847730	-2.6453960
C	4.3355760	-3.4691530	-2.2913500
C	-3.5734420	-1.3966770	-0.1170310
C	-3.2016830	-1.8391490	-1.5488760
C	-5.1174910	-1.3156900	-0.0221110
C	-3.7876450	-0.9245890	-2.6331010
C	-5.7357810	-0.4044380	-1.0920700
C	-5.3137310	-0.8279470	-2.5053550
C	-0.2039980	3.6201040	-1.2439260
C	-0.1086280	5.0922230	-0.7886110
C	-1.6674960	3.3200000	-1.6147540
C	0.4322630	-2.2468110	3.2810530
C	-0.3524170	-3.3790710	1.1746160
C	1.9489340	-3.8254400	2.0941790
H	1.2986470	-1.8875500	3.8578950
H	-0.2876950	-1.4211540	3.2155630
H	-0.0466130	-3.0604910	3.8536440
H	-0.0953580	-3.7672840	0.1797930
H	-0.7524120	-4.2216940	1.7676050
H	-1.1641060	-2.6485340	1.0507690
H	1.5596620	-4.6212000	2.7537860
H	2.2547480	-4.3020930	1.1529490
H	2.8466540	-3.4064780	2.5757880
H	2.6192890	-0.9437330	-1.1375740
H	0.9784990	-2.7361030	-1.6762680
H	1.9775140	-3.9147530	-0.8198430

H	4.1317090	-3.0375790	0.4913550
H	4.4817430	-1.3073320	0.4486560
H	2.4527670	-4.0079460	-3.2493720
H	2.8188980	-2.2770020	-3.2747390
H	4.9540950	-1.4165060	-1.9849410
H	5.9592290	-2.5943690	-1.1282490
H	4.3959100	-4.4314000	-1.7492230
H	4.9370720	-3.5891250	-3.2081620
H	-3.2233840	-2.1737640	0.5779810
H	-3.6043410	-2.8594350	-1.6937460
H	-2.1094970	-1.9314600	-1.6672660
H	-5.4395130	-0.9984620	0.9809290
H	-5.5112810	-2.3415600	-0.1543950
H	-3.3472370	0.0843910	-2.5451050
H	-3.5091270	-1.3009870	-3.6318630
H	-6.8350680	-0.4168240	-1.0016630
H	-5.4149350	0.6362110	-0.9122450
H	-5.7548540	-1.8167660	-2.7326900
H	-5.7189600	-0.1260460	-3.2534970
H	0.9159050	5.3740500	-0.4970050
H	-0.4068260	5.7593530	-1.6170970
H	-1.7874790	2.3181250	-2.0567420
H	-2.0196140	4.0514700	-2.3633230
C	-2.9828230	0.3059030	2.3152050
C	-1.7842740	1.0380700	2.9404800
C	-4.2455120	1.1600850	2.5745350
C	-3.1590950	-1.0701680	2.9734040
H	-0.8449820	0.4796670	2.8200430
H	-1.6264970	2.0292090	2.4913160
H	-1.9596990	1.1755570	4.0221590
H	-5.1537700	0.7129140	2.1432990
H	-4.4089040	1.2574290	3.6629250
H	-4.1377150	2.1723900	2.1556960
H	-3.2125820	-0.9489430	4.0697790
H	-4.0864350	-1.5705230	2.6586480
H	-2.3210620	-1.7453630	2.7589510
C	0.6881240	3.4138820	-2.4766650
H	1.7305410	3.7100550	-2.2866940
H	0.6840810	2.3664710	-2.8177220
H	0.3207880	4.0357860	-3.3130590
H	-0.7728460	5.2948380	0.0659670
H	-2.3365320	3.3731640	-0.7420460
C	2.1328780	2.7610400	0.4285090
C	2.5593410	2.6415280	1.9076980
C	3.0408790	1.8763310	-0.4435560
H	2.2903590	3.8125370	0.1209920
C	4.0450760	2.9791210	2.0988210
H	2.3724910	1.6070830	2.2473200
H	1.9350520	3.2988730	2.5347370
C	4.5248630	2.2279160	-0.2670070
H	2.9044340	0.8330000	-0.1252240
H	2.7571780	1.9290050	-1.5042100
C	4.9443630	2.1184210	1.2034390
H	4.3257990	2.8550210	3.1585920
H	4.2076340	4.0460130	1.8551060
H	5.1438040	1.5640110	-0.8952520
H	4.7043440	3.2584170	-0.6267940
H	6.0023340	2.4056140	1.3271500
H	4.8633940	1.0622860	1.5203150

2d

Bi	0.14921700	0.05360200	-0.38033700
P	2.47995400	-0.36998400	0.99199000
P	-0.86163300	2.30368600	0.79865800
P	-2.10788200	-1.15709400	0.47052500
C	2.62511400	-2.24934500	1.39387400
C	3.53794600	-0.02147900	-0.56057700
C	4.98703200	0.35169500	-0.17489600
C	3.52698200	-1.00764100	-1.74337200
C	5.76185300	0.90419400	-1.38044500
C	4.30596500	-0.44947400	-2.94438700
C	5.74214600	-0.07512200	-2.55991000
C	-2.03995900	-2.88550700	-0.34237000
C	-1.54657200	-2.85757300	-1.80509900
C	-3.43809000	-3.55229300	-0.31254200
C	-2.52685300	-2.16542700	-2.76160000
C	-4.44272800	-2.87566500	-1.25643200

C	-3.91382800	-2.81840300	-2.69576600
C	-1.29120200	3.41332300	-0.71251900
C	-2.51324600	4.29324700	-0.34317800
C	-1.58163000	2.70816800	-2.05360900
C	-3.83122900	3.50646500	-0.33411000
C	-2.89532100	1.91494300	-2.05723700
C	-4.08200300	2.80692100	-1.67509500
C	2.09586100	-2.37732500	2.83588800
C	1.83414600	-3.18987700	0.47054700
C	4.11189400	-2.65461900	1.39863300
H	2.71227200	-1.80007700	3.54242100
H	1.06248700	-2.01842500	2.92779500
H	2.11009700	-3.43470700	3.15377800
H	2.16244400	-3.12298500	-0.57587200
H	1.96594400	-4.23843900	0.79421000
H	0.75731300	-2.97041400	0.50130000
H	4.20979500	-3.67264900	1.81569700
H	4.54741100	-2.67170500	0.39023200
H	4.71816100	-1.98120600	2.02486500
C	0.60697300	3.27892100	1.57972000
C	0.96361000	2.55646400	2.89239300
C	1.85494900	3.40053300	0.69230800
C	0.09710300	4.69327500	1.92665200
H	0.09143500	2.48781100	3.56181200
H	1.34778300	1.54256100	2.71138100
H	1.75392700	3.11653100	3.42304500
H	1.62601700	3.83167900	-0.29538500
H	2.60032500	4.05945500	1.17323600
H	2.33871300	2.42580000	0.54316400
H	0.86827300	5.22298300	2.51318700
H	-0.10389400	5.30126100	1.03162200
H	-0.82002700	4.66015500	2.53619500
H	3.07828500	0.91617400	-0.93032800
H	2.49299800	-1.24054400	-2.04609600
H	3.98525900	-1.96209500	-1.43681800
H	5.51059800	-0.53881800	0.21114300
H	4.97824200	1.08776200	0.64544300
H	4.30499300	-1.18354000	-3.76809900
H	3.78530100	0.44875800	-3.32510200
H	5.30443300	1.86060100	-1.69517800
H	6.79996200	1.13578800	-1.08747400
H	6.29136200	-0.99273100	-2.27737700
H	6.27400700	0.35279600	-3.42636600
H	-1.34406600	-3.51116500	0.23495300
H	-1.42094500	-3.90782500	-2.12978900
H	-0.54526300	-2.40202000	-1.87804500
H	-3.84095600	-3.58648700	0.71112500
H	-3.30984800	-4.60717000	-0.62200100
H	-2.61142100	-1.09651300	-2.50057600
H	-2.13376200	-2.20357400	-3.79161700
H	-5.40488400	-3.41413000	-1.22376800
H	-4.64148500	-1.85008800	-0.90025000
H	-3.84492700	-3.84750500	-3.09612900
H	-4.62413100	-2.27813200	-3.34400800
H	-0.42006800	4.06945400	-0.86707500
H	-2.36484200	4.78698700	0.62894100
H	-2.58425600	5.10433300	-1.09362700
H	-0.73719100	2.06601500	-2.35278900
H	-1.64875000	3.49443800	-2.82973300
H	-2.83625200	1.07822200	-1.33955800
H	-3.05459600	1.46235000	-3.05073500
H	-5.00972900	2.21183600	-1.63091100
H	-4.23383000	3.56977400	-2.46212100
H	-3.79163100	2.74877200	0.46821600
H	-4.66733900	4.18331800	-0.08969400
C	-2.19257100	-1.53970000	2.36605400
C	-1.42299500	-0.45634200	3.14005200
C	-3.68739700	-1.42186800	2.74244100
C	-1.67808100	-2.93118100	2.76195900
H	-0.34056800	-0.50074000	2.95261600
H	-1.76171000	0.55451400	2.86906600
H	-1.58246700	-0.59211500	4.22436400
H	-4.31326000	-2.15612900	2.21271200
H	-3.81251900	-1.59959700	3.82575200
H	-4.07932900	-0.41883800	2.51434300
H	-1.70646000	-3.03436900	3.86110800
H	-2.29535700	-3.74060800	2.34563300
H	-0.64085100	-3.09874600	2.44329900

TS1

Bi	0.78033500	-1.42157900	-0.40309600
P	1.10603600	-0.26148700	2.04282100
P	1.99381300	0.55422100	-1.86589600
C	0.09089100	-2.90444000	2.91531600
H	-0.93356200	-2.53053800	3.03365100
H	0.22639700	-3.74067700	3.62526300
H	0.17840600	-3.32557800	1.90127600
C	-0.47669200	0.71850200	2.41233700
H	-0.60107100	1.28328700	1.47277200
C	1.15120900	-1.82093800	3.18094100
C	-0.21849200	1.76999800	3.51546200
H	-0.05930400	1.26317900	4.48231900
H	0.71147000	2.32145200	3.29970700
C	-1.79904300	-0.02400000	2.66151800
H	-1.99266000	-0.73361900	1.84192700
H	-1.72725900	-0.60737100	3.59494000
C	1.90515300	2.08234800	-0.73702600
H	2.16441200	1.78287300	0.29315800
C	-2.57491800	0.52814600	-1.21603300
H	-2.02667600	1.13130400	-0.47401800
C	1.04617800	-1.35082900	4.64452700
H	1.77019900	-0.55178500	4.87052100
H	1.25646700	-2.19684400	5.32331200
H	0.03977700	-0.97974500	4.88675900
C	3.88053000	-1.54045200	-2.13207300
H	3.46935300	-2.20299400	-1.35417000
H	4.92904400	-1.84731300	-2.29076300
H	3.33164500	-1.73321600	-3.06881000
C	3.82994700	-0.05244500	-1.73086500
C	-1.39866800	2.74351700	3.64999000
H	-1.20291000	3.46350600	4.46278000
H	-1.48350600	3.33550800	2.72025900
C	-3.38086000	-2.41229000	-2.95644100
H	-4.08985100	-1.57298500	-2.90381400
H	-3.95673600	-3.30988200	-3.24716100
H	-2.65358000	-2.19331200	-3.75226400
C	0.45481000	2.59843900	-0.74560100
H	0.16697100	2.85415800	-1.78086800
H	-0.22983800	1.80038000	-0.43007500
C	-2.67598900	-2.66067700	-1.60940200
C	2.55148800	-2.43060000	2.96133200
H	2.69209500	-2.78216800	1.92505700
H	2.69078900	-3.30444600	3.62191200
H	3.35027600	-1.70515600	3.18199700
C	4.45917400	0.12194200	-0.33996500
H	4.52507700	1.17759600	-0.04006400
H	5.48690900	-0.28418600	-0.33757500
H	3.88901100	-0.40157700	0.44313200
C	-2.71685700	1.99966200	3.89318200
H	-3.55674100	2.71253400	3.95283400
H	-2.67001200	1.48715300	4.87223600
C	-2.96822200	0.96349100	2.79273400
H	-3.10212500	1.48986700	1.83177300
H	-3.90721000	0.41490700	2.97947300
C	-3.02822600	2.64422300	-2.54797500
H	-2.43381900	3.26584700	-1.85223500
H	-2.93968000	3.11677600	-3.54080500
C	-2.43471400	1.22885500	-2.58198400
H	-2.95365800	0.62691900	-3.34894100
H	-1.37445100	1.25612100	-2.87579000
C	2.84879200	3.22757100	-1.15822200
H	3.89923100	2.91034500	-1.10438700
H	2.65279900	3.49366500	-2.21353000
C	0.26432800	3.82833300	0.14832000
H	0.46498400	3.54859400	1.19746200
H	-0.78697900	4.16218700	0.10831800
C	-4.04376800	0.50775000	-0.75320400
H	-4.64449400	-0.11044500	-1.44159000
H	-4.12345500	0.04456100	0.24168500
C	-4.63181400	1.92791300	-0.72930800
H	-5.69053700	1.88902000	-0.42118500
H	-4.10564000	2.52247000	0.04022700
C	-3.71227400	-3.01940500	-0.52644800
H	-4.45149600	-2.21988400	-0.38125800
H	-3.22254800	-3.20662800	0.44103100

H	-4.26322500	-3.93292400	-0.81763200
C	-4.48987600	2.62801300	-2.08584600
H	-4.89180200	3.65387200	-2.03192700
H	-5.10026500	2.09121800	-2.83586800
C	4.64151800	0.72888300	-2.78858300
H	4.23670900	0.56815500	-3.80015900
H	5.68967800	0.37876500	-2.78423500
H	4.65625500	1.81098900	-2.60344200
C	2.66237900	4.47030000	-0.27223300
H	2.96581900	4.21956800	0.76098900
H	3.33915300	5.27316300	-0.61097600
C	1.21079100	4.95989100	-0.26243600
H	0.93836400	5.31093600	-1.27502000
H	1.09897600	5.82580000	0.41171500
C	-1.69893500	-3.83795200	-1.76720700
H	-2.26226600	-4.74054600	-2.06144200
H	-1.17936300	-4.07085900	-0.82375800
H	-0.94264900	-3.64216200	-2.54284300
P	-1.66756900	-1.11348800	-1.13184300

TS2’ [for inverse sequence: rotation/inversion; not inversion/rotation]

Bi	-0.09660900	-0.87705800	-0.81577400
P	-0.79374900	1.76627700	-1.02055500
P	-2.20144300	-1.65374600	0.74843600
C	0.89057600	1.59689600	-3.46708200
H	1.77563300	2.03073000	-2.98627700
H	0.94427300	1.84306800	-4.54334600
H	0.96388200	0.50089500	-3.38327900
C	0.50610300	2.61669200	0.06653700
H	0.56175300	1.92367100	0.92693000
C	-0.42626000	2.13180100	-2.87772400
C	-0.03345900	3.96171300	0.59893000
H	-0.12483600	4.67944000	-0.23614200
H	-1.04963600	3.82655100	1.00323500
C	1.94545100	2.76823500	-0.44738300
H	2.33453300	1.79064300	-0.76515500
H	1.96082700	3.43078300	-1.32918000
C	-3.31093100	-0.10650100	0.72305800
H	-3.26773400	0.35888500	-0.27641200
C	-0.49460300	3.65981300	-3.06778300
H	-1.42747400	4.07813400	-2.65721500
H	-0.45823000	3.90733600	-4.14421100
H	0.34937600	4.17305400	-2.58357900
C	-1.93562700	-3.95202400	-0.85976700
H	-1.16153500	-3.54416800	-1.52838500
H	-2.40896600	-4.79586900	-1.39121500
H	-1.43180400	-4.35722800	0.03292600
C	-3.00669700	-2.90708700	-0.48541800
C	0.89310000	4.55010000	1.67284300
H	0.50245100	5.52196100	2.01970800
H	0.89214200	3.87941800	2.55249100
C	-2.73893800	0.90397900	1.73866000
H	-2.74501200	0.44502300	2.74407300
H	-1.68810300	1.12711100	1.50795200
C	-1.60423000	1.48641000	-3.63749700
H	-1.60329800	0.38666300	-3.54371700
H	-1.53459300	1.71994700	-4.71451400
H	-2.57561000	1.85350500	-3.27042700
C	-3.57387500	-2.26838100	-1.76234700
H	-4.36478200	-1.53485200	-1.54928200
H	-4.01023000	-3.04487900	-2.41618200
H	-2.79295600	-1.75332700	-2.34616000
C	2.32946800	4.69923700	1.15771800
H	2.98664800	5.09142800	1.95238900
H	2.34502000	5.44563600	0.34146500
C	2.86407100	3.36370200	0.62908400
H	2.93915000	2.64662000	1.46531700
H	3.88531400	3.48338100	0.22877100
C	-4.79197800	-0.37643200	1.05804600
H	-5.24589800	-1.05028800	0.31850200
H	-4.86277500	-0.88654000	2.03657100
C	-3.54251200	2.20857000	1.76769800
H	-3.45004800	2.70013600	0.78316400
H	-3.10551000	2.90023900	2.50826700
C	-4.11941500	-3.65711500	0.28103600
H	-3.71952300	-4.14623900	1.18315000
H	-4.54783800	-4.44328400	-0.36624200

H	-4.94397300	-3.00343100	0.59228200
C	-5.60610000	0.92728600	1.09859200
H	-5.61904000	1.36933600	0.08525900
H	-6.65530200	0.70121900	1.35504400
C	-5.01844400	1.94505300	2.08065700
H	-5.10783200	1.54899200	3.10946200
H	-5.59685200	2.88412300	2.05444500
P	1.71182500	-0.98237400	1.00826600
C	1.64433400	-0.81496900	2.90608600
C	0.29121100	-0.21245700	3.30205600
C	2.77929300	0.10315800	3.39324000
C	1.79206800	-2.21168200	3.53890800
H	0.16981700	0.80667000	2.90563200
H	0.22303600	-0.15505100	4.40240800
H	-0.54743000	-0.82700100	2.94418700
H	2.68221200	1.11598100	2.97619100
H	3.77485500	-0.28288700	3.12449300
H	2.74501400	0.18079900	4.49501500
H	1.78590500	-2.13125500	4.64174500
H	2.73911600	-2.69434700	3.24871400
H	0.96670600	-2.87097400	3.23318200
C	3.45566500	-1.39673700	0.46346900
C	3.50171700	-2.49655900	-0.62611000
C	4.32990200	-0.20238600	0.04269200
H	3.89121800	-1.81652200	1.38767700
C	4.85927300	-2.53338500	-1.36710000
H	2.69860100	-2.33312700	-1.36456200
H	3.28504400	-3.46909000	-0.15514700
C	5.82096000	-0.60687000	-0.03373800
H	3.97934700	0.15120900	-0.94246600
H	4.19558700	0.64317000	0.73170300
C	6.00454100	-2.08220000	-0.45577400
H	4.82389500	-1.86641000	-2.24658400
H	5.04359000	-3.54621700	-1.76101400
H	6.30237500	-0.44105200	0.94507200
H	6.33950400	0.05718400	-0.74602400
H	6.02191000	-2.73075000	0.43799400
H	6.97913700	-2.22188900	-0.95114100

TS2T-shape

Bi	0.04185500	-1.01327200	-0.39778500
P	0.01749400	1.69318900	-0.87352700
P	-2.46634300	-1.15188600	0.65931500
P	1.67901900	-1.22888300	1.70403100
C	-3.06415600	-2.17695500	-2.05986900
H	-3.46517900	-1.22013100	-2.41560500
H	-3.56276800	-2.97919400	-2.63369600
H	-1.99358700	-2.21312800	-2.31636100
C	-3.20739100	0.57267600	0.39701600
H	-2.36413200	1.22828200	0.67942300
C	-3.29848500	-2.39437800	-0.55516300
C	-4.33443500	0.83764300	1.41943100
H	-5.21129100	0.21013200	1.17870600
H	-4.00775600	0.53946000	2.42910900
C	-3.63806400	1.01955700	-1.00623100
H	-2.82497300	0.84994500	-1.72287000
H	-4.49693700	0.41522800	-1.34466900
C	3.21692600	-1.69414900	0.67881200
H	4.04626400	-1.84985100	1.39462400
C	1.41359200	2.38233300	0.23718000
H	1.68740000	1.53280400	0.89224000
C	-4.81045700	-2.37823700	-0.25791400
H	-5.01576200	-2.52446800	0.81461500
H	-5.30783000	-3.19512600	-0.81105300
H	-5.28276500	-1.43532500	-0.56995100
C	-0.40131600	-2.00791000	3.45853800
H	-1.04331500	-2.21186000	2.59438700
H	-1.06909200	-1.76148100	4.30427600
H	0.16618400	-2.91516100	3.70850900
C	0.54553700	-0.82434400	3.22211800
C	-4.74928200	2.31701700	1.40924200
H	-5.57486100	2.48406700	2.12223200
H	-3.89862500	2.92811000	1.76393700
C	1.77349600	0.97386500	-3.17305000
H	2.68318200	1.34925700	-2.68759400
H	1.92008000	1.07462100	-4.26386400
H	1.68697100	-0.10217400	-2.95571400

C	3.63416800	-0.54396100	-0.24855700
H	2.79281100	-0.26632000	-0.90091500
H	3.86125800	0.34402000	0.35541900
C	0.51368500	1.74460100	-2.74465700
C	-2.73271400	-3.77427000	-0.15961700
H	-1.64793900	-3.84409300	-0.34630500
H	-3.21917900	-4.56558700	-0.75692600
H	-2.90731600	-3.99597300	0.90505400
C	-0.18913000	0.48948700	2.96614900
H	0.51092100	1.33234700	2.92934100
H	-0.89524900	0.65410400	3.79794200
H	-0.77545700	0.48901100	2.04134700
C	-5.14976500	2.78516600	0.00474500
H	-5.40027200	3.85963800	0.01438400
H	-6.06892500	2.25244100	-0.30425200
C	-4.03894300	2.50106200	-1.01328300
H	-3.14633500	3.10573400	-0.77211400
H	-4.35919700	2.80048100	-2.02638300
C	3.70302800	3.35850100	0.72808400
H	3.98188200	2.48183700	1.34145000
H	4.63590000	3.73142300	0.27122700
C	2.72366300	2.91347400	-0.37047800
H	2.51432400	3.78053500	-1.01888500
H	3.20276400	2.15617700	-1.00390300
C	3.01825000	-3.02705700	-0.06834200
H	2.80561900	-3.82622100	0.65670200
H	2.13603600	-2.96142100	-0.72690100
C	4.83489900	-0.92509300	-1.12791000
H	5.72998400	-1.04313600	-0.49030900
H	5.05159400	-0.09560900	-1.82251300
C	0.78896300	3.47819100	1.13213400
H	0.50668800	4.34047600	0.49981000
H	-0.14958300	3.11603100	1.58155600
C	1.75802400	3.94450000	2.22789400
H	1.29162900	4.74012100	2.83374800
H	1.95757300	3.10266300	2.91673200
C	0.64157900	3.22497800	-3.15430100
H	1.53796100	3.69877400	-2.73252400
H	-0.23694900	3.80747400	-2.83413900
H	0.71590700	3.30209800	-4.25408500
C	3.08574600	4.42847200	1.63501700
H	3.78957600	4.71014400	2.43658400
H	2.90428000	5.34357700	1.04100500
C	1.42693100	-0.64009100	4.48017500
H	1.99359100	-1.55394100	4.71542500
H	0.77949200	-0.41751200	5.34672900
H	2.13243400	0.19582100	4.35901900
C	4.24600700	-3.36609500	-0.92917700
H	5.11361000	-3.55779100	-0.27148500
H	4.05699900	-4.30275400	-1.48030500
C	4.58672500	-2.22676000	-1.89524700
H	3.74626800	-2.08072200	-2.59913900
H	5.46690500	-2.48796400	-2.50588500
C	-0.69664500	1.15127500	-3.49586800
H	-0.51046200	1.18391600	-4.58359400
H	-1.61610500	1.72209100	-3.29733700
H	-0.88339800	0.09835800	-3.22859300

2-inv

Bi	0.51280000	-1.10240000	0.87920000
P	0.13150000	1.50510000	1.49310000
P	2.26960000	-0.77590000	-1.18530000
C	-1.41810000	0.38110000	3.75490000
H	-2.38710000	0.60110000	3.29020000
H	-1.56590000	0.39420000	4.85000000
H	-1.13600000	-0.64740000	3.47940000
C	-1.35230000	2.20990000	0.53470000
H	-1.19820000	1.77990000	-0.46970000
C	-0.32930000	1.39570000	3.36390000
C	-1.19280000	3.74080000	0.38610000
H	-1.30140000	4.22100000	1.37410000
H	-0.17720000	3.98020000	0.03510000
C	-2.78510000	1.85540000	0.96240000
H	-2.90700000	0.76540000	1.02170000
H	-2.97880000	2.24910000	1.97350000
C	2.95360000	0.97220000	-0.84730000
H	2.98480000	1.15900000	0.24020000

C	-2.15850000	-0.82480000	-1.72210000
H	-2.54110000	-0.10060000	-0.98680000
C	-0.74360000	2.80580000	3.82980000
H	0.00860000	3.56240000	3.55550000
H	-0.84610000	2.81760000	4.92960000
H	-1.71020000	3.11700000	3.40860000
C	2.92220000	-3.39750000	-0.43610000
H	2.26240000	-3.43610000	0.44540000
H	3.68110000	-4.18840000	-0.30440000
H	2.31740000	-3.65180000	-1.32160000
C	3.61740000	-2.02860000	-0.59080000
C	-2.23780000	4.32520000	-0.57490000
H	-2.11720000	5.41970000	-0.64220000
H	-2.05410000	3.92770000	-1.59010000
C	-3.39180000	-4.04650000	-0.41310000
H	-3.98160000	-3.60070000	-1.22460000
H	-4.09670000	-4.56840000	0.25870000
H	-2.72770000	-4.80350000	-0.85890000
C	1.96980000	1.98110000	-1.47770000
H	1.90360000	1.78320000	-2.56330000
H	0.95840000	1.83470000	-1.07520000
C	-2.58710000	-3.00080000	0.39210000
C	0.98400000	1.01650000	4.08010000
H	1.33250000	0.00870000	3.80000000
H	0.82830000	1.01600000	5.17320000
H	1.79150000	1.72930000	3.85070000
C	4.31830000	-1.65550000	0.72400000
H	4.84740000	-0.69370000	0.65880000
H	5.06500000	-2.42590000	0.98920000
H	3.60500000	-1.58860000	1.56120000
C	-3.66420000	3.96620000	-0.14330000
H	-4.39850000	4.36520000	-0.86320000
H	-3.88120000	4.45060000	0.82710000
C	-3.82820000	2.44970000	0.00360000
H	-3.71900000	1.97680000	-0.98780000
H	-4.84400000	2.20090000	0.35520000
C	-1.76720000	1.01310000	-3.46090000
H	-2.11870000	1.81300000	-2.78450000
H	-0.99920000	1.47070000	-4.10710000
C	-1.13700000	-0.11160000	-2.63130000
H	-0.67870000	-0.85330000	-3.30910000
H	-0.30680000	0.29110000	-2.03630000
C	4.36400000	1.23110000	-1.41440000
H	5.10260000	0.56120000	-0.95360000
H	4.37040000	1.01140000	-2.49800000
C	2.40020000	3.43370000	-1.24500000
H	2.36920000	3.64130000	-0.16050000
H	1.67640000	4.11920000	-1.71850000
C	-3.35120000	-1.31870000	-2.56760000
H	-3.00860000	-2.12630000	-3.24050000
H	-4.12830000	-1.75480000	-1.92410000
C	-3.97770000	-0.19000000	-3.40170000
H	-4.80660000	-0.59430000	-4.00720000
H	-4.42630000	0.55480000	-2.71910000
C	-3.53450000	-1.98160000	1.03820000
H	-4.10570000	-1.40590000	0.29500000
H	-2.98340000	-1.26880000	1.66960000
H	-4.26570000	-2.49860000	1.68560000
C	-2.94610000	0.50460000	-4.29510000
H	-3.41380000	1.33180000	-4.85540000
H	-2.57390000	-0.21530000	-5.04730000
C	4.65130000	-2.16960000	-1.73000000
H	4.16480000	-2.45830000	-2.67500000
H	5.38040000	-2.95850000	-1.47180000
H	5.21780000	-1.24740000	-1.91220000
C	4.80610000	2.68570000	-1.18650000
H	4.89480000	2.86480000	-0.09900000
H	5.81260000	2.83930000	-1.61200000
C	3.81250000	3.68870000	-1.78030000
H	3.80760000	3.58470000	-2.88130000
H	4.13090000	4.72260000	-1.56380000
C	-1.83300000	-3.76370000	1.49950000
H	-2.54390000	-4.38040000	2.07660000
H	-1.33780000	-3.08660000	2.21270000
H	-1.06740000	-4.43970000	1.08400000
P	-1.27600000	-2.24100000	-0.80910000

2-rot [for inverse sequence: rotation/inversion; not inversion/rotation]

Bi	-0.10854200	-1.11154500	0.72572900
P	-1.82753400	-1.67903800	-1.27129100
P	0.73370300	1.36782600	1.48636500
P	2.14539600	-1.93292500	-0.56078300
C	-1.88550200	1.43314400	2.86297600
H	-2.32950600	2.10898900	2.12156600
H	-2.40645700	1.60676100	3.82211300
H	-2.11028400	0.39993800	2.55542500
C	0.36341700	2.72472800	0.21504900
H	0.69734100	2.23942500	-0.71697700
C	-0.37421000	1.66359600	3.04128100
C	1.30043800	3.93737800	0.42543300
H	1.02280400	4.46647500	1.35242600
H	2.33810500	3.59436600	0.56600800
C	-1.07863400	3.19910900	-0.01553800
H	-1.73803500	2.33916800	-0.18962700
H	-1.45205800	3.70727200	0.88904600
C	3.14636200	-0.32199300	-0.74356100
H	3.04352900	0.27603400	0.17818100
C	-3.44320800	-1.19689700	-0.39660000
H	-3.24428300	-1.56943900	0.62804600
C	-0.13254200	3.10342700	3.53569200
H	0.94077400	3.32043300	3.65489000
H	-0.61373500	3.24147100	4.52039500
H	-0.56091200	3.85366300	2.85581500
C	1.97637400	-3.95145000	1.40319100
H	1.14971400	-3.49715900	1.97048800
H	2.48830800	-4.65578400	2.08182000
H	1.54052900	-4.53892500	0.57818300
C	2.98438600	-2.90413000	0.88820500
C	1.21444700	4.91613500	-0.75505800
H	1.87131100	5.78378500	-0.57363900
H	1.59792700	4.41664700	-1.66333800
C	-1.37754100	0.85471000	-2.68473000
H	-2.23090800	1.36925200	-2.22632400
H	-1.19006500	1.33368200	-3.66285500
H	-0.49908900	1.03527500	-2.05275300
C	2.54478400	0.47870200	-1.91561200
H	2.61089600	-0.12846900	-2.83634500
H	1.47305200	0.65615200	-1.74769100
C	-1.63526400	-0.64519500	-2.87675600
C	0.15791600	0.68941300	4.11351400
H	0.00587000	-0.36454900	3.82888900
H	-0.37742500	0.85106100	5.06566900
H	1.23360100	0.83790200	4.29758800
C	3.45300300	-2.02274000	2.05578600
H	4.21346500	-1.29006700	1.74905300
H	3.89983800	-2.64961300	2.84856400
H	2.61972700	-1.46084900	2.50695600
C	-0.22722200	5.37579200	-1.00219500
H	-0.27354100	6.04760700	-1.87583100
H	-0.57264400	5.96703100	-0.13356500
C	-1.16251600	4.17689600	-1.19718900
H	-0.88365300	3.64504400	-2.12439500
H	-2.20399200	4.51540800	-1.33262500
C	-5.05908700	0.47131500	0.63061400
H	-4.80494900	0.15630900	1.65974900
H	-5.32522300	1.54057900	0.68930700
C	-3.82662600	0.28504400	-0.26567000
H	-4.04684500	0.69963900	-1.26400200
H	-2.98102800	0.86067900	0.13557200
C	4.65057100	-0.53971000	-1.00534200
H	5.12441300	-1.05284800	-0.15775100
H	4.77977200	-1.19613400	-1.88585600
C	3.26682400	1.81136700	-2.14481000
H	3.13724400	2.45196400	-1.25580500
H	2.80419900	2.34955400	-2.98969700
C	-4.64216800	-2.02251900	-0.91651800
H	-4.89371500	-1.70773600	-1.94323800
H	-4.36948400	-3.08866100	-0.97479600
C	-5.87552900	-1.83391500	-0.01988400
H	-6.72648800	-2.40828900	-0.42373300
H	-5.66109900	-2.25370300	0.98049600
C	-2.89943400	-0.83884000	-3.73720100
H	-3.77423400	-0.32742900	-3.30914600
H	-3.15129100	-1.90321600	-3.86762900

H	-2.72747400	-0.41071800	-4.74074200
C	-6.24902900	-0.35360500	0.12598900
H	-7.11336500	-0.23859600	0.80158200
H	-6.56691900	0.03760100	-0.85858600
C	4.17540600	-3.68533900	0.28836900
H	3.84613500	-4.34738800	-0.52792800
H	4.63262100	-4.31734400	1.07074900
H	4.96428900	-3.03335100	-0.10742500
C	5.38371200	0.79017400	-1.24557200
H	5.34180400	1.39209600	-0.31903700
H	6.45139600	0.59469400	-1.44361400
C	4.76220500	1.59318400	-2.39203100
H	4.89723100	1.03940900	-3.33959600
H	5.28168200	2.55855500	-2.51476900
C	-0.43666600	-1.28607800	-3.60531600
H	-0.24462300	-0.75272100	-4.55299800
H	-0.62897300	-2.34410600	-3.84205200
H	0.48220400	-1.24679500	-3.00314300

2a-Birad

Bi	0.19438200	-1.67387600	-0.36433000
P	1.92897400	0.26484900	-0.97038300
P	-1.55981900	0.00816700	0.82012700
C	-2.53849200	0.62998800	-0.69692900
H	-2.84310700	-0.23616800	-1.31046200
C	1.67654100	1.55997300	0.38741700
H	0.57619500	1.64856300	0.37454700
C	-1.86744300	-2.18007100	2.56931200
H	-1.30255800	-2.89870500	1.95425100
H	-2.50963900	-2.76783500	3.24831300
H	-1.14587400	-1.62400200	3.19025800
C	-2.73753400	-1.23395700	1.71868800
C	3.86953500	-1.42971900	0.47960700
H	3.73545800	-0.86590900	1.41167800
H	4.89627600	-1.83907700	0.48133600
H	3.17546100	-2.28434800	0.50098000
C	-1.63481600	1.53784400	-1.55817200
H	-1.28520200	2.38502600	-0.93931300
H	-0.73073700	1.00102900	-1.88594400
C	3.66269300	-0.55673200	-0.76977800
C	-3.62684400	-2.05976800	0.77760800
H	-4.31401700	-1.43091700	0.19259100
H	-4.24576100	-2.76367300	1.36252500
H	-3.02932900	-2.65455200	0.06805800
C	1.57004000	2.32733100	2.79588600
H	0.46520500	2.33299600	2.78444700
H	1.87463200	2.09018300	3.82970300
C	2.06219400	1.23169500	1.83802300
H	3.15941900	1.14624400	1.92384200
H	1.63992600	0.25747000	2.13188700
C	-3.81171500	1.40544000	-0.29870000
H	-4.50236900	0.76021600	0.26210500
H	-3.53278000	2.23259000	0.38007800
C	-2.37101500	2.09116100	-2.78530200
H	-2.64509600	1.25237000	-3.45154700
H	-1.69285200	2.74071800	-3.36396400
C	2.22019900	2.94372200	-0.02925600
H	3.32351800	2.93360800	-0.02991400
H	1.90926000	3.17609100	-1.06101800
C	1.72922200	4.03510500	0.93408900
H	2.14591100	5.01377000	0.64061800
H	0.63028000	4.12209300	0.84705600
C	4.71768800	0.56615400	-0.78659000
H	4.69069600	1.16729700	0.13416200
H	4.58347000	1.24470300	-1.64403000
H	5.72753700	0.12501400	-0.86178500
C	2.09363600	3.70956400	2.38762700
H	1.70150200	4.48627000	3.06591500
H	3.19461500	3.72680700	2.49568200
C	-3.60986100	-0.41333900	2.69507700
H	-2.98755900	0.15441000	3.40445100
H	-4.25351000	-1.09483700	3.27979100
H	-4.26802000	0.30116200	2.18285600
C	-4.54768900	1.97121900	-1.52353500
H	-4.92051500	1.12990700	-2.13680800
H	-5.43711900	2.53496300	-1.19410800
C	-3.63718800	2.85293500	-2.38273500

H	-3.35117800	3.75130900	-1.80464400
H	-4.17671600	3.21237200	-3.27525300
C	3.84364400	-1.43009100	-2.02749300
H	4.84517100	-1.89509200	-2.02174400
H	3.74710900	-0.83863300	-2.95143900
H	3.10448200	-2.24768300	-2.07096700

2a-Prad

P	0.93541900	-1.04149300	-0.56781400
C	-0.67483600	-0.35017500	0.12311500
H	-0.53864200	-0.21899700	1.21279400
C	3.57447400	-0.74119300	0.19233300
H	3.46443600	-1.55393400	0.92845400
H	4.43437400	-0.12037900	0.50057200
H	3.82384700	-1.19876500	-0.77917900
C	2.29980200	0.12466400	0.10454700
C	-1.79572400	-1.39153300	-0.08654700
H	-1.90804200	-1.58832400	-1.16925200
H	-1.51155200	-2.35248600	0.37426100
C	1.98952200	0.70744900	1.49225400
H	1.12999600	1.39418900	1.47508700
H	2.85890300	1.28125500	1.85989700
H	1.77993700	-0.08331300	2.23093900
C	-1.09386500	1.01300400	-0.47016400
H	-0.32394000	1.77443900	-0.27333600
H	-1.16646500	0.91951700	-1.56944800
C	-3.13871900	-0.90938200	0.47845500
H	-3.05513000	-0.81238800	1.57697300
H	-3.92033200	-1.66477700	0.29010900
C	2.54624500	1.26598000	-0.90491200
H	2.78693200	0.87627200	-1.90706600
H	3.40087200	1.88300200	-0.57209400
H	1.67523500	1.93085800	-1.00318200
C	-2.44270700	1.48862100	0.09088800
H	-2.33088600	1.68761400	1.17298600
H	-2.72577500	2.44764900	-0.37553300
C	-3.54464400	0.44376800	-0.11667400
H	-3.72859000	0.32206700	-1.20038500
H	-4.49374500	0.79166400	0.32483500

s1-a

Bi	0.0252510	-0.0395140	-0.8318170
P	2.2734560	-0.3696860	0.6374840
P	-1.1589960	1.8932970	0.6444620
P	-1.3288840	-2.1121070	0.3034680
C	-0.1575610	3.6552390	-1.5198910
H	0.7014020	4.0007490	-0.9317500
H	-0.4216470	4.4632500	-2.2262980
H	0.1683610	2.7926780	-2.1227800
C	0.1105930	2.4317130	1.9574460
H	0.5197060	1.4589480	2.2758760
C	-1.3750370	3.3115880	-0.6441920
C	-0.6282780	3.0339340	3.1653140
H	-1.0750600	4.0125140	2.9270510
H	-1.4367560	2.3747210	3.5171900
C	1.3038740	3.2955800	1.5460340
H	1.8559980	2.8583280	0.7052960
H	1.0003790	4.3166890	1.2683770
C	-2.0108570	-1.3592970	1.9157120
H	-2.4363060	-0.3696570	1.6836270
C	2.2456400	-2.2324190	1.0403190
H	1.2234870	-2.3398090	1.4369170
C	-1.8283070	4.5679200	0.1257600
H	-2.6939160	4.3578890	0.7741560
H	-2.1267170	5.3560000	-0.5885660
H	-1.0246580	4.9811730	0.7524260
C	-2.3223900	-2.3251510	-2.3295730
H	-1.9182270	-1.3677860	-2.6933510
H	-3.1456720	-2.6089000	-3.0080490
H	-1.5324090	-3.0883440	-2.4277760
C	-2.8501300	-2.2300680	-0.8842420
C	3.4437010	-0.6675250	-2.0766450
H	3.3491590	-1.7599480	-2.0514650
H	4.3068830	-0.4236790	-2.7223080
H	2.5469930	-0.2615140	-2.5703960
C	-0.8579280	-1.1643690	2.9117970
H	-0.4313970	-2.1316580	3.2259500

H	-0.0376620	-0.5550710	2.5063520
C	3.6557220	-0.0566030	-0.6805910
C	-2.5299690	2.8531470	-1.5584720
H	-2.2626460	1.9574520	-2.1437950
H	-2.7781220	3.6509450	-2.2804430
H	-3.4386470	2.6210240	-0.9812760
C	-3.8320390	-1.0541110	-0.7732400
H	-4.2885420	-0.9825550	0.2244520
H	-4.6532700	-1.1779360	-1.5021080
H	-3.3441860	-0.0894180	-0.9807600
C	2.3851080	-3.2582490	-0.0872310
H	3.3986580	-3.2669770	-0.5167000
H	1.6645360	-3.0808770	-0.8995300
C	-3.0986710	-2.2270950	2.5667310
H	-4.0289100	-2.2529780	1.9834990
H	-2.7580880	-3.2660870	2.7133130
C	3.2028890	-2.5389330	2.2055470
H	4.2596590	-2.4863900	1.9018310
H	3.0599670	-1.8387280	3.0430970
C	4.9810200	-0.5790800	-0.0924870
H	5.0106450	-1.6777250	-0.0573760
H	5.1568480	-0.1977040	0.9261160
H	5.8239890	-0.2481500	-0.7249580
C	-3.5706180	-3.5614420	-0.5745940
H	-2.8904450	-4.4197520	-0.6919180
H	-4.4117760	-3.6985640	-1.2776150
H	-3.9838110	-3.5982340	0.4415690
C	3.7529540	1.4747130	-0.8272350
H	4.5668630	1.7330640	-1.5272290
H	3.9663240	1.9643010	0.1351760
H	2.8251960	1.9104120	-1.2340370
H	2.1895320	-4.2709620	0.3055920
H	3.0203390	-3.5605480	2.5825230
H	-1.2250840	-0.6536830	3.8180470
H	-3.3520060	-1.8180250	3.5600990
H	0.0751110	3.1871120	4.0026900
H	2.0081750	3.3845640	2.3912780

s1-b

Bi	-0.0122600	-0.1249740	-0.8576060
P	2.0829440	-0.8847610	0.6635710
P	-0.6724970	2.1379390	0.4644940
P	-1.9023130	-1.6876540	0.3102860
C	1.2684690	3.4038290	-1.3765980
H	2.0596060	3.4371150	-0.6172770
H	1.4453630	4.2419520	-2.0750460
H	1.3884800	2.4725190	-1.9527800
C	0.4144910	2.2390820	2.0273630
H	0.4190030	1.1889160	2.3576370
C	-0.1400550	3.5195130	-0.7683760
C	-0.3304610	3.0514580	3.1017990
H	-0.3894770	4.1200460	2.8391150
H	-1.3587810	2.6864720	3.2475790
C	1.8768680	2.6793000	1.9217270
H	2.4396270	2.0822680	1.1940690
H	1.9728370	3.7402270	1.6446900
C	-2.2594960	-0.7838520	1.9511450
H	-2.3043160	0.2989360	1.7459440
C	3.4301100	-0.0256340	-0.3951500
H	2.9948550	0.9835770	-0.5058450
C	-0.2806680	4.8702800	-0.0384330
H	-1.2773440	4.9841500	0.4168850
H	-0.1427140	5.6983990	-0.7564090
H	0.4721530	4.9944980	0.7533400
C	-2.9801100	-1.5797120	-2.2920590
H	-2.2978840	-0.8050420	-2.6753510
H	-3.8671960	-1.5838810	-2.9490570
H	-2.4812400	-2.5572710	-2.3998390
C	-3.4139570	-1.3146740	-0.8365110
C	3.7044490	-3.1888060	0.6740410
H	4.3869000	-2.8417560	-0.1147570
H	3.7837330	-4.2895840	0.7150630
H	4.0631390	-2.7945620	1.6383810
C	-1.1044000	-1.0614960	2.9262950
H	-1.0948430	-2.1194480	3.2356230
H	-0.1145500	-0.8375580	2.5037890
C	2.2404140	-2.7918290	0.4017640

C	-1.1792050	3.4686470	-1.9074240
H	-1.1294490	2.5215130	-2.4701900
H	-0.9900110	4.2853440	-2.6261650
H	-2.2061310	3.5821580	-1.5259720
C	-3.9688830	0.1129060	-0.7196350
H	-4.3420240	0.3357870	0.2901220
H	-4.8132220	0.2467410	-1.4199000
H	-3.2097410	0.8728910	-0.9590880
C	4.7227990	0.1453200	0.4216560
H	5.2456870	-0.8118440	0.5675890
H	4.5204420	0.5706460	1.4166560
C	-3.5818230	-1.2086030	2.6069920
H	-4.4648590	-0.8805160	2.0425400
H	-3.6403840	-2.3031970	2.7304960
C	3.7289170	-0.5382890	-1.8090650
H	4.2121190	-1.5266250	-1.7950060
H	2.8217540	-0.6151280	-2.4279710
C	1.8017010	-3.3306240	-0.9705270
H	2.3651980	-2.8867850	-1.8018710
H	0.7281710	-3.1589910	-1.1463830
H	1.9606970	-4.4236280	-1.0080890
C	-4.5118220	-2.3464640	-0.4935580
H	-4.1415230	-3.3773430	-0.6091970
H	-5.3677200	-2.2162420	-1.1798890
H	-4.8940740	-2.2402420	0.5297050
C	1.3512410	-3.4305390	1.4877140
H	1.4606260	-4.5290590	1.4553970
H	0.2868460	-3.2022630	1.3293380
H	1.6328560	-3.0910340	2.4968880
H	5.4183380	0.8234200	-0.1032720
H	4.4215670	0.1522200	-2.3212430
H	0.1973940	2.9771350	4.0688860
H	2.3729750	2.5536120	2.8995900
H	-1.2244780	-0.4487320	3.8360140
H	-3.6542700	-0.7586920	3.6123320

s1-c

Bi	0.1509840	-0.1984590	-0.7956630
P	-1.6674610	-1.6989270	0.5988960
P	2.1779270	-0.9480310	0.8597650
P	0.3579260	2.4834680	-0.3971280
C	-3.0140830	-0.5896320	1.3988790
C	-2.4242120	-2.5420640	-0.9434010
C	-3.0692920	-3.8846770	-0.5543410
C	-3.3361140	-1.7377380	-1.8767330
C	-1.2185010	3.1892250	-1.2063350
C	-1.5365550	2.4835890	-2.5315020
C	-1.0425900	4.6982220	-1.4467090
C	3.7753010	-0.5264260	-0.1486360
C	4.9623240	-0.9505020	0.7437630
C	3.8430820	0.9993770	-0.3365310
C	-2.4004150	-0.1513450	2.7430940
C	-3.4261490	0.6518350	0.5939780
C	-4.2570920	-1.4541400	1.6868860
H	-2.1726620	-1.0174470	3.3834090
H	-1.4676720	0.4126430	2.6007090
H	-3.1043970	0.5020170	3.2875470
H	-3.9095010	0.3947530	-0.3572180
H	-4.1418430	1.2610170	1.1758290
H	-2.5576820	1.2838550	0.3708410
H	-4.9640660	-0.8818310	2.3134180
H	-4.7900520	-1.7348360	0.7670360
H	-3.9995170	-2.3759460	2.2316610
H	-1.5122420	-2.7913100	-1.5169930
H	-2.8767390	-0.7895340	-2.1958510
H	-4.3010350	-1.5030710	-1.4029310
H	-4.0006570	-3.7428480	0.0145280
H	-2.3917800	-4.4961170	0.0614170
H	-2.0740480	3.0443760	-0.5287110
H	-2.3623900	3.0012740	-3.0481210
H	-1.8514040	1.4387410	-2.3851430
H	-0.9051050	5.2629980	-0.5139880
H	-1.9370290	5.1078620	-1.9478250
H	4.9931500	-2.0379010	0.9188670
H	5.9124670	-0.6741610	0.2524500
H	3.0487700	1.3851520	-0.9930070
H	4.8110900	1.2745130	-0.7908690

C	0.2968490	3.1339600	1.4268560
C	0.9729370	2.1136340	2.3543790
C	1.1513580	4.4224700	1.4361850
C	-1.1108910	3.4481260	1.9527840
H	0.4111290	1.1726190	2.4257960
H	1.9868060	1.8544760	2.0180890
H	1.0569800	2.5375680	3.3704440
H	0.7384030	5.2096770	0.7887240
H	1.1967050	4.8275130	2.4630790
H	2.1820330	4.2227510	1.1051540
H	-1.0410670	3.7991550	2.9975450
H	-1.6096370	4.2431580	1.3792280
H	-1.7607470	2.5639380	1.9467880
C	3.8968070	-1.2162310	-1.5159850
H	3.9364870	-2.3120300	-1.4247010
H	3.0600360	-0.9576220	-2.1844520
H	4.8293690	-0.8973870	-2.0162600
H	4.9304100	-0.4495980	1.7244930
H	3.7666420	1.5335710	0.6239770
C	2.1481010	-2.8590210	0.7631700
C	1.5674280	-3.4104970	2.0753420
C	1.4913990	-3.5432060	-0.4435210
H	3.2238520	-3.1046100	0.7411580
H	0.4982230	-3.1602760	2.1645540
H	2.0891300	-3.0021760	2.9547620
H	0.3958040	-3.4496010	-0.3985840
H	1.8338980	-3.1421140	-1.4077480
H	-0.1717190	4.8986400	-2.0924040
H	-0.6682520	2.4832450	-3.2119740
H	-3.5577860	-2.3218070	-2.7869000
H	-3.3221070	-4.4629150	-1.4605020
H	1.7154230	-4.6248560	-0.4332630
H	1.6586180	-4.5108190	2.1032490

s1-d

Bi	0.1206820	0.0691820	-0.7750030
P	1.6787690	-1.6047250	0.7452970
P	0.4216460	2.3485510	0.6976310
P	-2.4822170	0.2307030	-0.0382720
C	0.9102950	-3.3755140	0.6128530
C	3.1486070	-1.5298360	-0.4855870
C	4.4460010	-2.0294450	0.1743160
C	2.9848470	-2.1020490	-1.9004530
C	-3.3423440	-1.0547880	-1.1527860
C	-2.8531180	-0.9529920	-2.6045330
C	-4.8640390	-0.8317950	-1.1046980
C	-0.1436930	3.7217830	-0.5256500
C	-0.9107970	4.7749090	0.2921290
C	-0.9743830	3.2854990	-1.7380290
C	0.1233820	-3.5612240	1.9246680
C	-0.0275530	-3.5911990	-0.5863500
C	2.0287590	-4.4348490	0.5917970
H	0.7909050	-3.5295550	2.7995250
H	-0.6376090	-2.7842410	2.0634580
H	-0.3927920	-4.5379170	1.9231720
H	0.4821480	-3.4296770	-1.5474960
H	-0.4153650	-4.6261940	-0.5850470
H	-0.8981040	-2.9179980	-0.5440660
H	1.5791490	-5.4369980	0.7077920
H	2.5939600	-4.4386200	-0.3494620
H	2.7392240	-4.2960610	1.4215850
C	2.3139160	2.7077190	0.7910120
C	2.9100920	1.7404490	1.8287240
C	3.0602840	2.5808520	-0.5455690
C	2.4779600	4.1464410	1.3270620
H	2.4333840	1.8681210	2.8135300
H	2.7858030	0.6847270	1.5472250
H	3.9908990	1.9336710	1.9479640
H	2.6400990	3.2399770	-1.3214070
H	4.1225030	2.8578060	-0.4184940
H	3.0354190	1.5518010	-0.9346860
H	3.5466200	4.3444420	1.5233610
H	2.1331100	4.9077470	0.6108250
H	1.9319390	4.2919940	2.2729060
H	3.2677030	-0.4372320	-0.5911840
H	2.0970350	-1.7030490	-2.4151870
H	2.9031140	-3.1988280	-1.8961110

H	4.4583670	-3.1233600	0.2878190
H	4.5891730	-1.5859810	1.1717660
H	-3.1223940	-2.0701040	-0.7851370
H	-3.4632580	-1.6011320	-3.2563880
H	-1.8050530	-1.2692370	-2.7196710
H	-5.2810820	-0.9455910	-0.0941070
H	-5.3765710	-1.5617190	-1.7555280
H	0.7790250	4.1862950	-0.9093610
H	-0.3270940	5.1438250	1.1495170
H	-1.1687140	5.6433980	-0.3400210
H	-0.4100450	2.6228590	-2.4132860
H	-1.2724530	4.1718450	-2.3251440
C	-2.8549840	-0.3615300	1.7654670
C	-1.6452660	-0.0654850	2.6686560
C	-4.0367560	0.5092900	2.2500900
C	-3.2406290	-1.8445910	1.8721790
H	-0.7733720	-0.6866610	2.4190090
H	-1.3264760	0.9850440	2.5965750
H	-1.9135340	-0.2697220	3.7204210
H	-4.9385450	0.3761260	1.6333260
H	-4.2988130	0.2340490	3.2874530
H	-3.7765030	1.5787980	2.2380280
H	-3.3479960	-2.1224110	2.9354490
H	-4.1995350	-2.0648310	1.3816690
H	-2.4812940	-2.5055300	1.4343560
H	-1.8503240	4.3533060	0.6865030
H	-1.8945830	2.7624720	-1.4334920
H	-5.1239100	0.1788280	-1.4609870
H	-2.9386710	0.0774380	-2.9894200
H	3.8648190	-1.8465010	-2.5166620
H	5.3166260	-1.7553440	-0.4472330

s1-TS2

Bi	-0.1690000	0.0007640	-0.9626460
P	-0.6434730	2.0342250	0.8421740
P	-1.6233380	-1.8985620	0.3611470
C	-0.2164370	3.7934290	-1.5229330
H	0.7989270	4.0970690	-1.2421140
H	-0.6495510	4.6146940	-2.1228180
H	-0.1368830	2.9166350	-2.1852510
C	1.0712090	2.3344480	1.6088470
H	1.4713680	1.3036310	1.6656850
C	-1.1124740	3.5140540	-0.3033670
C	0.9069520	2.8546110	3.0469290
H	0.5066040	3.8819230	3.0634100
H	0.2220110	2.2213730	3.6311610
C	2.1001570	3.1674530	0.8399470
H	2.2848640	2.7704820	-0.1681980
H	1.7917110	4.2191790	0.7412800
C	-2.0737080	-1.0232050	1.9921040
H	-2.4020740	0.0034400	1.7657240
C	1.9815170	-2.5590170	1.0612800
H	0.9416420	-2.5092690	1.4174940
C	-1.1874600	4.7727840	0.5836450
H	-1.8460710	4.6157340	1.4525830
H	-1.5907180	5.6217680	0.0019970
H	-0.1978020	5.0725340	0.9594030
C	-2.8564850	-2.0759590	-2.1709590
H	-2.3863280	-1.1792090	-2.6043510
H	-3.7607010	-2.2868690	-2.7679620
H	-2.1625640	-2.9220090	-2.3054690
C	-3.2438360	-1.8828190	-0.6914100
C	4.5287430	-1.7889360	-1.2012880
H	4.7434770	-2.4555270	-0.3526050
H	5.5007770	-1.4646000	-1.6150200
H	4.0007780	-2.3709610	-1.9711370
C	-0.8211010	-0.9314390	2.8765460
H	-0.4967930	-1.9299590	3.2147180
H	0.0305430	-0.4574500	2.3673530
C	3.7161690	-0.5550630	-0.7667530
C	-2.5319530	3.1835070	-0.8097380
H	-2.5314090	2.3240500	-1.5013900
H	-2.9453210	4.0430930	-1.3661320
H	-3.2191890	2.9504680	0.0186740
C	-4.0844050	-0.6064970	-0.5416420
H	-4.4358890	-0.4541740	0.4888860
H	-4.9779560	-0.6627710	-1.1890410

H	-3.5216650	0.2936410	-0.8331050
C	2.1545350	-3.9257400	0.3823930
H	3.1615050	-4.0519830	-0.0434610
H	1.4223080	-4.0567710	-0.4270740
C	-3.1884440	-1.7449720	2.7648350
H	-4.1653850	-1.6791330	2.2670010
H	-2.9545800	-2.8120620	2.9184820
C	2.9070870	-2.3950910	2.2735790
H	3.9695080	-2.4459880	1.9853430
H	2.7391680	-1.4365580	2.7866080
C	4.4975460	0.2195530	0.3121880
H	4.6774790	-0.3957290	1.2051100
H	3.9540630	1.1194820	0.6279340
H	5.4817440	0.5320530	-0.0834070
C	-4.0699850	-3.1194110	-0.2710950
H	-3.4963310	-4.0489290	-0.4117520
H	-4.9788270	-3.1857190	-0.8955940
H	-4.3942820	-3.0809260	0.7767980
C	3.4895550	0.3558350	-1.9846540
H	4.4629940	0.7072400	-2.3685830
H	2.9016700	1.2511870	-1.7253370
H	2.9725220	-0.1744630	-2.7991450
P	2.0070540	-1.1065270	-0.1328660
H	3.0631070	3.1667540	1.3793940
H	1.8829640	2.8698060	3.5644820
H	-3.3024140	-1.2843850	3.7612820
H	-1.0411460	-0.3302940	3.7744670
H	2.0016940	-4.7350000	1.1191980
H	2.7307480	-3.2110850	2.9965270

s1-inv

Bi	-0.22606799	-0.15280121	-0.74385516
P	0.55685110	2.28283250	0.13321649
P	-2.44183562	-0.44589538	0.81907910
C	2.59382807	2.01262316	-2.00421973
H	3.39788707	1.75804253	-1.30282688
H	3.06125299	2.50053168	-2.87871989
H	2.14375058	1.07288401	-2.36159573
C	1.75442729	2.08912807	1.60477908
H	1.25535910	1.29723090	2.18606024
C	1.54273554	2.95032120	-1.38532945
C	-2.91586317	1.36743040	1.19534237
H	-2.62516299	2.01319675	0.34997062
C	1.66198856	-1.68102474	2.10615416
H	2.28952334	-0.80654098	1.88515185
C	2.21593842	4.27069874	-0.96048675
H	1.49615682	4.96543017	-0.49918548
H	2.64359924	4.77111187	-1.84754349
H	3.03868729	4.10814814	-0.24944759
C	-3.01962438	-2.34912629	-1.15464699
H	-2.14702750	-2.12609400	-1.78932802
H	-3.75778524	-2.86443682	-1.79361477
H	-2.69473511	-3.05461337	-0.37294722
C	-3.65052998	-1.07565400	-0.55318901
C	2.81805251	-4.17649639	-0.32621841
H	3.23530709	-4.25509724	0.68580763
H	3.60387724	-4.49949305	-1.03243889
H	1.98338180	-4.89091845	-0.40177307
C	2.36186297	-2.74295764	-0.68047267
C	0.47335261	3.27051144	-2.45068212
H	-0.04128033	2.36252938	-2.80618737
H	0.94833804	3.74186842	-3.32902069
H	-0.29080718	3.96260370	-2.06346413
C	-3.94621864	-0.06838844	-1.67436970
H	-4.41246294	0.85331195	-1.29722884
H	-4.64071970	-0.51230040	-2.41054684
H	-3.03144154	0.21736694	-2.21796469
C	3.54897015	-1.77355485	-0.60402129
H	3.97919569	-1.71371608	0.40653098
H	3.25776806	-0.75746961	-0.91018147
H	4.35473796	-2.10399328	-1.28412422
C	-4.96225212	-1.50046856	0.14297616
H	-4.77009544	-2.23243638	0.94326283
H	-5.63376276	-1.97622119	-0.59414415
H	-5.50684976	-0.65529485	0.58261881
C	1.82885911	-2.78028101	-2.12681356
H	2.58928703	-3.22459035	-2.79242423

H	1.60329176	-1.77565280	-2.51720622
H	0.91485702	-3.39125097	-2.21105992
P	0.88172236	-2.30589943	0.48347993
C	0.54224602	-1.25622259	3.06753873
H	-0.11486831	-0.48462256	2.64546943
H	-0.10103951	-2.11067815	3.33250886
H	0.97728222	-0.85686509	3.99964636
C	2.54311014	-2.74135861	2.78444135
H	2.84126670	-2.38928695	3.78714540
H	2.00283672	-3.69401438	2.91430420
H	3.46704677	-2.94666630	2.22672433
C	3.19419202	1.63374716	1.35181286
H	3.79109134	2.41591888	0.85904338
H	3.68987510	1.40687826	2.31185155
H	3.24309584	0.73055224	0.73110564
C	1.72088142	3.36453694	2.46601550
H	2.27689700	3.20294241	3.40628480
H	2.18732528	4.21831379	1.95012181
H	0.69091548	3.65223552	2.72571851
C	-2.10934186	1.79288783	2.43243116
H	-1.02764068	1.66836025	2.29339491
H	-2.28419307	2.85886615	2.65552508
H	-2.40646010	1.20382286	3.31597152
C	-4.40975266	1.57858548	1.48092294
H	-4.57467386	2.61371429	1.82670197
H	-5.04256256	1.42810821	0.59622150
H	-4.76891853	0.90559560	2.27738921

s1-rot

Bi	-0.1227070	-0.1605220	-0.8566870
P	1.8354760	-1.9569440	-0.4123220
P	-0.4567670	2.2145830	0.4401750
P	-2.0426040	-1.5791370	0.4587990
C	1.4738560	3.1841290	-1.5811040
H	2.3034550	3.2170550	-0.8639560
H	1.6756820	3.9466900	-2.3552440
H	1.4959600	2.2024970	-2.0800130
C	0.6782270	2.4137720	1.9545690
H	0.5643540	1.4276970	2.4290170
C	0.1115140	3.4599410	-0.9208340
C	0.0704510	3.4371350	2.9314120
H	0.1330290	4.4653120	2.5430100
H	-0.9890310	3.2211690	3.1375900
C	2.1749880	2.6580630	1.7518920
H	2.6224190	1.9259200	1.0685540
H	2.3767100	3.6645340	1.3555890
C	-2.5654080	-0.3846300	1.8516430
H	-2.5862460	0.6424040	1.4531510
C	3.2306720	-1.1452900	-1.4283680
H	2.6598820	-0.8002470	-2.3110630
C	0.1184850	4.8707170	-0.2992620
H	-0.8348600	5.1010870	0.2022670
H	0.2698490	5.6238270	-1.0931050
H	0.9301300	4.9994620	0.4310130
C	-2.8922930	-2.0566420	-2.1825410
H	-2.1582910	-1.3864640	-2.6560430
H	-3.7159590	-2.1984980	-2.9037710
H	-2.4091980	-3.0356030	-2.0285190
C	-3.4502040	-1.4825680	-0.8644660
C	2.5480120	-0.8505830	2.2112870
H	3.3281450	-0.1946550	1.8058320
H	2.8174700	-1.0797630	3.2582820
H	1.6084450	-0.2846930	2.2265990
C	-1.5019040	-0.4590550	2.9570980
H	-1.5232350	-1.4380100	3.4636220
H	-0.4836500	-0.3158040	2.5721040
C	2.4025650	-2.1512360	1.4117060
C	-0.9879900	3.4132380	-2.0028120
H	-1.0391090	2.4307400	-2.5000820
H	-0.7768070	4.1639670	-2.7845460
H	-1.9817500	3.6304780	-1.5806190
C	-3.9985770	-0.0684980	-1.1072030
H	-4.4413870	0.3700890	-0.2011270
H	-4.7873590	-0.0947110	-1.8806730
H	-3.2147730	0.6214750	-1.4588140
C	3.9666950	0.0696970	-0.8563330
H	4.5888450	-0.1994360	0.0109120

H	3.2710810	0.8608070	-0.5435660
C	-3.9400950	-0.7018010	2.4579230
H	-4.7690740	-0.4876000	1.7702580
H	-4.0157200	-1.7575280	2.7686050
C	4.2195100	-2.2075880	-1.9431660
H	4.8688850	-2.5878810	-1.1404560
H	3.6968540	-3.0680190	-2.3883370
C	3.7430250	-2.9121860	1.4270940
H	4.5745290	-2.2934880	1.0586530
H	3.7030600	-3.8329600	0.8238820
H	3.9854980	-3.2030750	2.4646070
C	-4.5841300	-2.4260140	-0.4045300
H	-4.2107990	-3.4496770	-0.2435650
H	-5.3642110	-2.4715150	-1.1854830
H	-5.0694320	-2.0955100	0.5222310
C	1.3219180	-3.0366260	2.0646140
H	1.5727640	-3.2114010	3.1257660
H	1.2472040	-4.0154150	1.5658000
H	0.3260230	-2.5721160	2.0247150
H	4.6408260	0.5011530	-1.6168470
H	4.8758980	-1.7703480	-2.7157310
H	-1.6821970	0.3192070	3.7177710
H	-4.0971780	-0.0808340	3.3569550
H	0.6163240	3.4133610	3.8910110
H	2.7041540	2.5783020	2.7172720

s1-a-Birad

Bi	1.5105830	1.4571410	-1.5779110
P	2.9647630	0.2252380	0.2988560
P	-0.3342200	1.8682900	0.3147430
C	-1.1443570	4.0878080	-1.4588040
H	-0.5140620	4.8072210	-0.9205370
H	-1.9969970	4.6456540	-1.8870150
H	-0.5593470	3.6927720	-2.3044690
C	0.6049150	2.9053860	1.6123560
H	1.5234690	2.3009340	1.7176500
C	-1.6651220	2.9628220	-0.5481410
C	-0.1189370	2.8577670	2.9690570
H	-1.0444100	3.4540590	2.9666930
H	-0.3842670	1.8275530	3.2523490
C	1.0307890	4.3323110	1.2539370
H	1.5825290	4.3726250	0.3022350
H	0.1679050	5.0122770	1.1778520
C	1.8348460	-1.2130370	0.8429160
H	0.8884730	-0.6670050	1.0054520
C	-2.5656840	3.5674940	0.5464550
H	-2.9314280	2.8006590	1.2477720
H	-3.4467520	4.0435670	0.0806210
H	-2.0439400	4.3424500	1.1268670
C	4.2007680	-1.0997860	-2.0345260
H	3.5086170	-1.9494610	-1.9750340
H	5.1438340	-1.4676400	-2.4782190
H	3.7763760	-0.3671780	-2.7392350
C	4.4844650	-0.4726480	-0.6589140
C	-2.5021290	1.9820440	-1.3943230
H	-1.9000070	1.5054570	-2.1859240
H	-3.3284620	2.5222610	-1.8890080
H	-2.9389440	1.1820880	-0.7762090
C	1.5488560	-2.3463900	-0.1468650
H	2.4382000	-2.9708170	-0.3253860
H	1.1964810	-1.9678630	-1.1186310
C	2.2761960	-1.7589490	2.2118780
H	3.2088460	-2.3401980	2.1456700
H	2.4365730	-0.9489900	2.9400540
C	5.1781940	-1.5119970	0.2429040
H	4.5864960	-2.4341910	0.3383160
H	5.3663440	-1.1181360	1.2543990
H	6.1529790	-1.7911490	-0.1950430
C	5.4282790	0.7328980	-0.8443910
H	6.3578390	0.4095590	-1.3451340
H	5.7009310	1.1881380	0.1206070
H	4.9730600	1.5182230	-1.4707760
H	0.7655290	-3.0106560	0.2580790
H	1.4993180	-2.4305100	2.6174120
H	0.5341260	3.2689730	3.7586200
H	1.6905860	4.7368570	2.0412360

s1-a-Prad

P	0.9388620	-1.0539230	-0.5406890
C	-0.6780850	-0.3481010	0.1362540
H	-0.5418110	-0.2145300	1.2243370
C	3.5830890	-0.7381920	0.1889120
H	3.4841490	-1.5502050	0.9273710
H	4.4428350	-0.1127300	0.4878500
H	3.8252790	-1.1960820	-0.7842500
C	2.3036130	0.1215800	0.1121530
C	-1.7889930	-1.3887920	-0.0811950
H	-1.9638500	-1.5684810	-1.1556590
H	-1.5431610	-2.3571400	0.3831170
C	2.0038690	0.7048730	1.5020190
H	1.1418030	1.3885220	1.4922230
H	2.8743640	1.2823800	1.8610840
H	1.8042100	-0.0858990	2.2435070
C	-1.0736220	1.0069270	-0.4737990
H	-0.3548840	1.8030380	-0.2316220
H	-1.1480230	0.9433020	-1.5723390
C	2.5343350	1.2617960	-0.9020240
H	2.7689340	0.8709940	-1.9052000
H	3.3881960	1.8844670	-0.5780200
H	1.6590220	1.9215340	-0.9944650
H	-2.7394100	-1.0365060	0.3549680
H	-2.0594780	1.3252490	-0.0918870

s2-a

Bi	0.5932360	-0.1275600	-1.4025510
P	-0.6973880	-1.8067220	0.3048280
P	2.5746670	0.8464770	0.1924180
P	-1.3033050	1.7883670	-1.1605620
C	4.0805960	-1.5779250	-0.5971490
H	3.8448330	-2.1012510	0.3395090
H	5.0512950	-1.9674790	-0.9533290
H	3.3256330	-1.8537230	-1.3501190
C	4.1682590	-0.0578860	-0.4075160
C	5.2590530	0.2660330	0.6310410
H	5.3548290	1.3512700	0.7962480
H	6.2354480	-0.1087720	0.2758260
H	5.0475760	-0.2082950	1.6017350
C	0.5468820	3.5545340	-2.3374150
H	1.4376340	2.9303440	-2.1612090
H	0.9007150	4.5890660	-2.4903120
H	0.0670990	3.2249620	-3.2742180
C	-0.4245220	3.5064370	-1.1461630
C	-0.7515480	-3.6996680	-1.9554400
H	-1.7986790	-3.4499930	-2.1790370
H	-0.5847800	-4.7421840	-2.2818810
H	-0.1032340	-3.0550850	-2.5704370
C	-0.4426730	-3.5675460	-0.4581130
C	4.5283650	0.6019250	-1.7524620
H	3.7697770	0.3982820	-2.5268760
H	5.4891230	0.2032640	-2.1223410
H	4.6288050	1.6944790	-1.6552570
C	0.3154930	3.8548230	0.1511130
H	-0.3631780	3.8722440	1.0162350
H	0.7657170	4.8598540	0.0579360
H	1.1284360	3.1447840	0.3580780
C	-1.3726430	-4.5066800	0.3355740
H	-2.4339160	-4.2738280	0.1599470
H	-1.1848590	-4.4455670	1.4198550
H	-1.2001260	-5.5516180	0.0220720
C	-1.5647500	4.5219010	-1.3694940
H	-2.0902480	4.3469600	-2.3216470
H	-1.1508560	5.5458660	-1.3906840
H	-2.3062280	4.4809900	-0.5552590
C	1.0182820	-3.9666590	-0.1907230
H	1.1981170	-4.9976730	-0.5416420
H	1.2576230	-3.9359920	0.8841370
H	1.7304870	-3.3142450	-0.7183080
C	-1.9912670	1.6857370	0.5564880
C	-1.2563900	1.3487010	1.7070610
C	-3.3641060	1.9584780	0.6984010
C	-1.8710570	1.2870620	2.9590150
H	-0.1944390	1.1233520	1.6331890
C	-3.9786790	1.9106360	1.9533780
H	-3.9613710	2.1897080	-0.1873000

C	-3.2348550	1.5715640	3.0869460
H	-1.2740960	1.0108470	3.8320680
H	-5.0479110	2.1208820	2.0404730
H	-3.7179760	1.5213500	4.0664250
C	2.2755360	0.2156010	1.9068060
C	2.1807340	1.1955820	2.9156360
C	2.0995310	-1.1308020	2.2761910
C	1.9181540	0.8436780	4.2428550
H	2.3071900	2.2486980	2.6507470
C	1.8486360	-1.4858250	3.6032400
H	2.1380800	-1.9116060	1.5192310
C	1.7536780	-0.5005740	4.5910730
H	1.8446060	1.6227690	5.0062160
H	1.7078650	-2.5383730	3.8627420
H	1.5479130	-0.7794900	5.6278200
C	-2.4891560	-1.4992130	-0.0776090
C	-3.0395210	-1.2239410	-1.3435760
C	-3.3600000	-1.5450780	1.0269710
C	-4.4111910	-1.0084300	-1.4988110
H	-2.3955790	-1.1595300	-2.2225720
C	-4.7331640	-1.3396980	0.8723500
H	-2.9491990	-1.7234890	2.0237150
C	-5.2633260	-1.0698200	-0.3919240
H	-4.8135940	-0.7844700	-2.4901790
H	-5.3869820	-1.3717520	1.7475630
H	-6.3358740	-0.8975530	-0.5141690

s2-b

Bi	-0.201743152	-0.281644338	-1.009736552
P	1.582508438	-1.16682575	0.806424136
P	-0.64336016	2.127095873	0.164411405
P	-2.16635043	-1.662137619	0.234338241
C	1.003023586	3.138742022	-2.077295474
H	1.900413109	3.156123216	-1.445992164
H	1.110450582	3.931977297	-2.838994221
H	0.999873385	2.175427114	-2.611450758
C	-0.302878303	3.344192288	-1.289682911
C	-0.372927861	4.774525054	-0.712927585
H	-1.317564543	4.939718134	-0.170482047
H	-0.326311931	5.508442085	-1.536957269
H	0.450968932	4.997208432	-0.02167624
C	-3.761976335	-2.038518797	-1.974652075
H	-2.891838814	-1.838022752	-2.622274401
H	-4.668888228	-1.840748879	-2.572218599
H	-3.751113218	-3.108722888	-1.715400869
C	-3.758672235	-1.145799742	-0.716057875
C	3.407994307	-3.268407102	0.917866107
H	4.258071668	-2.581131344	0.788787247
H	3.741870653	-4.276084355	0.613233475
H	3.158903463	-3.308125336	1.990704942
C	2.194216226	-2.848191175	0.065380035
C	-1.490354187	3.167418538	-2.259244023
H	-1.490094719	2.174931849	-2.738699571
H	-1.42560725	3.91697941	-3.066793964
H	-2.458317628	3.299119984	-1.750821154
C	-3.861529315	0.322714319	-1.159525733
H	-3.759628411	1.03679392	-0.332037059
H	-4.84023086	0.5004577	-1.640446306
H	-3.088929518	0.574945913	-1.901635295
C	2.608307496	-2.802757989	-1.411445254
H	3.407868812	-2.067925605	-1.589147571
H	1.76055623	-2.555214686	-2.070345932
H	2.989372827	-3.791302765	-1.725272619
C	-4.966751288	-1.524251445	0.162839701
H	-4.907099761	-2.570395466	0.504002148
H	-5.897547758	-1.413925445	-0.421166277
H	-5.052060479	-0.885187891	1.053080102
C	1.061358559	-3.869437606	0.266809945
H	1.401094924	-4.870704682	-0.051169764
H	0.159735182	-3.621607666	-0.314014604
H	0.762131848	-3.93887764	1.325164265
C	0.724504908	2.306355182	1.397021009
C	0.53124991	1.592752326	2.599228177
C	1.85873905	3.13650722	1.307346535
C	1.424012835	1.710120359	3.664501684
H	-0.333377837	0.939706612	2.705725212
C	2.742462075	3.266807662	2.381548467

H	2.072924616	3.692626034	0.397488065
C	2.531228698	2.557782283	3.566523632
H	1.24174061	1.136575639	4.57697576
H	3.614260654	3.917905649	2.279686566
H	3.228724383	2.658237909	4.402111237
C	3.035971156	-0.064484613	0.446654097
C	3.329309328	0.546061368	-0.787356873
C	3.913173498	0.170371382	1.520881491
C	4.451630072	1.363789498	-0.940381347
H	2.675624841	0.387290724	-1.647842632
C	5.047292143	0.972242297	1.366521365
H	3.691455404	-0.266868021	2.497205627
C	5.318123015	1.576520811	0.136631253
H	4.650693531	1.833300224	-1.907515024
H	5.709731707	1.141639171	2.219323079
H	6.197581316	2.214678626	0.017458779
C	-2.2372554	-0.898958095	1.914901274
C	-1.50652476	-1.583317287	2.908892577
C	-2.937633712	0.262829891	2.296755779
C	-1.475418787	-1.127066602	4.228640802
H	-0.953286717	-2.48593928	2.639145817
C	-2.924669606	0.706409709	3.62199312
H	-3.489012512	0.841243956	1.558711714
C	-2.192411225	0.01697766	4.593363175
H	-0.892961642	-1.67275408	4.975510899
H	-3.476537965	1.610670612	3.891065408
H	-2.175734832	0.37312334	5.62645211

s2-c

Bi	-0.275721962	0.099435854	-0.736843165
P	-1.593780747	-1.205840977	1.283225735
P	1.937585452	-0.929756869	0.444488278
P	0.586104234	2.571975479	-0.077336729
C	-3.392337183	-0.506690797	1.49911302
C	3.437892055	-0.515660691	-0.713399448
C	4.500822117	-1.605199541	-0.475534719
C	3.987481103	0.838487948	-0.227076494
C	-3.344615285	0.328071939	2.793968949
C	-3.907825894	0.354932753	0.33844287
C	-4.350946747	-1.691949554	1.719572279
H	-3.08018233	-0.296984525	3.661028186
H	-2.611987755	1.14406584	2.738603941
H	-4.3329149	0.780603653	2.989007034
H	-3.936833721	-0.203073335	-0.60941435
H	-4.93607799	0.700504496	0.549121793
H	-3.287459174	1.251338132	0.191003907
H	-5.330681228	-1.309177488	2.05600236
H	-4.511658611	-2.272851265	0.800485028
H	-3.978129203	-2.382995271	2.492476564
H	4.191189076	-2.580815873	-0.878513995
H	5.440976519	-1.313420334	-0.976093419
H	3.252359881	1.64803806	-0.349188094
H	4.888354495	1.104533072	-0.808253901
C	0.016499318	3.146276587	1.667555996
C	0.40818133	2.049809463	2.670853462
C	0.82789319	4.425598244	1.958436914
C	-1.478283431	3.469288805	1.761387404
H	-0.192052035	1.136432612	2.546519321
H	1.466427412	1.760537051	2.569325285
H	0.25407727	2.415793268	3.700965757
H	0.618561067	5.216347424	1.219733658
H	0.554819343	4.819544325	2.953692428
H	1.911425002	4.229885861	1.95581233
H	-1.746146598	3.711379754	2.805194252
H	-1.748659116	4.332776555	1.136336448
H	-2.101130813	2.622316363	1.445857909
C	3.120003686	-0.433779804	-2.212776158
H	2.705457995	-1.376301938	-2.599781337
H	2.411181022	0.378809108	-2.438661979
H	4.045041214	-0.224998593	-2.78031008
H	4.720923719	-1.735625719	0.5967123
H	4.272474463	0.797544103	0.836637775
C	1.683603507	-2.739252863	0.090655767
C	1.998179623	-3.622238691	1.139902254
C	1.26756125	-3.29363301	-1.135336364
C	1.931480829	-5.008299844	0.963905676
H	2.307919111	-3.212790606	2.105260862

C	1.198598458	-4.676318325	-1.313610858
H	0.983020446	-2.642451113	-1.964403594
C	1.538807846	-5.539114201	-0.266683994
H	2.187009498	-5.673622325	1.793075588
H	0.863836336	-5.081829388	-2.271445722
H	1.482150262	-6.621655047	-0.407129478
C	-1.900336626	-2.735078853	0.265738985
C	-1.57212396	-3.961409123	0.870687729
C	-2.462627925	-2.765511704	-1.026241366
C	-1.811051957	-5.174345613	0.219473554
H	-1.115128456	-3.961430828	1.863016916
C	-2.699747038	-3.976846873	-1.680581606
H	-2.724991296	-1.834345646	-1.533806252
C	-2.378021705	-5.186863898	-1.057058714
H	-1.539773162	-6.112646348	0.709139184
H	-3.138525765	-3.974941539	-2.6820835
H	-2.561547618	-6.134983615	-1.569216988
C	-0.414514949	3.649870048	-1.211080496
C	0.254491867	4.753662249	-1.774858955
C	-1.767394549	3.455852629	-1.550225113
C	-0.407176818	5.640861497	-2.630032113
H	1.310538435	4.914558614	-1.540775763
C	-2.427870685	4.333824148	-2.413156326
H	-2.31938599	2.612319815	-1.13168242
C	-1.750609065	5.432125686	-2.953373691
H	0.132880447	6.493029022	-3.051207682
H	-3.47812234	4.159998146	-2.662271197
H	-2.267949598	6.119666544	-3.627522326

s2-d

Bi	0.082734156	-0.122974337	-0.756093726
P	-1.597820366	1.424405317	0.723036614
P	-0.441962486	-2.585546883	0.195169073
P	2.239851596	0.339171803	0.824090991
C	-1.022144165	3.253831057	0.504816448
C	-0.019153509	3.490994737	1.648829375
C	-0.375002056	3.613379048	-0.839490622
C	-2.266399058	4.134192273	0.726074335
H	-0.487119942	3.333609626	2.63328146
H	0.848703303	2.820102923	1.578411264
H	0.359372872	4.527247649	1.609772973
H	-1.059257747	3.446258876	-1.684334794
H	-0.103883386	4.684345106	-0.846749848
H	0.55232021	3.050216082	-1.019003062
H	-1.960389014	5.193587751	0.787091203
H	-2.988294436	4.037853447	-0.099183397
H	-2.786124534	3.881716271	1.664774629
C	3.523425838	-1.057472387	0.432473613
C	-1.300565311	-2.559530846	1.920029375
C	-0.370220738	-1.819051679	2.894762278
C	-2.695827675	-1.926866731	1.94597652
C	-1.405830847	-4.044292595	2.325240501
H	0.646012776	-2.241703509	2.895590122
H	-0.295619201	-0.747334183	2.657205194
H	-0.768600912	-1.906666237	3.92068649
H	-3.401141916	-2.450450782	1.284773557
H	-3.101279353	-1.976139665	2.972673174
H	-2.662141274	-0.868498853	1.657218498
H	-1.866353731	-4.122256271	3.326168301
H	-2.037356238	-4.612810825	1.623373812
H	-0.417366295	-4.528357167	2.368506196
C	3.182248526	-2.236441902	1.361124143
H	3.149350139	-1.924421016	2.417209594
H	2.21539422	-2.692495763	1.100898252
H	3.954517988	-3.019701029	1.26413836
C	3.559414314	-1.544323737	-1.022519131
H	3.805448031	-0.733456087	-1.723589112
H	4.337928383	-2.320327803	-1.13540422
H	2.603907145	-1.999735302	-1.327308042
C	4.903726518	-0.491730491	0.818390227
H	5.659809639	-1.29391812	0.752910012
H	5.217828318	0.322897032	0.148495249
H	4.913732737	-0.107313049	1.851472004
C	-3.064269618	1.294953898	-0.421112589
C	-4.301133489	0.991762797	0.180724221
C	-3.032349842	1.42533845	-1.82445806
C	-5.45728282	0.813471751	-0.585432947

H	-4.353868988	0.886018148	1.267354424
C	-4.1864064	1.251784977	-2.592760414
H	-2.092490434	1.646808961	-2.333925736
C	-5.403004837	0.940549762	-1.976258057
H	-6.40241542	0.571002202	-0.092604969
H	-4.132201079	1.350618111	-3.680115821
H	-6.30392497	0.796894529	-2.578395991
C	-1.80660429	-3.107266942	-0.952028823
C	-1.725445504	-4.414355234	-1.469917496
C	-2.896547928	-2.305624976	-1.345402991
C	-2.703400451	-4.908207804	-2.339641985
H	-0.881532284	-5.050028408	-1.187616911
C	-3.868926795	-2.794098996	-2.221521663
H	-2.999545301	-1.289284893	-0.965507256
C	-3.777106513	-4.097985851	-2.71964859
H	-2.620936036	-5.92765836	-2.726066376
H	-4.699602845	-2.145447164	-2.510930258
H	-4.538435703	-4.480050957	-3.404903058
C	2.943550593	1.779309515	-0.133256922
C	2.998637991	1.895645808	-1.536399568
C	3.451766541	2.846483658	0.631949087
C	3.534249885	3.032738128	-2.147563449
H	2.607280307	1.096587297	-2.16966939
C	3.988822832	3.985441892	0.024466595
H	3.419829303	2.781982407	1.722915991
C	4.029680842	4.083459566	-1.36901174
H	3.55968609	3.099239711	-3.238548912
H	4.372884411	4.800432547	0.643717763
H	4.443648822	4.974955881	-1.847146071

s2-TS2

Bi	0.8837500	0.2976520	-1.3034490
P	2.0739490	-0.5344360	1.0805800
P	-0.3172850	2.5562160	-0.3829800
C	3.5574300	-2.3849880	-0.6850240
H	2.9485930	-3.2479770	-0.3860570
H	4.5535170	-2.7668160	-0.9734050
H	3.0977830	-1.9578090	-1.5901050
C	3.7015390	-1.3335030	0.4288100
C	4.4580960	-1.9259890	1.6366570
H	4.6066710	-1.1691750	2.4230220
H	5.4545780	-2.2808060	1.3168870
H	3.9330890	-2.7772320	2.0917770
C	1.1728120	4.3073180	-1.9041550
H	1.3765880	3.4954100	-2.6226880
H	1.9661790	5.0645360	-2.0303910
H	0.2128180	4.7694760	-2.1820490
C	1.1597710	3.7974870	-0.4482020
C	-2.1687330	-2.8496470	-3.2117120
H	-3.1836230	-2.4285680	-3.1355030
H	-2.2627820	-3.8970070	-3.5533360
H	-1.6223400	-2.2813780	-3.9794570
C	-1.4423370	-2.8007140	-1.8564070
C	4.5337370	-0.1531530	-0.1162480
H	4.0849610	0.2910830	-1.0203720
H	5.5422770	-0.5031830	-0.3965770
H	4.6488910	0.6465060	0.6327030
C	2.5570850	3.2530650	-0.1069180
H	2.6125910	2.7878310	0.8858280
H	3.2948380	4.0749890	-0.1331330
H	2.8951760	2.5038840	-0.8408100
C	-2.1990330	-3.6551790	-0.8261840
H	-3.2427600	-3.3247710	-0.7154060
H	-1.7148250	-3.6245440	0.1595790
H	-2.2220140	-4.7053570	-1.1691720
C	0.8096160	4.9685840	0.4895560
H	-0.1981310	5.3650860	0.2851900
H	1.5311200	5.7910080	0.3393370
H	0.8474110	4.6760730	1.5486290
C	-0.0054570	-3.3253170	-1.9994570
H	-0.0321930	-4.3678070	-2.3603740
H	0.5236590	-3.3142460	-1.0348160
H	0.5771930	-2.7422690	-2.7317810
P	-1.3251640	-0.9949140	-1.2694900
C	-0.6493200	2.2106750	1.4032710
C	0.2909880	2.1604270	2.4520130
C	-2.0024060	1.9661900	1.7158290

C	-0.1083630	1.8740500	3.7594110
H	1.3481230	2.3191740	2.2530800
C	-2.3978160	1.6612350	3.0208850
H	-2.7545150	2.0094030	0.9266730
C	-1.4520240	1.6151970	4.0489430
H	0.6420040	1.8310690	4.5528240
H	-3.4520560	1.4606160	3.2275970
H	-1.7579700	1.3759040	5.0705790
C	0.9442700	-1.8707190	1.6421200
C	-0.3208670	-1.4414270	2.1079600
C	1.2328650	-3.2484400	1.7410800
C	-1.2255110	-2.3347750	2.6831470
H	-0.5924610	-0.3885920	2.0463300
C	0.3188710	-4.1439960	2.3046620
H	2.1859150	-3.6419110	1.3923570
C	-0.9131480	-3.6935270	2.7879700
H	-2.1811880	-1.9564800	3.0553980
H	0.5792210	-5.2040070	2.3704320
H	-1.6216760	-4.3939080	3.2372010
C	-2.9194490	-0.2485030	-0.8091490
C	-3.6558430	-0.7240670	0.2958980
C	-3.4189080	0.8619720	-1.5192330
C	-4.8603710	-0.1209220	0.6636280
H	-3.2618030	-1.5515890	0.8858630
C	-4.6069540	1.4849940	-1.1272290
H	-2.8598530	1.2416080	-2.3760420
C	-5.3356500	0.9919750	-0.0397280
H	-5.4179920	-0.5062750	1.5216250
H	-4.9689700	2.3553070	-1.6807880
H	-6.2690930	1.4748210	0.2607930

s2-inv

Bi	-0.47213999	-1.51521108	0.58785181
P	0.21935029	0.28083372	2.49746681
P	1.73213103	-2.02235779	-0.86475874
C	-2.42547327	1.53629134	2.31527028
H	-2.02200829	2.46365353	1.88669291
H	-3.35625397	1.79736542	2.85102081
H	-2.69390497	0.86803450	1.48572892
C	-1.43887754	0.86876866	3.28102610
C	-1.05534159	1.86374751	4.39391373
H	-0.36399975	1.41357530	5.12416365
H	-1.96326779	2.17932975	4.93808663
H	-0.57882713	2.76739762	3.98339778
C	2.81719367	-3.52986431	1.20551178
H	2.00293538	-3.30241722	1.91296429
H	3.68785795	-3.85049091	1.80339631
H	2.50312841	-4.38301507	0.58263752
C	3.19415734	-2.30450130	0.35242983
C	-4.28841179	-0.72192391	-2.67489372
H	-4.42411417	0.36344838	-2.56174457
H	-5.29033665	-1.18621599	-2.69482406
H	-3.81583614	-0.90231397	-3.65369205
C	-3.45909169	-1.32994366	-1.52631281
C	-2.09371892	-0.37354183	3.91270618
H	-2.43527463	-1.09406626	3.15125114
H	-2.98105687	-0.07241837	4.49610320
H	-1.40384957	-0.89875309	4.59239309
C	3.56997300	-1.13583581	1.27273898
H	3.92353903	-0.26246735	0.70775705
H	4.39280779	-1.44578148	1.94221033
H	2.72812981	-0.82197317	1.90739675
C	-4.16162040	-1.07530941	-0.18225791
H	-4.31403681	-0.00822527	0.02609880
H	-3.60333248	-1.50729771	0.66302318
H	-5.16007298	-1.54816884	-0.18858340
C	4.40019123	-2.65543575	-0.54369547
H	4.19194578	-3.52239108	-1.19077688
H	5.27109132	-2.90546830	0.08776700
H	4.68532395	-1.80813763	-1.18715774
C	-3.35685316	-2.85479465	-1.74356015
H	-4.36603555	-3.30200972	-1.73922714
H	-2.77701770	-3.34761696	-0.94550671
H	-2.87984915	-3.09745608	-2.70590522
P	-1.64960779	-0.69457782	-1.73658712
C	2.19006586	-0.49165423	-1.80301770
C	2.28025254	-0.64007318	-3.20161225

C	2.44624190	0.77743661	-1.25266234
C	2.63472870	0.43748207	-4.01966611
H	2.06564969	-1.61354695	-3.65001217
C	2.80942455	1.85220499	-2.06778316
H	2.34027055	0.94174924	-0.18304545
C	2.90834212	1.68619589	-3.45283229
H	2.69600841	0.29959044	-5.10240362
H	2.99340579	2.82670227	-1.61067944
H	3.18719388	2.52988912	-4.08977340
C	-1.71119614	1.15036204	-1.78557278
C	-2.68862663	1.99483078	-1.22301590
C	-0.66924487	1.75111113	-2.52275966
C	-2.62639263	3.38065856	-1.39108665
H	-3.50720719	1.57861549	-0.63947022
C	-0.60103053	3.13611201	-2.68143105
H	0.09686946	1.12243018	-2.97991858
C	-1.58184262	3.95915902	-2.11991036
H	-3.39824081	4.01227849	-0.94329728
H	0.22742018	3.56797141	-3.24826822
H	-1.53471913	5.04366828	-2.24817192
C	0.96207621	1.83597573	1.82713957
C	2.18420237	2.22675841	2.41098480
C	0.42507262	2.64134383	0.80529669
C	2.84162519	3.39015572	2.00032887
H	2.62315139	1.60679832	3.19713608
C	1.07511922	3.81146741	0.40535769
H	-0.49014014	2.34524146	0.29625447
C	2.28298960	4.19064037	0.99969977
H	3.78988360	3.67166267	2.46577747
H	0.63706290	4.41480985	-0.39169391
H	2.79161645	5.10312289	0.67737590

s2-rot

Bi	-0.2040550	-1.3105500	-0.4751560
P	1.2585230	-1.6464610	1.7762170
P	0.1080470	1.0604530	-1.7749120
P	-2.5627620	-0.8460970	0.7720710
C	2.6318060	-0.2340860	-2.6409420
H	3.2115390	0.2457550	-1.8431050
H	3.3172840	-0.4041540	-3.4905330
H	2.3324810	-1.2245120	-2.2660830
C	1.4211550	0.5925800	-3.1067270
C	1.8911770	1.9020260	-3.7718830
H	1.0386740	2.5120490	-4.1117090
H	2.5076660	1.6642520	-4.6567950
H	2.4995470	2.5216950	-3.0986130
C	-3.5403430	-3.3538530	0.1504260
H	-2.5471650	-3.6982190	-0.1834220
H	-4.2899320	-4.0189840	-0.3123190
H	-3.5957210	-3.4865930	1.2424050
C	-3.8108440	-1.8933330	-0.2612140
C	0.5559260	1.0592540	2.6636940
H	1.3741170	1.5266640	2.1027270
H	0.3366430	1.7098720	3.5292450
H	-0.3306490	1.0684990	2.0152620
C	0.8854640	-0.3581470	3.1475890
C	0.6376610	-0.2288490	-4.1530820
H	0.2558180	-1.1752820	-3.7351730
H	1.3015370	-0.4893560	-4.9955920
H	-0.2191620	0.3339690	-4.5558380
C	-3.7395720	-1.7802190	-1.7916800
H	-4.0163950	-0.7791110	-2.1481260
H	-4.4487390	-2.4932250	-2.2491860
H	-2.7377820	-2.0217830	-2.1814940
C	2.0804220	-0.3394960	4.1227280
H	2.9816330	0.1128610	3.6870650
H	2.3425910	-1.3538770	4.4645400
H	1.8081310	0.2521620	5.0143820
C	-5.2151870	-1.4787830	0.2181660
H	-5.3138030	-1.5641320	1.3123180
H	-5.9747890	-2.1359100	-0.2411570
H	-5.4509770	-0.4414390	-0.0652870
C	-0.3383440	-0.9346440	3.8914510
H	-0.6015120	-0.2695920	4.7327250
H	-0.1298080	-1.9347910	4.3036940
H	-1.2180630	-1.0116050	3.2348080
C	0.8773950	2.2967350	-0.6382430

C	2.2516680	2.5212400	-0.4323920
C	-0.0325500	3.1266130	0.0496420
C	2.6957420	3.5254780	0.4323520
H	2.9920130	1.9011540	-0.9315100
C	0.4105360	4.1190810	0.9250450
H	-1.1029750	2.9907550	-0.0999300
C	1.7793780	4.3252920	1.1220020
H	3.7698390	3.6750010	0.5741570
H	-0.3233940	4.7347470	1.4514310
H	2.1299810	5.1044100	1.8036150
C	-3.0111490	0.9213140	0.4455940
C	-3.2509250	1.5127760	-0.8106100
C	-3.1447590	1.7305610	1.5919560
C	-3.6429240	2.8501800	-0.9110880
H	-3.1019030	0.9394800	-1.7233930
C	-3.5151030	3.0750370	1.4912180
H	-2.9594660	1.2943620	2.5769630
C	-3.7765480	3.6369770	0.2381300
H	-3.8219330	3.2861160	-1.8972650
H	-3.6077290	3.6812810	2.3961320
H	-4.0734550	4.6855660	0.1551920
C	2.9114400	-1.2600500	1.0357670
C	3.5262530	-2.3254420	0.3427020
C	3.6067770	-0.0385410	1.1055500
C	4.7696260	-2.1701610	-0.2733200
H	3.0180090	-3.2931760	0.2907560
C	4.8619940	0.1102750	0.5093130
H	3.1640850	0.8174260	1.6095800
C	5.4476550	-0.9496730	-0.1890220
H	5.2162950	-3.0105120	-0.8113130
H	5.3785460	1.0715450	0.5810720
H	6.4253620	-0.8270270	-0.6616390

s2-a-Birad

Bi	-0.0025780	-1.1411240	0.7901590
P	-1.4023490	0.7708140	-0.5017800
P	1.8688240	-0.6580380	-1.0524520
C	3.9287590	-1.6168230	0.8416060
H	4.3205590	-0.6133680	1.0588020
H	4.7603340	-2.3292840	0.9895070
H	3.1528050	-1.8552110	1.5867620
C	3.4037700	-1.7272540	-0.5970290
C	4.5161170	-1.3016250	-1.5760970
H	4.1969540	-1.4046410	-2.6254620
H	5.4073740	-1.9374740	-1.4297560
H	4.8192830	-0.2553390	-1.4125870
C	-2.4908030	1.6259170	2.0924610
H	-3.4693590	1.1721400	1.8773710
H	-2.6633740	2.4737960	2.7797780
H	-1.8769730	0.8860040	2.6318540
C	-1.7971070	2.1240260	0.8185470
C	2.9966650	-3.1843510	-0.8853780
H	2.2271340	-3.5435610	-0.1817200
H	3.8730330	-3.8472660	-0.7800630
H	2.6031810	-3.3036560	-1.9075450
C	-2.7089910	3.1414280	0.1045450
H	-3.6709230	2.6924520	-0.1893040
H	-2.2303620	3.5509250	-0.7993370
H	-2.9264350	3.9855580	0.7829030
C	-0.4620650	2.7968360	1.1759910
H	-0.6464460	3.6866800	1.8030500
H	0.0928040	3.1206750	0.2817620
H	0.1958390	2.1227530	1.7457730
C	2.3607510	1.0900290	-0.6566520
C	2.2638910	2.0147650	-1.7136430
C	2.7803940	1.5663520	0.6013040
C	2.5891010	3.3624220	-1.5267280
H	1.9223530	1.6701850	-2.6928990
C	3.1186210	2.9082050	0.7864380
H	2.8191520	0.8886960	1.4554770
C	3.0239550	3.8121050	-0.2773850
H	2.5008990	4.0622030	-2.3619990
H	3.4402950	3.2542870	1.7723580
H	3.2793420	4.8644920	-0.1288850
C	-3.0599540	-0.0479070	-0.7232860
C	-3.6907660	-0.9127180	0.1925150
C	-3.7308720	0.2364550	-1.9283230

C	-4.9454660	-1.4631230	-0.0814400
H	-3.1995020	-1.1645560	1.1353600
C	-4.9899450	-0.3073750	-2.2025040
H	-3.2547180	0.8936160	-2.6614050
C	-5.6011240	-1.1598610	-1.2793240
H	-5.4128830	-2.1335880	0.6448120
H	-5.4907930	-0.0683420	-3.1444970
H	-6.5823030	-1.5914950	-1.4935130

s2-a-Prad

P	-0.9872860	-2.3409790	0.6199100
C	-1.3242920	-3.0307950	-2.1449810
H	-2.3432230	-2.6252910	-2.2109630
H	-1.2198890	-3.7993200	-2.9319370
H	-0.6254330	-2.2133370	-2.3846330
C	-1.0109280	-3.6499610	-0.7687300
C	-1.9661060	-4.8170380	-0.4505080
H	-3.0211510	-4.5120650	-0.4211930
H	-1.7263030	-5.2769710	0.5216810
H	-1.8671730	-5.6009120	-1.2229760
C	0.4337820	-4.1968960	-0.7980790
H	0.5260280	-4.9711420	-1.5792170
H	0.7167540	-4.6548470	0.1636830
H	1.1660480	-3.4043700	-1.0226590
C	-2.6473030	-1.6146920	0.7577470
C	-3.8143460	-1.9049160	0.0099670
C	-2.7557410	-0.6201400	1.7654680
C	-5.0150480	-1.2390660	0.2577040
H	-3.7923690	-2.6580720	-0.7758920
C	-3.9557130	0.0436500	2.0112410
H	-1.8728650	-0.3709660	2.3624080
C	-5.0951130	-0.2625420	1.2571340
H	-5.8990260	-1.4855540	-0.3366830
H	-4.0044340	0.8041750	2.7949580
H	-6.0383230	0.2559360	1.4468510

s3-a

Bi	-0.6752270	-1.4033470	0.9124970
P	0.4764210	-1.5189930	-1.5504600
P	-2.8318810	0.2758450	0.8347650
P	1.2751020	0.0882160	2.0371060
C	-4.6685560	-1.9009460	0.0157200
H	-4.9084860	-1.4312450	-0.9474090
H	-5.5424770	-2.5086610	0.3129600
H	-3.8233950	-2.5909520	-0.1349180
C	-4.3750080	-0.8540860	1.0978940
C	-5.5816520	0.0993960	1.2097240
H	-5.4619730	0.8105360	2.0418530
H	-6.5002040	-0.4841620	1.3996920
H	-5.7338000	0.6740870	0.2828100
C	-0.3046840	-0.0003790	4.3563780
H	-1.2277020	-0.3142100	3.8431090
H	-0.6075310	0.4491310	5.3181530
H	0.2931020	-0.8993680	4.5792710
C	0.4896500	1.0270710	3.5313990
C	0.0987950	-4.4459600	-1.0466400
H	0.8911610	-4.5125890	-0.2920470
H	0.0124220	-5.4401090	-1.5211360
H	-0.8539520	-4.2549510	-0.5268320
C	0.3846610	-3.3883950	-2.1237510
C	-4.1682870	-1.5590410	2.4519980
H	-3.3581090	-2.3053480	2.4119730
H	-5.0899650	-2.0941620	2.7402670
H	-3.9297570	-0.8405870	3.2525970
C	-0.4108540	2.2157340	3.1777790
H	0.1199830	2.9560210	2.5602140
H	-0.7329340	2.7248630	4.1044330
H	-1.3151100	1.8862930	2.6481600
C	1.6924620	-3.7259720	-2.8588510
H	2.5524500	-3.7776660	-2.1776890
H	1.9296910	-2.9803430	-3.6342530
H	1.5922520	-4.7072880	-3.3559110
C	1.6804490	1.5352600	4.3692770
H	2.3487880	0.7160690	4.6763250
H	1.3020920	2.0256300	5.2837130
H	2.2720820	2.2802920	3.8155170
C	-0.7772770	-3.4266400	-3.1384850

H	-0.8487670	-4.4340410	-3.5854550
H	-0.6330680	-2.7024080	-3.9548120
H	-1.7452060	-3.2106260	-2.6614040
C	2.0658850	1.4330660	1.0108300
C	1.4154280	2.2579540	0.0522630
C	3.4648900	1.6066970	1.2166780
C	2.1542760	3.2212970	-0.6470410
C	4.1507180	2.6012250	0.5038250
C	3.5227700	3.4136830	-0.4396380
H	1.6364470	3.8410580	-1.3853750
H	5.2218040	2.7305910	0.6874980
C	-3.1616850	1.2499820	-0.7264190
C	-3.4380570	2.6383090	-0.5169650
C	-3.1072640	0.7722170	-2.0576510
C	-3.6416600	3.4780360	-1.6158520
C	-3.3111680	1.6612460	-3.1283000
C	-3.5783320	3.0140670	-2.9364280
H	-3.8522720	4.5370030	-1.4370370
H	-3.2526180	1.2679880	-4.1482890
C	2.2877830	-1.1349060	-1.3003750
C	3.1853390	-1.7579780	-0.3977950
C	2.7862390	-0.0999740	-2.1496750
C	4.5295980	-1.3549590	-0.3658320
C	4.1310800	0.2718560	-2.0674280
C	5.0246590	-0.3414100	-1.1833650
H	5.2051630	-1.8442370	0.3423290
H	4.4945170	1.0763080	-2.7133980
C	1.9274310	0.6207010	-3.1686890
H	1.5099270	-0.0729650	-3.9154410
H	1.0716970	1.1297990	-2.7048590
H	2.5218510	1.3790250	-3.6996590
C	2.8090260	-2.8711150	0.5500440
H	1.7312700	-2.9287190	0.7334200
H	3.1296110	-3.8507050	0.1553690
H	3.2985820	-2.7316700	1.5249790
C	6.4558580	0.1206640	-1.0953370
H	6.5091480	1.1164410	-0.6225160
H	7.0737300	-0.5674490	-0.4992890
H	6.9119630	0.2151200	-2.0940850
C	-2.8329940	-0.6577830	-2.4443750
H	-2.0014700	-0.7048100	-3.1602470
H	-2.5658170	-1.2796850	-1.5871440
H	-3.7149070	-1.1090800	-2.9289990
C	-3.5135420	3.2683050	0.8582570
H	-3.8081190	4.3256050	0.7804720
H	-4.2391420	2.7606580	1.5094540
H	-2.5450930	3.2263100	1.3782910
C	-3.8024280	3.9555230	-4.0931560
H	-4.8376170	4.3376550	-4.0971260
H	-3.1383640	4.8332530	-4.0270150
H	-3.6219010	3.4644200	-5.0609480
C	-0.0409050	2.1237470	-0.2994940
H	-0.2173790	1.2537040	-0.9515660
H	-0.6723760	1.9842620	0.5819980
H	-0.4096130	3.0064240	-0.8409170
C	4.2848370	0.7819190	2.1865600
H	4.3072300	1.2448810	3.1866380
H	3.8804610	-0.2306610	2.3085000
H	5.3246110	0.7033460	1.8362840
C	4.3007590	4.4203180	-1.2489150
H	5.1880690	4.7819560	-0.7066450
H	4.6557820	3.9684450	-2.1925000
H	3.6830170	5.2909640	-1.5187930

s3-b

Bi	-0.3544210	-0.6798480	1.1481940
P	0.9329530	-2.3237680	-0.6301030
P	-2.0164970	0.6771630	-0.4947740
P	1.5541610	1.2395150	1.3914700
C	-0.1851420	1.0538510	-2.5545910
H	-0.0924010	2.0833750	-2.1843990
H	0.0797370	1.0551200	-3.6262630
H	0.5614490	0.4400460	-2.0294520
C	-1.6127770	0.5185210	-2.3722300
C	-2.5962600	1.4812440	-3.0708440
H	-3.6434040	1.1685620	-2.9323690
H	-2.3911620	1.4868890	-4.1561140

H	-2.4841100	2.5134870	-2.7038310
C	2.6497750	0.8602410	3.8915700
H	2.3710360	-0.2045110	3.8468550
H	2.7878970	1.1206570	4.9558100
H	3.6183110	0.9780370	3.3824930
C	1.5625590	1.7628650	3.2691740
C	0.9344240	-4.1473440	1.7325420
H	1.7300070	-3.6066870	2.2586940
H	0.9761860	-5.2007080	2.0636430
H	-0.0337150	-3.7427390	2.0690060
C	1.0769530	-4.0822960	0.2043640
C	-1.7591210	-0.8755650	-2.9914920
H	-1.0521050	-1.5929530	-2.5535610
H	-1.5503910	-0.8175550	-4.0752630
H	-2.7819850	-1.2642810	-2.8746930
C	0.2818610	1.5741640	4.0977500
H	-0.5532790	2.1895470	3.7491350
H	0.4798290	1.8523850	5.1486590
H	-0.0474730	0.5227360	4.1004200
C	2.4043240	-4.7209990	-0.2373240
H	3.2777320	-4.2283810	0.2094820
H	2.5287870	-4.6855960	-1.3311940
H	2.4204380	-5.7826420	0.0672390
C	2.0427670	3.2227040	3.3356440
H	2.9732760	3.3682410	2.7637040
H	2.2477190	3.4979800	4.3856190
H	1.2994850	3.9274730	2.9399860
C	-0.0837730	-4.8880070	-0.4173570
H	-0.0506210	-5.9318700	-0.0577460
H	-0.0268820	-4.9029500	-1.5166040
H	-1.0634760	-4.4745730	-0.1376570
C	-3.7054830	-0.1165980	-0.3255750
C	-4.7839360	0.8096340	-0.1786920
C	-4.0108940	-1.5016340	-0.3413970
C	-6.0997820	0.3389440	-0.1064290
C	-5.3493420	-1.9225140	-0.2627310
C	-6.4107900	-1.0248570	-0.1564840
H	-6.9129930	1.0638520	-0.0016880
H	-5.5614940	-2.9962050	-0.2816080
C	1.0809410	2.7084350	0.3439220
C	-0.0712750	3.5302350	0.4546160
C	2.0076500	2.9786850	-0.7093780
C	-0.2812810	4.5602410	-0.4764130
C	1.7593900	4.0301450	-1.5993170
C	0.6144360	4.8287630	-1.5122300
H	-1.1834540	5.1723860	-0.3831310
H	2.4799280	4.2213560	-2.4002670
C	2.6706370	-1.7144410	-0.9260620
C	3.0020960	-1.5015740	-2.2982950
C	3.6592560	-1.4452540	0.0559110
C	4.2596640	-0.9838420	-2.6340420
C	4.9027000	-0.9266780	-0.3336140
C	5.2213830	-0.6713800	-1.6680170
H	4.4955830	-0.8192200	-3.6896050
H	5.6442280	-0.7075830	0.4408180
C	-2.9717270	-2.5965990	-0.3702230
H	-2.0204860	-2.2841020	-0.8087020
H	-2.7698150	-2.9541000	0.6549010
H	-3.3282370	-3.4656780	-0.9444410
C	-4.5732850	2.3068780	-0.0772240
H	-3.9039420	2.5613460	0.7575680
H	-4.1125250	2.7273120	-0.9828490
H	-5.5332660	2.8206400	0.0822250
C	-7.8440100	-1.4922300	-0.1076840
H	-8.4121330	-0.9639400	0.6749880
H	-8.3554910	-1.2931550	-1.0655490
H	-7.9150280	-2.5727240	0.0880300
C	-1.0950890	3.3970180	1.5511470
H	-1.2689730	2.3521680	1.8295450
H	-0.7774600	3.9414960	2.4557770
H	-2.0576420	3.8217070	1.2358560
C	3.2513170	2.1495850	-0.9408470
H	3.8910230	2.0958470	-0.0479410
H	3.0034970	1.1094890	-1.1955160
H	3.8438200	2.5638990	-1.7704420
C	0.3476570	5.9130460	-2.5259390
H	-0.0402630	5.4828880	-3.4659600
H	-0.3968390	6.6376670	-2.1628970

H	1.2679650	6.4630420	-2.7794280
C	3.4809020	-1.7398760	1.5219050
H	4.0764790	-1.0525510	2.1357020
H	2.4364330	-1.6561200	1.8408030
H	3.8141670	-2.7653520	1.7561140
C	2.0654490	-1.8486780	-3.4376200
H	1.7757580	-2.9108010	-3.4135280
H	1.1309910	-1.2739030	-3.4020590
H	2.5487570	-1.6492720	-4.4058390
C	6.5368340	-0.0430460	-2.0518880
H	6.8618600	-0.3591560	-3.0552310
H	6.4486700	1.0578270	-2.0686240
H	7.3336700	-0.2951600	-1.3352790

s3-c

Bi	0.33851900	-0.76221700	-0.89084700
P	2.46762700	-1.28044300	0.71373900
P	0.41303300	1.76685300	0.13318700
P	-1.60943200	-1.20901900	0.99069200
C	3.36336000	-2.94380400	0.26533700
C	2.32298600	-4.07347100	0.31252200
C	4.12845700	-3.04034700	-1.06008600
C	4.36890300	-3.12472100	1.42563100
H	1.75307600	-4.08137300	1.25261100
H	1.60855400	-4.00137200	-0.52449100
H	2.83079300	-5.05022600	0.22379800
H	4.92561000	-2.28754900	-1.12652700
H	4.60952900	-4.03327800	-1.13074400
H	3.46441500	-2.93681300	-1.93109600
H	4.91437000	-4.07835800	1.30339800
H	5.11510200	-2.31345400	1.44224400
H	3.86280100	-3.14484000	2.40418400
C	-1.51945100	-2.96851000	1.77796400
C	-0.34342800	-2.90560500	2.77097800
C	-2.82600700	-3.16026000	2.57574800
C	-1.36594400	-4.15385500	0.81651300
H	0.60960000	-2.65986300	2.28074600
H	-0.51420900	-2.13329100	3.53896300
H	-0.22884200	-3.87734200	3.28488800
H	-3.71183200	-3.16039200	1.92107600
H	-2.79341800	-4.13300600	3.09947800
H	-2.95548800	-2.37854600	3.34052200
H	-1.26481600	-5.09317700	1.39101000
H	-2.24819400	-4.26005400	0.16879400
H	-0.47609300	-4.06201100	0.17932300
C	0.94631800	3.02076000	-1.26672100
C	2.39463600	3.39560800	-0.88884200
C	0.92922800	2.46850200	-2.70037600
H	3.05508900	2.51994400	-0.86204900
H	2.44141500	3.87097200	0.10375900
H	1.56162900	1.57415200	-2.80222900
H	-0.08342600	2.20975700	-3.03796400
H	1.32666000	3.22925900	-3.39780000
H	2.80584600	4.10752700	-1.62802600
C	3.73953900	0.03411100	0.31466700
C	4.20177000	0.76438700	1.45488900
C	4.31147900	0.33468500	-0.94907900
C	5.25104400	1.67781700	1.31588900
C	3.57508000	0.61109500	2.82541200
C	5.38224000	1.24326300	-1.02986800
C	3.80409900	-0.17469500	-2.28065900
C	5.87748300	1.91679200	0.08503400
H	5.59378900	2.22510700	2.20021000
H	2.49506200	0.82629100	2.78650800
H	3.67937300	-0.41198500	3.21816800
H	4.04061500	1.30472500	3.54274700
H	5.81600300	1.45032800	-2.01401000
H	2.92227300	-0.81735200	-2.19173800
H	3.53300400	0.68137500	-2.91893300
H	4.57791200	-0.74195000	-2.82116200
C	7.01681100	2.89945200	-0.01883600
H	7.41277700	2.96002600	-1.04376200
H	6.69445000	3.91157300	0.27987500
H	7.84687500	2.61748200	0.65061900
C	-3.18740500	-1.22398600	-0.03507400
C	-3.38449400	-1.79712800	-1.31975000
C	-4.27312300	-0.49098800	0.54396200

C	-4.59715700	-1.58596900	-2.00224100
C	-2.37742200	-2.64533700	-2.06952000
C	-5.45818300	-0.31082700	-0.17748900
C	-4.21619100	0.09429500	1.93737100
C	-5.64116300	-0.83599200	-1.46302000
H	-4.71860700	-2.02874900	-2.99696200
H	-2.04210200	-2.13358200	-2.98884000
H	-1.49275300	-2.89695700	-1.47929100
H	-2.84112200	-3.59635900	-2.38493200
H	-6.26777000	0.26778000	0.27963800
H	-4.27479100	-0.69846400	2.70011500
H	-3.28412600	0.64389200	2.11561300
H	-5.06056100	0.77909900	2.10412600
C	-6.92307400	-0.59060900	-2.22085800
H	-7.80244600	-0.88421300	-1.62312700
H	-7.04285000	0.48066700	-2.45972300
H	-6.95189600	-1.15048200	-3.16844100
C	-1.35257400	2.24969800	0.52463900
C	-2.45870200	2.26926700	-0.36132300
C	-1.53210200	2.76191200	1.84580300
C	-3.66870200	2.85118600	0.05110700
C	-2.44831500	1.69714900	-1.75897700
C	-2.75478700	3.34091100	2.20430000
C	-0.44735600	2.69135500	2.90122300
C	-3.83652100	3.40908400	1.31741300
H	-4.51145100	2.85035200	-0.64759500
H	-3.35503700	1.09494900	-1.92801500
H	-2.43408800	2.49922100	-2.51719500
H	-1.58217100	1.05174300	-1.94839600
H	-2.87226700	3.74026800	3.21664700
H	-0.10499900	1.65449700	3.04514000
H	0.44355600	3.27742600	2.62628300
H	-0.82263000	3.06717300	3.86561600
C	-5.15793000	3.99423300	1.74857900
H	-5.75912700	3.24290000	2.29242000
H	-5.02027000	4.85018600	2.42816600
H	-5.75552800	4.33014600	0.88675300
C	0.08243000	4.29118600	-1.19289300
H	-0.94896500	4.12140400	-1.52989700
H	0.03376700	4.69621200	-0.16966600
H	0.52748300	5.06790000	-1.84102600

s3-d

Bi	0.34496200	-0.77276400	-0.92521300
P	2.45507900	-1.28623800	0.70638100
P	0.42823800	1.77185500	0.05544700
P	-1.58876000	-1.21760800	0.97273700
C	3.37100400	-2.93584700	0.24727900
C	2.33728500	-4.07141200	0.22535800
C	4.18493800	-2.99172000	-1.04967100
C	4.33282900	-3.14412800	1.43805400
H	1.73698200	-4.11075200	1.14388600
H	1.65195500	-3.97817500	-0.63211100
H	2.85316600	-5.04232300	0.12587100
H	4.97211900	-2.22642500	-1.07131800
H	4.68208300	-3.97590100	-1.12324800
H	3.55254900	-2.87849900	-1.94140700
H	4.88793600	-4.09106800	1.31096300
H	5.07225700	-2.32984600	1.50455800
H	3.79093300	-3.19374100	2.39511200
C	0.91430900	2.99603600	-1.38540000
C	2.37333700	3.37698200	-1.06130500
C	0.85361200	2.41609600	-2.80635000
C	0.05456300	4.26879300	-1.30700900
H	2.45065800	3.87734700	-0.08396100
H	3.03347100	2.50167900	-1.02844100
H	2.76350200	4.06766600	-1.83016500
H	-0.16649600	2.14159800	-3.10412900
H	1.21997000	3.16610200	-3.53087900
H	1.49082400	1.52545900	-2.91325200
H	0.47947400	5.03290700	-1.98241100
H	-0.98710300	4.09622500	-1.60731800
H	0.03967000	4.69179300	-0.29042000
C	-1.51122700	-3.00330200	1.70387100
C	-0.33786500	-2.97902700	2.70133500
C	-2.81892900	-3.22179400	2.49116900
C	-1.35739500	-4.15416300	0.70226700

H	0.61559400	-2.71167700	2.22549100
H	-0.51257500	-2.23942800	3.49828600
H	-0.22232500	-3.96962000	3.17550800
H	-3.70417700	-3.19812000	1.83695100
H	-2.78645400	-4.21167500	2.98003900
H	-2.94953100	-2.46895300	3.28292900
H	-1.25606900	-5.11252000	1.24317100
H	-2.23987300	-4.23782500	0.05175700
H	-0.46784400	-4.04059800	0.06861600
C	3.72814600	0.04004000	0.35922000
C	4.16902500	0.74520900	1.52144500
C	4.32164000	0.36794700	-0.88697000
C	5.21996600	1.66211800	1.42063900
C	3.51960000	0.56097600	2.87647900
C	5.39206000	1.27854600	-0.92972400
C	3.83444200	-0.11289000	-2.23598400
C	5.86865800	1.92656200	0.20784600
H	5.54512600	2.19122600	2.32150600
H	2.43961000	0.76733600	2.82398300
H	3.62509500	-0.46832400	3.25160500
H	3.96660500	1.24413600	3.61423800
H	5.84151200	1.50840500	-1.90119600
H	2.96372400	-0.77309400	-2.17207500
H	3.55277700	0.75540500	-2.85303000
H	4.62268600	-0.65116200	-2.78438300
C	7.02984000	2.88669000	0.14715600
H	7.26867500	3.17485900	-0.88762900
H	6.82057700	3.80489100	0.71932000
H	7.93687200	2.43482900	0.58520000
C	-1.32405600	2.25376300	0.49844000
C	-1.46318600	2.76809700	1.82341400
C	-2.45731200	2.26814100	-0.35204200
C	-2.67502800	3.34495200	2.21864200
C	-0.34708200	2.69957000	2.84515900
C	-3.65477100	2.84816200	0.09680700
C	-2.48667200	1.69203700	-1.74719700
C	-3.78396000	3.40821200	1.36631900
H	-2.76192500	3.74555400	3.23327300
H	-0.00929500	1.66101700	2.98868800
H	0.53954900	3.27505400	2.53720300
H	-0.68946700	3.08671300	3.81699800
H	-4.51935900	2.84261300	-0.57386200
H	-1.65394500	1.00586900	-1.93938900
H	-3.42082900	1.13335500	-1.90664600
H	-2.44012600	2.48782900	-2.50994300
C	-5.09227200	3.99021500	1.83837100
H	-5.72660500	4.30407400	0.99545700
H	-5.66338100	3.24471700	2.42007800
H	-4.93598800	4.86034000	2.49498400
C	-3.19491600	-1.18974100	-0.00653500
C	-4.25692900	-0.47349500	0.63127700
C	-3.44027800	-1.73111000	-1.29547300
C	-5.47315700	-0.28924900	-0.03364400
C	-4.13985000	0.09576700	2.02685600
C	-4.68314400	-1.51882100	-1.91876100
C	-2.44879300	-2.52776100	-2.11582900
C	-5.70980200	-0.79396000	-1.31738200
H	-6.26411300	0.27680500	0.46794800
H	-4.15264100	-0.70472700	2.78271400
H	-3.20697000	0.65452700	2.16607300
H	-4.98224600	0.76867400	2.24131700
H	-4.84307800	-1.93728900	-2.91774300
H	-2.10214000	-1.94285600	-2.98451200
H	-1.56901400	-2.84413900	-1.55142200
H	-2.92771800	-3.43673300	-2.51485600
C	-7.03628200	-0.57256200	-2.00027100
H	-7.01831000	-0.90911500	-3.04738500
H	-7.84285900	-1.12194100	-1.48466700
H	-7.31782200	0.49324400	-1.98889000

s3-TS2

Bi	0.2833740	0.0330150	-1.8284900
P	2.3981240	-0.7004820	-0.1735030
P	-0.4557010	2.2668320	-0.4801830
C	3.3239250	-1.9293540	-2.7443190
H	2.6333170	-2.7784920	-2.7003140
H	4.2090920	-2.2537000	-3.3211130

H	2.8410320	-1.1289280	-3.3284830
C	3.7648170	-1.4345290	-1.3566520
C	4.5189070	-2.5431340	-0.6045390
H	4.8578600	-2.1995910	0.3857230
H	5.4125530	-2.8431900	-1.1811420
H	3.9023470	-3.4382220	-0.4472240
C	-1.8096900	3.7925620	-2.3203630
H	-2.1072970	2.8653990	-2.8357930
H	-1.8955550	4.6206630	-3.0460320
H	-2.5326900	3.9689460	-1.5099880
C	-0.3638060	3.7078180	-1.7868320
C	-4.1717440	-2.0765020	-2.2852260
H	-4.7774830	-1.7564850	-1.4236360
H	-4.6895320	-2.9300010	-2.7596840
H	-4.1374230	-1.2487540	-3.0081850
C	-2.7588240	-2.5041170	-1.8503020
C	4.7269490	-0.2460760	-1.5742850
H	4.2324770	0.5878120	-2.0977380
H	5.5849420	-0.5633620	-2.1936250
H	5.1165020	0.1419150	-0.6206360
C	0.5716010	3.5487850	-2.9957730
H	1.6292370	3.4940060	-2.7164050
H	0.4542970	4.4177350	-3.6681060
H	0.3243280	2.6523540	-3.5872350
C	-2.8672110	-3.7075100	-0.8982170
H	-3.4580800	-3.4541040	-0.0044790
H	-1.8770390	-4.0513510	-0.5676710
H	-3.3746360	-4.5480570	-1.4060880
C	-0.0328130	5.0015990	-1.0245050
H	-0.6928090	5.1401650	-0.1531810
H	-0.1714520	5.8707310	-1.6919430
H	1.0033920	5.0155570	-0.6590340
C	-1.9321960	-2.8811780	-3.0879630
H	-2.4175260	-3.7227360	-3.6119150
H	-0.9143840	-3.2026890	-2.8216910
H	-1.8584830	-2.0419170	-3.7975800
P	-1.9062930	-1.0353900	-0.9797680
C	0.7737120	2.6197260	0.8761750
C	0.1850180	2.6853190	2.1761630
C	2.1726450	2.8223790	0.7571890
C	0.9949400	2.9443920	3.2888610
C	2.9390510	3.0735870	1.9049860
C	2.3762420	3.1355620	3.1802650
H	0.5290740	2.9908380	4.2777300
H	4.0185480	3.2145150	1.7933210
C	1.8296600	-2.0550920	0.9678660
C	1.9474650	-1.7432040	2.3559590
C	1.2799090	-3.3131290	0.6054100
C	1.5333810	-2.6766470	3.3140100
C	0.8759320	-4.2110520	1.6040880
C	0.9877770	-3.9155030	2.9638450
H	1.6300520	-2.4200690	4.3733330
H	0.4457240	-5.1716800	1.3036530
C	-2.9272530	-0.3893050	0.4056780
C	-2.7399760	-0.8800250	1.7304900
C	-3.9074610	0.6121300	0.1736770
C	-3.5469590	-0.3904470	2.7634160
C	-4.6793360	1.0795690	1.2498590
C	-4.5197290	0.5938860	2.5479450
H	-3.3978710	-0.7766260	3.7763150
H	-5.4283620	1.8546350	1.0594900
C	-1.6706720	-1.8832880	2.0737300
H	-1.7751970	-2.8172970	1.5069370
H	-0.6726380	-1.4975070	1.8284140
H	-1.6848970	-2.1272860	3.1458590
C	-4.1905750	1.2043370	-1.1845950
H	-3.3614100	1.0225150	-1.8787320
H	-5.0982930	0.7552530	-1.6220090
H	-4.3648180	2.2879090	-1.1072220
C	-5.3297850	1.1374830	3.6981610
H	-6.2228820	1.6782380	3.3504060
H	-5.6564160	0.3329970	4.3764240
H	-4.7285420	1.8418600	4.2994970
C	0.4839700	-4.8704450	4.0161940
H	0.5191530	-5.9149940	3.6702390
H	1.0681330	-4.7953750	4.9465660
H	-0.5670340	-4.6460830	4.2721760
C	1.1139330	-3.7629500	-0.8223690

H	0.8616140	-2.9253370	-1.4830130
H	2.0381890	-4.2214940	-1.2097270
H	0.3196690	-4.5174830	-0.9100580
C	2.4852310	-0.4183240	2.8494670
H	3.5023160	-0.2203550	2.4787580
H	1.8695630	0.4240180	2.5042720
H	2.5040710	-0.3923090	3.9496190
C	2.9094810	2.8197120	-0.5561080
H	2.4339060	2.1679180	-1.2971870
H	2.9520520	3.8346290	-0.9866350
H	3.9430280	2.4736660	-0.4229150
C	-1.2980990	2.4985360	2.4178560
H	-1.8962890	3.2710650	1.9097890
H	-1.6652740	1.5356750	2.0398040
H	-1.5225580	2.5464110	3.4940140
C	3.2307640	3.3431650	4.4048670
H	4.1552310	3.8923640	4.1692240
H	2.6902170	3.8968910	5.1883110
H	3.5278500	2.3717040	4.8383610

s3-inv

Bi	-0.75795009	-1.10064870	1.15373280
P	0.62561991	-1.74383254	-1.08267537
P	-2.83373812	-0.80853263	-0.61211851
P	1.01868423	0.79956834	1.89086590
C	-4.34616319	-3.05449480	-0.22557135
H	-4.35083201	-3.13828962	-1.32297406
H	-5.21876794	-3.60622977	0.16674779
H	-3.44055993	-3.56429256	0.14302857
C	-4.39991712	-1.58081424	0.23778542
C	-5.65094684	-0.91864813	-0.36221037
H	-5.76052447	0.12925685	-0.05061623
H	-6.55450593	-1.46567501	-0.03851245
H	-5.62622272	-0.93443452	-1.46356921
C	-0.81860488	1.58376186	3.86027112
H	-1.68245601	1.11195974	3.36731403
H	-1.21628145	2.35573164	4.54186536
H	-0.31430701	0.82045644	4.47478123
C	0.14330192	2.22641727	2.84747614
C	-0.12088572	-4.46432585	-0.15705416
H	0.60101367	-4.45096871	0.66952926
H	-0.26717425	-5.51874021	-0.45345451
H	-1.08817094	-4.10259911	0.22618164
C	0.35388509	-3.64974422	-1.36967024
C	-4.46917374	-1.58193534	1.77400433
H	-3.59996006	-2.09165265	2.21987872
H	-5.36827501	-2.13401165	2.10221497
H	-4.52904227	-0.57688885	2.20464404
C	-0.59983818	3.24805847	1.97971323
H	0.07662425	3.73281344	1.26034436
H	-1.02949083	4.03904813	2.62076993
H	-1.42299551	2.79218558	1.41802532
C	1.66042409	-4.24986718	-1.91350216
H	2.45050151	-4.28029816	-1.14931609
H	2.05040300	-3.67803947	-2.77027425
H	1.47615003	-5.28348677	-2.25672806
C	1.26320668	2.95839281	3.61639255
H	1.81029049	2.28086384	4.28982602
H	0.82104298	3.76273349	4.23071280
H	1.98432653	3.42576627	2.92830318
C	-0.73557322	-3.71297554	-2.46098095
H	-0.96328733	-4.76600861	-2.70594594
H	-0.40922980	-3.21096698	-3.38531920
H	-1.66627107	-3.22557430	-2.13134814
C	2.00631684	1.67104187	0.57779259
C	1.50459959	2.16263787	-0.65661484
C	3.39240951	1.82954621	0.86796595
C	2.37619861	2.81470337	-1.54041126
C	4.21508294	2.49769734	-0.04867384
C	3.73312117	2.99380207	-1.26116041
H	1.97635668	3.18428334	-2.48958537
H	5.27627045	2.61753649	0.19002157
C	-3.14698920	0.97647610	-1.02972126
C	-3.55713835	2.00653141	-0.14474764
C	-2.87387940	1.31651308	-2.38857574
C	-3.68491690	3.32183854	-0.61713608
C	-3.02260580	2.64259397	-2.81109662

C	-3.42635414	3.66506108	-1.94533528
H	-3.98780534	4.10475767	0.08492043
H	-2.80440106	2.88863677	-3.85490453
C	2.45021859	-1.51662138	-0.74944412
C	3.18172943	-1.94158592	0.38731709
C	3.14158986	-0.80479065	-1.77636499
C	4.54405179	-1.61837862	0.49631709
C	4.49890932	-0.51063423	-1.61902434
C	5.22003834	-0.89369457	-0.48359192
H	5.08849863	-1.93866466	1.38997171
H	5.01007812	0.05082439	-2.40651122
C	2.47820444	-0.36340447	-3.06510851
H	2.08948361	-1.22052358	-3.63806639
H	1.62625359	0.30504894	-2.88551516
H	3.19894385	0.17140805	-3.70153758
C	2.60413078	-2.77978864	1.50223222
H	1.52089829	-2.65827335	1.61424215
H	2.79212936	-3.85181895	1.31798942
H	3.06863462	-2.52712444	2.46697550
C	6.66300468	-0.49229595	-0.31851657
H	6.73788823	0.58349829	-0.08118260
H	7.15385510	-1.04789422	0.49456390
H	7.23766714	-0.65670705	-1.24420580
C	-2.39901252	0.30119193	-3.40728593
H	-1.44117447	-0.15722905	-3.11474698
H	-3.11201619	-0.53052991	-3.51666477
H	-2.26641759	0.77614306	-4.39122037
C	-3.86517884	1.76509770	1.31040042
H	-3.78097551	2.69220732	1.89468347
H	-4.89092605	1.38743335	1.44584275
H	-3.18775034	1.02125421	1.74913516
C	-3.58733103	5.08015682	-2.44159948
H	-4.52031000	5.19077204	-3.02153690
H	-3.62629332	5.80143426	-1.61138959
H	-2.75875360	5.36907486	-3.10791121
C	0.08632581	1.95670470	-1.11653087
H	-0.04692138	0.93053652	-1.49400565
H	-0.65456469	2.08989738	-0.32528033
H	-0.17392427	2.64204087	-1.93479794
C	4.04467666	1.31599576	2.13438889
H	3.89082583	2.00670345	2.97906387
H	3.63697164	0.34310113	2.43683013
H	5.12948188	1.20683169	1.98890201
C	4.65785356	3.64035226	-2.26131460
H	5.49545451	4.15732266	-1.76778356
H	5.09470133	2.87944548	-2.93278618
H	4.12858658	4.36827263	-2.89540269

s3-rot

Bi	0.5576500	-0.4512240	-1.0669780
P	2.3003820	-0.7361530	0.9783890
P	-1.4766190	1.3828600	-1.2867250
P	-0.8828670	-2.0311030	0.6054610
C	0.3900790	2.6175540	-3.2879370
H	0.9043350	3.2714650	-2.5759210
H	0.4435040	3.1050870	-4.2779750
H	0.9599130	1.6774710	-3.3630910
C	-1.0815470	2.3635540	-2.9203290
C	-1.8563260	3.6910090	-2.8698950
H	-2.9182730	3.5310100	-2.6239790
H	-1.8109070	4.1855700	-3.8565950
H	-1.4460580	4.3854830	-2.1240990
C	1.0360450	-4.1032300	0.3458220
H	1.6112670	-3.6026490	-0.4487230
H	1.2551320	-5.1839800	0.2836590
H	1.4118620	-3.7269830	1.3082050
C	-0.4768500	-3.8825160	0.1877640
C	0.8263400	1.3135230	2.4241900
H	1.1240340	2.1086970	1.7324380
H	0.5976630	1.7893880	3.3945160
H	-0.1060740	0.8712230	2.0442360
C	1.9333700	0.2672000	2.5985990
C	-1.6922290	1.4725870	-4.0249930
H	-1.2005310	0.4871100	-4.0733640
H	-1.5654500	1.9541560	-5.0107580
H	-2.7678250	1.3041110	-3.8627680
C	-0.9204490	-4.3843170	-1.1911420

H	-2.0083940	-4.3061880	-1.3231450
H	-0.6511570	-5.4507050	-1.2979730
H	-0.4256530	-3.8371630	-2.0073890
C	3.2156310	0.9300280	3.1232520
H	3.5823660	1.7216600	2.4545150
H	4.0322600	0.2032040	3.2537460
H	3.0117140	1.3874120	4.1077640
C	-1.2134560	-4.6947950	1.2738840
H	-0.8664220	-4.4285110	2.2843820
H	-1.0175030	-5.7716630	1.1254820
H	-2.3036420	-4.5454990	1.2288890
C	1.4565450	-0.7965000	3.6114570
H	1.2199670	-0.3119580	4.5760320
H	2.2304320	-1.5586340	3.7963190
H	0.5530200	-1.3106920	3.2503630
C	-1.7403610	2.5928380	0.1072590
C	-0.8439980	3.5882310	0.5719080
C	-3.0054130	2.4672630	0.7596710
C	-1.1853070	4.3798940	1.6780220
C	-3.2958070	3.2769510	1.8646930
C	-2.3980630	4.2325370	2.3526000
H	-0.4700410	5.1322010	2.0250430
H	-4.2663160	3.1619450	2.3568680
C	-2.7171850	-1.9283270	0.2623820
C	-3.3675630	-1.9231790	-0.9972900
C	-3.5240720	-1.8614730	1.4420860
C	-4.7723980	-1.8943780	-1.0524060
C	-4.9175960	-1.8469580	1.3307780
C	-5.5679030	-1.8651240	0.0905910
H	-5.2534020	-1.8864910	-2.0358240
H	-5.5191330	-1.8018170	2.2437910
C	4.0116570	-0.2699060	0.4006940
C	4.9993960	-1.2836910	0.5659680
C	4.3952570	0.9574370	-0.1947200
C	6.3119040	-1.0452450	0.1383760
C	5.7210000	1.1488150	-0.6099340
C	6.6968230	0.1607900	-0.4575080
H	7.0622200	-1.8293120	0.2798240
H	5.9980520	2.1052060	-1.0643000
C	4.6967150	-2.6207000	1.2112410
H	3.9059990	-3.1660110	0.6744010
H	5.5974170	-3.2525830	1.2353040
H	4.3389210	-2.5032520	2.2466340
C	3.4154100	2.0779010	-0.4204630
H	2.7976150	1.8919900	-1.3156530
H	2.7265840	2.1905340	0.4230020
H	3.9289810	3.0383990	-0.5761660
C	8.1115010	0.3706500	-0.9372400
H	8.8429970	-0.0717560	-0.2424800
H	8.2683190	-0.1087390	-1.9196100
H	8.3489810	1.4393210	-1.0511660
C	-4.0847910	1.5208010	0.2839150
H	-4.4183960	1.7768940	-0.7345410
H	-3.7419980	0.4817470	0.2407310
H	-4.9590990	1.5621860	0.9501470
C	0.4791280	3.8652030	-0.0867920
H	0.8855400	2.9656090	-0.5561250
H	0.3761620	4.6307270	-0.8739290
H	1.2164640	4.2458130	0.6351670
C	-2.7259900	5.0537700	3.5741370
H	-2.5195030	4.4827290	4.4965020
H	-2.1278400	5.9766600	3.6181380
H	-3.7913590	5.3324870	3.5994260
C	-2.6715130	-1.9054400	-2.3377220
H	-1.5810640	-1.9287880	-2.2593910
H	-2.9792670	-2.7675020	-2.9513580
H	-2.9451900	-0.9912010	-2.8842910
C	-2.9345150	-1.7724420	2.8355220
H	-2.3468120	-2.6642820	3.0990360
H	-2.2537920	-0.9125670	2.9306010
H	-3.7340760	-1.6612830	3.5834780
C	-7.0728550	-1.8093760	0.0108120
H	-7.4449290	-0.8139390	0.3092020
H	-7.4359900	-2.0089750	-1.0084360
H	-7.5376970	-2.5424440	0.6900030

s3-a-Birad

Bi	-2.3212470	-0.0072970	0.0003970
P	-0.3842790	1.5662100	0.9428210
P	-0.3742160	-1.5679650	-0.9426360
C	-1.5562910	-4.0707200	0.0835210
H	-0.7373840	-4.1862740	0.8085420
H	-1.9132420	-5.0830610	-0.1788580
H	-2.3886660	-3.5457670	0.5768960
C	-1.0917930	-3.3428110	-1.1833200
C	0.0386790	-4.1595130	-1.8421890
H	0.3529490	-3.7193500	-2.8012800
H	-0.3135580	-5.1866720	-2.0436990
H	0.9214270	-4.2311640	-1.1874360
C	-1.5822530	4.0606260	-0.0852640
H	-0.7642270	4.1808350	-0.8105140
H	-1.9456260	5.0708780	0.1763500
H	-2.4113280	3.5299510	-0.5780720
C	-1.1128910	3.3367540	1.1820980
C	-2.2644040	-3.2144210	-2.1708500
H	-3.1254250	-2.6907120	-1.7231090
H	-2.6140710	-4.2174510	-2.4716070
H	-1.9714280	-2.6708880	-3.0835240
C	0.0126060	4.1611120	1.8399540
H	0.8947450	4.2376800	1.1849380
H	0.3298460	3.7238250	2.7993830
H	-0.3460070	5.1862350	2.0405910
C	-2.2844460	3.2018890	2.1700160
H	-2.6404080	4.2029760	2.4698610
H	-1.9878240	2.6611240	3.0831550
H	-3.1422190	2.6722840	1.7229750
C	0.9977430	-1.7785370	0.3141340
C	2.3137780	-1.7159130	-0.2379320
C	0.8611610	-1.9882860	1.7121010
C	3.4245710	-1.8987680	0.5927240
C	2.0120800	-2.1777990	2.4972780
C	3.2999580	-2.1380780	1.9651530
H	4.4243020	-1.8449270	0.1515280
H	1.8870470	-2.3441130	3.5718430
C	0.9864860	1.7845080	-0.3139140
C	0.8487580	1.9930710	-1.7119770
C	2.3027840	1.7301640	0.2383060
C	1.9985420	2.1896000	-2.4970270
C	3.4125460	1.9197680	-0.5922700
C	3.2866070	2.1580010	-1.9647360
H	1.8726000	2.3548890	-3.5716520
H	4.4125270	1.8722670	-0.1509250
C	2.5671520	1.4545840	1.7026420
H	2.1727820	2.2509460	2.3515390
H	2.0961860	0.5154780	2.0232830
H	3.6485060	1.3728400	1.8878160
C	-0.4549020	1.9458690	-2.4738730
H	-1.3343570	2.1149240	-1.8481340
H	-0.4631460	2.6929100	-3.2829030
H	-0.5718030	0.9507090	-2.9350580
C	4.5054200	2.3163450	-2.8382760
H	4.2651910	2.8188560	-3.7874690
H	5.2938010	2.8951830	-2.3316190
H	4.9366000	1.3304130	-3.0866330
C	-0.4428820	-1.9493950	2.4738040
H	-0.5668430	-0.9545760	2.9338970
H	-1.3210510	-2.1253650	1.8481590
H	-0.4459420	-2.6955660	3.2836690
C	2.5765890	-1.4384200	-1.7021830
H	3.6574160	-1.3495700	-1.8871570
H	2.1875730	-2.2372880	-2.3512310
H	2.0995190	-0.5024060	-2.0228060
C	4.5198350	-2.2895190	2.8384320
H	5.3081470	-2.8710010	2.3346370
H	4.9505520	-1.3016440	3.0796860
H	4.2811770	-2.7860860	3.7911160

s3-a-Prad

P	-1.0183600	-1.6044620	-0.6898490
C	-0.8106350	-3.9445460	-2.2155660
H	-1.7049580	-3.6814040	-2.8019920
H	-0.6699190	-5.0379490	-2.2907760
H	0.0587250	-3.4625170	-2.6917050
C	-0.9550600	-3.5340850	-0.7355860

C	-2.1857160	-4.2144590	-0.1235950
H	-3.1124920	-3.9376830	-0.6478160
H	-2.3186300	-3.9491780	0.9357860
H	-2.0755600	-5.3118620	-0.1867820
C	0.3147490	-3.9608400	0.0293600
H	0.4528460	-5.0542050	-0.0510710
H	0.2510130	-3.7097220	1.0997950
H	1.2192230	-3.4793200	-0.3767340
C	-2.6407550	-1.2448230	0.1260840
C	-3.8094390	-1.0654500	-0.6548480
C	-2.7132850	-1.0813250	1.5317330
C	-5.0154770	-0.7388220	-0.0210040
C	-3.9408590	-0.7543840	2.1226260
C	-5.1051800	-0.5771920	1.3663690
H	-5.9139710	-0.6056660	-0.6318860
H	-3.9882370	-0.6335530	3.2095260
C	-1.4993400	-1.2766430	2.4120180
H	-1.2004410	-2.3378740	2.4529430
H	-0.6273490	-0.7175130	2.0354940
H	-1.6959790	-0.9501360	3.4440430
C	-3.7895200	-1.2434720	-2.1563880
H	-2.9628600	-0.6836550	-2.6232980
H	-3.6496010	-2.3023940	-2.4326740
H	-4.7321680	-0.9061930	-2.6124630
C	-6.4070980	-0.1868620	2.0218470
H	-6.5054260	0.9116740	2.0787040
H	-7.2743490	-0.5620380	1.4566740
H	-6.4750980	-0.5730150	3.0507110

1

Bi	-0.0870310	0.0596880	-1.1142850
P	2.2800260	-0.0979740	0.1921380
P	-1.5826230	1.4336500	0.6734170
P	-1.0179440	-2.3339930	-0.2453580
C	-0.3474710	1.8500110	2.0503000
H	0.2149310	0.9087680	2.1695890
C	-1.0563460	2.1105610	3.3963260
H	-1.6506890	3.0394620	3.3359670
H	-1.7731320	1.3005400	3.6104700
C	0.6833960	2.9494080	1.7571920
H	1.1979490	2.7440240	0.8082410
H	0.1725130	3.9202250	1.6339980
C	-1.3090290	-2.0647630	1.6184160
H	-1.8238410	-1.0942340	1.7369840
C	2.6518520	-1.9460310	-0.0625090
H	1.7035620	-2.4314890	0.2183570
C	-0.0349890	2.2354600	4.5372850
H	-0.5530630	2.4511820	5.4873410
H	0.4720440	1.2623110	4.6700510
C	0.0161110	-2.0168540	2.4002020
H	0.5605580	-2.9676970	2.2519730
H	0.6707260	-1.2207250	2.0149830
C	1.0166490	3.3125060	4.2423100
H	1.7609410	3.3544580	5.0554940
H	0.5229190	4.3022680	4.2185920
C	1.7067230	3.0640610	2.8952310
H	2.2876620	2.1254170	2.9413290
H	2.4276190	3.8708360	2.6768700
C	3.0991600	-3.9540480	-1.5388440
H	2.1156860	-4.3984490	-1.3013440
H	3.3543110	-4.2790570	-2.5619740
C	2.9725590	-2.4239530	-1.4880980
H	3.9181610	-1.9731710	-1.8365860
H	2.1897250	-2.0912710	-2.1903120
C	-2.2236630	-3.1541470	2.2176470
H	-3.2002300	-3.1689880	1.7110830
H	-1.7649480	-4.1471670	2.0532580
C	-0.2098990	-1.7984630	3.9019440
H	-0.6685700	-0.8055800	4.0547950
H	0.7599690	-1.7781950	4.4272020
C	3.7047930	-2.4521850	0.9455680
H	4.6870170	-1.9951470	0.7317320
H	3.4306640	-2.1330170	1.9646080
C	3.8340150	-3.9813880	0.8836060
H	4.6114370	-4.3250590	1.5870940
H	2.8828330	-4.4339960	1.2200460
C	4.1437580	-4.4640600	-0.5385910

H	4.1987290	-5.5654550	-0.5677160
H	5.1424740	-4.0928320	-0.8365750
C	-2.4483290	-2.9395590	3.7228360
H	-3.0013030	-1.9934750	3.8698570
H	-3.0905640	-3.7431170	4.1220020
C	-1.1264400	-2.8729350	4.4937980
H	-0.6210170	-3.8548010	4.4337200
H	-1.3118850	-2.6799160	5.5640060
C	3.3787610	0.7517400	-1.1059760
C	3.0395380	2.2492830	-1.2328730
C	4.8715000	0.5883290	-0.7510160
H	3.2090930	0.2797370	-2.0900740
C	3.9394970	2.9558890	-2.2559330
H	3.1672110	2.7317030	-0.2470990
H	1.9811570	2.3884980	-1.5132570
C	5.7800090	1.2970360	-1.7670010
H	5.0479090	1.0069190	0.2569910
H	5.1432900	-0.4761890	-0.6968510
C	5.4223280	2.7801330	-1.9099730
H	3.6790280	4.0265580	-2.3102120
H	3.7464300	2.5344090	-3.2596830
H	6.8362510	1.1805900	-1.4703750
H	5.6744750	0.8014600	-2.7498650
H	6.0574550	3.2596410	-2.6738120
H	5.6340090	3.2969540	-0.9555170
C	-1.8312590	3.0711550	-0.2512710
C	-2.6312560	2.8390410	-1.5489450
C	-2.5729990	4.0862640	0.6436980
H	-0.8511940	3.5026390	-0.5217970
C	-2.9206720	4.1516430	-2.2902450
H	-3.5868570	2.3418280	-1.2980310
H	-2.0910250	2.1502300	-2.2202740
C	-2.8595540	5.4008300	-0.0969600
H	-3.5266950	3.6373650	0.9778030
H	-1.9931800	4.2954430	1.5552390
C	-3.6445680	5.1593120	-1.3905380
H	-3.5130210	3.9478980	-3.1983990
H	-1.9649110	4.5907100	-2.6315660
H	-3.4074510	6.0927120	0.5651130
H	-1.9001070	5.8939460	-0.3402110
H	-3.8084980	6.1089230	-1.9274010
H	-4.6459430	4.7642390	-1.1374220
C	-2.8245700	-2.2775340	-0.8649330
C	-3.0800580	-1.4433100	-2.1334060
C	-3.3087850	-3.7274440	-1.0929720
H	-3.4222930	-1.8246940	-0.0539680
C	-4.5516300	-1.5038010	-2.5710530
H	-2.4377810	-1.8091790	-2.9563220
H	-2.8082360	-0.3896140	-1.9613240
C	-4.7833670	-3.7837430	-1.5152410
H	-2.6846570	-4.1845560	-1.8826200
H	-3.1498610	-4.3386460	-0.1910890
C	-5.0331480	-2.9442980	-2.7724040
H	-4.6909250	-0.9155030	-3.4940450
H	-5.1721480	-1.0193230	-1.7946330
H	-5.0878580	-4.8310730	-1.6813920
H	-5.4120860	-3.4012140	-0.6897370
H	-6.1021900	-2.9587320	-3.0441970
H	-4.4865730	-3.3945280	-3.6218550

1-Birad

Bi	-0.0642680	-0.4076110	1.8615670
P	1.0675730	-0.1790720	-0.5743040
P	-2.5317100	0.0335750	0.8450380
C	1.7606440	1.5919860	-0.4482520
H	0.9190070	2.1590610	-0.0121590
C	2.0067700	2.1935610	-1.8490420
H	2.8071580	1.6323660	-2.3620000
H	1.1024940	2.0769660	-2.4690680
C	2.9751910	1.8087210	0.4691220
H	2.7665890	1.4163960	1.4788840
H	3.8387860	1.2382010	0.0856550
C	-2.1518710	1.5926260	-0.1856980
H	-1.2393640	1.3946900	-0.7760530
C	2.4063520	3.6741140	-1.7625000
H	2.6133320	4.0689270	-2.7715840
H	1.5518260	4.2550110	-1.3692400

C	-1.8955550	2.7984560	0.7382360
H	-2.7877820	2.9632460	1.3698810
H	-1.0650900	2.5836110	1.4333910
C	3.6166610	3.8808040	-0.8444210
H	3.8661900	4.9527220	-0.7710530
H	4.4986980	3.3836340	-1.2898730
C	3.3608200	3.2934840	0.5487130
H	2.5404450	3.8539850	1.0341420
H	4.2491140	3.4198090	1.1907730
C	-3.2710270	1.9261830	-1.1917740
H	-3.4119580	1.0953530	-1.8992100
H	-4.2277170	2.0509570	-0.6510490
C	-1.5889070	4.0783540	-0.0508130
H	-0.6372050	3.9466280	-0.5971160
H	-1.4355590	4.9217440	0.6436180
C	-2.9577860	3.2071800	-1.9807940
H	-2.0602430	3.0334240	-2.6031430
H	-3.7836850	3.4284370	-2.6780590
C	-2.7016840	4.3992890	-1.0536120
H	-3.6301880	4.6361490	-0.5016520
H	-2.4469490	5.2983110	-1.6399780
C	2.5915900	-1.2962800	-0.4021270
C	2.1484710	-2.7662520	-0.2544080
C	3.5214310	-1.1488110	-1.6247210
H	3.1641560	-1.0164700	0.5000660
C	3.3435970	-3.7267510	-0.1881220
H	1.5083030	-3.0372550	-1.1146010
H	1.5231740	-2.8916820	0.6460940
C	4.7171070	-2.1105220	-1.5561830
H	2.9395930	-1.3504270	-2.5431050
H	3.8870410	-0.1143170	-1.7100780
C	4.2630450	-3.5658640	-1.4036450
H	2.9851910	-4.7670020	-0.1082680
H	3.9203440	-3.5224600	0.7329240
H	5.3446380	-1.9924490	-2.4558870
H	5.3518830	-1.8355760	-0.6934270
H	5.1346880	-4.2367700	-1.3208530
H	3.7159730	-3.8718040	-2.3146920
C	-2.6489160	-1.2737810	-0.5395110
C	-2.0312640	-2.6396420	-0.1882080
C	-4.1331050	-1.4735020	-0.9233690
H	-2.0987520	-0.8742420	-1.4101580
C	-2.1906910	-3.6495580	-1.3337460
H	-2.5114350	-3.0383650	0.7252260
H	-0.9596930	-2.5295920	0.0411410
C	-4.2940730	-2.4697220	-2.0807730
H	-4.6771800	-1.8511720	-0.0383250
H	-4.6078100	-0.5156160	-1.1837830
C	-3.6568200	-3.8222300	-1.7450320
H	-1.7535710	-4.6196950	-1.0415150
H	-1.6078150	-3.2921520	-2.2026490
H	-5.3630680	-2.5953190	-2.3234200
H	-3.8132540	-2.0538540	-2.9856570
H	-3.7391390	-4.5139610	-2.6005150
H	-4.2147780	-4.2863110	-0.9106100

1-Prad

P	-2.1703160	2.2156660	-0.1663400
C	-0.8630250	2.5539630	1.1475210
H	0.1055450	2.6700120	0.6244040
C	-0.7384170	1.3694030	2.1257140
H	-1.7168510	1.1994810	2.6133210
H	-0.5028160	0.4439300	1.5739500
C	-1.1380160	3.8633030	1.9211390
H	-1.1876120	4.7195470	1.2279870
H	-2.1305700	3.7936760	2.4033010
C	0.3236080	1.6296390	3.2034480
H	1.3176570	1.6908490	2.7225220
H	0.3679140	0.7786800	3.9041300
C	-0.0713580	4.1201900	2.9962390
H	0.9038840	4.2902990	2.5034490
H	-0.3086950	5.0451940	3.5488530
C	0.0509060	2.9346360	3.9610170
H	-0.8924440	2.8328070	4.5291500
H	0.8444560	3.1226830	4.7037560
C	-1.5540530	3.3774510	-1.5154730
C	-0.3690750	2.7777980	-2.3058680

C	-2.6946710	3.7698020	-2.4749630
H	-1.1937850	4.2976020	-1.0167060
C	0.1151000	3.7303580	-3.4094040
H	-0.6892230	1.8237800	-2.7639550
H	0.4649590	2.5332260	-1.6271600
C	-2.2084110	4.7170800	-3.5810060
H	-3.1087210	2.8538710	-2.9371270
H	-3.5221400	4.2336770	-1.9122970
C	-1.0267560	4.1209820	-4.3553770
H	0.9415860	3.2648280	-3.9729230
H	0.5287740	4.6432330	-2.9420630
H	-3.0387650	4.9552180	-4.2671950
H	-1.8960340	5.6745130	-3.1239420
H	-0.6681970	4.8314510	-5.1192010
H	-1.3681600	3.2210530	-4.9000200

3

Bi	-0.0002740	-0.0002280	-0.6303610
P	-2.2670280	-1.0443170	0.4767270
P	2.0359610	-1.4407890	0.4817470
P	0.2278300	2.4835800	0.4808440
C	1.7386490	-2.7487020	-2.1827830
H	1.1642200	-3.5828720	-1.7602080
H	2.2771250	-3.1297800	-3.0693270
H	1.0233280	-1.9937880	-2.5439270
C	1.3335950	-2.8779230	1.5665100
C	2.7460130	-2.1579420	-1.1798150
C	2.5354170	-3.5671380	2.2519270
H	3.1625410	-4.1332800	1.5519960
H	3.1762290	-2.8377750	2.7719340
C	0.4785730	-3.9249320	0.8395120
H	-0.3799970	-3.4676560	0.3270810
H	1.0647950	-4.4985280	0.1069390
C	1.8175130	2.5916250	1.5749820
C	-3.1564830	0.2766150	1.5720500
C	3.8082150	-3.2255700	-0.8573780
H	4.5209570	-2.8819110	-0.0912060
H	4.3839450	-3.4573830	-1.7710230
H	3.3556610	-4.1663640	-0.5130110
C	-0.8882720	3.4790870	-1.8442990
H	-1.2513090	2.4694770	-2.0976860
H	-0.8437970	4.0528460	-2.7867300
H	-1.6397550	3.9522850	-1.1929540
C	0.5058100	3.4551670	-1.1799430
C	-3.2525550	-0.1122260	-2.1799670
H	-3.6855580	0.8001650	-1.7505790
H	-3.8531600	-0.3806400	-3.0678500
H	-2.2409880	0.1303850	-2.5403570
C	1.6538630	1.5356070	2.6851050
H	0.6987860	1.6446440	3.2219450
H	1.7251620	0.5153200	2.2887030
C	-3.2457680	-1.2870340	-1.1852420
C	3.4617740	-0.9574110	-1.8369020
H	2.7671020	-0.1394050	-2.0888620
H	3.9403790	-1.2771940	-2.7792500
H	4.2440110	-0.5435560	-1.1814330
C	1.5243110	2.8736290	-2.1770210
H	2.5308780	2.7880920	-1.7482790
H	1.5936560	3.5304650	-3.0629620
H	1.2254430	1.8782760	-2.5403180
C	-3.6365830	1.5454210	0.8540920
H	-4.4275230	1.3295950	0.1211980
H	-2.8118410	2.0636620	0.3441520
C	1.8123540	3.9776890	2.2591700
H	1.9974760	4.8026330	1.5598270
H	0.8567300	4.1716450	2.7713710
C	-4.3531510	-0.4231560	2.2558660
H	-5.1587890	-0.6783250	1.5561270
H	-4.0412650	-1.3462690	2.7693390
C	-4.7017600	-1.6724250	-0.8633690
H	-5.2877770	-0.8115440	-0.5112980
H	-4.7608520	-2.4672570	-0.1032010
H	-5.1927800	-2.0470010	-1.7790780
C	0.9027820	4.9076790	-0.8565370
H	0.2462680	5.3560550	-0.0943260
H	0.8228750	5.5217600	-1.7710990
H	1.9421460	4.9825830	-0.5065400

C	-2.5672640	-2.5038420	-1.8518750
H	-3.0859970	-2.7514370	-2.7947230
H	-2.5997460	-3.3924420	-1.2021520
H	-1.5117880	-2.3109940	-2.1050090
H	-4.0614100	2.2500430	1.5916980
H	-4.7820900	0.2554330	3.0144600
H	2.1593670	-4.2819890	3.0052470
H	0.0770850	-4.6485990	1.5717330
H	2.6135460	4.0075370	3.0189100
H	2.4651170	1.6563800	3.4242250
C	3.1555000	2.3715990	0.8557750
H	3.1906410	1.3981880	0.3458920
H	3.3640360	3.1641640	0.1225300
H	3.9789160	2.3866050	1.5925250
C	0.4955260	-2.2147330	2.6764790
H	1.0621830	-1.4397250	3.2154860
H	-0.4254620	-1.7709990	2.2789990
H	0.1974290	-2.9804990	3.4138900
C	-2.1604350	0.6634840	2.6821950
H	-1.7727290	-0.2180760	3.2158020
H	-1.3155070	1.2401780	2.2863910
H	-2.6723640	1.3012460	3.4239750

3-Birad

Bi	0.0368900	0.0757820	-1.3196680
P	1.5923310	-0.0388410	0.9163180
P	-2.5014300	0.0110210	-0.3954870
C	3.2075990	1.8352170	-0.7376450
H	3.8362240	1.0319610	-1.1420060
H	3.8138430	2.7592090	-0.7327610
H	2.3762120	2.0021210	-1.4411840
C	2.6761000	-1.6249180	0.6340090
C	2.7030730	1.5394010	0.6854970
C	3.3963590	-1.9265310	1.9668840
H	4.1392780	-1.1623870	2.2302390
H	2.6805860	-2.0037200	2.8001180
C	3.7010620	-1.5653390	-0.5070430
H	3.2282190	-1.3305490	-1.4738270
H	4.4880280	-0.8209790	-0.3208140
C	-2.6842550	-1.6256460	0.6155710
C	3.9074470	1.4548430	1.6418990
H	3.5990030	1.2073540	2.6699360
H	4.4220700	2.4316740	1.6729880
H	4.6471360	0.7103430	1.3143180
C	-2.3919520	2.7634280	-0.1615390
H	-1.3284610	2.8130840	-0.4465420
H	-2.6309840	3.7006280	0.3704210
H	-2.9906290	2.7366120	-1.0861470
C	-2.6899610	1.5562260	0.7530210
C	-2.2033460	-2.7486400	-0.3263930
H	-2.6768580	-2.6827270	-1.3195480
H	-1.1121220	-2.7330870	-0.4641930
C	1.8119810	2.7140100	1.1409180
H	0.9247050	2.8343760	0.4989720
H	2.3837380	3.6571710	1.0884680
H	1.4634700	2.5857150	2.1772260
C	-1.7860560	1.6285210	1.9926540
H	-2.0830650	0.9050640	2.7623180
H	-1.8589740	2.6338810	2.4458550
H	-0.7313390	1.4399790	1.7550540
C	-4.1899060	-1.8483470	0.8738550
H	-4.6088930	-1.1090600	1.5701760
H	-4.7741480	-1.8075260	-0.0589590
C	-4.1657780	1.6458660	1.1925390
H	-4.8572860	1.5658310	0.3387850
H	-4.3444900	2.6188380	1.6840070
H	-4.4264980	0.8647520	1.9208820
H	3.9304050	-2.8906700	1.8889070
H	4.2004040	-2.5455210	-0.6122670
H	-4.3415300	-2.8448510	1.3259750
H	-2.4633850	-3.7309550	0.1051330
C	-1.9153990	-1.7081610	1.9422990
H	-0.8649890	-1.4007960	1.8379170
H	-2.3715630	-1.0758260	2.7163680
H	-1.9357460	-2.7464180	2.3206550
C	1.7002890	-2.7878770	0.3660250
H	0.8996000	-2.8393340	1.1201800

H	1.2310960	-2.7131000	-0.6278900
H	2.2479940	-3.7460330	0.3958840

3-Prad

P	2.2069110	0.4503400	-0.3792160
C	3.5030890	-1.2930950	-2.2242790
H	3.6030970	-2.1273800	-1.5165170
H	4.2745890	-1.4255440	-3.0046220
H	2.5191810	-1.3785200	-2.7131460
C	2.4480990	-0.4845560	1.2757460
C	3.6819820	0.0804460	-1.5448000
C	3.4368690	0.2732070	2.1859380
H	4.4649740	0.2499890	1.7994080
H	3.1450700	1.3283870	2.3116850
C	2.8837670	-1.9516170	1.1273050
H	2.2026120	-2.5167110	0.4713990
H	3.9028500	-2.0476240	0.7268470
C	5.0648390	0.1644980	-0.8779390
H	5.2178750	1.1314820	-0.3729330
H	5.8534980	0.0631850	-1.6451860
H	5.2241440	-0.6355450	-0.1412750
C	3.5869930	1.1785040	-2.6274050
H	2.6090650	1.1671360	-3.1358740
H	4.3656840	1.0200720	-3.3941910
H	3.7330950	2.1843340	-2.2014510
H	3.4489740	-0.1901500	3.1893600
H	2.8744350	-2.4433750	2.1167540
C	1.0504360	-0.4474330	1.9329500
H	0.6855180	0.5850460	2.0586550
H	0.3049460	-0.9978210	1.3364990
H	1.0921970	-0.9125730	2.9336310

4

Bi	0.14396700	0.15844800	-0.71300900
P	-1.26699800	-1.93598300	0.41800400
P	2.67989900	-0.21152200	0.23482600
C	2.67062100	-1.06247400	1.94705400
C	2.28087800	-2.55671600	1.98950200
C	4.08190800	-0.88933000	2.58103800
C	1.67948100	-0.26370200	2.82921300
H	2.99206200	-3.14988000	1.39476000
H	1.28349900	-2.69690100	1.54556700
C	2.29329700	-3.07758000	3.44246900
H	4.36707000	0.17616600	2.56675200
H	4.84195900	-1.42935500	1.99822500
C	4.08833200	-1.41798100	4.03254300
H	0.67187200	-0.33210300	2.40327300
H	1.94373800	0.80553400	2.82908900
C	1.67215500	-0.79082000	4.27601500
H	2.01390200	-4.14519100	3.44078800
C	3.70363700	-2.90980700	4.03776300
C	1.28079700	-2.28000800	4.28336700
H	5.10228700	-1.29381600	4.45024400
C	3.07775000	-0.62469400	4.88091000
H	0.93885700	-0.20976800	4.86172600
H	4.43462200	-3.49160900	3.44920100
H	3.73262600	-3.30479300	5.06823000
H	0.26702900	-2.40962400	3.87025200
H	1.25756400	-2.66140300	5.31915300
H	3.09143300	-0.98476700	5.92438800
H	3.35669100	0.44294500	4.90274400
C	-2.17901500	-2.56220300	-1.16588000
C	-1.07355200	-3.08952900	-2.12239600
C	-3.06826400	-3.77764600	-0.78581900
C	-3.03048300	-1.53886400	-1.95862600
H	-0.43110500	-3.81090500	-1.59125200
H	-0.42500800	-2.25706300	-2.44013400
C	-1.67953300	-3.74172400	-3.38060100
H	-3.87938500	-3.46939800	-0.11171300
H	-2.46369000	-4.52181400	-0.24004000
C	-3.68680300	-4.42212900	-2.04405800
H	-2.39814300	-0.69161700	-2.27233500
H	-3.82977000	-1.12190000	-1.33371200
C	-3.64895500	-2.19450600	-3.21228600
H	-0.85792800	-4.09482300	-4.02720600
C	-2.56668000	-4.92900600	-2.96872100
C	-2.52719700	-2.70186700	-4.13609200

H	-4.32375100	-5.26763000	-1.73243400
C	-4.53990600	-3.37670200	-2.78726200
H	-4.25727400	-1.44236400	-3.74362700
H	-1.96233100	-5.69175800	-2.44816000
H	-2.99762800	-5.41222200	-3.86276300
H	-1.89278000	-1.85826700	-4.45920900
H	-2.95678600	-3.15067000	-5.04845700
H	-5.01336500	-3.83379100	-3.67339500
H	-5.35574600	-3.02032100	-2.13432700
C	-2.59753200	-1.47529800	1.74529600
C	-3.99028100	-1.01109000	1.26066200
C	-2.79060800	-2.76088000	2.60617400
C	-2.01244300	-0.38588000	2.67758700
H	-4.45534800	-1.78071600	0.62961600
H	-3.89823000	-0.10874700	0.64677700
C	-4.92457400	-0.73017300	2.45776000
H	-1.81188800	-3.11068200	2.97297800
H	-3.20568300	-3.57670500	1.99476200
C	-3.73079900	-2.48559800	3.80006600
H	-1.82270500	0.54442600	2.12350500
H	-1.04166900	-0.72060200	3.06743500
C	-2.94845200	-0.10314700	3.86811900
H	-5.90311200	-0.39706500	2.07070000
C	-5.10299200	-2.01996100	3.27921500
C	-4.31685900	0.36905900	3.34701200
H	-3.84869800	-3.41726100	4.37970600
C	-3.12388100	-1.38976400	4.69448600
H	-2.49429900	0.68454200	4.49295400
H	-5.55856900	-2.80720000	2.65332600
H	-5.79040100	-1.84215100	4.12439100
H	-4.20155300	1.30398000	2.77187400
H	-4.99205800	0.59293700	4.19120200
H	-3.77835000	-1.20152500	5.56336200
H	-2.14790400	-1.72038600	5.08988700
P	-0.36602600	2.50731500	0.62702600
C	1.17976900	3.58140400	0.18725200
C	1.86172300	3.30853800	-1.17695800
C	0.82905000	5.09061900	0.26138900
C	2.20734500	3.28875100	1.31514200
H	1.16578300	3.50914500	-2.00482800
H	2.15736300	2.25156600	-1.24778100
C	3.12704000	4.17796700	-1.33913700
H	0.34095500	5.31862500	1.22423000
H	0.12016800	5.35945200	-0.53495600
C	2.09657300	5.95838900	0.10078000
H	2.49122300	2.22800600	1.28753500
H	1.74362800	3.48353800	2.29711300
C	3.47541100	4.14819500	1.14909600
H	3.58504600	3.95829800	-2.31909800
C	2.74332100	5.66661000	-1.26597200
C	4.12406400	3.84281600	-0.21424600
H	1.80406300	7.02129500	0.15438100
C	3.09803200	5.63736000	1.22389700
H	4.18176100	3.89900900	1.95959900
H	2.04039300	5.91805900	-2.07967900
H	3.63733000	6.29895700	-1.40561300
H	4.40386900	2.77730100	-0.26449300
H	5.04810800	4.43456200	-0.33709300
H	3.99804200	6.26871100	1.12201700
H	2.65126300	5.86677000	2.20690500
C	-1.85239700	3.12099800	-0.44107700
C	-2.92431600	2.00763800	-0.35557800
C	-2.47641400	4.37874600	0.23125900
C	-1.58605700	3.43089100	-1.93152800
H	-3.13543100	1.79328400	0.70254400
H	-2.54869100	1.07350700	-0.79958500
C	-4.22536700	2.40413800	-1.07406400
H	-1.77559300	5.22289600	0.21257900
H	-2.68544500	4.16243000	1.29264900
C	-3.77706500	4.79602900	-0.48992600
H	-1.15148300	2.54895800	-2.43176100
H	-0.85196200	4.24428600	-2.02849600
C	-2.89281400	3.84100800	-2.64429400
H	-4.94211000	1.56801600	-0.99763500
C	-4.81026900	3.65955200	-0.40576200
C	-3.91616200	2.69347500	-2.55395600
H	-4.17801400	5.70038800	-0.00107200
C	-3.46867400	5.10002600	-1.96854800

H	-2.67016400	4.05692600	-3.70331300
H	-5.05583700	3.44628300	0.64906200
H	-5.74957200	3.95721400	-0.90339000
H	-3.51385000	1.78689700	-3.03922400
H	-4.84146500	2.96385700	-3.09144400
H	-4.38658100	5.42470900	-2.48861600
H	-2.74631700	5.93162000	-2.04184600
C	3.37342000	-1.40665900	-1.09533800
C	3.54522800	-0.53411200	-2.37202500
C	4.78054500	-1.90684800	-0.67616700
C	2.50727100	-2.63244500	-1.46441300
H	4.16468800	0.34823000	-2.13799800
H	2.56426700	-0.15255400	-2.70541800
C	4.19039200	-1.34446900	-3.51313100
H	4.70494300	-2.54689000	0.21550900
H	5.42192300	-1.05029700	-0.40718300
C	5.43258800	-2.71690300	-1.81724300
H	1.51372600	-2.29719300	-1.78924400
H	2.34735600	-3.27690900	-0.58983100
C	3.15899800	-3.44611200	-2.60115000
H	4.28930400	-0.69691700	-4.40111500
C	5.58122000	-1.83052900	-3.06665300
C	3.30263100	-2.55719900	-3.84976200
H	6.42710700	-3.06049900	-1.48470300
C	4.54849600	-3.93347400	-2.15103700
H	2.51597700	-4.31266000	-2.83331300
H	6.23040800	-0.96630600	-2.84339200
H	6.06655900	-2.39768500	-3.87987700
H	2.30777700	-2.21682400	-4.18664600
H	3.74518900	-3.13333600	-4.68086600
H	5.01816600	-4.53701100	-2.94706700
H	4.45197100	-4.58605300	-1.26591400

4-Birad

Bi	0.0091980	0.1203640	-1.7469130
P	-2.2015710	-1.2622830	-1.0540900
C	-3.5001080	0.1185470	-0.7097850
C	-3.8764080	0.6829190	-2.1085110
C	-4.7798680	-0.4972740	-0.0872750
C	-3.0473080	1.3034820	0.1753500
H	-4.2055480	-0.1394810	-2.7653510
H	-2.9880080	1.1343530	-2.5849770
C	-4.9884210	1.7449080	-1.9945800
H	-4.5606090	-0.8858290	0.9183520
H	-5.1258020	-1.3492950	-0.6966340
C	-5.8981110	0.5608640	0.0234060
H	-2.1577340	1.7730660	-0.2693770
H	-2.7518290	0.9536830	1.1742850
C	-4.1658100	2.3588410	0.2985030
H	-5.2243110	2.1258060	-3.0029490
C	-6.2429330	1.1024830	-1.3752220
C	-4.5083540	2.9032840	-1.1003220
H	-6.7899480	0.0885950	0.4698470
C	-5.4180980	1.7159250	0.9218910
H	-3.8092030	3.1820760	0.9416710
H	-6.6066280	0.2835580	-2.0195700
H	-7.0569640	1.8448210	-1.3058360
H	-3.6202830	3.3837160	-1.5465790
H	-5.2919210	3.6774250	-1.0280200
H	-6.2178080	2.4684550	1.0340290
H	-5.1866850	1.3381650	1.9331060
C	-1.8591920	-2.2732020	0.5362370
C	-1.7222710	-1.4793320	1.8543550
C	-2.9876190	-3.3326270	0.7029310
C	-0.5414440	-3.0531710	0.2820990
H	-2.6599530	-0.9453390	2.0732610
H	-0.9265850	-0.7264640	1.7553080
C	-1.4016540	-2.4316940	3.0262350
H	-3.0910650	-3.9145970	-0.2283480
H	-3.9566130	-2.8469890	0.8833020
C	-2.6736160	-4.2770570	1.8842170
H	0.2904560	-2.3474510	0.1661320
H	-0.6212610	-3.6269820	-0.6579100
C	-0.2212850	-3.9986660	1.4551650
H	-1.3054880	-1.8383470	3.9519210
C	-2.5426790	-3.4544880	3.1805120
C	-0.0791040	-3.1697340	2.7459040

H	-3.4991570	-5.0024190	1.9862290
C	-1.3557940	-5.0246550	1.6159150
H	0.7285840	-4.5188980	1.2414190
H	-3.4922130	-2.9336000	3.3962320
H	-2.3417950	-4.1242510	4.0348090
H	0.7404990	-2.4404130	2.6357090
H	0.1745840	-3.8294630	3.5941610
H	-1.1329840	-5.7154080	2.4478900
H	-1.4467440	-5.6368400	0.7019500
P	1.5685140	0.0463030	0.4876500
C	3.2459690	-0.5475830	-0.2478840
C	3.6972200	0.0595020	-1.5995840
C	4.3773720	-0.3274970	0.7901120
C	3.0782390	-2.0812490	-0.4455700
H	3.8288070	1.1469330	-1.5130510
H	2.9209650	-0.1077900	-2.3653790
C	5.0183260	-0.5830320	-2.0756140
H	4.0804680	-0.7504960	1.7649340
H	4.5470540	0.7489790	0.9433850
C	5.6945250	-0.9748010	0.3130660
H	2.2823990	-2.2810180	-1.1836380
H	2.7570890	-2.5463090	0.5015180
C	4.3926750	-2.7264830	-0.9261180
H	5.3130270	-0.1236060	-3.0346750
C	6.1173480	-0.3398850	-1.0247030
C	4.8149930	-2.0969920	-2.2662210
H	6.4741520	-0.7950640	1.0730880
C	5.4914840	-2.4886220	0.1253190
H	4.2310430	-3.8099810	-1.0577480
H	6.2840270	0.7436800	-0.8939980
H	7.0731010	-0.7735600	-1.3664350
H	4.0417080	-2.2816920	-3.0318310
H	5.7468580	-2.5640340	-2.6294240
H	6.4355400	-2.9624540	-0.1951120
H	5.2054980	-2.9543060	1.0842570
C	1.7093120	1.8881650	1.0258390
C	0.2634880	2.4404390	1.1249260
C	2.3094600	1.9133620	2.4615130
C	2.5263570	2.8416800	0.1267430
H	-0.3583840	1.7713120	1.7430960
H	-0.1935520	2.4598420	0.1223650
C	0.2451840	3.8680170	1.7022130
H	3.3392980	1.5289660	2.4578790
H	1.7244600	1.2459690	3.1161800
C	2.3062510	3.3478400	3.0306940
H	2.1130810	2.8421530	-0.8964360
H	3.5685320	2.4971420	0.0516390
C	2.5127390	4.2756650	0.7008520
H	-0.8001980	4.2187450	1.7492560
C	0.8596380	3.8653140	3.1127890
C	1.0642840	4.7918070	0.7817040
H	2.7537890	3.3315670	4.0391790
C	3.1329220	4.2664300	2.1110380
H	3.1044140	4.9314510	0.0393080
H	0.2649840	3.2221190	3.7841730
H	0.8404130	4.8833920	3.5390100
H	0.6142190	4.8176870	-0.2259340
H	1.0498920	5.8256720	1.1679540
H	3.1587470	5.2909410	2.5209210
H	4.1777660	3.9129880	2.0629700

4-Prad

P	0.0000110	1.6941020	-0.0000860
C	1.5534210	0.5957310	-0.0090140
C	1.8810170	0.0690750	1.4164630
C	1.5290290	-0.5990220	-0.9935400
C	2.7073370	1.5476450	-0.4401510
H	1.0916550	-0.6124290	1.7659040
H	1.9127940	0.9122670	2.1277450
C	3.2353340	-0.6717240	1.4204000
H	1.3066180	-0.2418510	-2.0134960
H	0.7321750	-1.3067010	-0.7203330
C	2.8848000	-1.3376490	-0.9818310
H	2.7475980	2.4170550	0.2390420
H	2.5039520	1.9445150	-1.4497820
C	4.0618950	0.8124690	-0.4309610
H	3.4369060	-1.0430810	2.4398510

C	3.1714060	-1.8582900	0.4397120
C	4.3546620	0.2931360	0.9888190
H	2.8353590	-2.1869400	-1.6847760
C	4.0051270	-0.3731960	-1.4118440
H	4.8543720	1.5147510	-0.7412480
H	2.3820230	-2.5648050	0.7508280
H	4.1246030	-2.4146560	0.4545310
H	4.4194640	1.1382510	1.6958440
H	5.3307490	-0.2220390	1.0115950
H	4.9748090	-0.9003850	-1.4287210
H	3.8184660	-0.0087240	-2.4368550
C	-1.5533420	0.5955880	0.0089190
C	-2.7074890	1.5482210	0.4379010
C	-1.8799810	0.0668690	-1.4160170
C	-1.5297890	-0.5977280	0.9952190
H	-2.7471280	2.4166800	-0.2425320
H	-2.5047720	1.9464720	1.4471280
C	-4.0621170	0.8132180	0.4287350
H	-1.0904610	-0.6153050	-1.7639430
H	-1.9112040	0.9089530	-2.1286160
C	-3.2343390	-0.6737720	-1.4198510
H	-1.3081270	-0.2391850	2.0147700
H	-0.7328650	-1.3058180	0.7236160
C	-2.8856550	-1.3361900	0.9836340
H	-4.8547470	1.5160640	0.7374290
C	-4.3538700	0.2918100	-0.9904930
C	-4.0061980	-0.3709980	1.4114020
H	-3.4351720	-1.0466060	-2.4389080
C	-3.1712380	-1.8588630	-0.4373580
H	-2.8368350	-2.1844270	1.6878890
H	-4.4180940	1.1358220	-1.6987970
H	-5.3299150	-0.2233100	-1.0132170
H	-3.8202440	-0.0050150	2.4360210
H	-4.9759910	-0.8980350	1.4283490
H	-4.1244620	-2.4151280	-0.4520290
H	-2.3817480	-2.5659030	-0.7468620

Bi(PCy*t*Bu)2_cation

Bi	-0.63319300	-1.91803300	-0.40853900
P	-1.85004600	0.17637700	0.12363000
P	1.97838000	-1.37847700	-0.14171800
C	-3.67203700	1.03943700	-1.83518700
H	-3.20039500	2.03151400	-1.85691000
H	-4.72248600	1.16390400	-2.14842300
H	-3.17211000	0.39985600	-2.57779300
C	-0.96800400	1.70308300	0.71258500
H	0.07132300	1.34100300	0.72522900
C	-3.65272200	0.40559300	-0.42846500
C	-1.32213700	2.09773000	2.16557400
H	-2.36390300	2.45139400	2.21553300
H	-1.24998500	1.22290700	2.83146100
C	-1.03298900	2.91299100	-0.24100500
H	-0.76235400	2.61415400	-1.26573800
H	-2.06364100	3.30286600	-0.27869600
C	2.26108200	0.41239300	-0.67934900
H	1.73115100	1.10093200	-0.00311200
C	-4.37934600	1.30957900	0.58465400
H	-4.35178800	0.89913400	1.60532600
H	-5.43722000	1.38727600	0.28421600
H	-3.97064600	2.32961800	0.60217600
C	1.55797900	-2.70035800	2.31315300
H	0.47153800	-2.51658600	2.37148500
H	1.89931200	-2.90732500	3.34088800
H	1.73325500	-3.61498200	1.72305200
C	2.32458300	-1.48791600	1.74978300
C	-0.38002600	3.21391800	2.64338700
H	-0.65481600	3.50857600	3.66871000
H	0.64993000	2.81638700	2.69685000
C	1.73401200	0.62523900	-2.11451000
H	2.20092300	-0.11950900	-2.78407200
H	0.64596700	0.44695400	-2.15599400
C	-4.31690200	-0.98182200	-0.46779400
H	-3.83826000	-1.65466900	-1.19747600
H	-5.36551700	-0.86202400	-0.78503900
H	-4.31931100	-1.47001800	0.51864600
C	1.93191000	-0.21908000	2.51710700
H	2.47424100	0.67128500	2.16596000

H	2.17189300	-0.34498500	3.58655100
H	0.85158200	-0.01886900	2.44545500
C	-0.41457300	4.42514900	1.70424400
H	0.29175300	5.19624100	2.05004100
H	-1.41908400	4.88448400	1.73916200
C	-0.09410800	4.02189000	0.26052300
H	0.94942300	3.66241000	0.20141500
H	-0.16259400	4.89096400	-0.41311600
C	3.76683600	0.75633900	-0.60227600
H	4.12751700	0.68037600	0.43341200
H	4.34177200	0.02164800	-1.19409400
C	2.03667900	2.03601500	-2.63967800
H	1.47336000	2.77518800	-2.04221100
H	1.67213900	2.12824300	-3.67561100
C	3.83688200	-1.77046500	1.90313900
H	4.13344000	-2.69071400	1.37712300
H	4.07003700	-1.90414600	2.97295500
H	4.46485200	-0.94962700	1.53229300
C	4.04623400	2.17229600	-1.12927000
H	3.55710300	2.90772200	-0.46356900
H	5.12801200	2.37421700	-1.07429000
C	3.53086600	2.36005500	-2.55801100
H	4.08801900	1.69152300	-3.23881500
H	3.72027600	3.38856600	-2.90457200

PCy*t*Bu_anion

P	-1.09786100	-0.59095600	-1.23649100
C	-2.30772400	1.63174800	0.22527200
H	-1.34528000	2.13467500	0.40519000
H	-3.02115900	1.99479800	1.00114300
H	-2.66629500	1.96582000	-0.76121800
C	0.73661900	-0.28833000	-0.76676400
H	1.20841800	-0.49481200	-1.75173600
C	-2.15738500	0.09469300	0.24804700
C	1.38376600	-1.29092700	0.21955600
H	0.95131300	-1.14968100	1.22561800
H	1.11127000	-2.31379900	-0.08703300
C	1.16409400	1.14554200	-0.38932600
H	0.74119300	1.85130500	-1.12317000
H	0.72227400	1.41249900	0.58848800
C	-1.67022600	-0.32890300	1.64447100
H	-1.54595300	-1.42228800	1.69957500
H	-2.38427800	-0.01778600	2.43981800
H	-0.69895200	0.12922900	1.89150700
C	2.91235700	-1.13456200	0.31611600
H	3.34004600	-1.84845900	1.04623400
H	3.36431100	-1.38311900	-0.66424600
C	-3.55670400	-0.51303300	0.03040400
H	-3.94526000	-0.25550300	-0.97079800
H	-4.28346700	-0.13854700	0.78173300
H	-3.52011900	-1.61284400	0.09582700
C	3.30502600	0.30122400	0.68876000
H	4.40537400	0.41122400	0.72660500
H	2.93366600	0.51942400	1.70892800
C	2.69110000	1.31281700	-0.28813500
H	3.13768800	1.15640900	-1.28950000
H	2.95760000	2.34417600	0.01398900

Bi(PCy*t*Bu)_singlet

Bi	2.02559100	-0.67098500	0.03692700
P	-0.11694600	0.44485800	-0.04738400
C	-0.65664600	2.84728700	1.34441400
H	-1.55506300	2.40820000	1.80112000
H	-0.79314400	3.94344400	1.32691500
H	0.20277000	2.62300400	1.99437600
C	-1.72437800	-0.52688800	0.06797100
H	-1.31896200	-1.51506400	0.34663300
C	-0.41698600	2.33740200	-0.09114400
C	-2.45788200	-0.70297900	-1.27904600
H	-2.84576900	0.26798800	-1.62776000
H	-1.74970700	-1.05634700	-2.04544100
C	-2.69417200	-0.09248600	1.18410200
H	-2.15918100	-0.01279800	2.14414500
H	-3.10083500	0.90858500	0.96146200
C	-1.61941100	2.68250200	-0.98711900
H	-1.48247600	2.31451800	-2.01558400
H	-1.72899300	3.77998100	-1.03595500

H	-2.56463500	2.27921200	-0.59749600
C	-3.62834800	-1.68808100	-1.13814700
H	-4.15913300	-1.77848200	-2.10083900
H	-3.22902400	-2.69201500	-0.90444200
C	0.84677000	3.00183700	-0.66001900
H	1.74021300	2.75498400	-0.06739200
H	0.71444000	4.09768000	-0.64854100
H	1.03862400	2.68902100	-1.69786000
C	-4.59578400	-1.25925600	-0.02842400
H	-5.41226800	-1.99320000	0.07581800
H	-5.07042700	-0.30094000	-0.31122200
C	-3.86344900	-1.08225700	1.30702700
H	-3.47296400	-2.06104700	1.64060500
H	-4.56163300	-0.74030800	2.08960000

Bi(PCy*t*Bu)_triplet

Bi	1.86636400	-0.73889100	-0.01810400
P	-0.19250500	0.70522300	-0.87436100
C	0.06690600	2.14428900	1.69654300
H	-0.67577400	1.50480500	2.19081100
H	0.04051400	3.13108900	2.19369900
H	1.06211400	1.70990700	1.88056300
C	-1.67724800	-0.38813900	-0.39974300
H	-1.33914700	-1.37379200	-0.77438100
C	-0.20836100	2.31521400	0.19327000
C	-2.92859900	-0.01972800	-1.22890400
H	-3.30415600	0.96914000	-0.91606100
H	-2.66147500	0.07118800	-2.29414300
C	-2.04426100	-0.56827400	1.08305600
H	-1.15570300	-0.86064100	1.66595500
H	-2.38949600	0.39520000	1.49519500
C	-1.57193700	3.00507700	-0.00321000
H	-1.82825800	3.11504800	-1.06889200
H	-1.54171600	4.01602300	0.44057800
H	-2.38666600	2.45605000	0.49139500
C	-4.04541700	-1.05819600	-1.04421200
H	-4.93639000	-0.75942800	-1.62217300
H	-3.71106100	-2.02586000	-1.46218000
C	0.89234700	3.20521900	-0.41815600
H	1.89119900	2.74953600	-0.31491000
H	0.91881100	4.17905200	0.10160900
H	0.71587300	3.39453100	-1.48857200
C	-4.40249000	-1.24579000	0.43499600
H	-5.18090800	-2.01888200	0.54933900
H	-4.83549200	-0.30546300	0.82470400
C	-3.16266100	-1.60653800	1.26170800
H	-2.78899500	-2.59753500	0.94372600
H	-3.42362800	-1.69913500	2.32973100

*t*BuCyP–PCy*t*Bu

C	0.27523900	2.41130200	-1.66295600
H	1.07294200	1.88746100	-2.20656000
H	0.24506100	3.45376700	-2.02853400
H	-0.67725500	1.93927100	-1.94504700
C	2.33723300	-0.05691200	0.28906400
H	2.16464000	-1.11666800	0.54171300
C	0.50978000	2.40570200	-0.14334000
C	3.42868900	0.41008500	1.27636200
H	3.68073900	1.46708800	1.09755800
H	3.05151000	0.34896300	2.31065300
C	2.85146100	-0.02649300	-1.16099100
H	2.07602700	-0.39696700	-1.84967800
H	3.06419000	1.01438700	-1.45730300
C	1.80508500	3.17954100	0.16909000
H	2.04000000	3.17170300	1.24534900
H	1.68008100	4.23388200	-0.13396300
H	2.67185900	2.78415100	-0.37748100
C	4.70429800	-0.43128800	1.11940100
H	5.48203500	-0.07128900	1.81419000
H	4.48745900	-1.47706200	1.40684200
C	-0.64312500	3.14873900	0.56653900
H	-1.62136800	2.67507600	0.40523600
H	-0.71287300	4.18123700	0.18126300
H	-0.47607300	3.20239500	1.65371500
C	5.22051000	-0.40288200	-0.32457400
H	6.12075600	-1.03247900	-0.42515800
H	5.53055500	0.62872200	-0.57615600

C	4.13701200	-0.85464100	-1.31118300
H	3.90307800	-1.92015100	-1.12931200
H	4.50799400	-0.79155300	-2.34831700
P	0.62573100	0.65953300	0.69550500
P	-0.62582800	-0.66182900	-0.69559800
C	-2.33680800	0.05648200	-0.28960800
C	-0.51055800	-2.40763700	0.14396400
H	-2.16280200	1.11565700	-0.54364700
C	-2.85090000	0.02847500	1.16050000
C	-3.42905200	-0.41024200	-1.27611700
C	0.63968700	-3.15277200	-0.56810900
C	-1.80767900	-3.18007400	-0.16482000
C	-0.27238400	-2.41257600	1.66303500
H	-3.06509800	-1.01180200	1.45791700
H	-2.07495100	0.39861500	1.84878400
C	-4.13520600	0.85869900	1.30989700
H	-3.05197000	-0.35131200	-2.31057100
H	-3.68271200	-1.46659600	-1.09560600
C	-4.70334800	0.43331100	-1.12015500
H	0.46957200	-3.20764200	-1.65474700
H	0.70940900	-4.18482600	-0.18165700
H	1.61885300	-2.67989700	-0.41020500
H	-2.67249400	-2.78351000	0.38395900
H	-1.68316800	-4.23449600	0.13808800
H	-2.04555900	-3.17221000	-1.24037700
H	0.68181300	-1.94239000	1.94243800
H	-0.24355200	-3.45477900	2.02941100
H	-1.06759500	-1.88646000	2.20808600
H	-4.50616400	0.79728500	2.34713900
H	-3.89966900	1.92365100	1.12685700
C	-5.21948800	0.40749400	0.32389700
H	-4.48490100	1.47837600	-1.40895000
H	-5.48169400	0.07363000	-1.81442600
H	-6.11872100	1.03865300	0.42377100
H	-5.53116700	-0.62330600	0.57674400

Ethylene

C	0.00000000	0.66664200	0.00000000
H	0.93115400	1.24262400	0.00000000
H	-0.93115600	1.24262400	0.00000000
C	0.00000000	-0.66664200	0.00000000
H	-0.93115400	-1.24262400	0.00000000
H	0.93115600	-1.24262400	0.00000000

1-Pentene

C	-1.19123500	-0.25527800	-0.39304200
H	-1.23365600	-0.49575100	-1.46472300
C	-2.10439200	0.57651500	0.11641700
H	-2.89426500	1.01213300	-0.50235200
H	-2.09783800	0.85032500	1.17724600
C	-0.06716300	-0.90152100	0.36850100
H	-0.17841000	-1.99986500	0.30281500
H	-0.14201100	-0.64093400	1.43923200
C	1.32876700	-0.51960600	-0.16113200
H	2.08369800	-1.14031600	0.35215400
H	1.39356700	-0.78766800	-1.23170800
C	1.66839000	0.96017100	0.02087200
H	0.94037900	1.60420200	-0.49802000
H	1.65175100	1.24128400	1.08766400
H	2.67058300	1.19490300	-0.37200200

1-Hexene

C	-1.76534200	0.03406300	0.45071600
H	-1.76279500	0.13577100	1.54512300
C	-2.52206600	-0.91620800	-0.10567500
H	-3.14284500	-1.58411000	0.49850800
H	-2.55122300	-1.05929000	-1.19150300
C	-0.87869000	0.99826300	-0.28789800
H	-1.22484200	2.02803100	-0.08139600
H	-0.98309200	0.84421700	-1.37643400
C	0.60502400	0.89415200	0.11104000
H	1.16125400	1.72475200	-0.35937200
H	0.70012600	1.04765600	1.20257900
C	1.26084700	-0.43518500	-0.27231300
H	0.69544800	-1.26536900	0.18561900
H	1.16885500	-0.57872700	-1.36474800

C	2.73131600	-0.52060600	0.13743600
H	2.84720500	-0.41587000	1.22957000
H	3.17919400	-1.48419800	-0.15403000
H	3.32619300	0.28026300	-0.33375100

9-H

Bi	0.98503500	-0.16337100	-0.98827300
P	0.13587500	-2.10071700	0.73961000
C	-2.17908000	0.54996200	-0.60655100
H	-2.18629400	-0.38313300	-0.02581300
H	-2.38484500	0.26826800	-1.64935000
P	2.98423000	1.02430100	0.44356700
P	-3.60757200	1.63461900	-0.01744700
C	-5.02139400	0.99545000	-1.16440200
C	-4.78366400	1.73889000	-2.49750800
H	-4.81298600	2.83138000	-2.36314900
H	-5.56559500	1.46247800	-3.22673300
H	-3.81154600	1.48159000	-2.94939500
C	-3.87569500	0.95978600	1.76923600
C	-4.37403800	-0.48813100	1.88590800
H	-3.71984200	-1.19731900	1.35502500
H	-5.39840800	-0.60709300	1.50502200
H	-4.38455200	-0.79270400	2.94773500
C	-2.51051600	1.06335400	2.48267200
H	-1.78938000	0.31692000	2.11731800
H	-2.64795700	0.87565100	3.56178100
H	-2.06428500	2.06551700	2.37776800
C	-0.51738500	-3.43748600	-0.52402800
C	0.55072000	-4.14150100	-1.37402500
H	1.14344600	-3.42622700	-1.96576400
H	0.06502600	-4.83549700	-2.08371600
H	1.24368800	-4.73670100	-0.76421800
C	-1.52176200	-2.75031200	-1.47009400
H	-1.05456200	-1.98684900	-2.11247300
H	-2.34675300	-2.27688400	-0.91977400
H	-1.96588900	-3.50557200	-2.14156600
C	-1.32018100	-4.48413900	0.28090700
H	-0.68964200	-5.09850400	0.93474000
H	-1.83343900	-5.16990800	-0.41696100
H	-2.08919500	-4.00330000	0.90588300
C	1.74244200	-2.80217500	1.57388700
C	1.45173200	-4.19603900	2.16248700
H	1.35535900	-4.96919900	1.38760300
H	0.53797600	-4.20288700	2.77743900
H	2.29246600	-4.49385800	2.81335100
C	3.00186500	-2.87466900	0.69139800
H	3.32409400	-1.87601500	0.36348000
H	2.86622900	-3.50818800	-0.19416600
H	3.83642200	-3.30088300	1.27750300
C	2.02335500	-1.84340300	2.74762800
H	2.87041800	-2.22674200	3.34341800
H	1.15260800	-1.74542100	3.41496700
H	2.30813700	-0.84595800	2.38939800
C	3.96126300	1.78420900	-1.05254100
C	3.13278000	2.51062000	-2.12753800
H	3.80256100	2.89757800	-2.91679800
H	2.57255100	3.36297300	-1.72237300
H	2.41494800	1.83705000	-2.62187100
C	5.04467900	2.74419900	-0.52663700
H	5.74693500	2.98687000	-1.34374900
H	5.62817300	2.29930600	0.29461300
H	4.61913800	3.69444100	-0.17412300
C	4.67043500	0.57451800	-1.69935600
H	5.29131800	0.91478300	-2.54676400
H	3.95672700	-0.16554100	-2.09695600
H	5.32695500	0.05867400	-0.98136400
C	2.27379700	2.44772300	1.54541800
C	1.26515500	1.79339600	2.51355300
H	1.75944700	1.07667200	3.18518600
H	0.45304900	1.25950500	2.00552100
H	0.80609800	2.57288100	3.14606100
C	3.43020300	2.98908600	2.41841300
H	4.16062200	3.57436200	1.84614800
H	3.96824500	2.17453700	2.92799100
H	3.01467000	3.65545500	3.19524800
C	-6.38213900	1.44003600	-0.59982100
H	-7.17030400	1.27984500	-1.35647500

H	-6.66765700	0.86427200	0.29279300
H	-6.38684200	2.50968200	-0.33500800
C	-4.85780400	1.90919200	2.48463900
H	-5.86738400	1.87736500	2.05189800
H	-4.94650400	1.62326300	3.54769500
H	-4.50652800	2.95243500	2.44396400
C	-5.05729100	-0.51731600	-1.44819800
H	-4.13086400	-0.87730500	-1.91905700
H	-5.22967100	-1.11497500	-0.54466900
H	-5.88039600	-0.73966700	-2.15091600
C	1.61593300	3.62021300	0.80259700
H	0.80362100	3.29795000	0.13867200
H	1.18730200	4.33081200	1.53208600
H	2.34608200	4.17995500	0.20058100
C	-0.82674500	1.24212400	-0.51176300
H	-0.64744300	1.65911100	0.48282000
H	-0.74144200	2.06601100	-1.23751300

9-*n*Pr

Bi	1.04219300	-0.14777900	-0.94209400
C	-0.78028300	1.25344100	-0.32598800
H	-0.56901700	1.53930900	0.71149300
P	0.12150200	-2.37447500	0.33483200
C	-2.09641400	0.48143000	-0.42195500
H	-1.97059200	-0.55278400	-0.07407800
H	-2.39163700	0.41388100	-1.47936400
P	3.05253400	0.67580100	0.71371700
P	-3.56453800	1.19910500	0.53838900
C	-5.00590800	0.75732000	-0.66550200
C	-4.95023900	1.86271900	-1.74311400
H	-5.07881200	2.86423900	-1.30378200
H	-5.75668600	1.70877200	-2.48170800
H	-3.99657600	1.85743700	-2.29583500
C	-3.62107100	0.03284100	2.06904700
C	-3.94372200	-1.44443800	1.80155800
H	-3.25592100	-1.89502600	1.06946900
H	-4.97581900	-1.58583200	1.44961100
H	-3.83618900	-2.02251700	2.73677500
C	-2.22100300	0.12440900	2.71117900
H	-1.45672300	-0.39657900	2.11693900
H	-2.23926500	-0.35921000	3.70337500
H	-1.90520900	1.16980800	2.85922200
C	-0.46864300	-3.43432400	-1.19208200
C	0.64094000	-3.98381400	-2.10046700
H	1.27432700	-3.18032400	-2.50819400
H	0.19191900	-4.51906700	-2.95666900
H	1.29078600	-4.69883100	-1.57781000
C	-1.41335800	-2.56038800	-2.04090800
H	-0.90359000	-1.70045800	-2.50417800
H	-2.26048100	-2.18100400	-1.45306500
H	-1.83026000	-3.16680800	-2.86371600
C	-1.32319900	-4.59967400	-0.64489800
H	-0.73555300	-5.33472200	-0.08180100
H	-1.79703000	-5.13528400	-1.48713300
H	-2.12604100	-4.23175000	0.01331400
C	1.66972200	-3.24124100	1.11609500
C	1.32999800	-4.71090400	1.42986900
H	1.27357600	-5.33177500	0.52478700
H	0.38014900	-4.80786700	1.97905200
H	2.12671100	-5.13662600	2.06485600
C	2.97732800	-3.18299800	0.30588200
H	3.32537300	-2.14891400	0.17333600
H	2.88712500	-3.65265400	-0.68165100
H	3.77282600	-3.71908000	0.85500300
C	1.88824200	-2.50842900	2.45444600
H	2.69863200	-3.00239100	3.01892300
H	0.98137900	-2.51695900	3.07960900
H	2.19667300	-1.46746600	2.29346100
C	4.12973600	1.60723000	-0.60628900
C	3.39273200	2.56279600	-1.56193000
H	4.11698200	3.03169300	-2.25242600
H	2.87249700	3.37069200	-1.03131700
H	2.65378100	2.03554500	-2.18477900
C	5.25332000	2.38438100	0.10501000
H	5.99803100	2.71529700	-0.64039200
H	5.77778800	1.76447000	0.84892100
H	4.87787600	3.28660300	0.60843900

C	4.78225300	0.47847000	-1.43386800
H	5.45503200	0.91362100	-2.19376300
H	4.03675100	-0.13042900	-1.97092600
H	5.37674300	-0.19714900	-0.79922900
C	2.40730100	1.93714000	2.03194900
C	1.31027400	1.20924200	2.83338900
H	1.70418500	0.32153600	3.34924300
H	0.46989600	0.88401200	2.21016900
H	0.90806600	1.88703900	3.60623300
C	3.56554100	2.21851100	3.01697200
H	4.36427500	2.82758200	2.57577600
H	4.01412200	1.28475600	3.39039300
H	3.17290100	2.77501300	3.88664100
C	-0.67070600	2.48106100	-1.24024800
H	-0.96516400	2.19823400	-2.26901300
H	0.38550900	2.79585700	-1.31839200
C	-1.48052400	3.71473800	-0.80994700
H	-2.55417700	3.47441500	-0.78983800
H	-1.22065100	3.96751800	0.23319900
C	-1.22907000	4.91890400	-1.71796600
H	-1.50820400	4.69621600	-2.76200500
H	-1.81229300	5.79705500	-1.39763000
H	-0.16353500	5.20649000	-1.71787100
C	-6.34694300	0.87646200	0.07991000
H	-7.17967100	0.85839500	-0.64507700
H	-6.50622500	0.04158900	0.77800300
H	-6.41871600	1.81818400	0.64750000
C	-4.63918000	0.61780300	3.06856500
H	-5.67337000	0.56182000	2.70194000
H	-4.59592300	0.05302900	4.01657000
H	-4.41761600	1.67305000	3.29420700
C	-4.93647700	-0.61148300	-1.36682000
H	-4.04378600	-0.71102900	-2.00095800
H	-4.94408400	-1.45154500	-0.66131300
H	-5.81244400	-0.72943600	-2.02971100
C	1.86394700	3.26902900	1.49630400
H	1.04328300	3.12780200	0.78237400
H	1.47550500	3.87757000	2.33268900
H	2.64647100	3.86276800	1.00271400

9-*n*Bu

Bi	1.10430200	-0.11107200	-0.93678000
C	-0.86161900	1.03404200	-0.24023300
H	-0.67190800	1.29372800	0.80819500
P	0.46962400	-2.48784500	0.23912900
C	-2.08019800	0.11901800	-0.36273100
H	-1.83015900	-0.90849600	-0.06596200
H	-2.37996800	0.06696400	-1.41972100
P	3.02217200	0.86919100	0.74243400
P	-3.60803900	0.61483500	0.64397100
C	-5.00539100	0.07596700	-0.57220000
C	-5.08711300	1.23415300	-1.59124900
H	-5.32046400	2.19143300	-1.09947300
H	-5.88110400	1.02885400	-2.33083500
H	-4.14619500	1.36365300	-2.15056200
C	-3.51279000	-0.62770300	2.11102600
C	-3.66720600	-2.11656100	1.76828900
H	-2.94285000	-2.44632200	1.00762600
H	-4.68112600	-2.35768600	1.41763700
H	-3.48084900	-2.72604100	2.67059400
C	-2.12480500	-0.40812600	2.74861900
H	-1.31264400	-0.80557200	2.12324300
H	-2.07519700	-0.94123200	3.71402300
H	-1.92995300	0.65777300	2.94962100
C	-0.01370200	-3.53732200	-1.33187800
C	1.13991500	-3.91024600	-2.27444100
H	1.66986300	-3.02001200	-2.64813300
H	0.74468900	-4.45369000	-3.15174200
H	1.87561900	-4.56803000	-1.79200500
C	-1.06538100	-2.74192000	-2.13078300
H	-0.66534600	-1.80832000	-2.55779300
H	-1.94272000	-2.49092900	-1.51882500
H	-1.42035200	-3.35488000	-2.97744800
C	-0.71876300	-4.81889700	-0.83373200
H	-0.04182500	-5.50467200	-0.31010300
H	-1.13740600	-5.36744600	-1.69662700
H	-1.55060900	-4.57846600	-0.15299900

C	2.12030100	-3.20130600	0.96388000
C	1.96069700	-4.71410200	1.20848600
H	1.96407300	-5.29374700	0.27478100
H	1.03723800	-4.94859400	1.76116800
H	2.81141000	-5.07226700	1.81445400
C	3.40006000	-2.94996100	0.14613300
H	3.62168200	-1.87674400	0.06140000
H	3.35161000	-3.37934100	-0.86244200
H	4.26111800	-3.41349800	0.66134500
C	2.27032900	-2.51238900	2.33464600
H	3.14070900	-2.93416300	2.86748000
H	1.37953800	-2.65722000	2.96616800
H	2.45271800	-1.43589000	2.22255700
C	3.96226300	1.98642900	-0.53748800
C	3.10330900	2.89033400	-1.44005000
H	3.75690500	3.47616900	-2.11146400
H	2.49590100	3.60284500	-0.86706600
H	2.42584300	2.30774100	-2.08314800
C	4.99283000	2.85893600	0.20347400
H	5.68247100	3.31319800	-0.52985600
H	5.59784700	2.27207300	0.91220600
H	4.51774000	3.68371900	0.75342200
C	4.73558900	0.98594300	-1.42389800
H	5.34120800	1.53523800	-2.16614200
H	4.06214500	0.31766000	-1.98499700
H	5.41526500	0.35770800	-0.82723800
C	2.24700800	1.97805300	2.12612400
C	1.25605900	1.08495500	2.89825200
H	1.76102000	0.22826400	3.36769500
H	0.45354600	0.68970600	2.26559900
H	0.78438000	1.67133500	3.70580400
C	3.37572000	2.34918000	3.11573900
H	4.08918900	3.07146800	2.69997400
H	3.93861500	1.45954300	3.43861900
H	2.93027000	2.81076500	4.01501800
C	-0.90931400	2.30757400	-1.09505200
H	-1.17943300	2.03989800	-2.13427000
H	0.10050300	2.75000800	-1.16357000
C	-1.85660400	3.41052700	-0.60006600
H	-2.89459300	3.04354100	-0.58703300
H	-1.62017800	3.64430300	0.45391500
C	-1.77277700	4.68683100	-1.44079200
H	-1.99643100	4.44184200	-2.49558000
H	-0.73230000	5.06171300	-1.43491400
C	-6.34208300	0.00542900	0.18704800
H	-7.17646800	-0.06869000	-0.53245400
H	-6.39845400	-0.87686700	0.84134800
H	-6.51157500	0.90267100	0.80380300
C	-4.57908700	-0.21622300	3.14614600
H	-5.60439900	-0.37265500	2.78370800
H	-4.45938300	-0.81991000	4.06308800
H	-4.47772500	0.84465000	3.42512000
C	-4.79180200	-1.23899300	-1.34390300
H	-3.89982200	-1.20652400	-1.98601100
H	-4.69931500	-2.10936800	-0.68254300
H	-5.65587600	-1.41974000	-2.00824700
C	1.54066800	3.25922600	1.66136700
H	0.73292300	3.05520300	0.94795900
H	1.09382700	3.77558800	2.52996400
H	2.23959600	3.96583000	1.19157900
C	-2.71919600	5.78629100	-0.95750100
H	-2.64202100	6.69438400	-1.57698400
H	-3.76847100	5.44760900	-0.98728400
H	-2.49729700	6.07135300	0.08469100

References

1 Á. Sinai, D. C. Simkó, F. Szabó, A. Paczal, T. Gáti, A. Bényei, Z. Novák and A. Kotschy, *Eur. J. Org. Chem.*, 2020, **2020**, 1122–1128.

2 J. Meiners, A. Friedrich, E. Herdtweck and S. Schneider, *Organometallics*, 2009, **28**, 6331–6338.

3 C.-J. Li, J. Lü, Z.-X. Zhang, K. Zhou, Y. Li and G.-H. Qi, *Res. Chem. Intermed.*, 2018, **44**, 4547–4562.

4 C. A. Busacca, J. C. Lorenz, N. Grinberg, N. Haddad, M. Hrapchak, B. Latli, H. Lee, P. Sabila, A. Saha, M. Sarvestani, S. Shen, R. Varsolona, X. Wei and C. H. Senanayake, *Org. Lett.*, 2005, **7**, 4277–4280.

5 A. Dashti-Mommertz and B. Neumüller, *Z. Anorg. Allg. Chem.*, 1999, **625**, 954–960.

6 L. Wu, V. T. Annibale, H. Jiao, A. Brookfield, D. Collison and I. Manners, *Nat. Commun.*, 2019, **10**, 2786.

7 W. McFarlane and C. T. Regius, *Polyhedron*, 1997, **16**, 1855–1861.

8 F. Brunner, A. Babaei, A. Pertegás, J. M. Junquera-Hernández, A. Prescimone, E. C. Constable, H. J. Bolink, M. Sessolo, E. Ortí and C. E. Housecroft, *Dalton Trans.*, 2019, **48**, 446–460.

9 A. I. Arkhypchuk, T. T. Tran, R. Charaf, L. Hammarström and S. Ott, *Inorg. Chem.*, 2023, **62**, 18391–18398.

10 L. Meca, D. Reha and Z. Havlas, *J. Org. Chem.*, 2003, **68**, 5677–5680.

11 P. R. Blakemore, C. Kilner and S. D. Milicevic, *J. Org. Chem.*, 2006, **71**, 8212–8218.

12 J. Jeener, B. H. Meier, P. Bachmann and R. R. Ernst, *J. Chem. Phys.*, 1979, **71**, 4546–4553.

13 G. Fischer and E. Kleinpeter, *Magn. Reson. Chem.*, 1991, **29**, 204–206.

14 C. L. Perrin and T. J. Dwyer, *Chem. Rev.*, 1990, **90**, 935–967.

15 P. W. Kuchel, B. T. Bulliman and B. E. Chapman, *Biophys. Chem.*, 1988, **32**, 89–95.

16 Y. Huang, S. Macura and R. R. Ernst, *J. Am. Chem. Soc.*, 1981, **103**, 5327–5333.

17 J. E. Münzer, G. H. Sieg, R. Vehlies, P. A. Fuzon, X. Xie, B. Neumüller and I. Kuzu, *Polyhedron*, 2021, **196**, 115014.

18 M. Klein, X. Xie, O. Burghaus and J. Sundermeyer, *Organometallics*, 2019, **38**, 3768–3777.

19 J. F. Kögel, X. Xie, E. Baal, D. Gesevičius, B. Oelkers, B. Kovačević and J. Sundermeyer, *Chem. Eur. J.*, 2014, **20**, 7670–7685.

20 B. Song, T. Storr, S. Liu and C. Orvig, *Inorg. Chem.*, 2002, **41**, 685–692.

21 T. Gädt, B. Grau, K. Eichele, I. Pantenburg and L. Wesemann, *Chem. Eur. J.*, 2006, **12**, 1036–1045.

22 S. Freye, D. M. Engelhard, M. John and G. H. Clever, *Chem. Eur. J.*, 2013, **19**, 2114–2121.

23 C. Zhao, M. Devany and N. L. Greenbaum, *Biochem. Biophys. Res. Commun.*, 2014, **453**, 692–695.

24 D. Li, C. Sun and P. G. Williard, *J. Am. Chem. Soc.*, 2008, **130**, 11726–11736.

25 G. Hilmersson and B. Malmros, *Chem. Eur. J.*, 2001, **7**, 337–341.

26 W. Scherer, V. Herz, A. Brück, C. Hauf, F. Reiner, S. Altmannshofer, D. Leusser and D. Stalke, *Angew. Chem. Int. Ed.*, 2011, **50**, 2845–2849.

27 J. Huber and S. Mecking, *Macromolecules*, 2010, **43**, 8718–8723.

28 R. G. Agarwal, S. C. Coste, B. D. Groff, A. M. Heuer, H. Noh, G. A. Parada, C. F. Wise, E. M. Nichols, J. J. Warren and J. M. Mayer, *Chem. Rev.*, 2022, **122**, 1–49.

29 S. Stoll and A. Schweiger, *J. Magn. Reson.*, 2006, **178**, 42–55.

30 D. L. Haire, E. G. Janzen, V. J. Robinson and I. Hrvoic, *Magn. Reson. Chem.*, 2004, **42**, 835–843.

31 D. L. Haire, U. M. Oehler, P. H. Krygsman and E. G. Janzen, *J. Org. Chem.*, 1988, **53**, 4535–4542.

32 M. J. Frisch, G. W. Trucks, H. B. Schlegel, G. E. Scuseria, M. A. Robb, J. R. Cheeseman, G. Scalmani, V. Barone, G. A. Petersson, H. Nakatsuji, X. Li, M. Caricato, A. V. Marenich, J. Bloino, B. G. Janesko, R. Gomperts, B. Mennucci, H. P. Hratchian, J. V. Ortiz, A. F. Izmaylov, J. L. Sonnenberg, D. Williams-Young, F. Ding, F. Lipparini, F. Egidi, J. Goings, B. Peng, A. Petrone, T. Henderson, D. Ranasinghe, V. G. Zakrzewski, J. Gao, N. Rega, G. Zheng, W. Liang, M. Hada, M. Ehara, K. Toyota, R. Fukuda, J. Hasegawa, M. Ishida, T. Nakajima, Y. Honda, O. Kitao, H. Nakai, T. Vreven, K. Throssell, Montgomery, J. A., Jr., J. E. Peralta, F. Ogliaro, M. J. Bearpark, J. J. Heyd, E. N. Brothers, K. N. Kudin, V. N. Staroverov, T. A. Keith, R. Kobayashi, J. Normand, K. Raghavachari, A. P. Rendell, J. C. Burant, S. S. Iyengar, J. Tomasi, M. Cossi, J. M. Millam, M. Klene, C. Adamo, R. Cammi, J. W. Ochterski, R. L. Martin, K. Morokuma, O. Farkas, J. B. Foresman and D. J. Fox, *Gaussian16*, **2016**.

33 Y. Zhao and D. G. Truhlar, *Acc. Chem. Res.*, 2008, **41**, 157–167.

34 F. Weigend and R. Ahlrichs, *Phys. Chem. Chem. Phys.*, 2005, **7**, 3297–3305.

35 F. Weigend, *Phys. Chem. Chem. Phys.*, 2006, **8**, 1057–1065.

36 S. H. Vosko, L. Wilk and M. Nusair, *Can. J. Phys.*, 1980, **58**, 1200–1211.

37 A. V. Marenich, C. J. Cramer and D. G. Truhlar, *J. Phys. Chem. B*, 2009, **113**, 6378–6396.

38 P. J. Stephens, F. J. Devlin, C. F. Chabalowski and M. J. Frisch, *J. Phys. Chem.*, 1994, **98**, 11623–11627.

39 C. Lee, W. Yang and R. G. Parr, *Phys. Rev. B*, 1988, **37**, 785–789.

40 A. D. Becke, *J. Chem. Phys.*, 1993, **98**, 5648–5652.

41 O. V. Dolomanov, A. J. Blake, N. R. Champness and M. Schröder, *J Appl Cryst*, 2003, **36**, 1283–1284.

42 O. V. Dolomanov, L. J. Bourhis, R. J. Gildea, J. A. K. Howard and H. Puschmann, *J. Appl. Crystallogr.*, 2009, **42**, 339–341.

43 L. J. Farrugia, *J. Appl. Crystallogr.*, 1999, **32**, 837–838.

44 P. W. Betteridge, J. R. Carruthers, R. I. Cooper, K. Prout and D. J. Watkin, *J. Appl. Crystallogr.*, 2003, **36**, 1487.

45 W. Schmitz, *Cryst. Res. Technol.*, 1975, **10**. DOI: 10.1002/crat.19750101116.

46 D. Kratzert, J. J. Holstein and I. Krossing, *J. Appl. Crystallogr.*, 2015, **48**, 933–938.
